# Noninvasive imaging signatures of HER2 and HR using ADC in invasive breast cancer: repeatability, reproducibility, and association with pathological complete response to neoadjuvant chemotherapy

**DOI:** 10.1186/s13058-023-01674-9

**Published:** 2023-06-28

**Authors:** Xinzhi Teng, Jiang Zhang, Xinyu Zhang, Xinyu Fan, Ta Zhou, Yu-hua Huang, Lu Wang, Elaine Yuen Phin Lee, Ruijie Yang, Jing Cai

**Affiliations:** 1 Department of Health Technology and Informatics, The Hong Kong Polytechnic University, Hung Hom, Kowloon, Hong Kong, China; 2 Department of Diagnostic Radiology, Li Ka Shing Faculty of Medicine, The University of Hong Kong, Y920, Lee Shau Kee Building, Hong Kong, China; 3 Department of Radiation Oncology, Peking University Third Hospital, Beijing, China; 4 Department of Applied Mathematics, The Hong Kong Polytechnic University, Hong Kong, China; 5 The Hong Kong Polytechnic University Shenzhen Research Institute, Hong Kong, China; 6 Research Institute for Smart Aging, The Hong Kong Polytechnic University, Hong Kong, China

**Keywords:** Immunohistochemistry, HER2, HR, Image signatures, ADC

## Abstract

**Background:**

The immunohistochemical test (IHC) of HER2 and HR can provide prognostic information and treatment guidance for invasive breast cancer patients. We aimed to develop noninvasive image signatures IS_HER2_ and IS_HR_ of HER2 and HR, respectively. We independently evaluate their repeatability, reproducibility, and association with pathological complete response (pCR) to neoadjuvant chemotherapy.

**Methods:**

Pre-treatment DWI, IHC receptor status HER2/HR, and pCR to neoadjuvant chemotherapy of 222 patients from the multi-institutional ACRIN 6698 trial were retrospectively collected. They were pre-separated for development, independent validation, and test–retest. 1316 image features were extracted from DWI-derived ADC maps within manual tumor segmentations. IS_HER2_ and IS_HR_ were developed by RIDGE logistic regression using non-redundant and test–retest reproducible features relevant to IHC receptor status. We evaluated their association with pCR using area under receiver operating curve (AUC) and odds ratio (OR) after binarization. Their reproducibility was further evaluated using the test–retest set with intra-class coefficient of correlation (ICC).

**Results:**

A 5-feature IS_HER2_ targeting HER2 was developed (AUC = 0.70, 95% CI 0.59 to 0.82) and validated (AUC = 0.72, 95% CI 0.58 to 0.86) with high perturbation repeatability (ICC = 0.92) and test–retest reproducibility (ICC = 0.83). IS_HR_ was developed using 5 features with higher association with HR during development (AUC = 0.75, 95% CI 0.66 to 0.84) and validation (AUC = 0.74, 95% CI 0.61 to 0.86) and similar repeatability (ICC = 0.91) and reproducibility (ICC = 0.82). Both image signatures showed significant associations with pCR with AUC of 0.65 (95% CI 0.50 to 0.80) for IS_HER2_ and 0.64 (95% CI 0.50 to 0.78) for IS_HER2_ in the validation cohort. Patients with high IS_HER2_ were more likely to achieve pCR to neoadjuvant chemotherapy with validation OR of 4.73 (95% CI 1.64 to 13.65, *P* value = 0.006). Low IS_HR_ patients had higher pCR with OR = 0.29 (95% CI 0.10 to 0.81, *P* value = 0.021). Molecular subtypes derived from the image signatures showed comparable pCR prediction values to IHC-based molecular subtypes (*P* value > 0.05).

**Conclusion:**

Robust ADC-based image signatures were developed and validated for noninvasive evaluation of IHC receptors HER2 and HR. We also confirmed their value in predicting treatment response to neoadjuvant chemotherapy. Further evaluations in treatment guidance are warranted to fully validate their potential as IHC surrogates.

**Supplementary Information:**

The online version contains supplementary material available at 10.1186/s13058-023-01674-9.

## Introduction

Breast cancer is one of the most common malignant neoplasm in women, and 2.3 million women were diagnosed with breast cancer in 2020 [1]. The hormone receptor (HR) and the human epidermal growth receptor (HER2) derived from immunohistochemistry (IHC) are two routinely measured biomarkers for prognosis and treatment decision. Breast cancer patients with positive HR status (HR+) are suggested to receive endocrine therapy, and patients with positive HER2 status (HER2+) are suggested to receive HER2-targeted therapy, according to the 2021 ASCO guideline for optimized neoadjuvant therapy [2]. For patients with negative HR and HER2 status or triple-negative breast cancer, chemotherapy alone is suggested. In addition to treatment guidance, HR and HER2 status also play an important role in the development of experimental agents [3]. For example, the pan-Akt inhibitor MK-2206 [4] and the poly (ADP-ribose) polymerase (PARP) inhibitor Veliparib combined with carboplatin [5] showed a significant improvement in pathological complete response (pCR) in one or more signatures defined by HER2 and HR. The measurement of HR and HER2 status, however, requires an invasive percutaneous biopsy, and therefore, a noninvasive measurement of receptor status would be desirable.

The apparent diffusion coefficient (ADC) derived from diffusion-weighted imaging (DWI), as a noninvasive imaging technique, quantitatively measures water diffusion in tissues, and the absolute ADC value has been proposed as a biomarker in differentiating malignancies of breast tumor [6]. However, as confirmed by a meta-analysis by Meyer et al., there is no significant difference in mono-exponential ADC values between breast cancer subtypes [7]. On the other hand, radiomics, which is a more sophisticated image characterization method for tumor phenotyping based on high-throughput feature extraction and advanced machine learning algorithms[8–12], has shown great potential to predict molecular subtypes in breast cancer patients. Baysal et al. predicted breast cancer molecular subtypes with ADC radiomics features using a neural network [13]. Leighner et al. also observed a significant association between ADC-based radiomic signatures with breast cancer receptor status and molecular subtypes [14]. The clinical implementation of ADC-based image signatures requires further assessments of their repeatability [15–17], reproducibility, and clinical utility.

The purpose of this study was to provide reliable noninvasive ADC-based assessments of IHC-derived HER2 and HR receptor status, namely IS_HER2_ and IS_HR_, respectively, and investigate their potential in treatment response prediction to neoadjuvant chemotherapy. This was achieved through three objectives: (1) to develop and independently validate the association of IS_HER2_ and IS_HR_ with HER2 and HR status, (2) to evaluate their repeatability and reproducibility with a test–retest dataset combined with the perturbation method, and (3) to evaluate their association with pCR after neoadjuvant chemotherapy.

## Materials and methods

### Patient data

We collected 222 patients from the publicly available BMMR2 challenge dataset [18–20] derived from the ACRIN 6698 trial, where female patients with invasive breast cancer were prospectively enrolled from ten institutions between 2012 and 2015. Institutional review board (IRB) approval was waived due to the sole use of public data. The patients eligible for the research included 25- to 77-year-old women with invasive breast tumors of 0.44 to 15 cm on clinical examination or imaging, who were scheduled for neoadjuvant chemotherapy. Patients with evidence of distant metastases were excluded. The discovery (*n* = 117), validation (*n* = 74), and test–retest cohort (*n* = 71, 40 overlap with discovery) were adopted, the same as the BMMR2 challenge (Additional file 1: Figure S1).

### Pathology data

The receptor status of HER2 and HR was collected as the targets of signature building. They were determined with pre-treatment core biopsy by IHC or fluorescence in situ hybridization (FISH). HR positivity (estrogen receptor positivity or progesterone receptor positivity) was defined as at least 5% positive tumor staining, and HER2 positivity was determined by IHC 3 + or FISH overexpression [21].

The pathologic complete response (pCR) was also collected as a surrogate for treatment response to neoadjuvant chemotherapy. It was defined as the elimination of tumor in the breast and axillary lymph nodes at surgery (ypT0/is, ypN0), which has been validated as an independent predictor of event-free survival and overall survival [22].

### Imaging data and tumor segmentation

The image data were used to extract the image features for radiomics signature development. Pre-treatment DWI-derived ADC maps and manual tumor segmentations were downloaded from The Cancer Imaging Archive [20] in DICOM format. The ADC maps were derived from DWI acquired with four *b* values (*b* = 0, 100, 600, 800 s/mm^2^), and tumor segmentations were manually defined in the region with hyperintensity at high-b-value DWI (on *b* = 800 s/mm^2^ images) and relatively low ADC value in ACRIN 6698 trial [19]. The biopsy clip artifacts, adjacent adipose, fibroglandular tissue, and high-T2 signals (necrotic or hemorrhagic area) were excluded from the segmentations. Furthermore, the DWI test–retest data in pre-treatment or early treatment were also collected for image signature reproducibility validation. The detailed image acquisition parameters and tumor definition are listed in Additional file 1.

### Image feature extraction

A comprehensive set of radiomics features was extracted from the original and filtered ADC maps within the tumor volume. Filters include Log-sigma filters with sigma value of 1 mm, 3 mm, and 5 mm, and eight Coiflet1 wavelet filters (LLL, HLL, LHL, LLH, LHH, HLH, HHL, and HHH). All the images were preprocessed by isotropic resampling (1 × 1 × 1 mm) for better repeatability and 32-bin-number discretization before feature extraction for noise suppression. Both first-order (*n* = 18) and texture features (*n* = 70) were extracted from each preprocessed image within tumor segmentation, and shape features (*n* = 14) were extracted from the tumor segmentation. The definitions and extraction of radiomic features follow the standardization by the Image Biomarker Standardization Initiative. In total, 1316 radiomics features were extracted for each patient. Detailed settings of the image preprocessing and feature extraction parameters are listed in Additional file 1: Table S1 and Table S2.

### Image signature development

**Fig. 1:**
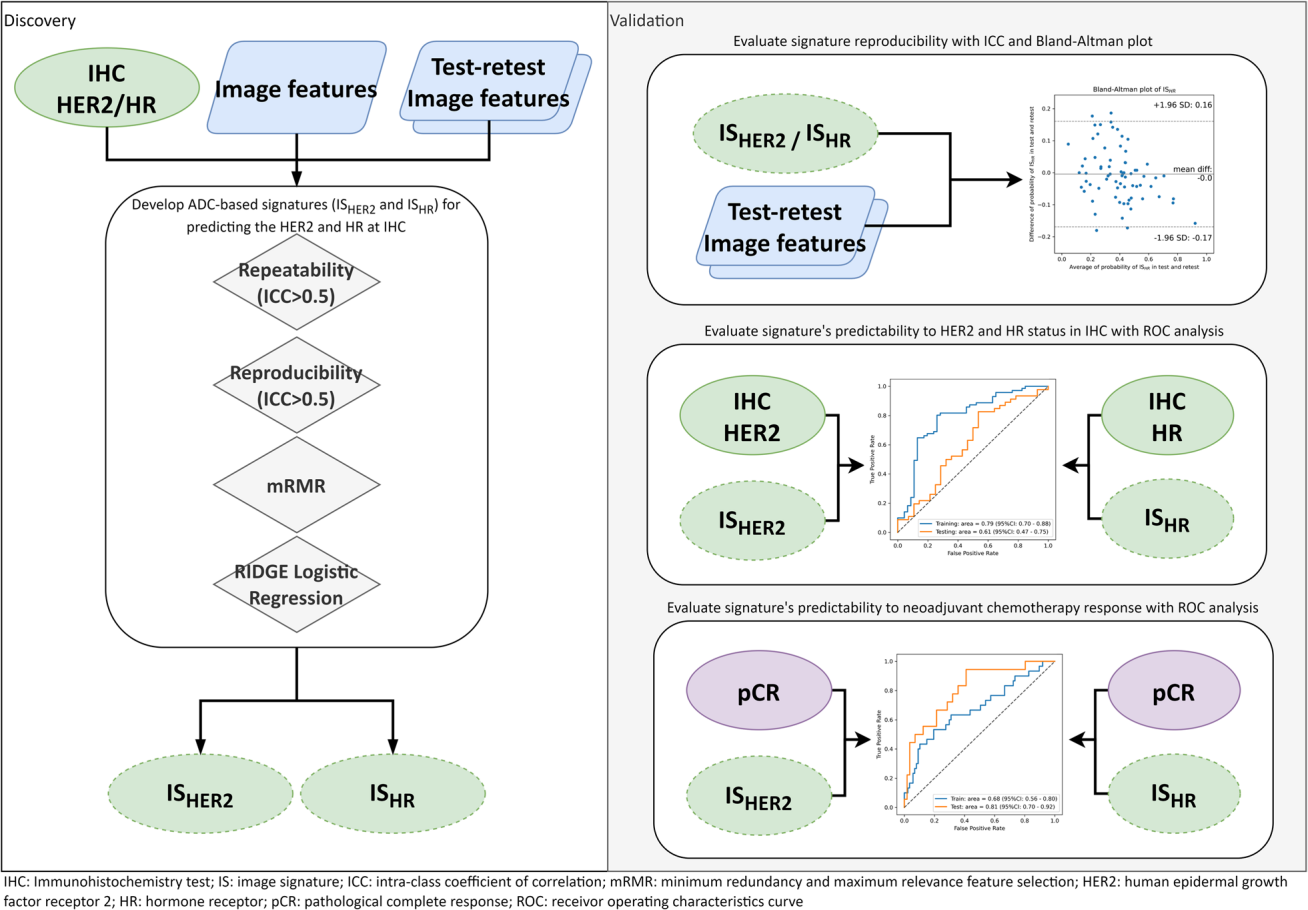
The study workflow showing the discovery and validation steps

The image signature (IS) construction was carried out by image feature selection and signature building in the discovery cohort. We first removed low repeatable and low reproducible features for enhanced generalizability and robustness of image signature [16]. The repeatability of the image features was evaluated using the perturbation method [23], and the reproducibility of image features was evaluated using test–retest images [24]. Secondly, we identified relevant features which were strongly correlated with the IHC receptor status [25]. Third, we adopted the minimum redundancy and maximum relevance (mRMR) feature selection algorithm [26] to rank the features based on both redundancy and outcome relevancy. The optimal feature number for signature building was determined by maximizing the validation performance in a threefold cross-validation. Finally, the signature was established by fitting the final selected features with RIDGE logistic regression using IHC-derived receptors status as the targets. The workflow of image signature development is shown in Fig. 1.

### Image signature evaluation

We evaluated various properties of the developed image signatures, including the association with IHC-derived receptors status, molecular subtypes, and treatment response (pCR) in the discovery and unseen validation cohort. Their repeatability and reproducibility were also measured in the validation and test–retest cohort, respectively. The area under the receiver operating curve (AUC) was used to evaluate the association between image signatures and IHC-derived receptors status. The agreement between the predicted and actual probability of the receptor status was visualized by a calibration curve [27] and quantified by Briers score $BS=\frac{1}{N}\sum_{i=1}^{N}{\left({p}_{i}-{o}_{i}\right)}^{2}$, where *N* is the number of samples, $p$ is the predicted probability of image signature, and $o$ is the target status. The associations between image signatures and pCR were evaluated by AUC as well. To acquire the binarized image signature status, the cutoff values were selected by maximizing Youden’s J statistic which is the sum of sensitivity and specificity [28]. With the dichotomized image signatures, the odds ratios (OR) were calculated to quantify the association between dichotomized signatures and pCR. Furthermore, we evaluated the accuracy of subtypes based on the image signatures compared to IHC molecular subtypes, namely HER2 + HR + , HER2 + HR-, HER2-HR+, HER2-HR-.

In addition to the diagnostic and predictive performances, the variabilities of the image signatures were assessed under random conditions. We evaluated the repeatability of signature values under image perturbations by the one-way random effects intra-class coefficient of correlation ICC (1,1). The perturbed images were generated from the test–retest cohort by adding translations and rotations on the images and randomizations to tumor segmentations. Details of image perturbations can be found in Additional file 1. Additional file 1: Figure S3 and Figure S4 visualize randomized tumor segmentation and image perturbation separately. Meanwhile, the reproducibility of image signatures was evaluated by the two-way mixed effects absolute agreement ICC (2,1) in the test–retest dataset [29], which measures feature value consistency between test and retest scans. The workflow of image signature evaluation is shown in Fig. 1.

### Statistical analysis and software

During statistical comparisons, we used the chi-square test when total number of categories exceeds 5 and the Fisher’s exact test when less than 5. The Student’s *t* test was used to compare the means of variables. Differences with *P* value < 0.05 (two-tailed) were considered statistically significant. The 95% confidence interval (95% CI) of the estimated AUC was derived from variance using the DeLong method. The DeLong test was also used to compare the AUCs of two models. The OR was calculated by Fisher’s exact test.

The primary analysis was carried out on Python 3.8 [30] and validated independently on *R*. The radiomic-based features were extracted using package PyRadiomics 3.0 [31] which the feature definition is compliant to Image Biomarker Standardization Initiative [32]. The RIDGE logistic regression was carried out with package scikit-learn 0.23 [33].

## Results

### Patient characteristics

Table 1 shows the patient clinical characteristics in discovery (*N* = 117) and validation (*N* = 74). No statistically significant differences were observed in race, lesion type, IHC receptor status, SBR grade, pCR, and treatment arm, while the MRI measured longest diameter (MRLD) (cm) at baseline study was slightly different (4.02 cm vs. 4.73 cm, *P* value = 0.049). Patients’ characteristics comparison between receptor status is also tabulated in Additional file 1: Table S3 and Table S4. There were no significant differences in the receptor status of HER2 and HR between the discovery and validation groups (*P* value = 0.974 and 0.959, respectively). Correlations were also observed between HR status and SBR grade, mean MRLD, and pCR with *P* value < 0.05. Other parameters, including mean age, race, lesion type, HER2, and arm, were independent of HR.

**Table 1:** Patients characteristics in discovery and validation cohort

Variable	Discovery cohort		Validation cohort		*P* value
Total, *N*	117		74		
*Age*					
Mean (range)	49.03 (25–77)		48.58 (27–72)		0.769
*Race*					0.414
White	87	74.35%	53	71.62%	
Asian	10	8.55%	3	4.05%	
Black	9	7.69%	9	12.16%	
Unknown	11	9.41%	8	10.81%	
Native Hawaiian or other Pacific Islander	0	0	1	1.35%	
*Lesion type*					0.309
Multiple masses	65	55.56%	33	44.59%	
Multiple NME	5	4.27%	6	8.11%	
Single mass	43	36.75%	30	40.54%	
Single NME	4	3.42%	5	6.76%	
*HER2*					0.974
Positive	30	25.64%	18	24.32%	
Negative	87	74.36%	56	75.68%	
*HR*					0.959
Positive	71	60.68%	46	62.16%	
Negative	46	39.32%	28	37.84%	
*SBR grade*					0.592
I (Low)	3	2.56%	2	2.70%	
II (Intermediate)	36	30.77%	17	22.97%	
III (High)	77	65.81%	55	74.32%	
Unknown	1	0.85%	0	0	
*MRLD*					
Mean (range)	4.02 (0.44–15)		4.73 (1.6–13.2)		0.049
*pCR*					1.000
pCR	36	30.77%	23	31.08%	
non-pCR	81	69.23%	51	68.92%	
Treatment arm*					0.351
Paclitaxel	23	19.66%	15	20.27%	
Paclitaxel + Trastuzumab	3	2.56%	1	1.35%	
Paclitaxel + MK-2206	11	9.40%	6	8.11%	
Paclitaxel + Trastuzumab + MK-2206	9	7.69%	2	2.70%	
Paclitaxel + Trebananib	27	23.08%	12	16.22%	
Paclitaxel + Trastuzumab + Trebananib	2	1.71%	4	5.41%	
Trastuzumab + Pertuzumab	10	8.55%	4	5.41%	
Paclitaxel + Trastuzumab + Pertuzumab	6	5.13%	4	5.41%	
Paclitaxel + Ganitumab	24	20.51%	20	27.03%	
Paclitaxel + Ganetespib	2	1.71%	3	4.05%	
Paclitaxel + Neratinib	0	0	2	2.70%	
Unknown	0	0	1	1.35%	

NME, non-mass-like enhancement; HR, hormone receptor; HER2, human epidermal growth factor receptor 2; SBR, grade Scarff-Bloom-Richardson grade; MRLD, MRI measured longest diameter (cm) at baseline (T0) study; pCR, pathologic complete response; MK-2206, AKT inhibitor

*All treatment arms are followed by 4 cycles of doxorubicin/cyclophosphamide

### Image feature repeatability and reproducibility

The averaged feature repeatability ICC against image was 0.73 (standard deviation: 0.20), and 226/1316 (17.2%) image features showed excellent repeatability (ICC > 0.9). The averaged feature reproducibility ICC against test–retest imaging is 0.55 (standard deviation: 0.22), and 808/1316 (61.4%) image features showed good to excellent repeatability (ICC > 0.5). Distributions of the feature repeatability and reproducibility ICCs are visualized in Additional file 1: Figure S2, and the ICC values with 95% CI are tabulated in Additional file 1: Table S11. After removing low repeatable (ICC < 0.9) and reproducible (ICC < 0.5) image features, 219 remained for further feature selection and image signature (IS_HER2_ and IS_HR_) establishment.

### Image signatures and association to IHC status

**Fig. 2:**
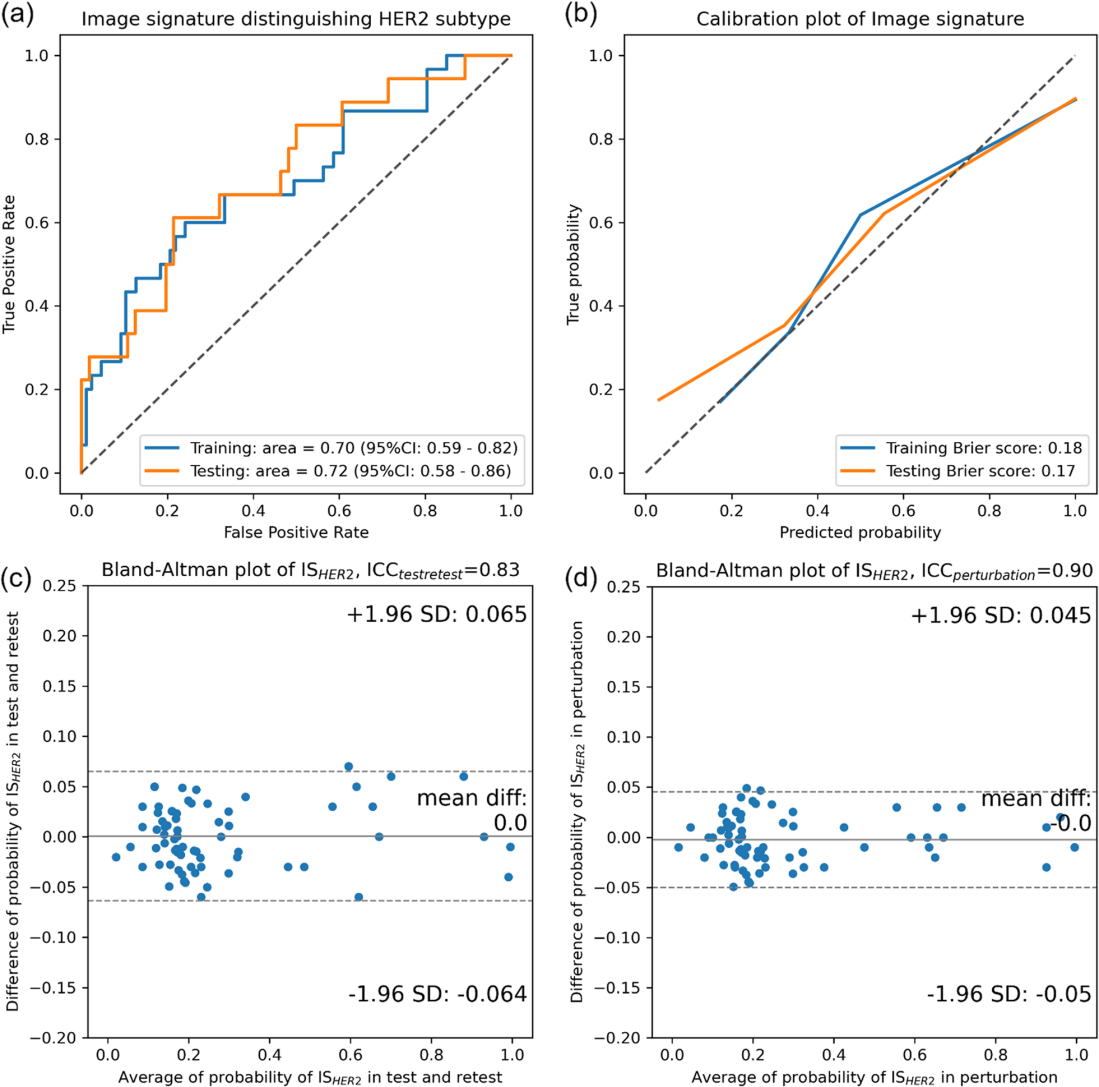
Performance of image signature, IS_HER2_, in **a** the ROC analysis in both discovery and validation cohorts, **b** the calibration of IS_HER2_ between true probabilities and predicted probability with brier score, **c**, **d** the Bland–Altman plot of IS_HER2_ in test–retest cohort and perturbation cohorts

Five image features were finally selected to construct HER2-associated image signature IS_HER2_ through RIDGE logistic regression. The coefficients, repeatability ICC, and reproducibility ICC of the selected image features are tabulated in Additional file 1: Table S5. Figure 2 visualizes the classification, calibration, and robustness performances of the signature in the discovery and validation cohort. As shown by the ROC curves in Fig. 2a, the AUC for classifying HER2 + from HER2- was 0.70 (95% CI 0.59 to 0.82) in the discovery cohort and 0.72 (95% CI 0.58 to 0.86) in the validation cohort. The calibration curves are also drawn in Fig. 2b with Brier score of 0.18/0.17 (discovery/validation). Additional file 1: Figure S5 shows the example cases of consistent and inconsistent IS_HER2_ and HER2. During the signature robustness evaluation, the average reproducibility ICC was 0.83 (95% CI 0.77 to 0.89) against test–retest and the repeatability ICC was 0.90 (95% CI 0.86 to 0.93) against image perturbations. Bland–Altman plot visualizing the probability differences versus the average probability in the test–retest scans is drawn in Fig. 2c and perturbation images in Fig. 2d. Both test–retest and perturbation evaluations resulted in an average probability difference of 0 and standard deviation of 0.06 and 0.05, respectively.

**Fig. 3:**
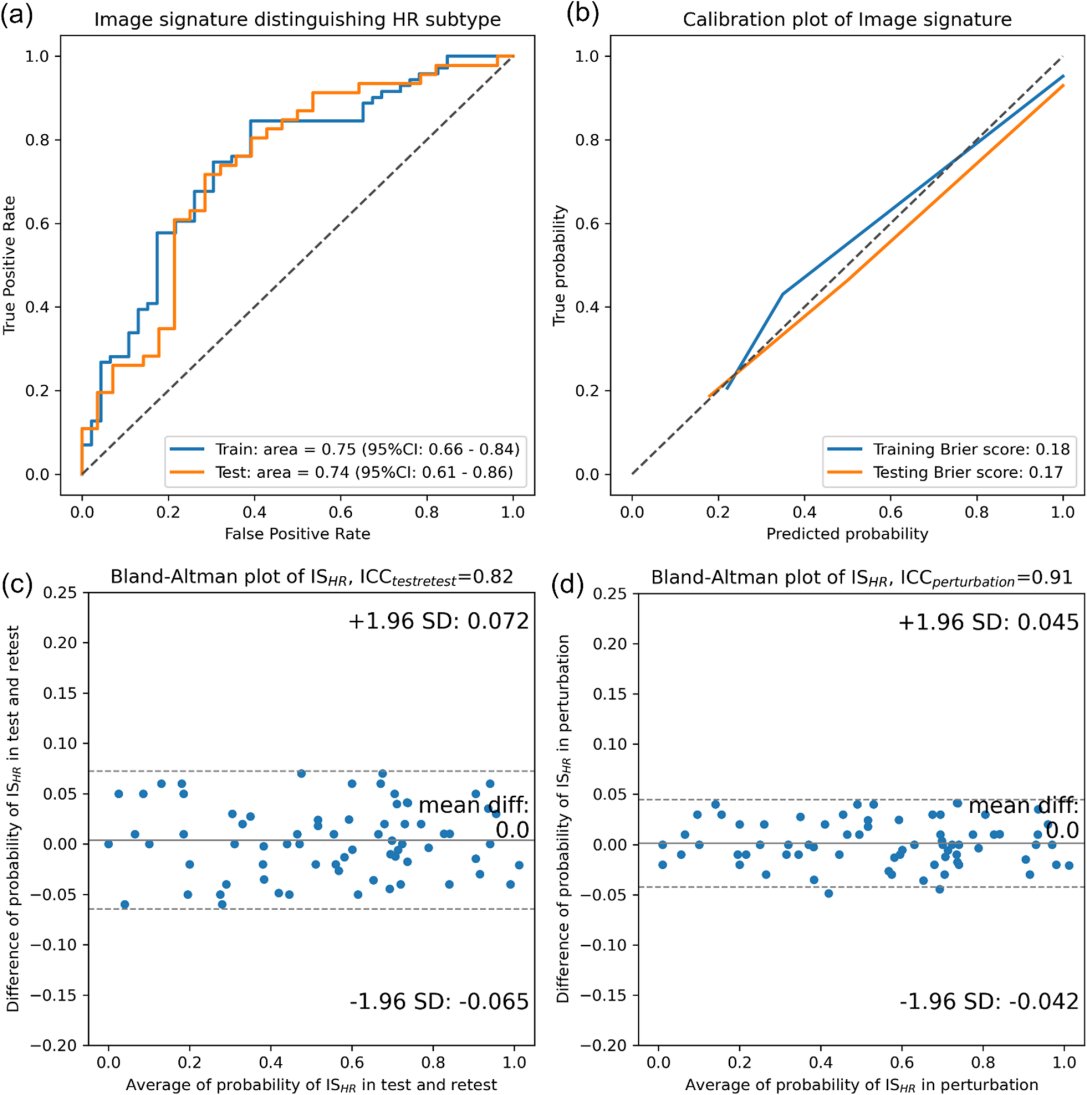
Performance of image signature, IS_HR_, in **a** the ROC analysis in both discovery and validation cohorts, **b** the calibration of IS_HR_ between true probabilities and predicted probability with brier score, **c**, **d** the Bland–Altman plot of IS_HR_ in test–retest cohort and perturbation cohorts

HR-associated image signature IS_HR_ was constructed by five image features after feature selection. The coefficients, repeatability ICC, and reproducibility ICC of the selected image features are tabulated in Additional file 1: Table S6. Compared to IS_HER2,_ higher AUCs for classifying HR + from HR- were achieved with 0.75 (95% CI 0.66 to 0.84) in discovery and 0.74 (95% CI 0.61 to 0.86) in validation (Fig. 3a). Figure 3b shows the calibration curves with Brier score of 0.18/0.17, which were consistent with IS_HER2_. Example images of consistent and inconsistent IS_HR_ and IHC HR are shown in Additional file 1: Figure S5. During the signature robustness evaluation, the average reproducibility ICC was 0.82 (95% CI 0.78 to 0.86) against test–retest and the repeatability ICC was 0.91 (95% CI 0.88 to 0.94) against image perturbations. Both test–retest and perturbation evaluations resulted in an average probability difference of 0 and standard deviation of 0.07 and 0.04, respectively.

### pCR prediction performance

**Fig. 4:**
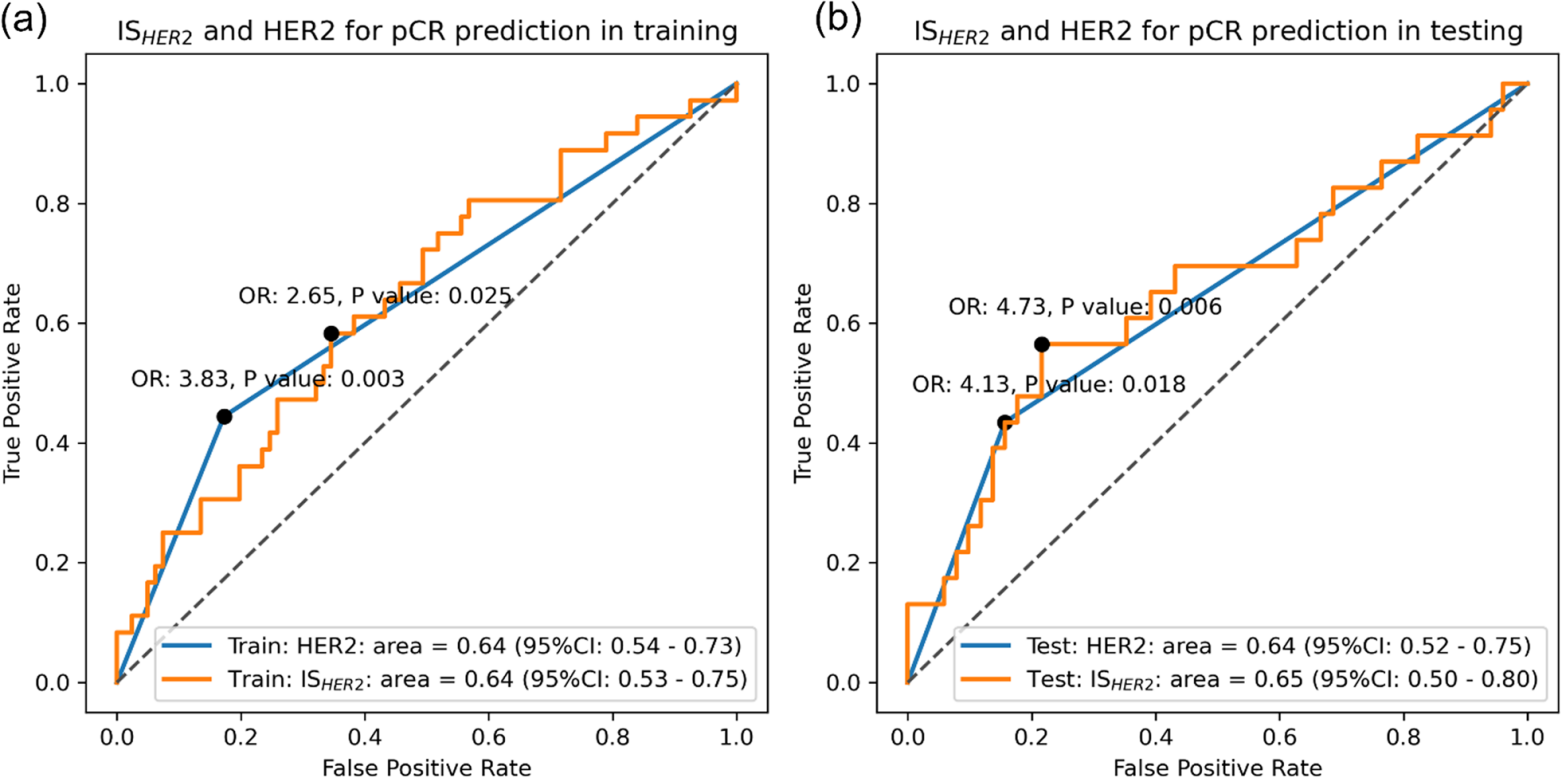
The ROC analysis evaluating the association between IS_HER2_ and pCR and IS_HER2_ and HER2 both showed significant association to pCR. The odds ratio between dichotomized IS_HER2_ was also calculated, and a significant association to pCR was also observed

The associations between image signatures and pCR were evaluated for clinical utility assessment. The ROCs of IS_HER2_ in classifying pCR and non-pCR are shown in Fig. 4 where an AUC of 0.64 (95% CI 0.53 to 0.75) and 0.65 (95% CI 0.50 to 0.80) was reported in the discovery and validation cohort. Similarly, the baseline prognostic ability of molecular subtype HER2 in predicting the pCR showed an AUC of 0.64 (95% CI 0.54 to 0.73)/0.64 (95% CI 0.52 to 0.75). No statistical significance difference in ROC was observed between IS_HER2_ and IHC HER2 in both the discovery and validation cohort (*P* value > 0.05). Furthermore, there were more pCR cases in the IS_HER2_-positive group (cutoff value of 0.25 in the discovery cohort and 0.26 in the validation cohort) with OR of 2.65 (95% CI 1.18 to 5.93, *P* value = 0.025) / 4.73 (95% CI 1.64 to 13.65, *P* value = 0.006). The dichotomized IS_HER2_ and IHC receptor HER2 did not show a statistically significant difference in pCR prediction after neoadjuvant chemotherapy.

**Fig. 5:**
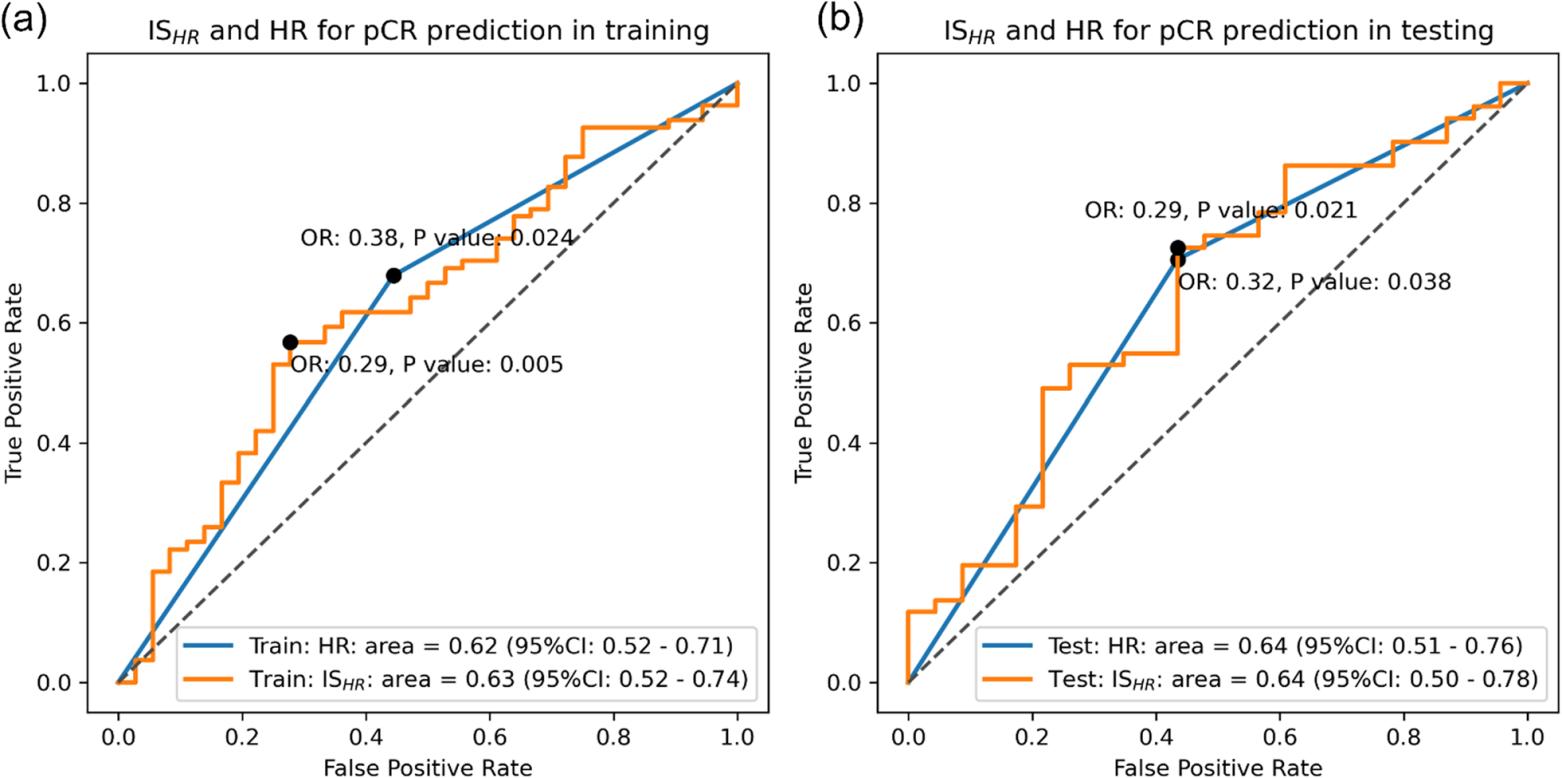
The ROC analysis evaluating the association between IS_HR_ and pCR and IS_HR_ and HR both showed significant association to pCR. The odds ratio between dichotomized IS_HR_ was also calculated, and a significant association to pCR was also observed

Similar performances in predicting pCR were observed in IS_HR_. As shown in Fig. 5, IS_HR_ reached an AUC of 0.63 (95% CI 0.52 to 0.74) in the discovery cohort and AUC of 0.64 (95% CI 0.50 to 0.78) in the validation cohort. The baseline prognostic ability of molecular subtype HR in predicting the pCR showed similar AUCs of 0.62 (95% CI 0.52 to 0.71) / 0.64 (95% CI 0.51 to 0.76). It is worth noting that both IS_HR_ and HR were negatively associated with pCR (i.e., patients with HR-positive status are less likely to achieve pCR), and we purposely reversed the ROC curve for a consistent visualization without affecting the analysis results. No statistically significant difference between ROC curves of IS_HR_ and IHC receptor HR on predicting pCR was found in both discovery and validation cohort (*P* value > 0.05). A higher pCR rate was observed In the IS_HR_-negative group (cutoff value = 0.61/0.53) with OR of 0.29 (95% CI 0.12 to 0.69, *P* value = 0.005)/0.29 (95% CI 0.10 to 0.81, *P* value = 0.021). Similar to IS_HER2,_ the binarized IS_HR_ and receptor subtype HR also did not show a statistically significant difference in pCR prediction after neoadjuvant chemotherapy (*P* value > 0.05).

### Image signature subtypes and subgroup analysis

Moderate associations between image signatures subtypes and IHC molecular subtypes were also observed in the discovery cohort and validation cohort. The accuracies were 64%, 68%, 75%, and 73% for HER2-/HR+, HER2-/HR-, HER2+/HR+, and HER2+/HR-, respectively, in the discovery cohort and 59%, 73%, 82% and 88% in the validation cohort. In addition to high pCR prediction performance of the two image signatures, significant pCR rate differences were observed between the following subtype groups: IS_HER2-HR+_ vs. non-IS_HER2-HR+_ (OR: 0.15, *P* value = 0.002), IS_HER2+HR-_ vs. non-IS_HER2+HR-_ (OR: 3.66, *P* value = 0.007) in the discovery cohort, and IS_HER2-HR+_ vs. non-IS_HER2-HR+_ (OR: 0.12, *P* value = 0.001), IS_HER2+HR-_ versus non-IS_HER2+HR-_ (OR: 8.65, *P* value = 0.009) in the validation cohort, as shown in Table 2.

**Table 2:** The association between pCR and IHC molecular subtype as well as image molecular subtypes was quantified with odds ratio

	Discovery				Validation			
	Image signature		IHC		Image signature		IHC	
	OR	*P* value	OR	*P* value	OR	*P* value	OR	*P* value
HER2 + versus HER2-	2.65	**0.025**	3.83	**0.003**	4.73	**0.006**	4.13	**0.018**
HR + versus HR-	0.29	**0.005**	0.38	**0.024**	0.29	**0.021**	0.32	**0.038**
HER2-HR + versus non-HER2-HR +	0.15	**0.002**	0.25	**0.002**	0.12	**0.001**	0.23	**0.011**
HER2-HR- versus non-HER2-HR-	1.26	0.663	1.19	0.828	1.42	0.572	1.28	0.787
HER2 + HR + versus non-HER2 + HR +	0.98	1.000	1.64	0.425	2.04	0.236	1.49	0.526
HER2 + HR- versus non-HER2 + HR-	3.66	**0.007**	11.29	**0.001**	8.65	**0.009**	28.33	**0.002**

The significant *P* value was bold

The image signatures also demonstrated independent pCR prediction values to IHC receptor subgroups. As shown in Additional file 1: Tables S7 and S8, the image signature for HER2 showed significant associations to pCR in HR + (AUC = 0.70/0.77, *P* value = 0.013/0.010) subgroup, and the signature for HR showed significant associations in HER2- (AUC = 0.74/0.73, *P* value = 0.001/0.013) and HER2-/HR + (AUC = 0.72/0.79, *P* value = 0.046/0.045) subgroups. The multivariate analysis, as reported in Additional file 1: Table S9, also confirmed the independent predictive value of both IS_HER2_ and IS_HR_ with *P* values of 0.001 and 0.008. The multivariate model combining IHC HR and HER2 and the corresponding image signatures achieved the highest AUC of 0.78 (95% CI 0.71–0.84), while the IHC receptors alone had an AUC of 0.71 (95% CI 0.63–0.79) (Additional file 1: Table S10).

## Discussion

HER2 and HR characterize the expression of human epidermal growth factor receptor 2 and hormone receptor based on IHC staining of surgical specimens and are widely used biomarkers for invasive breast cancer for treatment selection and response prediction [3]. In this study, we successfully developed two noninvasive imaging signatures, IS_HER2_ and IS_HR_, by radiomic analysis of pre-treatment ADC maps. Both demonstrated high repeatability and reproducibility under image perturbation and test–retest scans. They were validated to have strong associations with HER2 and HR in both the discovery and unseen validation cohort. We also independently confirmed the prediction value of the image signatures for neoadjuvant chemotherapy treatment response. Moreover, both signatures demonstrated independent prediction values to the IHC receptors. Our results partially demonstrated the potential of the proposed image signatures as noninvasive alternatives to HER2 and HR. Their unique advantages in noninvasiveness and accessibility enable continuous disease monitoring throughout the treatment course and timely disease intervention. Meanwhile, the sophisticated characterizations of ADC maps may capture a more accurate and complete representation of the tumor’s condition than a single-site biopsy for IHC test.

Despite the rather complex acquisitions of the proposed ADC signatures, they achieved superior performance than the single-valued ADC in both classifications of IHC receptor status and predictions of treatment response. As explained in the Introduction section, inconsistent results of the correlations of ADC values with receptor status were reported in previous studies [7]. For instance, Martincich et al. (2012) found a weak but significant correlation with ER but insufficient significance with the HER2 status [34]. Similar observations were reported by Horvat et al. where maximum whole tumor ADC values were significantly associated with ER and PR with AUC of 0.72–0.73 and 0.66–0.67, respectively, but less significant for HER2 [35]. Conversely, Park et al. discovered significant associations between mean ADC values and HER2 status in invasive ductal carcinoma patients [36]. In contrast, our proposed image signatures can quantify the patterns of ADC maps within the tumor volume, which are much more sophisticated than simple statistics such as mean or median, resulting in better classification performances with AUC of 0.74–0.75 for HR and 0.70–0.72 for HER2. The primary analysis of ACRIN6698 trial discovered that mid-treatment percent change in tumor ADC was significantly predictive to pCR with overall AUC = 0.60 (95% CI 0.52–0.68), and a higher performance was achieved with AUC = 0.72 (95% CI 0.61–0.83) when incorporating breast cancer subtype as covariate [19]. Our images signatures, developed from baseline ADC map only, demonstrated stronger pCR associations with AUC = 0.64–0.65 alone and AUC = 0.75–0.78 when combined with IHC test results. Nevertheless, we do acknowledge the complex calculations of the images signatures and have provided the source code to automate the computation and reduce the learning curve in clinical application.

Several considerations were made when formulating the study methodology for clinical utility. First, the ADC map was chosen for image signature development for optimal reproducibility due to its quantitative nature. Although other images modalities such as DCE-MR have also been demonstrated to be valuable in predicting IHC receptor status and pCR [37–39], they are more susceptible to image acquisition settings and less accessible due to contrast agent administration. Second, we attempted to develop image signatures based on IHC receptor status during diagnosis instead of directly targeting the prognostic endpoint pCR. Although a higher pCR prediction performance is more likely to be achieved in the study cohort if using the latter approach [40], it may impose a higher risk of overfitting, as pCR can be affected by a lot more factors than the tumor appearance. We believe that it is more practical to discover the relationships of image phenotypes with tumor biological condition, and the resulting image signatures are more explainable with more robust and generalizable prognostic performance. Compared to existing studies that aimed to construct image signatures by fitting them with the molecular signatures [25], we further validated the clinical values in pCR association, which is often missing in their analysis.

Another important strength of our study is that we explicitly designed and validated the image signatures with high repeatability and reproducibility. Despite the well-recognized concerns on repeatability and reproducibility in radiomic analysis, only a handful of studies attempted to extensively evaluate the reliability of constructed image signatures [41]. The lack of reliability assessment is likely to result in the poor generalizability [16] of the signatures and eventually hinders any potential clinical utility [15]. In this study, we attempted to overcome the potential instability of radiomics and the ADC maps [42] by extensively incorporate the reliability assessment in both signature construction and evaluation using image perturbation and test–retest.

Several limitations were notified in the study. Firstly, the sample size is limited despite countermeasures such as reliability assessment and independent validation on its association with IHC receptor status as well as pCR to neoadjuvant chemotherapy. Secondly, the heterogeneity of the treatment arms prevents specific drug recommendations for patients [43]. In addition to neoadjuvant chemotherapy response prediction, one of the most important roles of HER2 and HR from IHC is guiding targeted therapy [3], which could not be directly validated on the proposed image signatures due to the retrospective nature of this study. Finally, we did not include other image modalities such as DCE-MR for signature development. However, we plan to explore the use of both ADC maps and DCE images, or other imaging modalities, to further improve the predictive performance in future studies.

## Conclusions

In conclusion, we developed two reliable radiomic signatures IS_HER2_ and IS_HR_ that had significant associations with HER2 and HR status. They shared similar performance in treatment response prediction to neoadjuvant chemotherapy. Further investigations on their ability in treatment guidance are warranted in order to fully validate their potentials as noninvasive surrogates to IHC tests.

## Supplementary Information

**Additional file 1**. Supplementary material.

## Data Availability

The dataset that supports the findings of this study is a public dataset available at ACRIN 6698/I-SPY2 Breast DWI (ACRIN 6698) - The Cancer Imaging Archive (TCIA) Public Access - Cancer Imaging Archive Wiki.
