# Supplementary material for: Noninvasive imaging signatures of HER2 and HR using ADC in invasive breast cancer: repeatability, reproducibility, and association with pathological complete response to neoadjuvant chemotherapy
Source: Breast Cancer Res. 2023 Jun 28;25:77. doi: 10.1186/s13058-023-01674-9 (PMC10308682; doi:10.1186/s13058-023-01674-9)
Supplement: Supplementary file 1 — Additional file 1. Supplementary material. [file 13058_2023_1674_MOESM1_ESM.docx]

# Imaging data and tumor segmentation

MRI was performed by using a 1.5- or 3.0-T field scanner with a breast radiofrequency coil. DW MRI was performed before DCE MRI by using a DW single-shot echo planar imaging sequence with parallel imaging (reduction factor, two or greater); fat suppression; a repetition time of greater than 4,000 msec; echo time minimum; flip angle, 90°; field of view, 300–360 mm; acquired matrix, 128 x 128 to 192 x 192; in-plane resolution, 1.7–2.8 mm; section thickness, 4–5 mm; and imaging time, 5 or fewer minutes. Diffusion gradients were applied in three orthogonal directions by using diffusion weightings (b values) of 0, 100, 600, and 800 sec/mm^2^. No respiratory triggering or other motion compensation methods were used. The study protocol did not specify the gadolinium agent to be used, but required that the same agent be used for all MRI examinations for an individual patient.

Reference:

ACRIN 6698 trial protocol and imaging materials. https://www.acrin.org/PROTOCOLSUMMARYTABLE/PROTOCOL6698/6698ImagingMaterials.aspx. Accessed July 9, 2018

# Imaging feature extraction parameters

Table S1 The parameters for perturbation and feature extraction.

| Pixel value offset | 0 |
| --- | --- |
| Resample pixel size (mm) | [1,1,1] |
| Image/mask interpolation algorithm | B-spline |
| Mask partial volume threshold | 0.5 |
| Interpolation grid alignment | Align grid origins |
| Translation distances (pixel) | [ 0.0, 0.2, 0.4, 0.6, 0.8] |
| Rotation angles (degree) | [-5,0,5] |
| Rotation axis | Mask bounding box center, axial direction |
| Contour randomization smoothing sigma (mm) | [10,10,10] |
| Contour randomization intensity (mm) | [1,1,1] |
| Perturbation times | 40 |
| Image discretization bin number | 32 |
| Image filters | Unfiltered, Laplacian-of-Gaussian (3D), Wavelet |
| Kernel size of Laplacian-of-Gaussian filter (mm) | [1,2,3,4,5] |
| Wavelet filter starting level | 0 |
| Wavelet filter total level | 1 |
| Wavelet filter type | Coilf1 |
| Wavelet filter decompositions | [LLL, HLL, LHL, LLH, LHH, HLH, HHL, HHH] |
| Feature class | shape, firstorder, glcm, glrlm, glszm, gldm, ngtdm |

# Imaging feature definition

All the image feature calculated are compliant with the Image Biomarker Standardization Initiative (IBSI) using the PyRadiomics package.

Table S2 Image features extracted for feature selection and signature building.

|  | **Gray level cooccurrence matrix（GLCM）** |  | **Gray level difference matrix（GLDM）** |
| --- | --- | --- | --- |
| **Feature Name** | Autocorrelation |  | DependenceEntropy |
|  | ClusterProminence |  | DependenceNonUniformity |
|  | ClusterShade |  | DependenceNonUniformityNormalized |
|  | ClusterTendency |  | DependenceVariance |
|  | Contrast |  | GrayLevelNonUniformity |
|  | Correlation |  | GrayLevelVariance |
|  | DifferenceAverage |  | HighGrayLevelEmphasis |
|  | DifferenceEntropy |  | LargeDependenceEmphasis |
|  | DifferenceVariance |  | LargeDependenceHighGrayLevelEmphasis |
|  | Id |  | LargeDependenceLowGrayLevelEmphasis |
|  | Idm |  | LowGrayLevelEmphasis |
|  | Idmn |  | SmallDependenceEmphasis |
|  | Idn |  | SmallDependenceHighGrayLevelEmphasis |
|  | Imc1 |  | SmallDependenceLowGrayLevelEmphasis |
|  | Imc2 |  |  |
|  | InverseVariance |  |  |
|  | JointAverage |  |  |
|  | JointEnergy |  |  |
|  | JointEntropy |  |  |
|  | MCC |  |  |
|  | MaximumProbability |  |  |
|  | SumAverage |  |  |
|  | SumEntropy |  |  |
|  | SumSquares |  |  |
|  | **Gray level run length matrix（GLRLM）** |  | **Gray level size zone matrix（GLSZM）** |
| **Feature Name** | GrayLevelNonUniformity |  | GrayLevelNonUniformity |
|  | GrayLevelNonUniformityNormalized |  | GrayLevelNonUniformityNormalized |
|  | GrayLevelVariance |  | GrayLevelVariance |
|  | HighGrayLevelRunEmphasis |  | HighGrayLevelZoneEmphasis |
|  | LongRunEmphasis |  | LargeAreaEmphasis |
|  | LongRunHighGrayLevelEmphasis |  | LargeAreaHighGrayLevelEmphasis |
|  | LongRunLowGrayLevelEmphasis |  | LargeAreaLowGrayLevelEmphasis |
|  | LowGrayLevelRunEmphasis |  | LowGrayLevelZoneEmphasis |
|  | RunEntropy |  | SizeZoneNonUniformity |
|  | RunLengthNonUniformity |  | SizeZoneNonUniformityNormalized |
|  | RunLengthNonUniformityNormalized |  | SmallAreaEmphasis |
|  | RunPercentage |  | SmallAreaHighGrayLevelEmphasis |
|  | RunVariance |  | SmallAreaLowGrayLevelEmphasis |
|  | ShortRunEmphasis |  | ZoneEntropy |
|  | ShortRunHighGrayLevelEmphasis |  | ZonePercentage |
|  | ShortRunLowGrayLevelEmphasis |  | ZoneVariance |

# Patient cohorts

The patient cohorts were splits into discovery, validation and test-retest. The discovery is used for signature development. All three cohorts were used to evaluate the developed image signature.


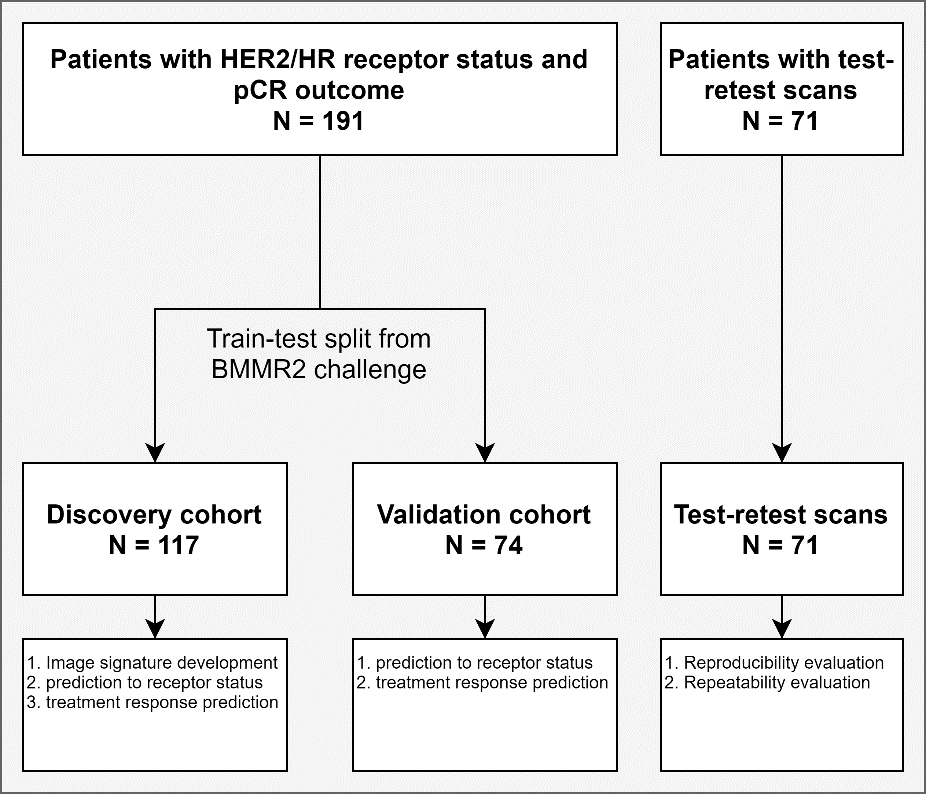


Figure S1 The patients split into discovery (N=117) and validation (N=74) cohorts. Test-retest scans (N=71) are used for evaluation of repeatability and reproducibility.

Table S3 Patient characteristics comparison between HER2 positive and HER2 negative in discovery and validation cohorts.

|  |  | **Discovery cohort** | | | | | **Validation cohort** | | | | |  |
| --- | --- | --- | --- | --- | --- | --- | --- | --- | --- | --- | --- | --- |
|  |  | **HER2 Positive** | | **HER2 Negative** | | **P value** | **HER2 Positive** | | **HER2 Negative** | | **P value** |  |
| **Total,N** | | 30 | | 87 | |  | 18 | | 56 | |  |  |
| **Mean age(range)** | | 50.97(29-71) |  | 48.36(25-77) |  | 0.2819 | 48.94(27-72) |  | 48.46(31-66) |  | 0.8796 |  |
| **Race** | |  |  |  |  | 0.1967 |  |  |  |  | 0.6340 |  |
|  | White | 24 | 80.00% | 63 | 72.41% |  | 14 | 77.78% | 39 | 69.64% |  |  |
|  | Asian | 4 | 13.33% | 6 | 6.90% |  | 0 | 0.00% | 3 | 5.36% |  |  |
|  | Black | 0 | 0.00% | 9 | 10.34% |  | 1 | 5.56% | 8 | 14.29% |  |  |
|  | Unknown | 2 | 6.67% | 9 | 10.34% |  | 3 | 16.67% | 5 | 8.93% |  |  |
|  | Native Hawaiian or other Nacific Islander | 0 | 0.00% | 0 | 0.00% |  | 0 | 0.00% | 1 | 1.79% |  |  |
| **Lesion type** | |  |  |  |  | 0.6733 |  |  |  |  | 0.7842 |  |
|  | Multiple masses | 17 | 56.67% | 48 | 55.17% |  | 7 | 38.89% | 26 | 46.43% |  |  |
|  | Multiple NME | 1 | 3.33% | 4 | 4.60% |  | 1 | 5.56% | 5 | 8.93% |  |  |
|  | Single mass | 10 | 33.33% | 33 | 37.93% |  | 8 | 44.44% | 22 | 39.29% |  |  |
|  | Single NME | 2 | 6.67% | 2 | 2.30% |  | 2 | 11.11% | 3 | 5.36% |  |  |
| **HR** | |  |  |  |  | 0.5746 |  |  |  |  | 0.4640 |  |
|  | Positive | 20 | 66.67% | 51 | 58.62% |  | 13 | 72.22% | 33 | 58.93% |  |  |
|  | Negative | 10 | 33.33% | 36 | 41.38% |  | 5 | 27.78% | 23 | 41.07% |  |  |
| **SBRgrade** | |  |  |  |  | 0.5664 |  |  |  |  | 0.8574 |  |
|  | I (Low) | 0 | 0.00% | 3 | 3.45% |  | 0 | 0.00% | 2 | 3.57% |  |  |
|  | II (Intermediate) | 12 | 40.00% | 24 | 27.59% |  | 5 | 27.78% | 12 | 21.43% |  |  |
|  | III (High) | 18 | 60.00% | 59 | 67.82% |  | 13 | 72.22% | 42 | 75.00% |  |  |
|  | Unknown | 0 | 0.00% | 1 | 1.15% |  | 0 | 0.00% | 0 | 0.00% |  |  |
| **Mean MRLD** | | 3.80(0.44-12) |  | 4.09(0.8-15) |  | 0.5635 | 4.64(2.4-9.1) |  | 4.75(1.6-13.2) |  | 0.8611 |  |
| **pCR** | |  |  |  |  | 0.0040 |  |  |  |  | 0.0224 |  |
|  | pCR | 16 | 53.33% | 20 | 22.99% |  | 10 | 55.56% | 13 | 23.21% |  |  |
|  | non-pCR | 14 | 46.67% | 67 | 77.01% |  | 8 | 44.44% | 43 | 76.79% |  |  |
| **Arm** | |  |  |  |  | 2.2E-16 |  |  |  |  | 6.47E-16 |  |
|  | AC-T | 0 | 0.00% | 23 | 26.44% |  | 0 | 0.00% | 15 | 26.79% |  |  |
|  | AC-T + MK-2206 | 0 | 0.00% | 11 | 12.64% |  | 0 | 0.00% | 6 | 10.71% |  |  |
|  | AC-T + MK-2206 + T-DM1 | 9 | 30.00% | 0 | 0.00% |  | 2 | 11.11% | 0 | 0.00% |  |  |
|  | AC-T + AMG 386 | 0 | 0.00% | 27 | 31.03% |  |  | 0.00% | 12 | 21.43% |  |  |
|  | AC-T + T-DM1 | 3 | 10.00% | 0 | 0.00% |  | 1 | 5.56% | 0 | 0.00% |  |  |
|  | AC-T + AMG 386 + T-DM1 | 2 | 6.67% | 0 | 0.00% |  | 4 | 22.22% | 0 | 0.00% |  |  |
|  | T-DM1 + Pertuzumab | 10 | 33.33% |  | 0.00% |  | 4 | 22.22% | 0 | 0.00% |  |  |
|  | AC-T + Pertuzumab + T-DM1 | 6 | 20.00% | 0 | 0.00% |  | 4 | 22.22% | 0 | 0.00% |  |  |
|  | AC-T + AMG 479 | 0 | 0.00% | 24 | 27.59% |  | 0 | 0.00% | 20 | 35.71% |  |  |
|  | AC-T + Ganetespib | 0 | 0.00% | 2 | 2.30% |  | 0 | 0.00% | 3 | 5.36% |  |  |
|  | AC-T + HKI-272 | 0 | 0.00% | 0 | 0.00% |  | 2 | 11.11% | 0 | 0.00% |  |  |
|  | Unknown | 0 | 0.00% | 0 | 0.00% |  | 1 | 5.56% | 0 | 0.00% |  |  |
| Abbreviations: NME non-mass-like enhancement, HR hormone receptor, HER2 human epidermal growth factor receptor 2, SBR grade Scarff-Bloom-Richardson grade, MRLD MRI measured longest diameter (cm) at baseline (T0) study, pCR pathologic complete response, AC-T Paclitaxel, AMG 479 Ganitumab, T-DM1 Trastuzumab, HKI-272 Neratinib | | | | | | | | | | | |  |
|  |  |  |  |  |  |  |  |  |  |  |  |  |

Table S4 Patient characteristics comparison between HR positive and HR negative in discovery and validation cohorts.

|  |  | **Discovery cohort** | | | | | **Validation cohort** | | | | |  |
| --- | --- | --- | --- | --- | --- | --- | --- | --- | --- | --- | --- | --- |
|  |  | **HR Positive** | | **HR Negative** | |  | **HR Positive** | | **HR Negative** | |  |  |
| **Total,N** | | 71 | | 46 | |  | 46 | | 28 | |  |  |
| **Mean age(range)** | | 49.46(29-71) |  | 48.35(25-77) |  | 0.6121 | 48.15(27-72) |  | 49.29(31-65) |  | 0.6136 |  |
| **Race** | |  |  |  |  | 0.9494 |  |  |  |  | 0.8878 |  |
|  | White | 54 | 76.06% | 33 | 71.74% |  | 33 | 71.74% | 20 | 71.43% |  |  |
|  | Asian | 6 | 8.45% | 4 | 8.70% |  | 1 | 2.17% | 2 | 7.14% |  |  |
|  | Black | 5 | 7.04% | 4 | 8.70% |  | 6 | 13.04% | 3 | 10.71% |  |  |
|  | Unknown | 6 | 8.45% | 5 | 10.87% |  | 5 | 10.87% | 3 | 10.71% |  |  |
|  | Native Hawaiian or other Nacific Islander | 0 | 0.00% | 0 | 0.00% |  | 1 | 2.17% | 0 | 0.00% |  |  |
| **Lesion type** | |  |  |  |  | 0.9051 |  |  |  |  | 0.3794 |  |
|  | Multiple masses | 41 | 57.75% | 24 | 52.17% |  | 23 | 50.00% | 10 | 35.71% |  |  |
|  | Multiple NME | 3 | 4.23% | 2 | 4.35% |  | 4 | 8.70% | 2 | 7.14% |  |  |
|  | Single mass | 25 | 35.21% | 18 | 39.13% |  | 15 | 32.61% | 15 | 53.57% |  |  |
|  | Single NME | 2 | 2.82% | 2 | 4.35% |  | 4 | 8.70% | 1 | 3.57% |  |  |
| **HER2** | |  |  |  |  | 0.5746 |  |  |  |  | 0.4640 |  |
|  | Positive | 20 | 28.17% | 10 | 21.74% |  | 13 | 28.26% | 5 | 17.86% |  |  |
|  | Negative | 51 | 71.83% | 36 | 78.26% |  | 33 | 71.74% | 23 | 82.14% |  |  |
| **SBRgrade** | |  |  |  |  | 0.0077 |  |  |  |  | 0.0490 |  |
|  | I (Low) | 3 | 4.23% | 0 | 0.00% |  | 2 | 4.35% | 0 | 0.00% |  |  |
|  | II (Intermediate) | 28 | 39.44% | 8 | 17.39% |  | 14 | 30.43% | 3 | 10.71% |  |  |
|  | III (High) | 39 | 54.93% | 38 | 82.61% |  | 30 | 65.22% | 25 | 89.29% |  |  |
|  | Unknown | 1 | 1.41% | 0 | 0.00% |  | 0 | 0.00% | 0 | 0.00% |  |  |
| **Mean MRLD** | | 3.71(0.44-10) |  | 4.50(1.9-15) |  | 0.0858 | 4.81(1.6-13.2) |  | 4.59(1.7-11.4) |  | 0.7058 |  |
| **pCR** | |  |  |  |  | 0.0284 |  |  |  |  | 0.0492 |  |
|  | pCR | 16 | 22.54% | 20 | 43.48% |  | 10 | 21.74% | 13 | 46.43% |  |  |
|  | non-pCR | 55 | 77.46% | 26 | 56.52% |  | 36 | 78.26% | 15 | 53.57% |  |  |
| **Arm** | |  |  |  |  | 0.5694 |  |  |  |  | 2.22E-01 |  |
|  | AC-T | 15 | 21.13% | 8 | 17.39% |  | 9 | 19.57% | 6 | 21.43% |  |  |
|  | AC-T + MK-2206 | 7 | 9.86% | 4 | 8.70% |  | 3 | 6.52% | 3 | 10.71% |  |  |
|  | AC-T + MK-2206 + T-DM1 | 7 | 9.86% | 2 | 4.35% |  | 0 | 0.00% | 2 | 7.14% |  |  |
|  | AC-T + AMG 386 | 12 | 16.90% | 15 | 32.61% |  | 9 | 19.57% | 3 | 10.71% |  |  |
|  | AC-T + T-DM1 | 1 | 1.41% | 2 | 4.35% |  | 1 | 2.17% | 0 | 0.00% |  |  |
|  | AC-T + AMG 386 + T-DM1 | 2 | 2.82% | 0 | 0.00% |  | 4 | 8.70% | 0 | 0.00% |  |  |
|  | T-DM1 + Pertuzumab | 7 | 9.86% | 3 | 6.52% |  | 2 | 4.35% | 2 | 7.14% |  |  |
|  | AC-T + Pertuzumab + T-DM1 | 3 | 4.23% | 3 | 6.52% |  | 10 | 21.74% | 0 | 0.00% |  |  |
|  | AC-T + AMG 479 | 15 | 21.13% | 9 | 19.57% |  | 2 | 4.35% | 10 | 35.71% |  |  |
|  | AC-T + Ganetespib | 2 | 2.82% | 0 | 0.00% |  | 0 | 0.00% | 1 | 3.57% |  |  |
|  | AC-T + HKI-272 | 0 | 0.00% | 0 | 0.00% |  | 2 | 4.35% | 0 | 0.00% |  |  |
|  | Unknown | 0 | 0.00% | 0 | 0.00% |  | 0 | 0.00% | 1 | 3.57% |  |  |
| Abbreviations: NME non-mass-like enhancement, HR hormone receptor, HER2 human epidermal growth factor receptor 2, SBR grade Scarff-Bloom-Richardson grade, MRLD MRI measured longest diameter (cm) at baseline (T0) study, pCR pathologic complete response, AC-T Paclitaxel, AMG 479 Ganitumab, T-DM1 Trastuzumab, HKI-272 Neratinib | | | | | | | | | | | |  |
|  |  |  |  |  |  |  |  |  |  |  |  |  |

# Image repeatability and reproducibility ICC


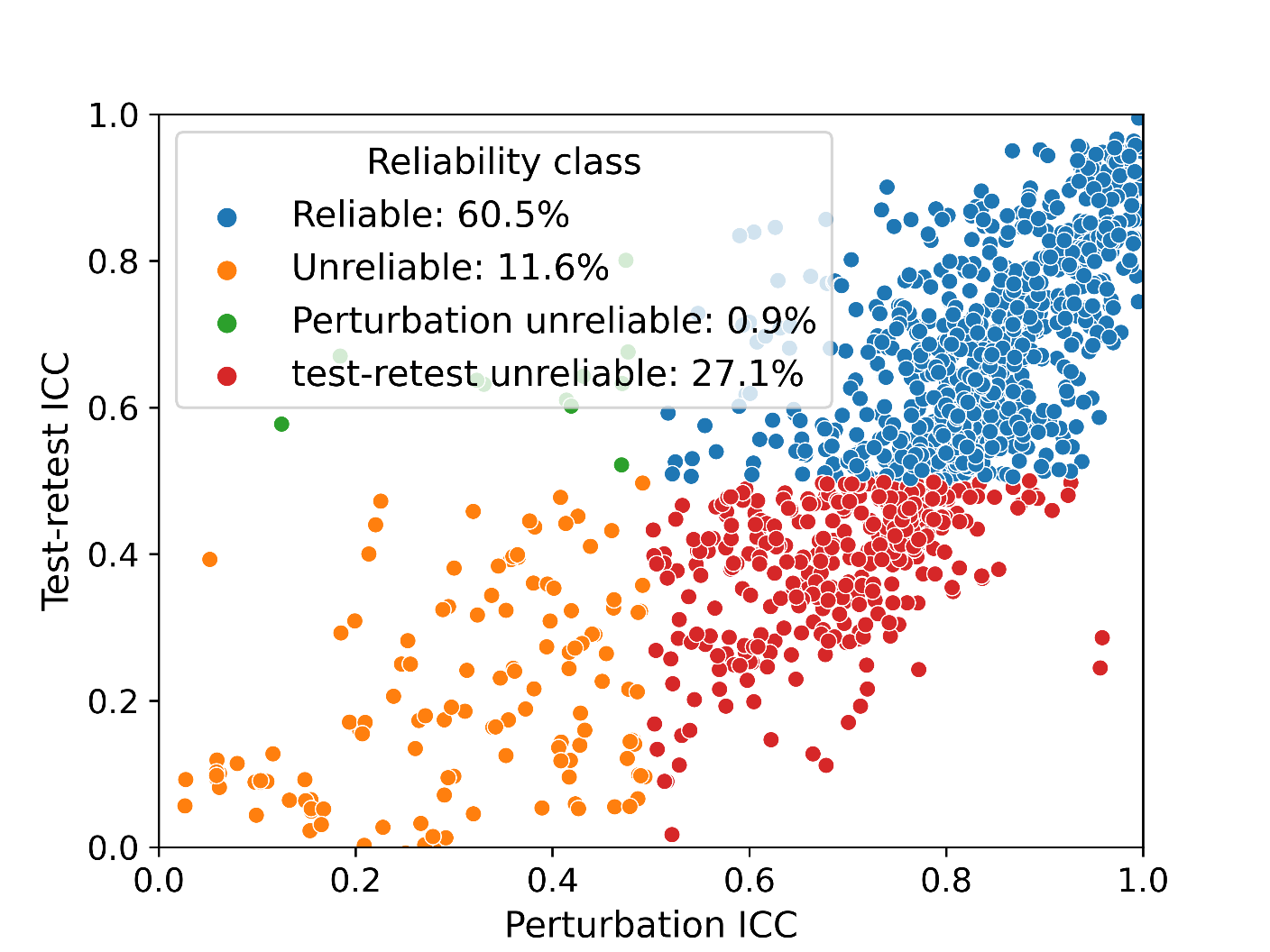


Figure S2 Image feature perturbation repeatability ICC (x-axis) and test-retest reproducibility ICC (y-axis).

# Image signature of HER2

Table S5 The image features and coefficients of Image signature for HER2 assessment. The corresponding test-retest ICC and perturbation ICC for each image features were also tabulated.

|  | coefficients | Test-retest ICC | Perturbation ICC |  |
| --- | --- | --- | --- | --- |
|  |  |  |  |  |
| wavelet-LLL_glcm_MaximumProbability | 3.942691198 | 0.79 | 0.95 |  |
| wavelet-LHL_glszm_SizeZoneNonUniformityNormalized | 0.66425085 | 0.67 | 0.86 |  |
| wavelet-LLH_glszm_ZoneEntropy | -0.057124361 | 0.84 | 0.93 |  |
| log-sigma-6-0-mm-3D_glszm_SizeZoneNonUniformityNormalized | 0.37826265 | 0.77 | 0.83 |  |
| wavelet-HHH_glcm_DifferenceVariance | 1.462787266 | 0.83 | 0.88 |  |
| intercept | -0.046558045 |  |  |  |

# Image signature of HR

Table S6 The image features and coefficients of Image signature for HR assessment. The corresponding test-retest ICC and perturbation ICC for each image features were also tabulated.

|  | coefficients | Test-retest ICC | Perturbation ICC |  |
| --- | --- | --- | --- | --- |
|  |  |  |  |  |
| log-sigma-1-0-mm-3D_glcm_InverseVariance | -0.480031525 | 0.83 | 0.89 |  |
| original_shape_Elongation | -0.161634264 | 0.79 | 0.94 |  |
| wavelet-HLH_glcm_ClusterShade | -0.112565945 | 0.73 | 0.93 |  |
| wavelet-HHH_glcm_MaximumProbability | -1.775958195 | 0.82 | 0.87 |  |
| wavelet-LHL_glcm_InverseVariance | -0.791255243 | 0.88 | 0.92 |  |
| intercept | 0.295168917 |  |  |  |

# Image Perturbation Algorithm

We have developed our own image perturbation algorithm that contains translation, rotation, and contour randomization to simulate test-retest imaging in clinic. Image translation displaces the image by a distance within the voxel size, and image rotation rotates the image along the axial axis at the center of the ROI bounding box. Contour randomization was achieved by deforming the original tumor segmentation by a 3-dimensional random displacement field.


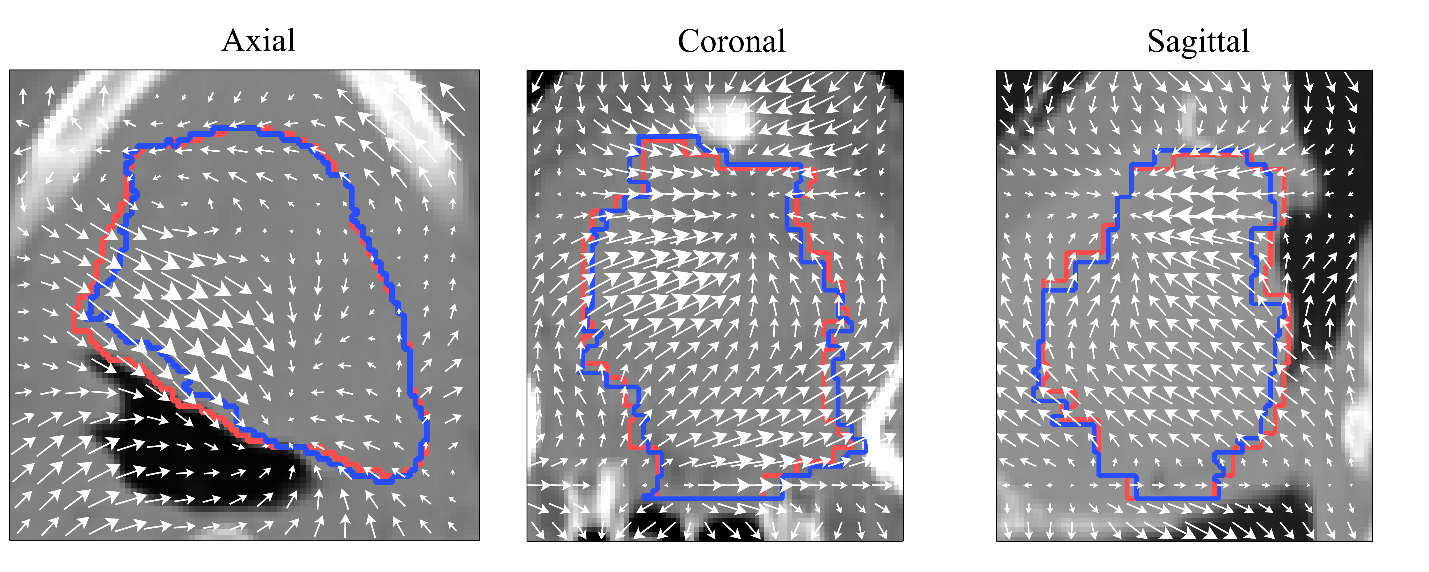


Figure S3. Vector field of a random displacement field and the corresponding original (red) and deformed (blue) contours.


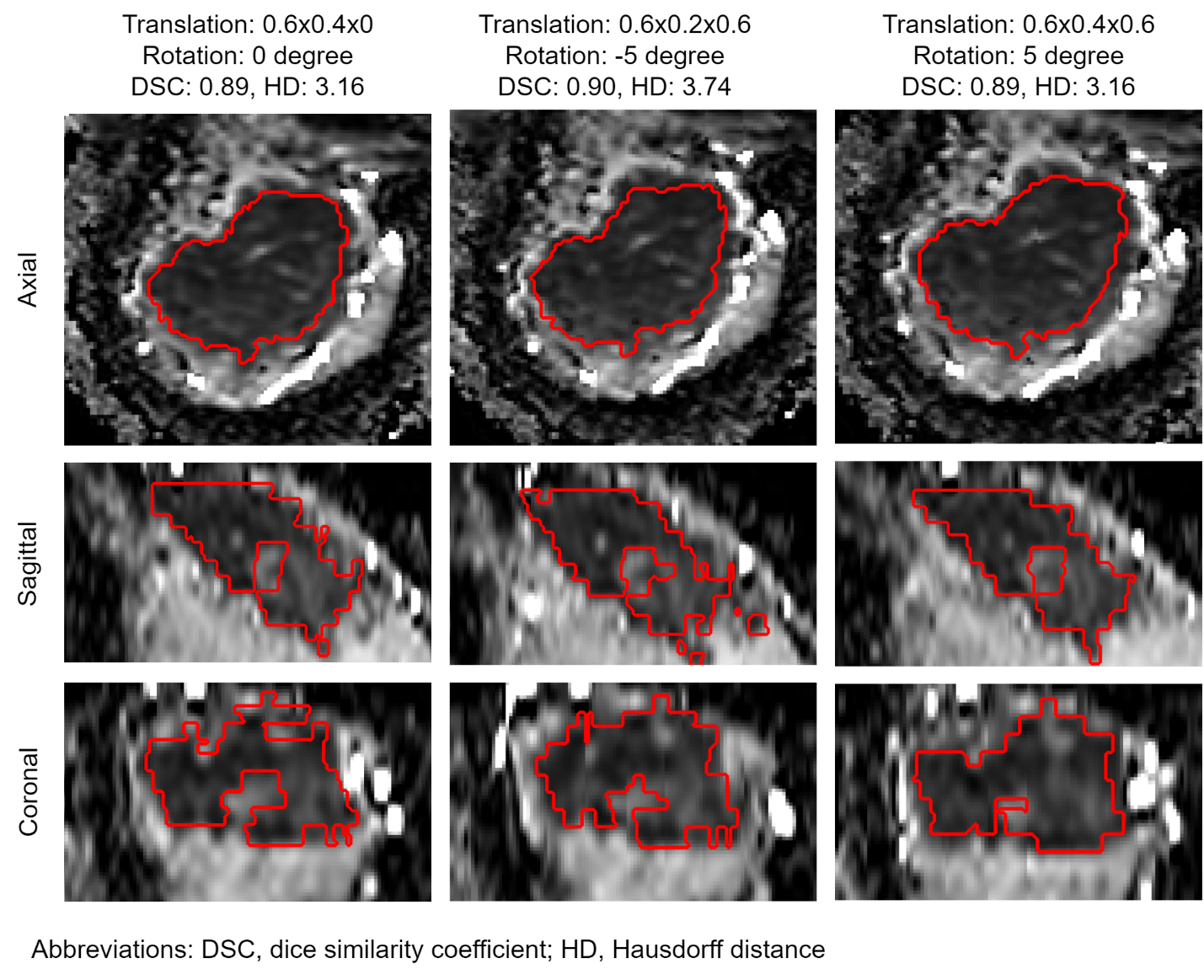


Figure S4. Demonstration of three image perturbations of one example ADC map in axial, sagittal, and coronal view. The target contours are drawn in red.

The algorithm of random displacement field generation was adapted from the methodology proposed by Simard et al [1]. A random field vector component on each dimension is generated randomly under a uniform distribution between -1 and 1 for each voxel point. All the z-component of the field vectors on the same slice were kept to the same value to mimic the uniform inter-slice contour variations from the slice-by-slice contouring. The field vectors were then normalized on each dimension by the root mean square and scaled by the user-defined intensity value. They were then smoothed by a gaussian filter with user-defined sigma to ensure the continuous change of the random displacement field and avoid sharp changes of the deformed contours. One example of random displacement field of one slice on the three directions were shown in Figure S3, where the original and the corresponding randomized contour were shown by the red and blue lines respectively.

Figure S4 demonstrated three perturbations of one example ADC in the three image views.

# Intra-class Correlation Coefficient

$$ICC(1,1)= \frac{MS_{R}-MS_{W}}{MS_{R}+\left( k+1 \right)MS_{W}}$$

$$ICC(2,1)= \frac{MS_{R}-MS_{E}}{MS_{R}+\left( k-1 \right)MS_{E}+\frac{k}{n}(MS_{C}-MS_{E})}$$

$MS_{R}$ represents the mean square of perturbations for each patient. $MS_{W}$ is the residual source of variance, which is calculated as the variance of perturbation values averaged across patients. $MS_{E}$ is the mean square of error, which is calculated as error between test and retest features. $k$ is the number of perturbations. $n$ is the number of subjects. The ICC (1,1) and ICC (2,1) quantified the image signatures’ repeatability and reproducibility respectively.

1. Simard PY, Steinkraus D, Platt JC (2003) Best practices for convolutional neural networks applied to visual document analysis. In: Seventh International Conference on Document Analysis and Recognition, 2003. Proceedings. IEEE Comput. Soc, Edinburgh, UK, pp 958–963

# Example patients


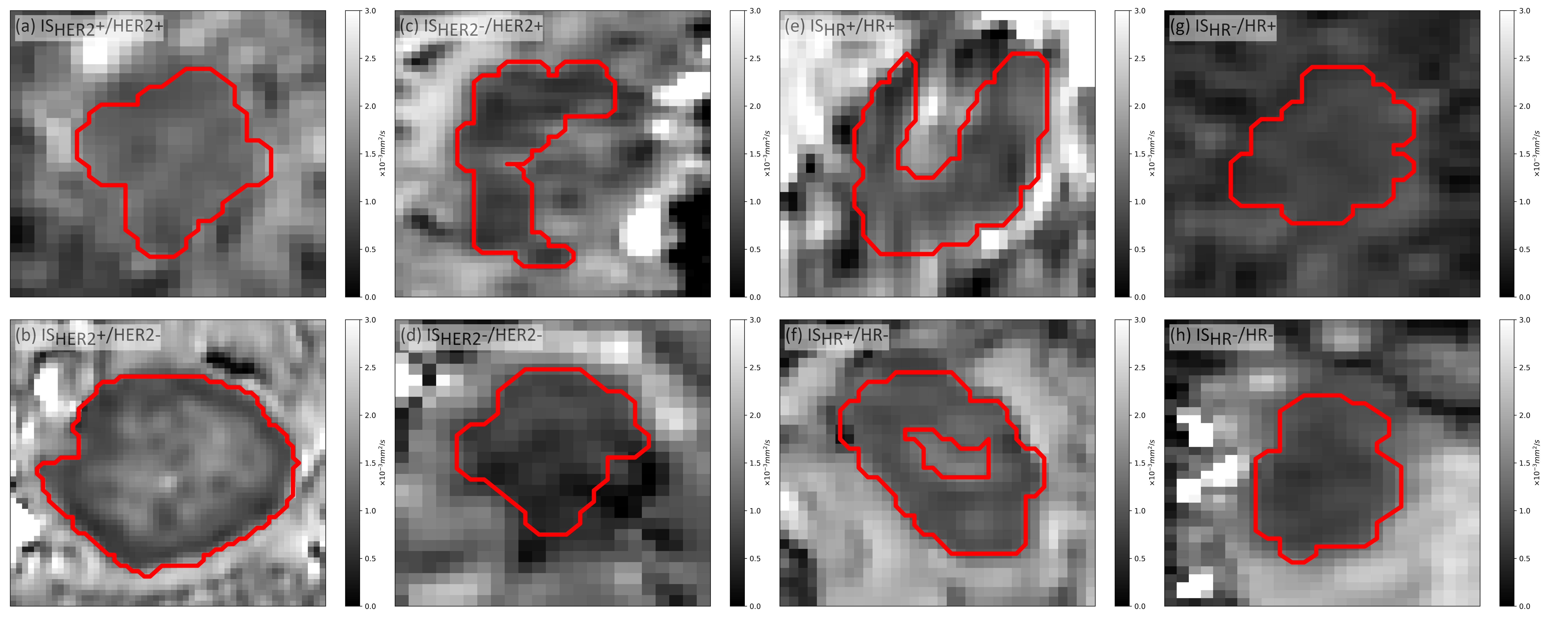


Figure S5 The axial slice of ADC maps of example patients with tumor contour, illustrating the consistency and inconsistency between image signatures and IHC receptor status., where IS_HER2+ and HER2+, (b) IS_HER2+ and HER2-, (c) IS_HER2- and HER2+, (b) IS_HER2- and HER2-, (e) IS_HR+ and HR+, (f) IS_HR+ and HR-, (g) IS_HR- and HR+, (b) IS_HR- and HR-. The display window is fixed between 0 x 10e-3 mm2/s to 3 x 10e-3 mm2/s.

# Subgroup Analysis

Table S7 The association between IS_HER2 and pCR under IHC-receptor subtypes. The IS_HER2 showed significant association to pCR in HR+ and HER2-HR+ groups. NA: all samples in the subgroup achieve pCR, thus the AUC cannot be evaluated.

|  | Discovery | | | |  | Validation | | | |
| --- | --- | --- | --- | --- | --- | --- | --- | --- | --- |
|  | AUC | 95% Confidence Interval | | P value |  | AUC | 95% Confidence Interval | | P value |
| HER2+ | 0.57 | 0.35 | 0.79 | 0.506 |  | 0.55 | 0.26 | 0.84 | 0.722 |
| HER2- | 0.61 | 0.47 | 0.75 | 0.130 |  | 0.59 | 0.38 | 0.80 | 0.337 |
| HR+ | 0.70 | 0.56 | 0.85 | **0.013** |  | 0.77 | 0.61 | 0.93 | **0.010** |
| HR- | 0.62 | 0.45 | 0.79 | 0.169 |  | 0.55 | 0.31 | 0.79 | 0.662 |
| HER2+HR+ | 0.74 | 0.49 | 0.99 | 0.076 |  | 0.58 | 0.18 | 0.97 | 0.661 |
| HER2+HR- | 0.56 | -0.09 | 1.00 | 0.794 |  | NA | NA | NA | NA |
| HER2-HR+ | 0.63 | 0.43 | 0.84 | 0.233 |  | 0.83 | 0.60 | 1.00 | **0.021** |
| HER2-HR- | 0.63 | 0.42 | 0.83 | 0.227 |  | 0.41 | 0.12 | 0.70 | 0.478 |

Table S8 The association between IS_HR and pCR under IHC-receptor subtypes. The IS_HR showed significant association to pCR in HER2- and HER2-HR+ groups. NA: all samples in the subgroup achieve pCR, thus the AUC cannot be evaluated.

|  | Discovery | | | |  | Validation | | | |
| --- | --- | --- | --- | --- | --- | --- | --- | --- | --- |
|  | AUC | 95% Confidence Interval | | P value |  | AUC | 95% Confidence Interval | | P value |
| HER2+ | 0.49 | 0.27 | 0.71 | 0.934 |  | 0.71 | 0.46 | 0.13 | 0.131 |
| HER2- | 0.74 | 0.62 | 0.86 | **0.001** |  | 0.73 | 0.57 | 0.01 | **0.013** |
| HR+ | 0.58 | 0.41 | 0.74 | 0.364 |  | 0.61 | 0.39 | 0.30 | 0.299 |
| HR- | 0.62 | 0.45 | 0.79 | 0.163 |  | 0.62 | 0.40 | 0.30 | 0.300 |
| HER2+HR+ | 0.63 | 0.38 | 0.89 | 0.316 |  | 0.55 | 0.17 | 0.77 | 0.770 |
| HER2+HR- | 0.69 | 0.05 | 1.00 | 0.433 |  | NA | NA | NA | NA |
| HER2-HR+ | 0.72 | 0.51 | 0.93 | **0.046** |  | 0.79 | 0.61 | 0.04 | **0.045** |
| HER2-HR- | 0.73 | 0.55 | 0.91 | **0.029** |  | 0.70 | 0.44 | 0.12 | 0.121 |

Table S9 The p value of variables in multi-variable logistic regression in predicting pCR. The image signatures were independently associated with pCR in multi-variable logistic regression.

|  | HER2 | HR | IS_HER2 | IS_HR |
| --- | --- | --- | --- | --- |
| pCR ~ HER2 + HR | 0.0002 | 0.0011 | / | / |
| pCR ~ HER2 + HR + IS_HER2 | 0.0025 | 0.0008 | 0.0196 | / |
| pCR ~ HER2 + HR + IS_HR | 0.0001 | 0.0097 | / | 0.0205 |
| pCR ~ HER2 + HR + IS_HR + IS_HER2 | 0.0014 | 0.0080 | 0.0244 | 0.0242 |

Table S10 The multivariable logistic regression to predict pCR, and evaluated with AUC. The P value is calculated with Delong Test.

|  | AUC | 95% CI lower | 95% CI upper | P value |
| --- | --- | --- | --- | --- |
| pCR ~ HER2 + HR | 0.71 | 0.63 | 0.79 | ref. |
| pCR ~ HER2 + HR + IS_HER2 | 0.75 | 0.68 | 0.82 | 0.034 |
| pCR ~ HER2 + HR + IS_HR | 0.76 | 0.69 | 0.83 | 0.004 |
| pCR ~ HER2 + HR + IS_HR + IS_HER2 | 0.78 | 0.71 | 0.84 | 0.002 |

# Image feature reproducibility ICC

See addition excel file: test_perturbation_icc.csv and test-retest_icc.csv

Table S11 The image feature ICC (2,1) for every single features.

|  | ICC value | Lower 95% CI | Higher 95% CI |
| --- | --- | --- | --- |
| original_shape_Elongation | 0.6963268 | 0.553722551 | 0.799221205 |
| original_shape_Flatness | 0.8640784 | 0.790920951 | 0.912936527 |
| original_shape_LeastAxisLength | 0.8568969 | 0.77972262 | 0.908376971 |
| original_shape_MajorAxisLength | 0.86757 | 0.795668405 | 0.915344783 |
| original_shape_Maximum2DDiameterColumn | 0.8315075 | 0.743351728 | 0.891357702 |
| original_shape_Maximum2DDiameterRow | 0.855383 | 0.778200457 | 0.907183716 |
| original_shape_Maximum2DDiameterSlice | 0.8986003 | 0.842503277 | 0.935465227 |
| original_shape_Maximum3DDiameter | 0.9184584 | 0.872497321 | 0.948330034 |
| original_shape_MeshVolume | 0.9551442 | 0.928701981 | 0.97187312 |
| original_shape_MinorAxisLength | 0.7441506 | 0.619778792 | 0.832186485 |
| original_shape_Sphericity | 0.7792312 | 0.66869199 | 0.856186414 |
| original_shape_SurfaceArea | 0.9490291 | 0.919107408 | 0.968007216 |
| original_shape_SurfaceVolumeRatio | 0.7937418 | 0.688972322 | 0.866086178 |
| original_shape_VoxelVolume | 0.9553189 | 0.928958158 | 0.971988027 |
| original_firstorder_10Percentile_32_binCount | 0.2446307 | 0.014128753 | 0.450630943 |
| original_firstorder_90Percentile_32_binCount | 0.9515662 | 0.923546499 | 0.969495949 |
| original_firstorder_Energy_32_binCount | 0.9959856 | 0.99357042 | 0.997494842 |
| original_firstorder_Entropy_32_binCount | 0.5973799 | 0.425462107 | 0.727938331 |
| original_firstorder_InterquartileRange_32_binCount | 0.5128339 | 0.320237543 | 0.665041265 |
| original_firstorder_Kurtosis_32_binCount | 0.7082869 | 0.569600389 | 0.80767603 |
| original_firstorder_Maximum_32_binCount | 0.7083964 | 0.571023928 | 0.807342587 |
| original_firstorder_MeanAbsoluteDeviation_32_binCount | 0.6569474 | 0.502585692 | 0.770948151 |
| original_firstorder_Mean_32_binCount | 0.8987421 | 0.842439644 | 0.935631333 |
| original_firstorder_Median_32_binCount | 0.9580052 | 0.933507356 | 0.973602754 |
| original_firstorder_Minimum_32_binCount | 0.6420953 | 0.481325687 | 0.760936279 |
| original_firstorder_Range_32_binCount | 0.7241817 | 0.592511368 | 0.818329089 |
| original_firstorder_RobustMeanAbsoluteDeviation_32_binCount | 0.4802419 | 0.281261199 | 0.640059467 |
| original_firstorder_RootMeanSquared_32_binCount | 0.9410122 | 0.907029384 | 0.962812217 |
| original_firstorder_Skewness_32_binCount | 0.5760773 | 0.396339338 | 0.71308457 |
| original_firstorder_TotalEnergy_32_binCount | 0.9959856 | 0.99357042 | 0.997494842 |
| original_firstorder_Uniformity_32_binCount | 0.6325789 | 0.470371392 | 0.753606045 |
| original_firstorder_Variance_32_binCount | 0.7376378 | 0.610879513 | 0.827670631 |
| original_glcm_Autocorrelation_32_binCount | 0.436434 | 0.231103677 | 0.605419294 |
| original_glcm_ClusterProminence_32_binCount | 0.3528667 | 0.131384948 | 0.540694581 |
| original_glcm_ClusterShade_32_binCount | 0.421445 | 0.21024462 | 0.595103357 |
| original_glcm_ClusterTendency_32_binCount | 0.4274454 | 0.219074768 | 0.599033924 |
| original_glcm_Contrast_32_binCount | 0.650262 | 0.493286838 | 0.76634663 |
| original_glcm_Correlation_32_binCount | 0.5746383 | 0.394567612 | 0.712010188 |
| original_glcm_DifferenceAverage_32_binCount | 0.629956 | 0.466484191 | 0.751879243 |
| original_glcm_DifferenceEntropy_32_binCount | 0.6010151 | 0.429861093 | 0.730676866 |
| original_glcm_DifferenceVariance_32_binCount | 0.6581508 | 0.504019403 | 0.771855533 |
| original_glcm_Id_32_binCount | 0.6360972 | 0.474729646 | 0.756208411 |
| original_glcm_Idm_32_binCount | 0.6301506 | 0.46690286 | 0.751962096 |
| original_glcm_Idmn_32_binCount | 0.6449138 | 0.486305157 | 0.762511328 |
| original_glcm_Idn_32_binCount | 0.6274421 | 0.463218262 | 0.750069083 |
| original_glcm_Imc1_32_binCount | 0.6011571 | 0.430211005 | 0.7307207 |
| original_glcm_Imc2_32_binCount | 0.6720409 | 0.520820032 | 0.782249365 |
| original_glcm_InverseVariance_32_binCount | 0.3990715 | 0.188583558 | 0.575624899 |
| original_glcm_JointAverage_32_binCount | 0.4557246 | 0.25137823 | 0.621442313 |
| original_glcm_JointEnergy_32_binCount | 0.604222 | 0.432291681 | 0.73360888 |
| original_glcm_JointEntropy_32_binCount | 0.5830234 | 0.407374182 | 0.717368452 |
| original_glcm_MCC_32_binCount | 0.5914881 | 0.418332964 | 0.723497346 |
| original_glcm_MaximumProbability_32_binCount | 0.6460548 | 0.487318393 | 0.763495314 |
| original_glcm_SumAverage_32_binCount | 0.4557246 | 0.25137823 | 0.621442313 |
| original_glcm_SumEntropy_32_binCount | 0.5868406 | 0.412487039 | 0.720071673 |
| original_glcm_SumSquares_32_binCount | 0.4563781 | 0.253204816 | 0.621521124 |
| original_glrlm_GrayLevelNonUniformity_32_binCount | 0.9414799 | 0.905940486 | 0.963561852 |
| original_glrlm_GrayLevelNonUniformityNormalized_32_binCount | 0.6169811 | 0.450796751 | 0.742126356 |
| original_glrlm_GrayLevelVariance_32_binCount | 0.4887789 | 0.29236894 | 0.646249825 |
| original_glrlm_HighGrayLevelRunEmphasis_32_binCount | 0.4428938 | 0.237953543 | 0.610766313 |
| original_glrlm_LongRunEmphasis_32_binCount | 0.6785762 | 0.529510619 | 0.786871808 |
| original_glrlm_LongRunHighGrayLevelEmphasis_32_binCount | 0.2863942 | 0.066967207 | 0.481829777 |
| original_glrlm_LongRunLowGrayLevelEmphasis_32_binCount | 0.6100194 | 0.440601421 | 0.737514451 |
| original_glrlm_LowGrayLevelRunEmphasis_32_binCount | 0.3232254 | 0.104714204 | 0.513627475 |
| original_glrlm_RunEntropy_32_binCount | 0.3549 | 0.140239487 | 0.539347789 |
| original_glrlm_RunLengthNonUniformity_32_binCount | 0.7400413 | 0.613042912 | 0.829686536 |
| original_glrlm_RunLengthNonUniformityNormalized_32_binCount | 0.6849944 | 0.539526548 | 0.790922735 |
| original_glrlm_RunPercentage_32_binCount | 0.7026574 | 0.563358038 | 0.803299434 |
| original_glrlm_RunVariance_32_binCount | 0.6736774 | 0.523087535 | 0.783377022 |
| original_glrlm_ShortRunEmphasis_32_binCount | 0.6770273 | 0.529112372 | 0.785227004 |
| original_glrlm_ShortRunHighGrayLevelEmphasis_32_binCount | 0.4834167 | 0.284298594 | 0.642793111 |
| original_glrlm_ShortRunLowGrayLevelEmphasis_32_binCount | 0.2437832 | 0.026887203 | 0.442915976 |
| original_glszm_GrayLevelNonUniformity_32_binCount | 0.6276309 | 0.462379522 | 0.750583305 |
| original_glszm_GrayLevelNonUniformityNormalized_32_binCount | 0.5090496 | 0.308446776 | 0.664962531 |
| original_glszm_GrayLevelVariance_32_binCount | 0.3959047 | 0.18415213 | 0.573452814 |
| original_glszm_HighGrayLevelZoneEmphasis_32_binCount | 0.3793382 | 0.164949147 | 0.560362514 |
| original_glszm_LargeAreaEmphasis_32_binCount | 0.7033693 | 0.563253928 | 0.804142293 |
| original_glszm_LargeAreaHighGrayLevelEmphasis_32_binCount | 0.8662299 | 0.793756795 | 0.914445236 |
| original_glszm_LargeAreaLowGrayLevelEmphasis_32_binCount | 0.6018525 | 0.430091547 | 0.731590393 |
| original_glszm_LowGrayLevelZoneEmphasis_32_binCount | 0.4105223 | 0.190936864 | 0.589411672 |
| original_glszm_SizeZoneNonUniformity_32_binCount | 0.4373953 | 0.232353877 | 0.606121393 |
| original_glszm_SizeZoneNonUniformityNormalized_32_binCount | 0.5277607 | 0.338135623 | 0.676429428 |
| original_glszm_SmallAreaEmphasis_32_binCount | 0.5034486 | 0.309196388 | 0.657776896 |
| original_glszm_SmallAreaHighGrayLevelEmphasis_32_binCount | 0.4228483 | 0.205532585 | 0.598931792 |
| original_glszm_SmallAreaLowGrayLevelEmphasis_32_binCount | 0.4773215 | 0.261074997 | 0.644478943 |
| original_glszm_ZoneEntropy_32_binCount | 0.5135845 | 0.322019655 | 0.665266154 |
| original_glszm_ZonePercentage_32_binCount | 0.6997749 | 0.559413937 | 0.801297951 |
| original_glszm_ZoneVariance_32_binCount | 0.7049217 | 0.565365202 | 0.80522352 |
| original_gldm_DependenceEntropy_32_binCount | 0.4744946 | 0.274576336 | 0.635569794 |
| original_gldm_DependenceNonUniformity_32_binCount | 0.8518529 | 0.772770283 | 0.904922309 |
| original_gldm_DependenceNonUniformityNormalized_32_binCount | 0.6858117 | 0.540700871 | 0.791472378 |
| original_gldm_DependenceVariance_32_binCount | 0.7490899 | 0.626297944 | 0.83568076 |
| original_gldm_GrayLevelNonUniformity_32_binCount | 0.9046715 | 0.851523389 | 0.939438883 |
| original_gldm_GrayLevelVariance_32_binCount | 0.4910977 | 0.29495841 | 0.648103363 |
| original_gldm_HighGrayLevelEmphasis_32_binCount | 0.445956 | 0.241588692 | 0.613137121 |
| original_gldm_LargeDependenceEmphasis_32_binCount | 0.7048483 | 0.566045709 | 0.804921263 |
| original_gldm_LargeDependenceHighGrayLevelEmphasis_32_binCount | 0.5070494 | 0.312601459 | 0.660893988 |
| original_gldm_LargeDependenceLowGrayLevelEmphasis_32_binCount | 0.6315959 | 0.469601657 | 0.75272138 |
| original_gldm_LowGrayLevelEmphasis_32_binCount | 0.3168396 | 0.098416909 | 0.50801204 |
| original_gldm_SmallDependenceEmphasis_32_binCount | 0.6898268 | 0.545510202 | 0.794484428 |
| original_gldm_SmallDependenceHighGrayLevelEmphasis_32_binCount | 0.6787219 | 0.523436988 | 0.789004894 |
| original_gldm_SmallDependenceLowGrayLevelEmphasis_32_binCount | 0.508324 | 0.314159504 | 0.661856404 |
| original_ngtdm_Busyness_32_binCount | 0.8620727 | 0.774637216 | 0.915051752 |
| original_ngtdm_Coarseness_32_binCount | 0.8505962 | 0.770733722 | 0.904146014 |
| original_ngtdm_Complexity_32_binCount | 0.4585228 | 0.256841049 | 0.622725741 |
| original_ngtdm_Contrast_32_binCount | 0.6623307 | 0.509568519 | 0.774821718 |
| original_ngtdm_Strength_32_binCount | 0.7286854 | 0.598149358 | 0.821617959 |
| log-sigma-1-mm-3D_firstorder_10Percentile_32_binCount | 0.7779084 | 0.666541728 | 0.8553732 |
| log-sigma-1-mm-3D_firstorder_90Percentile_32_binCount | 0.5860121 | 0.409491354 | 0.720168691 |
| log-sigma-1-mm-3D_firstorder_Energy_32_binCount | 0.5864091 | 0.411353682 | 0.71997001 |
| log-sigma-1-mm-3D_firstorder_Entropy_32_binCount | 0.5202992 | 0.330239224 | 0.670334195 |
| log-sigma-1-mm-3D_firstorder_InterquartileRange_32_binCount | 0.5590017 | 0.376303363 | 0.699960239 |
| log-sigma-1-mm-3D_firstorder_Kurtosis_32_binCount | 0.3573058 | 0.134910375 | 0.544929058 |
| log-sigma-1-mm-3D_firstorder_Maximum_32_binCount | 0.730825 | 0.600731518 | 0.823209151 |
| log-sigma-1-mm-3D_firstorder_MeanAbsoluteDeviation_32_binCount | 0.7047901 | 0.566030353 | 0.804860056 |
| log-sigma-1-mm-3D_firstorder_Mean_32_binCount | 0.8741263 | 0.805899173 | 0.919505024 |
| log-sigma-1-mm-3D_firstorder_Median_32_binCount | 0.840613 | 0.756504858 | 0.897433572 |
| log-sigma-1-mm-3D_firstorder_Minimum_32_binCount | 0.6763579 | 0.527939752 | 0.784845537 |
| log-sigma-1-mm-3D_firstorder_Range_32_binCount | 0.731387 | 0.602250005 | 0.823362174 |
| log-sigma-1-mm-3D_firstorder_RobustMeanAbsoluteDeviation_32_binCount | 0.5716428 | 0.39242848 | 0.709208386 |
| log-sigma-1-mm-3D_firstorder_RootMeanSquared_32_binCount | 0.7864563 | 0.678739001 | 0.861132395 |
| log-sigma-1-mm-3D_firstorder_Skewness_32_binCount | 0.215548 | -0.01998479 | 0.42754925 |
| log-sigma-1-mm-3D_firstorder_TotalEnergy_32_binCount | 0.5864091 | 0.411353682 | 0.71997001 |
| log-sigma-1-mm-3D_firstorder_Uniformity_32_binCount | 0.5651984 | 0.384861183 | 0.704253222 |
| log-sigma-1-mm-3D_firstorder_Variance_32_binCount | 0.7110715 | 0.57447446 | 0.80926754 |
| log-sigma-1-mm-3D_glcm_Autocorrelation_32_binCount | 0.1461837 | -0.08222536 | 0.36210583 |
| log-sigma-1-mm-3D_glcm_ClusterProminence_32_binCount | 0.4188793 | 0.208127483 | 0.592722795 |
| log-sigma-1-mm-3D_glcm_ClusterShade_32_binCount | 0.4528639 | 0.24988002 | 0.61845204 |
| log-sigma-1-mm-3D_glcm_ClusterTendency_32_binCount | 0.4577979 | 0.254881235 | 0.62262241 |
| log-sigma-1-mm-3D_glcm_Contrast_32_binCount | 0.4876533 | 0.291063569 | 0.645369281 |
| log-sigma-1-mm-3D_glcm_Correlation_32_binCount | 0.4784857 | 0.276153409 | 0.639911049 |
| log-sigma-1-mm-3D_glcm_DifferenceAverage_32_binCount | 0.5159797 | 0.325057671 | 0.667033611 |
| log-sigma-1-mm-3D_glcm_DifferenceEntropy_32_binCount | 0.5186176 | 0.328238708 | 0.669041907 |
| log-sigma-1-mm-3D_glcm_DifferenceVariance_32_binCount | 0.5086701 | 0.316222912 | 0.661472624 |
| log-sigma-1-mm-3D_glcm_Id_32_binCount | 0.5562667 | 0.374310672 | 0.697397877 |
| log-sigma-1-mm-3D_glcm_Idm_32_binCount | 0.5503086 | 0.36695081 | 0.692942936 |
| log-sigma-1-mm-3D_glcm_Idmn_32_binCount | 0.4869461 | 0.290214325 | 0.644827916 |
| log-sigma-1-mm-3D_glcm_Idn_32_binCount | 0.5196968 | 0.329551626 | 0.669859237 |
| log-sigma-1-mm-3D_glcm_Imc1_32_binCount | 0.5755911 | 0.398281302 | 0.711795567 |
| log-sigma-1-mm-3D_glcm_Imc2_32_binCount | 0.6669296 | 0.51427048 | 0.778550824 |
| log-sigma-1-mm-3D_glcm_InverseVariance_32_binCount | 0.3075053 | 0.085695939 | 0.501473626 |
| log-sigma-1-mm-3D_glcm_JointAverage_32_binCount | 0.1681948 | -0.05965303 | 0.38149014 |
| log-sigma-1-mm-3D_glcm_JointEnergy_32_binCount | 0.5980028 | 0.425096461 | 0.728811303 |
| log-sigma-1-mm-3D_glcm_JointEntropy_32_binCount | 0.5102298 | 0.318086413 | 0.662669623 |
| log-sigma-1-mm-3D_glcm_MCC_32_binCount | 0.3984713 | 0.184799336 | 0.57649968 |
| log-sigma-1-mm-3D_glcm_MaximumProbability_32_binCount | 0.5743651 | 0.395962875 | 0.71117651 |
| log-sigma-1-mm-3D_glcm_SumAverage_32_binCount | 0.1681948 | -0.05965303 | 0.38149014 |
| log-sigma-1-mm-3D_glcm_SumEntropy_32_binCount | 0.5178793 | 0.327214574 | 0.668534056 |
| log-sigma-1-mm-3D_glcm_SumSquares_32_binCount | 0.4658516 | 0.264637373 | 0.628765566 |
| log-sigma-1-mm-3D_glrlm_GrayLevelNonUniformity_32_binCount | 0.935593 | 0.895506867 | 0.96013119 |
| log-sigma-1-mm-3D_glrlm_GrayLevelNonUniformityNormalized_32_binCount | 0.5207855 | 0.330496321 | 0.670833296 |
| log-sigma-1-mm-3D_glrlm_GrayLevelVariance_32_binCount | 0.4779183 | 0.279447119 | 0.637884021 |
| log-sigma-1-mm-3D_glrlm_HighGrayLevelRunEmphasis_32_binCount | 0.1461038 | -0.08219101 | 0.361970521 |
| log-sigma-1-mm-3D_glrlm_LongRunEmphasis_32_binCount | 0.6898826 | 0.544944436 | 0.794735014 |
| log-sigma-1-mm-3D_glrlm_LongRunHighGrayLevelEmphasis_32_binCount | 0.5375407 | 0.348852322 | 0.684272717 |
| log-sigma-1-mm-3D_glrlm_LongRunLowGrayLevelEmphasis_32_binCount | 0.2850002 | 0.059127369 | 0.483762893 |
| log-sigma-1-mm-3D_glrlm_LowGrayLevelRunEmphasis_32_binCount | 0.2520935 | 0.027160169 | 0.454410276 |
| log-sigma-1-mm-3D_glrlm_RunEntropy_32_binCount | 0.2484543 | 0.024386248 | 0.450762862 |
| log-sigma-1-mm-3D_glrlm_RunLengthNonUniformity_32_binCount | 0.7368392 | 0.608629064 | 0.827478982 |
| log-sigma-1-mm-3D_glrlm_RunLengthNonUniformityNormalized_32_binCount | 0.6238833 | 0.459670639 | 0.747133519 |
| log-sigma-1-mm-3D_glrlm_RunPercentage_32_binCount | 0.64602 | 0.488324996 | 0.763110431 |
| log-sigma-1-mm-3D_glrlm_RunVariance_32_binCount | 0.712375 | 0.57520551 | 0.810507723 |
| log-sigma-1-mm-3D_glrlm_ShortRunEmphasis_32_binCount | 0.6249785 | 0.461023809 | 0.747947833 |
| log-sigma-1-mm-3D_glrlm_ShortRunHighGrayLevelEmphasis_32_binCount | 0.1185213 | -0.11089338 | 0.337835129 |
| log-sigma-1-mm-3D_glrlm_ShortRunLowGrayLevelEmphasis_32_binCount | 0.2813946 | 0.058607329 | 0.479102589 |
| log-sigma-1-mm-3D_glszm_GrayLevelNonUniformity_32_binCount | 0.7251557 | 0.592799483 | 0.819334933 |
| log-sigma-1-mm-3D_glszm_GrayLevelNonUniformityNormalized_32_binCount | 0.2791297 | 0.058395056 | 0.476098184 |
| log-sigma-1-mm-3D_glszm_GrayLevelVariance_32_binCount | 0.4138501 | 0.202338482 | 0.588749469 |
| log-sigma-1-mm-3D_glszm_HighGrayLevelZoneEmphasis_32_binCount | 0.1407822 | -0.08636995 | 0.356557634 |
| log-sigma-1-mm-3D_glszm_LargeAreaEmphasis_32_binCount | 0.6923579 | 0.548022948 | 0.796553246 |
| log-sigma-1-mm-3D_glszm_LargeAreaHighGrayLevelEmphasis_32_binCount | 0.5972792 | 0.423450494 | 0.7285399 |
| log-sigma-1-mm-3D_glszm_LargeAreaLowGrayLevelEmphasis_32_binCount | 0.6726326 | 0.523021034 | 0.782199369 |
| log-sigma-1-mm-3D_glszm_LowGrayLevelZoneEmphasis_32_binCount | 0.2767221 | 0.053216994 | 0.475356431 |
| log-sigma-1-mm-3D_glszm_SizeZoneNonUniformity_32_binCount | 0.4117111 | 0.203052343 | 0.585680003 |
| log-sigma-1-mm-3D_glszm_SizeZoneNonUniformityNormalized_32_binCount | 0.4360096 | 0.230780113 | 0.605016233 |
| log-sigma-1-mm-3D_glszm_SmallAreaEmphasis_32_binCount | 0.3959444 | 0.185307385 | 0.572998805 |
| log-sigma-1-mm-3D_glszm_SmallAreaHighGrayLevelEmphasis_32_binCount | 0.1392088 | -0.09456423 | 0.358829939 |
| log-sigma-1-mm-3D_glszm_SmallAreaLowGrayLevelEmphasis_32_binCount | 0.3772844 | 0.162765532 | 0.558649856 |
| log-sigma-1-mm-3D_glszm_ZoneEntropy_32_binCount | 0.6335364 | 0.47082669 | 0.754567299 |
| log-sigma-1-mm-3D_glszm_ZonePercentage_32_binCount | 0.5599296 | 0.378092219 | 0.700414913 |
| log-sigma-1-mm-3D_glszm_ZoneVariance_32_binCount | 0.6936204 | 0.549720558 | 0.797439244 |
| log-sigma-1-mm-3D_gldm_DependenceEntropy_32_binCount | 0.3332505 | 0.112038852 | 0.523596003 |
| log-sigma-1-mm-3D_gldm_DependenceNonUniformity_32_binCount | 0.8121934 | 0.714992821 | 0.878596692 |
| log-sigma-1-mm-3D_gldm_DependenceNonUniformityNormalized_32_binCount | 0.5806056 | 0.404593542 | 0.71549333 |
| log-sigma-1-mm-3D_gldm_DependenceVariance_32_binCount | 0.6699781 | 0.519774873 | 0.780224229 |
| log-sigma-1-mm-3D_gldm_GrayLevelNonUniformity_32_binCount | 0.8846225 | 0.821508063 | 0.926375375 |
| log-sigma-1-mm-3D_gldm_GrayLevelVariance_32_binCount | 0.4831618 | 0.285583494 | 0.641965777 |
| log-sigma-1-mm-3D_gldm_HighGrayLevelEmphasis_32_binCount | 0.1442565 | -0.08406272 | 0.360328211 |
| log-sigma-1-mm-3D_gldm_LargeDependenceEmphasis_32_binCount | 0.657188 | 0.5026365 | 0.771210209 |
| log-sigma-1-mm-3D_gldm_LargeDependenceHighGrayLevelEmphasis_32_binCount | 0.5286681 | 0.339079503 | 0.67718343 |
| log-sigma-1-mm-3D_gldm_LargeDependenceLowGrayLevelEmphasis_32_binCount | 0.3856686 | 0.167999268 | 0.567245711 |
| log-sigma-1-mm-3D_gldm_LowGrayLevelEmphasis_32_binCount | 0.2532343 | 0.028451835 | 0.455339468 |
| log-sigma-1-mm-3D_gldm_SmallDependenceEmphasis_32_binCount | 0.5599013 | 0.378570007 | 0.70020253 |
| log-sigma-1-mm-3D_gldm_SmallDependenceHighGrayLevelEmphasis_32_binCount | 0.4555101 | 0.249604513 | 0.621901263 |
| log-sigma-1-mm-3D_gldm_SmallDependenceLowGrayLevelEmphasis_32_binCount | 0.6009668 | 0.43014046 | 0.730523831 |
| log-sigma-1-mm-3D_ngtdm_Busyness_32_binCount | 0.8935718 | 0.834859916 | 0.932219862 |
| log-sigma-1-mm-3D_ngtdm_Coarseness_32_binCount | 0.8267133 | 0.736096478 | 0.888252395 |
| log-sigma-1-mm-3D_ngtdm_Complexity_32_binCount | 0.3656056 | 0.151587772 | 0.548331702 |
| log-sigma-1-mm-3D_ngtdm_Contrast_32_binCount | 0.488747 | 0.291621009 | 0.646510941 |
| log-sigma-1-mm-3D_ngtdm_Strength_32_binCount | 0.737474 | 0.609539873 | 0.827905509 |
| log-sigma-2-mm-3D_firstorder_10Percentile_32_binCount | 0.8708724 | 0.801070094 | 0.91737213 |
| log-sigma-2-mm-3D_firstorder_90Percentile_32_binCount | 0.846889 | 0.765312859 | 0.901692364 |
| log-sigma-2-mm-3D_firstorder_Energy_32_binCount | 0.7022712 | 0.562797668 | 0.803042249 |
| log-sigma-2-mm-3D_firstorder_Entropy_32_binCount | 0.6447843 | 0.484816978 | 0.762870352 |
| log-sigma-2-mm-3D_firstorder_InterquartileRange_32_binCount | 0.5463969 | 0.360649728 | 0.690572955 |
| log-sigma-2-mm-3D_firstorder_Kurtosis_32_binCount | 0.4005487 | 0.187216685 | 0.578134345 |
| log-sigma-2-mm-3D_firstorder_Maximum_32_binCount | 0.7897029 | 0.682750408 | 0.863503106 |
| log-sigma-2-mm-3D_firstorder_MeanAbsoluteDeviation_32_binCount | 0.7037074 | 0.564268296 | 0.804199315 |
| log-sigma-2-mm-3D_firstorder_Mean_32_binCount | 0.9435404 | 0.911117912 | 0.964379609 |
| log-sigma-2-mm-3D_firstorder_Median_32_binCount | 0.9269767 | 0.885356181 | 0.953847905 |
| log-sigma-2-mm-3D_firstorder_Minimum_32_binCount | 0.6796485 | 0.531001542 | 0.787608799 |
| log-sigma-2-mm-3D_firstorder_Range_32_binCount | 0.7693933 | 0.653912382 | 0.849781253 |
| log-sigma-2-mm-3D_firstorder_RobustMeanAbsoluteDeviation_32_binCount | 0.5585753 | 0.375999887 | 0.699559003 |
| log-sigma-2-mm-3D_firstorder_RootMeanSquared_32_binCount | 0.8543654 | 0.776577459 | 0.906548009 |
| log-sigma-2-mm-3D_firstorder_Skewness_32_binCount | 0.2640512 | 0.032530936 | 0.468165274 |
| log-sigma-2-mm-3D_firstorder_TotalEnergy_32_binCount | 0.7022712 | 0.562797668 | 0.803042249 |
| log-sigma-2-mm-3D_firstorder_Uniformity_32_binCount | 0.6555371 | 0.499350668 | 0.770408432 |
| log-sigma-2-mm-3D_firstorder_Variance_32_binCount | 0.7813681 | 0.671603546 | 0.857667517 |
| log-sigma-2-mm-3D_glcm_Autocorrelation_32_binCount | 0.0963119 | -0.14214809 | 0.322998316 |
| log-sigma-2-mm-3D_glcm_ClusterProminence_32_binCount | 0.4048943 | 0.189768164 | 0.582622862 |
| log-sigma-2-mm-3D_glcm_ClusterShade_32_binCount | 0.4798409 | 0.279711104 | 0.640180631 |
| log-sigma-2-mm-3D_glcm_ClusterTendency_32_binCount | 0.5606582 | 0.377000335 | 0.701693531 |
| log-sigma-2-mm-3D_glcm_Contrast_32_binCount | 0.698772 | 0.556859987 | 0.800985377 |
| log-sigma-2-mm-3D_glcm_Correlation_32_binCount | 0.4114069 | 0.196910959 | 0.587920846 |
| log-sigma-2-mm-3D_glcm_DifferenceAverage_32_binCount | 0.7033475 | 0.562890999 | 0.804233853 |
| log-sigma-2-mm-3D_glcm_DifferenceEntropy_32_binCount | 0.6733637 | 0.522562941 | 0.783190486 |
| log-sigma-2-mm-3D_glcm_DifferenceVariance_32_binCount | 0.6911912 | 0.546616567 | 0.795681866 |
| log-sigma-2-mm-3D_glcm_Id_32_binCount | 0.7066419 | 0.567311539 | 0.806547116 |
| log-sigma-2-mm-3D_glcm_Idm_32_binCount | 0.703621 | 0.563205377 | 0.804442861 |
| log-sigma-2-mm-3D_glcm_Idmn_32_binCount | 0.6969286 | 0.554356719 | 0.799699891 |
| log-sigma-2-mm-3D_glcm_Idn_32_binCount | 0.7031884 | 0.56266069 | 0.804127527 |
| log-sigma-2-mm-3D_glcm_Imc1_32_binCount | 0.5523024 | 0.3665929 | 0.695487985 |
| log-sigma-2-mm-3D_glcm_Imc2_32_binCount | 0.4340078 | 0.224089111 | 0.605276695 |
| log-sigma-2-mm-3D_glcm_InverseVariance_32_binCount | 0.5566361 | 0.3722418 | 0.698614522 |
| log-sigma-2-mm-3D_glcm_JointAverage_32_binCount | 0.1121062 | -0.12631245 | 0.33713915 |
| log-sigma-2-mm-3D_glcm_JointEnergy_32_binCount | 0.6634067 | 0.510348211 | 0.775802767 |
| log-sigma-2-mm-3D_glcm_JointEntropy_32_binCount | 0.6384906 | 0.476586125 | 0.758363132 |
| log-sigma-2-mm-3D_glcm_MCC_32_binCount | 0.0661727 | -0.17098532 | 0.295123674 |
| log-sigma-2-mm-3D_glcm_MaximumProbability_32_binCount | 0.6859687 | 0.54004658 | 0.791868274 |
| log-sigma-2-mm-3D_glcm_SumAverage_32_binCount | 0.1121062 | -0.12631245 | 0.33713915 |
| log-sigma-2-mm-3D_glcm_SumEntropy_32_binCount | 0.6267219 | 0.461342284 | 0.749879684 |
| log-sigma-2-mm-3D_glcm_SumSquares_32_binCount | 0.5805866 | 0.402139944 | 0.716361132 |
| log-sigma-2-mm-3D_glrlm_GrayLevelNonUniformity_32_binCount | 0.9537396 | 0.926946211 | 0.97087267 |
| log-sigma-2-mm-3D_glrlm_GrayLevelNonUniformityNormalized_32_binCount | 0.6246191 | 0.458618563 | 0.748363133 |
| log-sigma-2-mm-3D_glrlm_GrayLevelVariance_32_binCount | 0.6341167 | 0.47082296 | 0.755244399 |
| log-sigma-2-mm-3D_glrlm_HighGrayLevelRunEmphasis_32_binCount | 0.0988304 | -0.13965297 | 0.325272224 |
| log-sigma-2-mm-3D_glrlm_LongRunEmphasis_32_binCount | 0.7412294 | 0.61551389 | 0.830247255 |
| log-sigma-2-mm-3D_glrlm_LongRunHighGrayLevelEmphasis_32_binCount | 0.5093501 | 0.314114363 | 0.663134037 |
| log-sigma-2-mm-3D_glrlm_LongRunLowGrayLevelEmphasis_32_binCount | 0.2423817 | 0.00852107 | 0.450317083 |
| log-sigma-2-mm-3D_glrlm_LowGrayLevelRunEmphasis_32_binCount | 0.3487918 | 0.125222566 | 0.538110996 |
| log-sigma-2-mm-3D_glrlm_RunEntropy_32_binCount | 0.3624889 | 0.140897139 | 0.549037555 |
| log-sigma-2-mm-3D_glrlm_RunLengthNonUniformity_32_binCount | 0.7263867 | 0.595451382 | 0.819882986 |
| log-sigma-2-mm-3D_glrlm_RunLengthNonUniformityNormalized_32_binCount | 0.7402888 | 0.613441389 | 0.829839426 |
| log-sigma-2-mm-3D_glrlm_RunPercentage_32_binCount | 0.7495656 | 0.626379341 | 0.836186823 |
| log-sigma-2-mm-3D_glrlm_RunVariance_32_binCount | 0.7590963 | 0.640442463 | 0.842470146 |
| log-sigma-2-mm-3D_glrlm_ShortRunEmphasis_32_binCount | 0.7270727 | 0.595286766 | 0.820706094 |
| log-sigma-2-mm-3D_glrlm_ShortRunHighGrayLevelEmphasis_32_binCount | 0.1211379 | -0.11729295 | 0.345230082 |
| log-sigma-2-mm-3D_glrlm_ShortRunLowGrayLevelEmphasis_32_binCount | 0.3810557 | 0.161860207 | 0.563948236 |
| log-sigma-2-mm-3D_glszm_GrayLevelNonUniformity_32_binCount | 0.7108111 | 0.573023611 | 0.809436486 |
| log-sigma-2-mm-3D_glszm_GrayLevelNonUniformityNormalized_32_binCount | 0.3547653 | 0.133781459 | 0.542110346 |
| log-sigma-2-mm-3D_glszm_GrayLevelVariance_32_binCount | 0.4218249 | 0.211252512 | 0.595163274 |
| log-sigma-2-mm-3D_glszm_HighGrayLevelZoneEmphasis_32_binCount | 0.0591859 | -0.17879866 | 0.289297859 |
| log-sigma-2-mm-3D_glszm_LargeAreaEmphasis_32_binCount | 0.7461542 | 0.622236933 | 0.833663089 |
| log-sigma-2-mm-3D_glszm_LargeAreaHighGrayLevelEmphasis_32_binCount | 0.6373638 | 0.476393046 | 0.757117705 |
| log-sigma-2-mm-3D_glszm_LargeAreaLowGrayLevelEmphasis_32_binCount | 0.7913171 | 0.685052507 | 0.864590235 |
| log-sigma-2-mm-3D_glszm_LowGrayLevelZoneEmphasis_32_binCount | 0.1925423 | -0.04409367 | 0.407797599 |
| log-sigma-2-mm-3D_glszm_SizeZoneNonUniformity_32_binCount | 0.3404275 | 0.116561791 | 0.531021592 |
| log-sigma-2-mm-3D_glszm_SizeZoneNonUniformityNormalized_32_binCount | 0.4729907 | 0.270041068 | 0.635514217 |
| log-sigma-2-mm-3D_glszm_SmallAreaEmphasis_32_binCount | 0.4484982 | 0.241287291 | 0.616482212 |
| log-sigma-2-mm-3D_glszm_SmallAreaHighGrayLevelEmphasis_32_binCount | 0.1435153 | -0.09426206 | 0.364857117 |
| log-sigma-2-mm-3D_glszm_SmallAreaLowGrayLevelEmphasis_32_binCount | 0.1336294 | -0.10410253 | 0.356011385 |
| log-sigma-2-mm-3D_glszm_ZoneEntropy_32_binCount | 0.7600156 | 0.641360833 | 0.843210212 |
| log-sigma-2-mm-3D_glszm_ZonePercentage_32_binCount | 0.7099444 | 0.57227252 | 0.808696849 |
| log-sigma-2-mm-3D_glszm_ZoneVariance_32_binCount | 0.748076 | 0.624904908 | 0.834982089 |
| log-sigma-2-mm-3D_gldm_DependenceEntropy_32_binCount | 0.5094214 | 0.313456444 | 0.663474564 |
| log-sigma-2-mm-3D_gldm_DependenceNonUniformity_32_binCount | 0.79002 | 0.68379905 | 0.863539966 |
| log-sigma-2-mm-3D_gldm_DependenceNonUniformityNormalized_32_binCount | 0.7455106 | 0.621206669 | 0.83326373 |
| log-sigma-2-mm-3D_gldm_DependenceVariance_32_binCount | 0.7878731 | 0.680275537 | 0.862230697 |
| log-sigma-2-mm-3D_gldm_GrayLevelNonUniformity_32_binCount | 0.8925769 | 0.833116159 | 0.931639716 |
| log-sigma-2-mm-3D_gldm_GrayLevelVariance_32_binCount | 0.6388589 | 0.477031065 | 0.758639391 |
| log-sigma-2-mm-3D_gldm_HighGrayLevelEmphasis_32_binCount | 0.097623 | -0.14085545 | 0.324185659 |
| log-sigma-2-mm-3D_gldm_LargeDependenceEmphasis_32_binCount | 0.7419995 | 0.616045166 | 0.830942774 |
| log-sigma-2-mm-3D_gldm_LargeDependenceHighGrayLevelEmphasis_32_binCount | 0.5397474 | 0.350993313 | 0.686141769 |
| log-sigma-2-mm-3D_gldm_LargeDependenceLowGrayLevelEmphasis_32_binCount | 0.3901884 | 0.172502402 | 0.571128434 |
| log-sigma-2-mm-3D_gldm_LowGrayLevelEmphasis_32_binCount | 0.3544504 | 0.131632244 | 0.54265609 |
| log-sigma-2-mm-3D_gldm_SmallDependenceEmphasis_32_binCount | 0.7149589 | 0.578801703 | 0.812280253 |
| log-sigma-2-mm-3D_gldm_SmallDependenceHighGrayLevelEmphasis_32_binCount | 0.7440649 | 0.618638907 | 0.832444587 |
| log-sigma-2-mm-3D_gldm_SmallDependenceLowGrayLevelEmphasis_32_binCount | 0.4444823 | 0.235478634 | 0.613804515 |
| log-sigma-2-mm-3D_ngtdm_Busyness_32_binCount | 0.955857 | 0.930184938 | 0.972232467 |
| log-sigma-2-mm-3D_ngtdm_Coarseness_32_binCount | 0.8254551 | 0.733891915 | 0.887523144 |
| log-sigma-2-mm-3D_ngtdm_Complexity_32_binCount | 0.5447322 | 0.357694069 | 0.689664722 |
| log-sigma-2-mm-3D_ngtdm_Contrast_32_binCount | 0.697991 | 0.555714179 | 0.800468215 |
| log-sigma-2-mm-3D_ngtdm_Strength_32_binCount | 0.7444635 | 0.61920187 | 0.83271515 |
| log-sigma-3-mm-3D_firstorder_10Percentile_32_binCount | 0.8953039 | 0.837466999 | 0.933344719 |
| log-sigma-3-mm-3D_firstorder_90Percentile_32_binCount | 0.9215016 | 0.876980145 | 0.950330399 |
| log-sigma-3-mm-3D_firstorder_Energy_32_binCount | 0.8005886 | 0.698907163 | 0.8706478 |
| log-sigma-3-mm-3D_firstorder_Entropy_32_binCount | 0.6617627 | 0.507284231 | 0.774932004 |
| log-sigma-3-mm-3D_firstorder_InterquartileRange_32_binCount | 0.5260416 | 0.334681684 | 0.675656281 |
| log-sigma-3-mm-3D_firstorder_Kurtosis_32_binCount | 0.5352695 | 0.346204917 | 0.682514493 |
| log-sigma-3-mm-3D_firstorder_Maximum_32_binCount | 0.8131407 | 0.716221916 | 0.879269435 |
| log-sigma-3-mm-3D_firstorder_MeanAbsoluteDeviation_32_binCount | 0.7062598 | 0.567240343 | 0.806137718 |
| log-sigma-3-mm-3D_firstorder_Mean_32_binCount | 0.9543686 | 0.927945942 | 0.971267141 |
| log-sigma-3-mm-3D_firstorder_Median_32_binCount | 0.9442638 | 0.912207748 | 0.964848596 |
| log-sigma-3-mm-3D_firstorder_Minimum_32_binCount | 0.827835 | 0.737428693 | 0.889083163 |
| log-sigma-3-mm-3D_firstorder_Range_32_binCount | 0.8652775 | 0.792279429 | 0.913838554 |
| log-sigma-3-mm-3D_firstorder_RobustMeanAbsoluteDeviation_32_binCount | 0.5456323 | 0.358925658 | 0.69029276 |
| log-sigma-3-mm-3D_firstorder_RootMeanSquared_32_binCount | 0.8953354 | 0.837595868 | 0.933343275 |
| log-sigma-3-mm-3D_firstorder_Skewness_32_binCount | 0.4696914 | 0.266501053 | 0.632819953 |
| log-sigma-3-mm-3D_firstorder_TotalEnergy_32_binCount | 0.8005886 | 0.698907163 | 0.8706478 |
| log-sigma-3-mm-3D_firstorder_Uniformity_32_binCount | 0.6706793 | 0.519210449 | 0.781219658 |
| log-sigma-3-mm-3D_firstorder_Variance_32_binCount | 0.822805 | 0.730743703 | 0.885559473 |
| log-sigma-3-mm-3D_glcm_Autocorrelation_32_binCount | 0.4084222 | 0.19337254 | 0.585605511 |
| log-sigma-3-mm-3D_glcm_ClusterProminence_32_binCount | 0.4097907 | 0.194904884 | 0.586705179 |
| log-sigma-3-mm-3D_glcm_ClusterShade_32_binCount | 0.374435 | 0.157279487 | 0.557365055 |
| log-sigma-3-mm-3D_glcm_ClusterTendency_32_binCount | 0.512304 | 0.317250522 | 0.665544716 |
| log-sigma-3-mm-3D_glcm_Contrast_32_binCount | 0.7080874 | 0.569479382 | 0.807489187 |
| log-sigma-3-mm-3D_glcm_Correlation_32_binCount | 0.4823184 | 0.281023793 | 0.6427359 |
| log-sigma-3-mm-3D_glcm_DifferenceAverage_32_binCount | 0.7383099 | 0.610916512 | 0.828412204 |
| log-sigma-3-mm-3D_glcm_DifferenceEntropy_32_binCount | 0.7405943 | 0.61394295 | 0.830025145 |
| log-sigma-3-mm-3D_glcm_DifferenceVariance_32_binCount | 0.7173224 | 0.582018157 | 0.813924644 |
| log-sigma-3-mm-3D_glcm_Id_32_binCount | 0.7683165 | 0.65269748 | 0.848958789 |
| log-sigma-3-mm-3D_glcm_Idm_32_binCount | 0.7644338 | 0.64730627 | 0.846297269 |
| log-sigma-3-mm-3D_glcm_Idmn_32_binCount | 0.7079565 | 0.569281359 | 0.807404459 |
| log-sigma-3-mm-3D_glcm_Idn_32_binCount | 0.7412363 | 0.614969361 | 0.830423967 |
| log-sigma-3-mm-3D_glcm_Imc1_32_binCount | 0.6282491 | 0.463872013 | 0.750790904 |
| log-sigma-3-mm-3D_glcm_Imc2_32_binCount | 0.3879778 | 0.171541922 | 0.56869284 |
| log-sigma-3-mm-3D_glcm_InverseVariance_32_binCount | 0.5261153 | 0.335167091 | 0.675561409 |
| log-sigma-3-mm-3D_glcm_JointAverage_32_binCount | 0.3868953 | 0.168441364 | 0.568636363 |
| log-sigma-3-mm-3D_glcm_JointEnergy_32_binCount | 0.6173267 | 0.450156697 | 0.742759188 |
| log-sigma-3-mm-3D_glcm_JointEntropy_32_binCount | 0.6858791 | 0.539481832 | 0.791950276 |
| log-sigma-3-mm-3D_glcm_MCC_32_binCount | 0.4002692 | 0.183868185 | 0.579209262 |
| log-sigma-3-mm-3D_glcm_MaximumProbability_32_binCount | 0.678853 | 0.530191213 | 0.786965112 |
| log-sigma-3-mm-3D_glcm_SumAverage_32_binCount | 0.3868953 | 0.168441364 | 0.568636363 |
| log-sigma-3-mm-3D_glcm_SumEntropy_32_binCount | 0.6388608 | 0.47706401 | 0.758630424 |
| log-sigma-3-mm-3D_glcm_SumSquares_32_binCount | 0.5289241 | 0.33766909 | 0.678030517 |
| log-sigma-3-mm-3D_glrlm_GrayLevelNonUniformity_32_binCount | 0.9624908 | 0.940374258 | 0.976481199 |
| log-sigma-3-mm-3D_glrlm_GrayLevelNonUniformityNormalized_32_binCount | 0.6797124 | 0.531029703 | 0.787672625 |
| log-sigma-3-mm-3D_glrlm_GrayLevelVariance_32_binCount | 0.5630351 | 0.380357026 | 0.703312974 |
| log-sigma-3-mm-3D_glrlm_HighGrayLevelRunEmphasis_32_binCount | 0.4043456 | 0.188694114 | 0.582376596 |
| log-sigma-3-mm-3D_glrlm_LongRunEmphasis_32_binCount | 0.7525328 | 0.630986233 | 0.838072917 |
| log-sigma-3-mm-3D_glrlm_LongRunHighGrayLevelEmphasis_32_binCount | 0.6537728 | 0.49688984 | 0.769197397 |
| log-sigma-3-mm-3D_glrlm_LongRunLowGrayLevelEmphasis_32_binCount | 0.3876088 | 0.169396968 | 0.569143757 |
| log-sigma-3-mm-3D_glrlm_LowGrayLevelRunEmphasis_32_binCount | 0.4578796 | 0.251614812 | 0.624055346 |
| log-sigma-3-mm-3D_glrlm_RunEntropy_32_binCount | 0.2159325 | -0.01808767 | 0.427123213 |
| log-sigma-3-mm-3D_glrlm_RunLengthNonUniformity_32_binCount | 0.7148341 | 0.579741294 | 0.811839726 |
| log-sigma-3-mm-3D_glrlm_RunLengthNonUniformityNormalized_32_binCount | 0.8135522 | 0.716757181 | 0.879561246 |
| log-sigma-3-mm-3D_glrlm_RunPercentage_32_binCount | 0.8304435 | 0.741132493 | 0.890841991 |
| log-sigma-3-mm-3D_glrlm_RunVariance_32_binCount | 0.7501838 | 0.627916934 | 0.836402062 |
| log-sigma-3-mm-3D_glrlm_ShortRunEmphasis_32_binCount | 0.8079695 | 0.708679695 | 0.875837729 |
| log-sigma-3-mm-3D_glrlm_ShortRunHighGrayLevelEmphasis_32_binCount | 0.3438399 | 0.119696817 | 0.534094036 |
| log-sigma-3-mm-3D_glrlm_ShortRunLowGrayLevelEmphasis_32_binCount | 0.4725306 | 0.269146078 | 0.63529855 |
| log-sigma-3-mm-3D_glszm_GrayLevelNonUniformity_32_binCount | 0.6761554 | 0.526458846 | 0.78510476 |
| log-sigma-3-mm-3D_glszm_GrayLevelNonUniformityNormalized_32_binCount | 0.4308214 | 0.219946185 | 0.602960443 |
| log-sigma-3-mm-3D_glszm_GrayLevelVariance_32_binCount | 0.4687958 | 0.265194274 | 0.632226948 |
| log-sigma-3-mm-3D_glszm_HighGrayLevelZoneEmphasis_32_binCount | 0.3264081 | 0.100507091 | 0.519815223 |
| log-sigma-3-mm-3D_glszm_LargeAreaEmphasis_32_binCount | 0.8292159 | 0.740027625 | 0.889832111 |
| log-sigma-3-mm-3D_glszm_LargeAreaHighGrayLevelEmphasis_32_binCount | 0.7876807 | 0.680587458 | 0.861926717 |
| log-sigma-3-mm-3D_glszm_LargeAreaLowGrayLevelEmphasis_32_binCount | 0.877181 | 0.810284853 | 0.921547526 |
| log-sigma-3-mm-3D_glszm_LowGrayLevelZoneEmphasis_32_binCount | 0.4727482 | 0.269640464 | 0.635372139 |
| log-sigma-3-mm-3D_glszm_SizeZoneNonUniformity_32_binCount | 0.262475 | 0.030069372 | 0.467214242 |
| log-sigma-3-mm-3D_glszm_SizeZoneNonUniformityNormalized_32_binCount | 0.5098559 | 0.313995034 | 0.663799467 |
| log-sigma-3-mm-3D_glszm_SmallAreaEmphasis_32_binCount | 0.466733 | 0.261914585 | 0.630968806 |
| log-sigma-3-mm-3D_glszm_SmallAreaHighGrayLevelEmphasis_32_binCount | 0.4416284 | 0.232905605 | 0.611265303 |
| log-sigma-3-mm-3D_glszm_SmallAreaLowGrayLevelEmphasis_32_binCount | 0.4328332 | 0.221800433 | 0.60473761 |
| log-sigma-3-mm-3D_glszm_ZoneEntropy_32_binCount | 0.754488 | 0.633620718 | 0.839438797 |
| log-sigma-3-mm-3D_glszm_ZonePercentage_32_binCount | 0.7703256 | 0.655336288 | 0.85038126 |
| log-sigma-3-mm-3D_glszm_ZoneVariance_32_binCount | 0.828052 | 0.738341537 | 0.889056714 |
| log-sigma-3-mm-3D_gldm_DependenceEntropy_32_binCount | 0.6553635 | 0.499344586 | 0.770209938 |
| log-sigma-3-mm-3D_gldm_DependenceNonUniformity_32_binCount | 0.8566696 | 0.780001446 | 0.908057737 |
| log-sigma-3-mm-3D_gldm_DependenceNonUniformityNormalized_32_binCount | 0.7788553 | 0.667852991 | 0.856024166 |
| log-sigma-3-mm-3D_gldm_DependenceVariance_32_binCount | 0.8414579 | 0.757240236 | 0.898133981 |
| log-sigma-3-mm-3D_gldm_GrayLevelNonUniformity_32_binCount | 0.8900652 | 0.829442477 | 0.929979967 |
| log-sigma-3-mm-3D_gldm_GrayLevelVariance_32_binCount | 0.5764801 | 0.397343801 | 0.71320158 |
| log-sigma-3-mm-3D_gldm_HighGrayLevelEmphasis_32_binCount | 0.4041413 | 0.188449398 | 0.582219127 |
| log-sigma-3-mm-3D_gldm_LargeDependenceEmphasis_32_binCount | 0.8207686 | 0.727159967 | 0.884384681 |
| log-sigma-3-mm-3D_gldm_LargeDependenceHighGrayLevelEmphasis_32_binCount | 0.7151153 | 0.578816584 | 0.812451841 |
| log-sigma-3-mm-3D_gldm_LargeDependenceLowGrayLevelEmphasis_32_binCount | 0.5060326 | 0.309627007 | 0.660797692 |
| log-sigma-3-mm-3D_gldm_LowGrayLevelEmphasis_32_binCount | 0.4643116 | 0.259266284 | 0.629011088 |
| log-sigma-3-mm-3D_gldm_SmallDependenceEmphasis_32_binCount | 0.7676473 | 0.651481214 | 0.848586652 |
| log-sigma-3-mm-3D_gldm_SmallDependenceHighGrayLevelEmphasis_32_binCount | 0.8162333 | 0.720561164 | 0.881370936 |
| log-sigma-3-mm-3D_gldm_SmallDependenceLowGrayLevelEmphasis_32_binCount | 0.5764142 | 0.396902461 | 0.713282588 |
| log-sigma-3-mm-3D_ngtdm_Busyness_32_binCount | 0.9445625 | 0.912727489 | 0.965024397 |
| log-sigma-3-mm-3D_ngtdm_Coarseness_32_binCount | 0.7733784 | 0.659527397 | 0.852487493 |
| log-sigma-3-mm-3D_ngtdm_Complexity_32_binCount | 0.6592667 | 0.504031367 | 0.773142861 |
| log-sigma-3-mm-3D_ngtdm_Contrast_32_binCount | 0.700405 | 0.558960341 | 0.802161686 |
| log-sigma-3-mm-3D_ngtdm_Strength_32_binCount | 0.7025215 | 0.561716434 | 0.803675014 |
| log-sigma-4-mm-3D_firstorder_10Percentile_32_binCount | 0.8992644 | 0.8433399 | 0.935940072 |
| log-sigma-4-mm-3D_firstorder_90Percentile_32_binCount | 0.936661 | 0.900301205 | 0.960035713 |
| log-sigma-4-mm-3D_firstorder_Energy_32_binCount | 0.8306318 | 0.74202649 | 0.890790108 |
| log-sigma-4-mm-3D_firstorder_Entropy_32_binCount | 0.6606601 | 0.505694658 | 0.774192596 |
| log-sigma-4-mm-3D_firstorder_InterquartileRange_32_binCount | 0.5503383 | 0.363977927 | 0.694091033 |
| log-sigma-4-mm-3D_firstorder_Kurtosis_32_binCount | 0.5236832 | 0.331506808 | 0.673990175 |
| log-sigma-4-mm-3D_firstorder_Maximum_32_binCount | 0.8519409 | 0.772577805 | 0.905070002 |
| log-sigma-4-mm-3D_firstorder_MeanAbsoluteDeviation_32_binCount | 0.7406493 | 0.614005716 | 0.830067055 |
| log-sigma-4-mm-3D_firstorder_Mean_32_binCount | 0.9481793 | 0.918315227 | 0.967333441 |
| log-sigma-4-mm-3D_firstorder_Median_32_binCount | 0.9436666 | 0.91134181 | 0.964452877 |
| log-sigma-4-mm-3D_firstorder_Minimum_32_binCount | 0.8613226 | 0.786410174 | 0.911245987 |
| log-sigma-4-mm-3D_firstorder_Range_32_binCount | 0.8871966 | 0.824970913 | 0.928158316 |
| log-sigma-4-mm-3D_firstorder_RobustMeanAbsoluteDeviation_32_binCount | 0.5836992 | 0.40604338 | 0.718658203 |
| log-sigma-4-mm-3D_firstorder_RootMeanSquared_32_binCount | 0.9028017 | 0.848800169 | 0.938200528 |
| log-sigma-4-mm-3D_firstorder_Skewness_32_binCount | 0.5026038 | 0.305409521 | 0.658220281 |
| log-sigma-4-mm-3D_firstorder_TotalEnergy_32_binCount | 0.8306318 | 0.74202649 | 0.890790108 |
| log-sigma-4-mm-3D_firstorder_Uniformity_32_binCount | 0.6312398 | 0.4672952 | 0.753102659 |
| log-sigma-4-mm-3D_firstorder_Variance_32_binCount | 0.8425647 | 0.759405343 | 0.898712669 |
| log-sigma-4-mm-3D_glcm_Autocorrelation_32_binCount | 0.5115919 | 0.31625863 | 0.665054331 |
| log-sigma-4-mm-3D_glcm_ClusterProminence_32_binCount | 0.6400634 | 0.479921636 | 0.759053172 |
| log-sigma-4-mm-3D_glcm_ClusterShade_32_binCount | 0.5905436 | 0.415336268 | 0.723454318 |
| log-sigma-4-mm-3D_glcm_ClusterTendency_32_binCount | 0.628709 | 0.463905075 | 0.751316386 |
| log-sigma-4-mm-3D_glcm_Contrast_32_binCount | 0.7544956 | 0.633111148 | 0.839603098 |
| log-sigma-4-mm-3D_glcm_Correlation_32_binCount | 0.6217024 | 0.455109953 | 0.746165859 |
| log-sigma-4-mm-3D_glcm_DifferenceAverage_32_binCount | 0.8023577 | 0.700633654 | 0.872072709 |
| log-sigma-4-mm-3D_glcm_DifferenceEntropy_32_binCount | 0.8031047 | 0.701772431 | 0.872554202 |
| log-sigma-4-mm-3D_glcm_DifferenceVariance_32_binCount | 0.7237586 | 0.590770205 | 0.818403632 |
| log-sigma-4-mm-3D_glcm_Id_32_binCount | 0.8067589 | 0.707015747 | 0.875004731 |
| log-sigma-4-mm-3D_glcm_Idm_32_binCount | 0.8053289 | 0.704987071 | 0.874039042 |
| log-sigma-4-mm-3D_glcm_Idmn_32_binCount | 0.759565 | 0.640184901 | 0.843068579 |
| log-sigma-4-mm-3D_glcm_Idn_32_binCount | 0.8051842 | 0.704681845 | 0.873970336 |
| log-sigma-4-mm-3D_glcm_Imc1_32_binCount | 0.7131535 | 0.576529618 | 0.810965289 |
| log-sigma-4-mm-3D_glcm_Imc2_32_binCount | 0.5561789 | 0.372314123 | 0.698038037 |
| log-sigma-4-mm-3D_glcm_InverseVariance_32_binCount | 0.6533835 | 0.496113839 | 0.769008414 |
| log-sigma-4-mm-3D_glcm_JointAverage_32_binCount | 0.494821 | 0.295858885 | 0.652358915 |
| log-sigma-4-mm-3D_glcm_JointEnergy_32_binCount | 0.5449993 | 0.358392514 | 0.689726794 |
| log-sigma-4-mm-3D_glcm_JointEntropy_32_binCount | 0.7014147 | 0.560213077 | 0.802903554 |
| log-sigma-4-mm-3D_glcm_MCC_32_binCount | 0.5568903 | 0.372315266 | 0.698892786 |
| log-sigma-4-mm-3D_glcm_MaximumProbability_32_binCount | 0.5323394 | 0.342246082 | 0.680450489 |
| log-sigma-4-mm-3D_glcm_SumAverage_32_binCount | 0.494821 | 0.295858885 | 0.652358915 |
| log-sigma-4-mm-3D_glcm_SumEntropy_32_binCount | 0.6282019 | 0.463221591 | 0.750959899 |
| log-sigma-4-mm-3D_glcm_SumSquares_32_binCount | 0.6373256 | 0.475135338 | 0.757503778 |
| log-sigma-4-mm-3D_glrlm_GrayLevelNonUniformity_32_binCount | 0.957662 | 0.932356506 | 0.973537878 |
| log-sigma-4-mm-3D_glrlm_GrayLevelNonUniformityNormalized_32_binCount | 0.6770223 | 0.527437311 | 0.785775176 |
| log-sigma-4-mm-3D_glrlm_GrayLevelVariance_32_binCount | 0.6515993 | 0.493779531 | 0.767731843 |
| log-sigma-4-mm-3D_glrlm_HighGrayLevelRunEmphasis_32_binCount | 0.4816917 | 0.279964551 | 0.642377765 |
| log-sigma-4-mm-3D_glrlm_LongRunEmphasis_32_binCount | 0.7243908 | 0.592177065 | 0.818671155 |
| log-sigma-4-mm-3D_glrlm_LongRunHighGrayLevelEmphasis_32_binCount | 0.5878994 | 0.411956414 | 0.721526255 |
| log-sigma-4-mm-3D_glrlm_LongRunLowGrayLevelEmphasis_32_binCount | 0.388952 | 0.171880863 | 0.569803685 |
| log-sigma-4-mm-3D_glrlm_LowGrayLevelRunEmphasis_32_binCount | 0.4632862 | 0.25817396 | 0.628170029 |
| log-sigma-4-mm-3D_glrlm_RunEntropy_32_binCount | 0.3667877 | 0.145605196 | 0.552556684 |
| log-sigma-4-mm-3D_glrlm_RunLengthNonUniformity_32_binCount | 0.7385104 | 0.612080624 | 0.828273594 |
| log-sigma-4-mm-3D_glrlm_RunLengthNonUniformityNormalized_32_binCount | 0.8320213 | 0.743439319 | 0.891886835 |
| log-sigma-4-mm-3D_glrlm_RunPercentage_32_binCount | 0.8460096 | 0.763860721 | 0.901156717 |
| log-sigma-4-mm-3D_glrlm_RunVariance_32_binCount | 0.7183544 | 0.584073002 | 0.814436455 |
| log-sigma-4-mm-3D_glrlm_ShortRunEmphasis_32_binCount | 0.8216524 | 0.728386234 | 0.884988921 |
| log-sigma-4-mm-3D_glrlm_ShortRunHighGrayLevelEmphasis_32_binCount | 0.4659034 | 0.261173953 | 0.630231532 |
| log-sigma-4-mm-3D_glrlm_ShortRunLowGrayLevelEmphasis_32_binCount | 0.4792004 | 0.277141095 | 0.640406392 |
| log-sigma-4-mm-3D_glszm_GrayLevelNonUniformity_32_binCount | 0.7020484 | 0.561218491 | 0.803298864 |
| log-sigma-4-mm-3D_glszm_GrayLevelNonUniformityNormalized_32_binCount | 0.5281983 | 0.337063591 | 0.677377387 |
| log-sigma-4-mm-3D_glszm_GrayLevelVariance_32_binCount | 0.5725156 | 0.392057361 | 0.710387429 |
| log-sigma-4-mm-3D_glszm_HighGrayLevelZoneEmphasis_32_binCount | 0.389285 | 0.171219685 | 0.570517316 |
| log-sigma-4-mm-3D_glszm_LargeAreaEmphasis_32_binCount | 0.8663245 | 0.794334859 | 0.914386701 |
| log-sigma-4-mm-3D_glszm_LargeAreaHighGrayLevelEmphasis_32_binCount | 0.7592418 | 0.640688279 | 0.84255644 |
| log-sigma-4-mm-3D_glszm_LargeAreaLowGrayLevelEmphasis_32_binCount | 0.9565744 | 0.931345058 | 0.972677304 |
| log-sigma-4-mm-3D_glszm_LowGrayLevelZoneEmphasis_32_binCount | 0.2656725 | 0.033532136 | 0.46988275 |
| log-sigma-4-mm-3D_glszm_SizeZoneNonUniformity_32_binCount | 0.3877447 | 0.169899058 | 0.56910253 |
| log-sigma-4-mm-3D_glszm_SizeZoneNonUniformityNormalized_32_binCount | 0.4788422 | 0.279124154 | 0.639170991 |
| log-sigma-4-mm-3D_glszm_SmallAreaEmphasis_32_binCount | 0.4566066 | 0.252930395 | 0.621922217 |
| log-sigma-4-mm-3D_glszm_SmallAreaHighGrayLevelEmphasis_32_binCount | 0.4986235 | 0.301409017 | 0.654878926 |
| log-sigma-4-mm-3D_glszm_SmallAreaLowGrayLevelEmphasis_32_binCount | 0.1524691 | -0.08432604 | 0.372307688 |
| log-sigma-4-mm-3D_glszm_ZoneEntropy_32_binCount | 0.7387038 | 0.612293796 | 0.828422981 |
| log-sigma-4-mm-3D_glszm_ZonePercentage_32_binCount | 0.820541 | 0.726965582 | 0.884194176 |
| log-sigma-4-mm-3D_glszm_ZoneVariance_32_binCount | 0.8645269 | 0.791678136 | 0.913205093 |
| log-sigma-4-mm-3D_gldm_DependenceEntropy_32_binCount | 0.7358157 | 0.607468124 | 0.826695644 |
| log-sigma-4-mm-3D_gldm_DependenceNonUniformity_32_binCount | 0.8885293 | 0.827383514 | 0.928914285 |
| log-sigma-4-mm-3D_gldm_DependenceNonUniformityNormalized_32_binCount | 0.7977287 | 0.69402717 | 0.868957644 |
| log-sigma-4-mm-3D_gldm_DependenceVariance_32_binCount | 0.8371803 | 0.750945274 | 0.895313351 |
| log-sigma-4-mm-3D_gldm_GrayLevelNonUniformity_32_binCount | 0.9075026 | 0.855733483 | 0.941290431 |
| log-sigma-4-mm-3D_gldm_GrayLevelVariance_32_binCount | 0.6634263 | 0.509375926 | 0.776149724 |
| log-sigma-4-mm-3D_gldm_HighGrayLevelEmphasis_32_binCount | 0.4883655 | 0.287997176 | 0.647470991 |
| log-sigma-4-mm-3D_gldm_LargeDependenceEmphasis_32_binCount | 0.8226053 | 0.729822341 | 0.885607929 |
| log-sigma-4-mm-3D_gldm_LargeDependenceHighGrayLevelEmphasis_32_binCount | 0.6942404 | 0.550654849 | 0.797841848 |
| log-sigma-4-mm-3D_gldm_LargeDependenceLowGrayLevelEmphasis_32_binCount | 0.4732703 | 0.270623405 | 0.635629781 |
| log-sigma-4-mm-3D_gldm_LowGrayLevelEmphasis_32_binCount | 0.4706987 | 0.267165749 | 0.633809044 |
| log-sigma-4-mm-3D_gldm_SmallDependenceEmphasis_32_binCount | 0.8165011 | 0.720948307 | 0.881549619 |
| log-sigma-4-mm-3D_gldm_SmallDependenceHighGrayLevelEmphasis_32_binCount | 0.905432 | 0.85260231 | 0.939950079 |
| log-sigma-4-mm-3D_gldm_SmallDependenceLowGrayLevelEmphasis_32_binCount | 0.5674469 | 0.3858479 | 0.706589125 |
| log-sigma-4-mm-3D_ngtdm_Busyness_32_binCount | 0.9241184 | 0.88122406 | 0.951949195 |
| log-sigma-4-mm-3D_ngtdm_Coarseness_32_binCount | 0.7466488 | 0.622183281 | 0.834230785 |
| log-sigma-4-mm-3D_ngtdm_Complexity_32_binCount | 0.7863103 | 0.678311842 | 0.861098128 |
| log-sigma-4-mm-3D_ngtdm_Contrast_32_binCount | 0.7156382 | 0.57977647 | 0.812736977 |
| log-sigma-4-mm-3D_ngtdm_Strength_32_binCount | 0.7972727 | 0.693550617 | 0.86859971 |
| log-sigma-5-mm-3D_firstorder_10Percentile_32_binCount | 0.8723252 | 0.802848935 | 0.918427806 |
| log-sigma-5-mm-3D_firstorder_90Percentile_32_binCount | 0.9398214 | 0.905185146 | 0.962053084 |
| log-sigma-5-mm-3D_firstorder_Energy_32_binCount | 0.8234747 | 0.731733754 | 0.885999831 |
| log-sigma-5-mm-3D_firstorder_Entropy_32_binCount | 0.6341714 | 0.470914059 | 0.755276993 |
| log-sigma-5-mm-3D_firstorder_InterquartileRange_32_binCount | 0.6107849 | 0.440586772 | 0.738419774 |
| log-sigma-5-mm-3D_firstorder_Kurtosis_32_binCount | 0.5293554 | 0.337927289 | 0.67845678 |
| log-sigma-5-mm-3D_firstorder_Maximum_32_binCount | 0.8964666 | 0.838919771 | 0.934179108 |
| log-sigma-5-mm-3D_firstorder_MeanAbsoluteDeviation_32_binCount | 0.7646887 | 0.647343061 | 0.846567914 |
| log-sigma-5-mm-3D_firstorder_Mean_32_binCount | 0.9354707 | 0.898556075 | 0.959251712 |
| log-sigma-5-mm-3D_firstorder_Median_32_binCount | 0.9340325 | 0.896395382 | 0.958317616 |
| log-sigma-5-mm-3D_firstorder_Minimum_32_binCount | 0.8615622 | 0.786767316 | 0.911402665 |
| log-sigma-5-mm-3D_firstorder_Range_32_binCount | 0.8960584 | 0.838308223 | 0.93391334 |
| log-sigma-5-mm-3D_firstorder_RobustMeanAbsoluteDeviation_32_binCount | 0.6484383 | 0.489573796 | 0.765493604 |
| log-sigma-5-mm-3D_firstorder_RootMeanSquared_32_binCount | 0.8972755 | 0.840430636 | 0.934625979 |
| log-sigma-5-mm-3D_firstorder_Skewness_32_binCount | 0.5752487 | 0.395285755 | 0.712478013 |
| log-sigma-5-mm-3D_firstorder_TotalEnergy_32_binCount | 0.8234747 | 0.731733754 | 0.885999831 |
| log-sigma-5-mm-3D_firstorder_Uniformity_32_binCount | 0.6086873 | 0.438093293 | 0.736827289 |
| log-sigma-5-mm-3D_firstorder_Variance_32_binCount | 0.818127 | 0.723917978 | 0.882456031 |
| log-sigma-5-mm-3D_glcm_Autocorrelation_32_binCount | 0.5422294 | 0.35396865 | 0.688030694 |
| log-sigma-5-mm-3D_glcm_ClusterProminence_32_binCount | 0.6111525 | 0.441157626 | 0.738652066 |
| log-sigma-5-mm-3D_glcm_ClusterShade_32_binCount | 0.562811 | 0.380370136 | 0.703039409 |
| log-sigma-5-mm-3D_glcm_ClusterTendency_32_binCount | 0.6244459 | 0.458269872 | 0.748281038 |
| log-sigma-5-mm-3D_glcm_Contrast_32_binCount | 0.8456402 | 0.763333198 | 0.900908737 |
| log-sigma-5-mm-3D_glcm_Correlation_32_binCount | 0.7363382 | 0.608040591 | 0.827101784 |
| log-sigma-5-mm-3D_glcm_DifferenceAverage_32_binCount | 0.8383982 | 0.752757397 | 0.896110881 |
| log-sigma-5-mm-3D_glcm_DifferenceEntropy_32_binCount | 0.8271241 | 0.736609234 | 0.888549625 |
| log-sigma-5-mm-3D_glcm_DifferenceVariance_32_binCount | 0.8386309 | 0.753314024 | 0.896203893 |
| log-sigma-5-mm-3D_glcm_Id_32_binCount | 0.8176237 | 0.722601916 | 0.882289786 |
| log-sigma-5-mm-3D_glcm_Idm_32_binCount | 0.8178109 | 0.722883264 | 0.882411627 |
| log-sigma-5-mm-3D_glcm_Idmn_32_binCount | 0.8457707 | 0.763524474 | 0.900994983 |
| log-sigma-5-mm-3D_glcm_Idn_32_binCount | 0.8365851 | 0.750114247 | 0.894908185 |
| log-sigma-5-mm-3D_glcm_Imc1_32_binCount | 0.7899884 | 0.683215359 | 0.863678253 |
| log-sigma-5-mm-3D_glcm_Imc2_32_binCount | 0.6579784 | 0.502194376 | 0.772272094 |
| log-sigma-5-mm-3D_glcm_InverseVariance_32_binCount | 0.7013245 | 0.560387844 | 0.802745244 |
| log-sigma-5-mm-3D_glcm_JointAverage_32_binCount | 0.5576366 | 0.373150629 | 0.699481583 |
| log-sigma-5-mm-3D_glcm_JointEnergy_32_binCount | 0.5437065 | 0.356695954 | 0.688797665 |
| log-sigma-5-mm-3D_glcm_JointEntropy_32_binCount | 0.681088 | 0.53296785 | 0.788609648 |
| log-sigma-5-mm-3D_glcm_MCC_32_binCount | 0.6837405 | 0.536661074 | 0.790430867 |
| log-sigma-5-mm-3D_glcm_MaximumProbability_32_binCount | 0.5391801 | 0.351446652 | 0.68528471 |
| log-sigma-5-mm-3D_glcm_SumAverage_32_binCount | 0.5576366 | 0.373150629 | 0.699481583 |
| log-sigma-5-mm-3D_glcm_SumEntropy_32_binCount | 0.5995664 | 0.426256441 | 0.730247805 |
| log-sigma-5-mm-3D_glcm_SumSquares_32_binCount | 0.6301652 | 0.46569909 | 0.752397963 |
| log-sigma-5-mm-3D_glrlm_GrayLevelNonUniformity_32_binCount | 0.963295 | 0.940874232 | 0.977175742 |
| log-sigma-5-mm-3D_glrlm_GrayLevelNonUniformityNormalized_32_binCount | 0.6494245 | 0.490887197 | 0.766191549 |
| log-sigma-5-mm-3D_glrlm_GrayLevelVariance_32_binCount | 0.6098583 | 0.439500588 | 0.737710997 |
| log-sigma-5-mm-3D_glrlm_HighGrayLevelRunEmphasis_32_binCount | 0.4963569 | 0.297610567 | 0.653567705 |
| log-sigma-5-mm-3D_glrlm_LongRunEmphasis_32_binCount | 0.7329166 | 0.603595125 | 0.82465783 |
| log-sigma-5-mm-3D_glrlm_LongRunHighGrayLevelEmphasis_32_binCount | 0.5803953 | 0.402129456 | 0.716137252 |
| log-sigma-5-mm-3D_glrlm_LongRunLowGrayLevelEmphasis_32_binCount | 0.3685171 | 0.148228246 | 0.553651886 |
| log-sigma-5-mm-3D_glrlm_LowGrayLevelRunEmphasis_32_binCount | 0.497379 | 0.298891189 | 0.654327216 |
| log-sigma-5-mm-3D_glrlm_RunEntropy_32_binCount | 0.5269219 | 0.335490612 | 0.676420756 |
| log-sigma-5-mm-3D_glrlm_RunLengthNonUniformity_32_binCount | 0.7359282 | 0.608528917 | 0.826490046 |
| log-sigma-5-mm-3D_glrlm_RunLengthNonUniformityNormalized_32_binCount | 0.8267175 | 0.735807505 | 0.888339435 |
| log-sigma-5-mm-3D_glrlm_RunPercentage_32_binCount | 0.8328018 | 0.74471653 | 0.892365134 |
| log-sigma-5-mm-3D_glrlm_RunVariance_32_binCount | 0.7385792 | 0.61140213 | 0.828562382 |
| log-sigma-5-mm-3D_glrlm_ShortRunEmphasis_32_binCount | 0.8167339 | 0.721366405 | 0.881681498 |
| log-sigma-5-mm-3D_glrlm_ShortRunHighGrayLevelEmphasis_32_binCount | 0.4775149 | 0.274991186 | 0.639167182 |
| log-sigma-5-mm-3D_glrlm_ShortRunLowGrayLevelEmphasis_32_binCount | 0.5232478 | 0.330414584 | 0.673874817 |
| log-sigma-5-mm-3D_glszm_GrayLevelNonUniformity_32_binCount | 0.7711314 | 0.656419005 | 0.850944338 |
| log-sigma-5-mm-3D_glszm_GrayLevelNonUniformityNormalized_32_binCount | 0.5892855 | 0.414064178 | 0.722416559 |
| log-sigma-5-mm-3D_glszm_GrayLevelVariance_32_binCount | 0.5562284 | 0.371313249 | 0.698466587 |
| log-sigma-5-mm-3D_glszm_HighGrayLevelZoneEmphasis_32_binCount | 0.4460335 | 0.237632708 | 0.614875444 |
| log-sigma-5-mm-3D_glszm_LargeAreaEmphasis_32_binCount | 0.8877479 | 0.826060525 | 0.928446452 |
| log-sigma-5-mm-3D_glszm_LargeAreaHighGrayLevelEmphasis_32_binCount | 0.7961912 | 0.692477447 | 0.867733333 |
| log-sigma-5-mm-3D_glszm_LargeAreaLowGrayLevelEmphasis_32_binCount | 0.9212506 | 0.876677076 | 0.950148069 |
| log-sigma-5-mm-3D_glszm_LowGrayLevelZoneEmphasis_32_binCount | 0.2663174 | 0.034262006 | 0.470405695 |
| log-sigma-5-mm-3D_glszm_SizeZoneNonUniformity_32_binCount | 0.5228505 | 0.331010788 | 0.673163882 |
| log-sigma-5-mm-3D_glszm_SizeZoneNonUniformityNormalized_32_binCount | 0.5182028 | 0.327293137 | 0.66890216 |
| log-sigma-5-mm-3D_glszm_SmallAreaEmphasis_32_binCount | 0.5057881 | 0.310944047 | 0.659986719 |
| log-sigma-5-mm-3D_glszm_SmallAreaHighGrayLevelEmphasis_32_binCount | 0.5692848 | 0.388019422 | 0.707995802 |
| log-sigma-5-mm-3D_glszm_SmallAreaLowGrayLevelEmphasis_32_binCount | 0.2230945 | -0.01195811 | 0.43395532 |
| log-sigma-5-mm-3D_glszm_ZoneEntropy_32_binCount | 0.6928752 | 0.550170363 | 0.796442832 |
| log-sigma-5-mm-3D_glszm_ZonePercentage_32_binCount | 0.8067438 | 0.706981776 | 0.874998223 |
| log-sigma-5-mm-3D_glszm_ZoneVariance_32_binCount | 0.8829034 | 0.818804137 | 0.925290305 |
| log-sigma-5-mm-3D_gldm_DependenceEntropy_32_binCount | 0.7694381 | 0.653984308 | 0.849809042 |
| log-sigma-5-mm-3D_gldm_DependenceNonUniformity_32_binCount | 0.875516 | 0.807946729 | 0.920419873 |
| log-sigma-5-mm-3D_gldm_DependenceNonUniformityNormalized_32_binCount | 0.7847376 | 0.675598841 | 0.860179249 |
| log-sigma-5-mm-3D_gldm_DependenceVariance_32_binCount | 0.7647689 | 0.647921978 | 0.846481339 |
| log-sigma-5-mm-3D_gldm_GrayLevelNonUniformity_32_binCount | 0.9162628 | 0.868997313 | 0.946956089 |
| log-sigma-5-mm-3D_gldm_GrayLevelVariance_32_binCount | 0.6209754 | 0.453820808 | 0.745761706 |
| log-sigma-5-mm-3D_gldm_HighGrayLevelEmphasis_32_binCount | 0.5109596 | 0.315386546 | 0.664615528 |
| log-sigma-5-mm-3D_gldm_LargeDependenceEmphasis_32_binCount | 0.8044015 | 0.703771016 | 0.873383721 |
| log-sigma-5-mm-3D_gldm_LargeDependenceHighGrayLevelEmphasis_32_binCount | 0.6661839 | 0.513158616 | 0.778063072 |
| log-sigma-5-mm-3D_gldm_LargeDependenceLowGrayLevelEmphasis_32_binCount | 0.3525587 | 0.132076326 | 0.539980256 |
| log-sigma-5-mm-3D_gldm_LowGrayLevelEmphasis_32_binCount | 0.4872352 | 0.286867818 | 0.646517951 |
| log-sigma-5-mm-3D_gldm_SmallDependenceEmphasis_32_binCount | 0.8114881 | 0.713736091 | 0.878194599 |
| log-sigma-5-mm-3D_gldm_SmallDependenceHighGrayLevelEmphasis_32_binCount | 0.7905658 | 0.684085034 | 0.864053552 |
| log-sigma-5-mm-3D_gldm_SmallDependenceLowGrayLevelEmphasis_32_binCount | 0.6719682 | 0.520698306 | 0.782206184 |
| log-sigma-5-mm-3D_ngtdm_Busyness_32_binCount | 0.9216979 | 0.877539673 | 0.950388973 |
| log-sigma-5-mm-3D_ngtdm_Coarseness_32_binCount | 0.8258438 | 0.734454878 | 0.887782105 |
| log-sigma-5-mm-3D_ngtdm_Complexity_32_binCount | 0.7826078 | 0.670563854 | 0.859332764 |
| log-sigma-5-mm-3D_ngtdm_Contrast_32_binCount | 0.7032615 | 0.562715344 | 0.804192811 |
| log-sigma-5-mm-3D_ngtdm_Strength_32_binCount | 0.8151756 | 0.719123223 | 0.880638968 |
| wavelet-LLH_firstorder_10Percentile_32_binCount | 0.7865082 | 0.676047115 | 0.86198135 |
| wavelet-LLH_firstorder_90Percentile_32_binCount | 0.7016996 | 0.561192469 | 0.802911881 |
| wavelet-LLH_firstorder_Energy_32_binCount | 0.6700601 | 0.519874412 | 0.780285363 |
| wavelet-LLH_firstorder_Entropy_32_binCount | 0.5850073 | 0.407934841 | 0.719533527 |
| wavelet-LLH_firstorder_InterquartileRange_32_binCount | 0.6126343 | 0.445228744 | 0.73896423 |
| wavelet-LLH_firstorder_Kurtosis_32_binCount | 0.2797258 | 0.048824721 | 0.481563917 |
| wavelet-LLH_firstorder_Maximum_32_binCount | 0.5752317 | 0.397700873 | 0.711577886 |
| wavelet-LLH_firstorder_MeanAbsoluteDeviation_32_binCount | 0.6946143 | 0.552333791 | 0.797720536 |
| wavelet-LLH_firstorder_Mean_32_binCount | 0.8694498 | 0.79855873 | 0.916548974 |
| wavelet-LLH_firstorder_Median_32_binCount | 0.8707335 | 0.800612187 | 0.917349836 |
| wavelet-LLH_firstorder_Minimum_32_binCount | 0.6977514 | 0.555624857 | 0.800225107 |
| wavelet-LLH_firstorder_Range_32_binCount | 0.689701 | 0.545874613 | 0.794223932 |
| wavelet-LLH_firstorder_RobustMeanAbsoluteDeviation_32_binCount | 0.6220745 | 0.457329677 | 0.745827421 |
| wavelet-LLH_firstorder_RootMeanSquared_32_binCount | 0.6971138 | 0.555864412 | 0.799420641 |
| wavelet-LLH_firstorder_Skewness_32_binCount | 0.2734796 | 0.04201275 | 0.476381683 |
| wavelet-LLH_firstorder_TotalEnergy_32_binCount | 0.6700601 | 0.519874412 | 0.780285363 |
| wavelet-LLH_firstorder_Uniformity_32_binCount | 0.5838973 | 0.406707583 | 0.718655253 |
| wavelet-LLH_firstorder_Variance_32_binCount | 0.642664 | 0.483967759 | 0.760693893 |
| wavelet-LLH_glcm_Autocorrelation_32_binCount | 0.3956782 | 0.179699364 | 0.575098924 |
| wavelet-LLH_glcm_ClusterProminence_32_binCount | 0.5421065 | 0.356110982 | 0.687077685 |
| wavelet-LLH_glcm_ClusterShade_32_binCount | 0.7692624 | 0.653735942 | 0.849689908 |
| wavelet-LLH_glcm_ClusterTendency_32_binCount | 0.5359169 | 0.346958407 | 0.68301613 |
| wavelet-LLH_glcm_Contrast_32_binCount | 0.5564333 | 0.372135835 | 0.698409878 |
| wavelet-LLH_glcm_Correlation_32_binCount | 0.4888843 | 0.288605884 | 0.647873022 |
| wavelet-LLH_glcm_DifferenceAverage_32_binCount | 0.600236 | 0.427033264 | 0.7307634 |
| wavelet-LLH_glcm_DifferenceEntropy_32_binCount | 0.59637 | 0.422381168 | 0.727844491 |
| wavelet-LLH_glcm_DifferenceVariance_32_binCount | 0.5301324 | 0.340309458 | 0.678500155 |
| wavelet-LLH_glcm_Id_32_binCount | 0.616408 | 0.448270112 | 0.742339185 |
| wavelet-LLH_glcm_Idm_32_binCount | 0.6158751 | 0.447630112 | 0.741936964 |
| wavelet-LLH_glcm_Idmn_32_binCount | 0.5626352 | 0.379761211 | 0.703051825 |
| wavelet-LLH_glcm_Idn_32_binCount | 0.6038549 | 0.431701382 | 0.733384228 |
| wavelet-LLH_glcm_Imc1_32_binCount | 0.6009719 | 0.430352881 | 0.730451923 |
| wavelet-LLH_glcm_Imc2_32_binCount | 0.5850017 | 0.408384485 | 0.719365514 |
| wavelet-LLH_glcm_InverseVariance_32_binCount | 0.3816215 | 0.162420587 | 0.564436093 |
| wavelet-LLH_glcm_JointAverage_32_binCount | 0.360349 | 0.138880525 | 0.547140432 |
| wavelet-LLH_glcm_JointEnergy_32_binCount | 0.5578281 | 0.373675009 | 0.699518757 |
| wavelet-LLH_glcm_JointEntropy_32_binCount | 0.5801459 | 0.40177127 | 0.715969549 |
| wavelet-LLH_glcm_MCC_32_binCount | 0.2968036 | 0.068635324 | 0.495156355 |
| wavelet-LLH_glcm_MaximumProbability_32_binCount | 0.583988 | 0.406699878 | 0.718765823 |
| wavelet-LLH_glcm_SumAverage_32_binCount | 0.360349 | 0.138880525 | 0.547140432 |
| wavelet-LLH_glcm_SumEntropy_32_binCount | 0.5718623 | 0.391155995 | 0.709934676 |
| wavelet-LLH_glcm_SumSquares_32_binCount | 0.5427825 | 0.355426268 | 0.688154961 |
| wavelet-LLH_glrlm_GrayLevelNonUniformity_32_binCount | 0.9575295 | 0.930462453 | 0.973856651 |
| wavelet-LLH_glrlm_GrayLevelNonUniformityNormalized_32_binCount | 0.5525507 | 0.367590766 | 0.695417697 |
| wavelet-LLH_glrlm_GrayLevelVariance_32_binCount | 0.5627694 | 0.3798752 | 0.703170916 |
| wavelet-LLH_glrlm_HighGrayLevelRunEmphasis_32_binCount | 0.3923411 | 0.175666711 | 0.572538324 |
| wavelet-LLH_glrlm_LongRunEmphasis_32_binCount | 0.6892812 | 0.54386525 | 0.7944007 |
| wavelet-LLH_glrlm_LongRunHighGrayLevelEmphasis_32_binCount | 0.4733165 | 0.271987659 | 0.635141012 |
| wavelet-LLH_glrlm_LongRunLowGrayLevelEmphasis_32_binCount | 0.0641773 | -0.17162119 | 0.292529767 |
| wavelet-LLH_glrlm_LowGrayLevelRunEmphasis_32_binCount | 0.0552485 | -0.17989835 | 0.284074929 |
| wavelet-LLH_glrlm_RunEntropy_32_binCount | 0.3617924 | 0.140204062 | 0.548436404 |
| wavelet-LLH_glrlm_RunLengthNonUniformity_32_binCount | 0.7037698 | 0.564192478 | 0.804294598 |
| wavelet-LLH_glrlm_RunLengthNonUniformityNormalized_32_binCount | 0.6637071 | 0.51012151 | 0.776224589 |
| wavelet-LLH_glrlm_RunPercentage_32_binCount | 0.6774239 | 0.528333902 | 0.785940105 |
| wavelet-LLH_glrlm_RunVariance_32_binCount | 0.7162157 | 0.580500201 | 0.813158482 |
| wavelet-LLH_glrlm_ShortRunEmphasis_32_binCount | 0.6440451 | 0.484345093 | 0.762173201 |
| wavelet-LLH_glrlm_ShortRunHighGrayLevelEmphasis_32_binCount | 0.4515373 | 0.244509729 | 0.618987049 |
| wavelet-LLH_glrlm_ShortRunLowGrayLevelEmphasis_32_binCount | 0.089827 | -0.14581349 | 0.315568178 |
| wavelet-LLH_glszm_GrayLevelNonUniformity_32_binCount | 0.7612766 | 0.642892036 | 0.84414183 |
| wavelet-LLH_glszm_GrayLevelNonUniformityNormalized_32_binCount | 0.4177986 | 0.206085639 | 0.592205914 |
| wavelet-LLH_glszm_GrayLevelVariance_32_binCount | 0.4526374 | 0.245723789 | 0.619874037 |
| wavelet-LLH_glszm_HighGrayLevelZoneEmphasis_32_binCount | 0.3436256 | 0.120638277 | 0.533389552 |
| wavelet-LLH_glszm_LargeAreaEmphasis_32_binCount | 0.7708496 | 0.655974681 | 0.850767124 |
| wavelet-LLH_glszm_LargeAreaHighGrayLevelEmphasis_32_binCount | 0.6770342 | 0.528940532 | 0.785292561 |
| wavelet-LLH_glszm_LargeAreaLowGrayLevelEmphasis_32_binCount | 0.4001086 | 0.186107251 | 0.578045538 |
| wavelet-LLH_glszm_LowGrayLevelZoneEmphasis_32_binCount | 0.0957875 | -0.13962409 | 0.320800896 |
| wavelet-LLH_glszm_SizeZoneNonUniformity_32_binCount | 0.4438007 | 0.234977476 | 0.613152108 |
| wavelet-LLH_glszm_SizeZoneNonUniformityNormalized_32_binCount | 0.5808849 | 0.405012895 | 0.715673408 |
| wavelet-LLH_glszm_SmallAreaEmphasis_32_binCount | 0.5580255 | 0.376473644 | 0.698715957 |
| wavelet-LLH_glszm_SmallAreaHighGrayLevelEmphasis_32_binCount | 0.359054 | 0.138164294 | 0.545767725 |
| wavelet-LLH_glszm_SmallAreaLowGrayLevelEmphasis_32_binCount | 0.0536613 | -0.17745947 | 0.280242988 |
| wavelet-LLH_glszm_ZoneEntropy_32_binCount | 0.5792292 | 0.400455845 | 0.715352569 |
| wavelet-LLH_glszm_ZonePercentage_32_binCount | 0.6497472 | 0.491478627 | 0.766365479 |
| wavelet-LLH_glszm_ZoneVariance_32_binCount | 0.7725084 | 0.658305316 | 0.851895578 |
| wavelet-LLH_gldm_DependenceEntropy_32_binCount | 0.3591421 | 0.137657867 | 0.54610753 |
| wavelet-LLH_gldm_DependenceNonUniformity_32_binCount | 0.8290209 | 0.739353876 | 0.889814105 |
| wavelet-LLH_gldm_DependenceNonUniformityNormalized_32_binCount | 0.6631534 | 0.509022631 | 0.775953334 |
| wavelet-LLH_gldm_DependenceVariance_32_binCount | 0.6884359 | 0.543156766 | 0.793668161 |
| wavelet-LLH_gldm_GrayLevelNonUniformity_32_binCount | 0.9344297 | 0.896968688 | 0.958581612 |
| wavelet-LLH_gldm_GrayLevelVariance_32_binCount | 0.5662036 | 0.384255019 | 0.705682575 |
| wavelet-LLH_gldm_HighGrayLevelEmphasis_32_binCount | 0.3966307 | 0.180559459 | 0.575954471 |
| wavelet-LLH_gldm_LargeDependenceEmphasis_32_binCount | 0.6681795 | 0.516055333 | 0.779394772 |
| wavelet-LLH_gldm_LargeDependenceHighGrayLevelEmphasis_32_binCount | 0.5442278 | 0.357141628 | 0.689261429 |
| wavelet-LLH_gldm_LargeDependenceLowGrayLevelEmphasis_32_binCount | 0.0526489 | -0.18293668 | 0.281981812 |
| wavelet-LLH_gldm_LowGrayLevelEmphasis_32_binCount | 0.0556739 | -0.17954096 | 0.284499921 |
| wavelet-LLH_gldm_SmallDependenceEmphasis_32_binCount | 0.6465513 | 0.487095396 | 0.764146324 |
| wavelet-LLH_gldm_SmallDependenceHighGrayLevelEmphasis_32_binCount | 0.5764387 | 0.396743015 | 0.713369298 |
| wavelet-LLH_gldm_SmallDependenceLowGrayLevelEmphasis_32_binCount | 0.6597012 | 0.505760647 | 0.773064218 |
| wavelet-LLH_ngtdm_Busyness_32_binCount | 0.8277852 | 0.735191818 | 0.889659672 |
| wavelet-LLH_ngtdm_Coarseness_32_binCount | 0.8534111 | 0.775078399 | 0.905945308 |
| wavelet-LLH_ngtdm_Complexity_32_binCount | 0.474709 | 0.271468266 | 0.637081882 |
| wavelet-LLH_ngtdm_Contrast_32_binCount | 0.589229 | 0.413258406 | 0.722637881 |
| wavelet-LLH_ngtdm_Strength_32_binCount | 0.6637046 | 0.509722481 | 0.776354505 |
| wavelet-LHL_firstorder_10Percentile_32_binCount | 0.5345528 | 0.345408653 | 0.681944702 |
| wavelet-LHL_firstorder_90Percentile_32_binCount | 0.3790746 | 0.16352653 | 0.560649796 |
| wavelet-LHL_firstorder_Energy_32_binCount | 0.4801072 | 0.280349848 | 0.64025702 |
| wavelet-LHL_firstorder_Entropy_32_binCount | 0.5518261 | 0.368835241 | 0.694074971 |
| wavelet-LHL_firstorder_InterquartileRange_32_binCount | 0.526106 | 0.335185242 | 0.67554324 |
| wavelet-LHL_firstorder_Kurtosis_32_binCount | 0.7282865 | 0.596563813 | 0.821665066 |
| wavelet-LHL_firstorder_Maximum_32_binCount | 0.7120003 | 0.570979302 | 0.811416931 |
| wavelet-LHL_firstorder_MeanAbsoluteDeviation_32_binCount | 0.6232102 | 0.458329661 | 0.746813123 |
| wavelet-LHL_firstorder_Mean_32_binCount | 0.6757163 | 0.526949911 | 0.784436465 |
| wavelet-LHL_firstorder_Median_32_binCount | 0.697232 | 0.555957919 | 0.799526022 |
| wavelet-LHL_firstorder_Minimum_32_binCount | 0.6625274 | 0.509950145 | 0.774919656 |
| wavelet-LHL_firstorder_Range_32_binCount | 0.7319574 | 0.600630959 | 0.824505197 |
| wavelet-LHL_firstorder_RobustMeanAbsoluteDeviation_32_binCount | 0.4973783 | 0.300487428 | 0.653703403 |
| wavelet-LHL_firstorder_RootMeanSquared_32_binCount | 0.7722864 | 0.658977271 | 0.851447786 |
| wavelet-LHL_firstorder_Skewness_32_binCount | -0.008116 | -0.24131682 | 0.225296292 |
| wavelet-LHL_firstorder_TotalEnergy_32_binCount | 0.4801072 | 0.280349848 | 0.64025702 |
| wavelet-LHL_firstorder_Uniformity_32_binCount | 0.68107 | 0.534167005 | 0.788192662 |
| wavelet-LHL_firstorder_Variance_32_binCount | 0.6175616 | 0.451322016 | 0.742626301 |
| wavelet-LHL_glcm_Autocorrelation_32_binCount | -0.045076 | -0.27230758 | 0.187613632 |
| wavelet-LHL_glcm_ClusterProminence_32_binCount | 0.2459994 | 0.012545598 | 0.453291525 |
| wavelet-LHL_glcm_ClusterShade_32_binCount | 0.1359458 | -0.08271706 | 0.347341767 |
| wavelet-LHL_glcm_ClusterTendency_32_binCount | 0.4071209 | 0.192496808 | 0.584313524 |
| wavelet-LHL_glcm_Contrast_32_binCount | 0.4261183 | 0.215125424 | 0.59900292 |
| wavelet-LHL_glcm_Correlation_32_binCount | 0.60156 | 0.429796331 | 0.73134935 |
| wavelet-LHL_glcm_DifferenceAverage_32_binCount | 0.4903579 | 0.293184139 | 0.647891417 |
| wavelet-LHL_glcm_DifferenceEntropy_32_binCount | 0.5240724 | 0.334807188 | 0.673198991 |
| wavelet-LHL_glcm_DifferenceVariance_32_binCount | 0.4368444 | 0.227847428 | 0.607309501 |
| wavelet-LHL_glcm_Id_32_binCount | 0.5829962 | 0.407615452 | 0.71724936 |
| wavelet-LHL_glcm_Idm_32_binCount | 0.5722066 | 0.394101634 | 0.709267203 |
| wavelet-LHL_glcm_Idmn_32_binCount | 0.4260661 | 0.215241801 | 0.598887914 |
| wavelet-LHL_glcm_Idn_32_binCount | 0.5007634 | 0.305966339 | 0.655734082 |
| wavelet-LHL_glcm_Imc1_32_binCount | 0.599165 | 0.42793504 | 0.729174886 |
| wavelet-LHL_glcm_Imc2_32_binCount | 0.6712782 | 0.52054192 | 0.781466281 |
| wavelet-LHL_glcm_InverseVariance_32_binCount | 0.5396491 | 0.350759769 | 0.686110235 |
| wavelet-LHL_glcm_JointAverage_32_binCount | 0.0026178 | -0.22799243 | 0.233478452 |
| wavelet-LHL_glcm_JointEnergy_32_binCount | 0.7729669 | 0.659898818 | 0.85192106 |
| wavelet-LHL_glcm_JointEntropy_32_binCount | 0.5502682 | 0.366910853 | 0.692909094 |
| wavelet-LHL_glcm_MCC_32_binCount | 0.290322 | 0.062665455 | 0.489265353 |
| wavelet-LHL_glcm_MaximumProbability_32_binCount | 0.7037696 | 0.564767568 | 0.804107365 |
| wavelet-LHL_glcm_SumAverage_32_binCount | 0.0026178 | -0.22799243 | 0.233478452 |
| wavelet-LHL_glcm_SumEntropy_32_binCount | 0.5746483 | 0.397197882 | 0.711060972 |
| wavelet-LHL_glcm_SumSquares_32_binCount | 0.4103156 | 0.19632304 | 0.586776317 |
| wavelet-LHL_glrlm_GrayLevelNonUniformity_32_binCount | 0.943154 | 0.901731167 | 0.966144684 |
| wavelet-LHL_glrlm_GrayLevelNonUniformityNormalized_32_binCount | 0.5637876 | 0.383047473 | 0.703220723 |
| wavelet-LHL_glrlm_GrayLevelVariance_32_binCount | 0.4016836 | 0.186491904 | 0.57990433 |
| wavelet-LHL_glrlm_HighGrayLevelRunEmphasis_32_binCount | -0.04792 | -0.27499155 | 0.184894683 |
| wavelet-LHL_glrlm_LongRunEmphasis_32_binCount | 0.801555 | 0.699715126 | 0.871466334 |
| wavelet-LHL_glrlm_LongRunHighGrayLevelEmphasis_32_binCount | 0.7731031 | 0.659707537 | 0.852129939 |
| wavelet-LHL_glrlm_LongRunLowGrayLevelEmphasis_32_binCount | 0.6325948 | 0.469641338 | 0.753876505 |
| wavelet-LHL_glrlm_LowGrayLevelRunEmphasis_32_binCount | 0.2818277 | 0.056877497 | 0.480539805 |
| wavelet-LHL_glrlm_RunEntropy_32_binCount | 0.2628819 | 0.037679204 | 0.464040809 |
| wavelet-LHL_glrlm_RunLengthNonUniformity_32_binCount | 0.5886407 | 0.412229393 | 0.722307821 |
| wavelet-LHL_glrlm_RunLengthNonUniformityNormalized_32_binCount | 0.6262338 | 0.462429286 | 0.748928649 |
| wavelet-LHL_glrlm_RunPercentage_32_binCount | 0.6915677 | 0.548325836 | 0.795552656 |
| wavelet-LHL_glrlm_RunVariance_32_binCount | 0.7788679 | 0.667750663 | 0.856068721 |
| wavelet-LHL_glrlm_ShortRunEmphasis_32_binCount | 0.6319575 | 0.469453906 | 0.753193039 |
| wavelet-LHL_glrlm_ShortRunHighGrayLevelEmphasis_32_binCount | 0.0946933 | -0.13066958 | 0.313988863 |
| wavelet-LHL_glrlm_ShortRunLowGrayLevelEmphasis_32_binCount | 0.3836552 | 0.169133243 | 0.564140104 |
| wavelet-LHL_glszm_GrayLevelNonUniformity_32_binCount | 0.6392246 | 0.47789869 | 0.7587688 |
| wavelet-LHL_glszm_GrayLevelNonUniformityNormalized_32_binCount | 0.3950294 | 0.184335969 | 0.572233801 |
| wavelet-LHL_glszm_GrayLevelVariance_32_binCount | 0.3439784 | 0.122509987 | 0.533008004 |
| wavelet-LHL_glszm_HighGrayLevelZoneEmphasis_32_binCount | -0.042356 | -0.27092027 | 0.190983319 |
| wavelet-LHL_glszm_LargeAreaEmphasis_32_binCount | 0.8671313 | 0.795486461 | 0.91492832 |
| wavelet-LHL_glszm_LargeAreaHighGrayLevelEmphasis_32_binCount | 0.8365055 | 0.750303459 | 0.894769062 |
| wavelet-LHL_glszm_LargeAreaLowGrayLevelEmphasis_32_binCount | 0.8007135 | 0.699126677 | 0.870719925 |
| wavelet-LHL_glszm_LowGrayLevelZoneEmphasis_32_binCount | 0.3808475 | 0.164279586 | 0.562624541 |
| wavelet-LHL_glszm_SizeZoneNonUniformity_32_binCount | 0.3459672 | 0.126694694 | 0.533729848 |
| wavelet-LHL_glszm_SizeZoneNonUniformityNormalized_32_binCount | 0.3741988 | 0.158936857 | 0.556320366 |
| wavelet-LHL_glszm_SmallAreaEmphasis_32_binCount | 0.3255735 | 0.105164206 | 0.516553678 |
| wavelet-LHL_glszm_SmallAreaHighGrayLevelEmphasis_32_binCount | 0.0325299 | -0.19636373 | 0.259688808 |
| wavelet-LHL_glszm_SmallAreaLowGrayLevelEmphasis_32_binCount | 0.1597078 | -0.077258 | 0.378842513 |
| wavelet-LHL_glszm_ZoneEntropy_32_binCount | 0.4716906 | 0.272103313 | 0.633056019 |
| wavelet-LHL_glszm_ZonePercentage_32_binCount | 0.526767 | 0.336055474 | 0.676017648 |
| wavelet-LHL_glszm_ZoneVariance_32_binCount | 0.8675052 | 0.796037578 | 0.915174532 |
| wavelet-LHL_gldm_DependenceEntropy_32_binCount | 0.6707973 | 0.520858624 | 0.780806185 |
| wavelet-LHL_gldm_DependenceNonUniformity_32_binCount | 0.7643842 | 0.647904234 | 0.846060285 |
| wavelet-LHL_gldm_DependenceNonUniformityNormalized_32_binCount | 0.5337648 | 0.346057892 | 0.6807389 |
| wavelet-LHL_gldm_DependenceVariance_32_binCount | 0.7148415 | 0.579760375 | 0.811841142 |
| wavelet-LHL_gldm_GrayLevelNonUniformity_32_binCount | 0.9260977 | 0.884251301 | 0.95322098 |
| wavelet-LHL_gldm_GrayLevelVariance_32_binCount | 0.4151846 | 0.201973393 | 0.590605273 |
| wavelet-LHL_gldm_HighGrayLevelEmphasis_32_binCount | -0.04785 | -0.27494638 | 0.1849747 |
| wavelet-LHL_gldm_LargeDependenceEmphasis_32_binCount | 0.7464947 | 0.623037659 | 0.83379421 |
| wavelet-LHL_gldm_LargeDependenceHighGrayLevelEmphasis_32_binCount | 0.5195502 | 0.326410651 | 0.670892297 |
| wavelet-LHL_gldm_LargeDependenceLowGrayLevelEmphasis_32_binCount | 0.2441373 | 0.016925897 | 0.448548468 |
| wavelet-LHL_gldm_LowGrayLevelEmphasis_32_binCount | 0.2499483 | 0.023008006 | 0.453530502 |
| wavelet-LHL_gldm_SmallDependenceEmphasis_32_binCount | 0.5417837 | 0.355252929 | 0.687009705 |
| wavelet-LHL_gldm_SmallDependenceHighGrayLevelEmphasis_32_binCount | 0.4955969 | 0.299994566 | 0.651685351 |
| wavelet-LHL_gldm_SmallDependenceLowGrayLevelEmphasis_32_binCount | 0.5157123 | 0.32136131 | 0.66813591 |
| wavelet-LHL_ngtdm_Busyness_32_binCount | 0.8558295 | 0.771232628 | 0.909520419 |
| wavelet-LHL_ngtdm_Coarseness_32_binCount | 0.8090699 | 0.710285343 | 0.876567737 |
| wavelet-LHL_ngtdm_Complexity_32_binCount | 0.3619657 | 0.144160867 | 0.546901619 |
| wavelet-LHL_ngtdm_Contrast_32_binCount | 0.4583833 | 0.251967298 | 0.624542983 |
| wavelet-LHL_ngtdm_Strength_32_binCount | 0.7700177 | 0.655771041 | 0.849909579 |
| wavelet-LHH_firstorder_10Percentile_32_binCount | 0.5084034 | 0.314058912 | 0.661993153 |
| wavelet-LHH_firstorder_90Percentile_32_binCount | 0.5563753 | 0.37218835 | 0.698320708 |
| wavelet-LHH_firstorder_Energy_32_binCount | 0.45944 | 0.256035579 | 0.624217516 |
| wavelet-LHH_firstorder_Entropy_32_binCount | 0.4354656 | 0.226321145 | 0.606197009 |
| wavelet-LHH_firstorder_InterquartileRange_32_binCount | 0.5462459 | 0.360261186 | 0.690535638 |
| wavelet-LHH_firstorder_Kurtosis_32_binCount | 0.4399247 | 0.2344669 | 0.608458886 |
| wavelet-LHH_firstorder_Maximum_32_binCount | 0.5125417 | 0.320467984 | 0.664591294 |
| wavelet-LHH_firstorder_MeanAbsoluteDeviation_32_binCount | 0.6276077 | 0.463122675 | 0.750300191 |
| wavelet-LHH_firstorder_Mean_32_binCount | 0.4721529 | 0.269388965 | 0.634731696 |
| wavelet-LHH_firstorder_Median_32_binCount | 0.6370659 | 0.474861964 | 0.757295221 |
| wavelet-LHH_firstorder_Minimum_32_binCount | 0.6263727 | 0.462576512 | 0.749043192 |
| wavelet-LHH_firstorder_Range_32_binCount | 0.6265819 | 0.463140435 | 0.749090992 |
| wavelet-LHH_firstorder_RobustMeanAbsoluteDeviation_32_binCount | 0.5458016 | 0.359718852 | 0.690201109 |
| wavelet-LHH_firstorder_RootMeanSquared_32_binCount | 0.6489794 | 0.49069764 | 0.765740527 |
| wavelet-LHH_firstorder_Skewness_32_binCount | 0.0922783 | -0.14595667 | 0.319248339 |
| wavelet-LHH_firstorder_TotalEnergy_32_binCount | 0.45944 | 0.256035579 | 0.624217516 |
| wavelet-LHH_firstorder_Uniformity_32_binCount | 0.4501255 | 0.243787799 | 0.617507061 |
| wavelet-LHH_firstorder_Variance_32_binCount | 0.5350583 | 0.345240094 | 0.682621306 |
| wavelet-LHH_glcm_Autocorrelation_32_binCount | 0.0649994 | -0.17257675 | 0.29430775 |
| wavelet-LHH_glcm_ClusterProminence_32_binCount | 0.3282982 | 0.104604617 | 0.520442836 |
| wavelet-LHH_glcm_ClusterShade_32_binCount | -0.077464 | -0.30824192 | 0.160140563 |
| wavelet-LHH_glcm_ClusterTendency_32_binCount | 0.340607 | 0.117237914 | 0.530952468 |
| wavelet-LHH_glcm_Contrast_32_binCount | 0.3375958 | 0.113035787 | 0.528887146 |
| wavelet-LHH_glcm_Correlation_32_binCount | 0.4683089 | 0.265461947 | 0.631511778 |
| wavelet-LHH_glcm_DifferenceAverage_32_binCount | 0.3853958 | 0.167477337 | 0.56711943 |
| wavelet-LHH_glcm_DifferenceEntropy_32_binCount | 0.4160264 | 0.203138799 | 0.591187688 |
| wavelet-LHH_glcm_DifferenceVariance_32_binCount | 0.3867032 | 0.168582973 | 0.568327843 |
| wavelet-LHH_glcm_Id_32_binCount | 0.4504062 | 0.243750823 | 0.617875322 |
| wavelet-LHH_glcm_Idm_32_binCount | 0.4441471 | 0.236301526 | 0.613045815 |
| wavelet-LHH_glcm_Idmn_32_binCount | 0.3348927 | 0.110020778 | 0.526690809 |
| wavelet-LHH_glcm_Idn_32_binCount | 0.3926982 | 0.175912741 | 0.572892028 |
| wavelet-LHH_glcm_Imc1_32_binCount | 0.4670625 | 0.262298882 | 0.631225653 |
| wavelet-LHH_glcm_Imc2_32_binCount | 0.6191123 | 0.451935694 | 0.744234222 |
| wavelet-LHH_glcm_InverseVariance_32_binCount | 0.2882608 | 0.058978856 | 0.488238977 |
| wavelet-LHH_glcm_JointAverage_32_binCount | 0.0634569 | -0.17428132 | 0.293009908 |
| wavelet-LHH_glcm_JointEnergy_32_binCount | 0.4122123 | 0.198866851 | 0.588123256 |
| wavelet-LHH_glcm_JointEntropy_32_binCount | 0.4274437 | 0.216924882 | 0.599934823 |
| wavelet-LHH_glcm_MCC_32_binCount | 0.0172579 | -0.2174369 | 0.24934061 |
| wavelet-LHH_glcm_MaximumProbability_32_binCount | 0.4324549 | 0.222512799 | 0.60396432 |
| wavelet-LHH_glcm_SumAverage_32_binCount | 0.0634569 | -0.17428132 | 0.293009908 |
| wavelet-LHH_glcm_SumEntropy_32_binCount | 0.4450743 | 0.237830953 | 0.613586442 |
| wavelet-LHH_glcm_SumSquares_32_binCount | 0.3408677 | 0.117261011 | 0.531285203 |
| wavelet-LHH_glrlm_GrayLevelNonUniformity_32_binCount | 0.8876638 | 0.820882512 | 0.929708253 |
| wavelet-LHH_glrlm_GrayLevelNonUniformityNormalized_32_binCount | 0.4511585 | 0.246755424 | 0.617589639 |
| wavelet-LHH_glrlm_GrayLevelVariance_32_binCount | 0.3583619 | 0.136873273 | 0.545437227 |
| wavelet-LHH_glrlm_HighGrayLevelRunEmphasis_32_binCount | 0.049318 | -0.18780268 | 0.279874024 |
| wavelet-LHH_glrlm_LongRunEmphasis_32_binCount | 0.1702779 | -0.06618763 | 0.387973109 |
| wavelet-LHH_glrlm_LongRunHighGrayLevelEmphasis_32_binCount | 0.1273693 | -0.11021707 | 0.350338284 |
| wavelet-LHH_glrlm_LongRunLowGrayLevelEmphasis_32_binCount | 0.1925355 | -0.04348115 | 0.407472059 |
| wavelet-LHH_glrlm_LowGrayLevelRunEmphasis_32_binCount | 0.0968511 | -0.14150779 | 0.323425478 |
| wavelet-LHH_glrlm_RunEntropy_32_binCount | 0.2155328 | -0.01743361 | 0.426234865 |
| wavelet-LHH_glrlm_RunLengthNonUniformity_32_binCount | 0.5129402 | 0.317959617 | 0.66605083 |
| wavelet-LHH_glrlm_RunLengthNonUniformityNormalized_32_binCount | 0.5244103 | 0.333275105 | 0.674203076 |
| wavelet-LHH_glrlm_RunPercentage_32_binCount | 0.5504749 | 0.365130922 | 0.69382819 |
| wavelet-LHH_glrlm_RunVariance_32_binCount | 0.1117437 | -0.12530002 | 0.336049155 |
| wavelet-LHH_glrlm_ShortRunEmphasis_32_binCount | 0.5409297 | 0.354408964 | 0.686291296 |
| wavelet-LHH_glrlm_ShortRunHighGrayLevelEmphasis_32_binCount | -0.013182 | -0.24690084 | 0.220994288 |
| wavelet-LHH_glrlm_ShortRunLowGrayLevelEmphasis_32_binCount | 0.1829573 | -0.05407297 | 0.39951651 |
| wavelet-LHH_glszm_GrayLevelNonUniformity_32_binCount | 0.7587204 | 0.639972205 | 0.842196393 |
| wavelet-LHH_glszm_GrayLevelNonUniformityNormalized_32_binCount | 0.3507436 | 0.130612233 | 0.538253403 |
| wavelet-LHH_glszm_GrayLevelVariance_32_binCount | 0.4941506 | 0.296145199 | 0.651420168 |
| wavelet-LHH_glszm_HighGrayLevelZoneEmphasis_32_binCount | 0.0226048 | -0.21335536 | 0.254968409 |
| wavelet-LHH_glszm_LargeAreaEmphasis_32_binCount | 0.7386866 | 0.611750457 | 0.828574225 |
| wavelet-LHH_glszm_LargeAreaHighGrayLevelEmphasis_32_binCount | 0.6191122 | 0.451739152 | 0.744302384 |
| wavelet-LHH_glszm_LargeAreaLowGrayLevelEmphasis_32_binCount | 0.7284743 | 0.598372071 | 0.82130981 |
| wavelet-LHH_glszm_LowGrayLevelZoneEmphasis_32_binCount | 0.1251451 | -0.11241016 | 0.348332316 |
| wavelet-LHH_glszm_SizeZoneNonUniformity_32_binCount | 0.3370378 | 0.112024128 | 0.528609405 |
| wavelet-LHH_glszm_SizeZoneNonUniformityNormalized_32_binCount | 0.4380196 | 0.227891458 | 0.608775202 |
| wavelet-LHH_glszm_SmallAreaEmphasis_32_binCount | 0.4147183 | 0.200575587 | 0.590598684 |
| wavelet-LHH_glszm_SmallAreaHighGrayLevelEmphasis_32_binCount | -0.077665 | -0.30764375 | 0.159426389 |
| wavelet-LHH_glszm_SmallAreaLowGrayLevelEmphasis_32_binCount | 0.2908404 | 0.061869411 | 0.49034022 |
| wavelet-LHH_glszm_ZoneEntropy_32_binCount | 0.4036994 | 0.192848333 | 0.57976228 |
| wavelet-LHH_glszm_ZonePercentage_32_binCount | 0.4429277 | 0.233651908 | 0.612595673 |
| wavelet-LHH_glszm_ZoneVariance_32_binCount | 0.7383654 | 0.611298286 | 0.82835567 |
| wavelet-LHH_gldm_DependenceEntropy_32_binCount | 0.303849 | 0.076564602 | 0.500873495 |
| wavelet-LHH_gldm_DependenceNonUniformity_32_binCount | 0.4804423 | 0.27977831 | 0.640901883 |
| wavelet-LHH_gldm_DependenceNonUniformityNormalized_32_binCount | 0.2907693 | 0.06242357 | 0.489982435 |
| wavelet-LHH_gldm_DependenceVariance_32_binCount | 0.5193993 | 0.325785895 | 0.670946361 |
| wavelet-LHH_gldm_GrayLevelNonUniformity_32_binCount | 0.8361543 | 0.749739387 | 0.894550821 |
| wavelet-LHH_gldm_GrayLevelVariance_32_binCount | 0.3570655 | 0.135182522 | 0.54449487 |
| wavelet-LHH_gldm_HighGrayLevelEmphasis_32_binCount | 0.0526539 | -0.18459761 | 0.282967553 |
| wavelet-LHH_gldm_LargeDependenceEmphasis_32_binCount | 0.5454924 | 0.358831232 | 0.690158969 |
| wavelet-LHH_gldm_LargeDependenceHighGrayLevelEmphasis_32_binCount | 0.4951471 | 0.296377559 | 0.652558405 |
| wavelet-LHH_gldm_LargeDependenceLowGrayLevelEmphasis_32_binCount | 0.1985486 | -0.03599808 | 0.412034008 |
| wavelet-LHH_gldm_LowGrayLevelEmphasis_32_binCount | 0.0949134 | -0.14343423 | 0.321679096 |
| wavelet-LHH_gldm_SmallDependenceEmphasis_32_binCount | 0.4470355 | 0.238560809 | 0.615756097 |
| wavelet-LHH_gldm_SmallDependenceHighGrayLevelEmphasis_32_binCount | 0.2869904 | 0.056696652 | 0.487610184 |
| wavelet-LHH_gldm_SmallDependenceLowGrayLevelEmphasis_32_binCount | 0.4748143 | 0.271846623 | 0.637062434 |
| wavelet-LHH_ngtdm_Busyness_32_binCount | 0.588384 | 0.414282064 | 0.721264263 |
| wavelet-LHH_ngtdm_Coarseness_32_binCount | 0.844635 | 0.762183909 | 0.900153514 |
| wavelet-LHH_ngtdm_Complexity_32_binCount | 0.2489432 | 0.015681321 | 0.455779177 |
| wavelet-LHH_ngtdm_Contrast_32_binCount | 0.3334685 | 0.109037428 | 0.525258724 |
| wavelet-LHH_ngtdm_Strength_32_binCount | 0.7155587 | 0.57995418 | 0.812590947 |
| wavelet-HLL_firstorder_10Percentile_32_binCount | 0.7125388 | 0.576469178 | 0.810289602 |
| wavelet-HLL_firstorder_90Percentile_32_binCount | 0.6071923 | 0.437617453 | 0.735232705 |
| wavelet-HLL_firstorder_Energy_32_binCount | 0.5793594 | 0.402379865 | 0.714810986 |
| wavelet-HLL_firstorder_Entropy_32_binCount | 0.5340523 | 0.343846997 | 0.681925555 |
| wavelet-HLL_firstorder_InterquartileRange_32_binCount | 0.702403 | 0.562713241 | 0.803219817 |
| wavelet-HLL_firstorder_Kurtosis_32_binCount | 0.4402953 | 0.231035513 | 0.610352825 |
| wavelet-HLL_firstorder_Maximum_32_binCount | 0.7735032 | 0.660669787 | 0.852281105 |
| wavelet-HLL_firstorder_MeanAbsoluteDeviation_32_binCount | 0.7892279 | 0.68267592 | 0.86300435 |
| wavelet-HLL_firstorder_Mean_32_binCount | 0.8456527 | 0.763333604 | 0.900921959 |
| wavelet-HLL_firstorder_Median_32_binCount | 0.8468838 | 0.765212522 | 0.901714848 |
| wavelet-HLL_firstorder_Minimum_32_binCount | 0.7110268 | 0.573317993 | 0.809586318 |
| wavelet-HLL_firstorder_Range_32_binCount | 0.7685821 | 0.653246686 | 0.849086387 |
| wavelet-HLL_firstorder_RobustMeanAbsoluteDeviation_32_binCount | 0.6846341 | 0.53885808 | 0.790732054 |
| wavelet-HLL_firstorder_RootMeanSquared_32_binCount | 0.8472278 | 0.765871885 | 0.901898778 |
| wavelet-HLL_firstorder_Skewness_32_binCount | 0.163588 | -0.0691266 | 0.380020029 |
| wavelet-HLL_firstorder_TotalEnergy_32_binCount | 0.5793594 | 0.402379865 | 0.714810986 |
| wavelet-HLL_firstorder_Uniformity_32_binCount | 0.5378847 | 0.348844649 | 0.684692239 |
| wavelet-HLL_firstorder_Variance_32_binCount | 0.7357577 | 0.608024723 | 0.826457011 |
| wavelet-HLL_glcm_Autocorrelation_32_binCount | 0.0100822 | -0.21614049 | 0.237544877 |
| wavelet-HLL_glcm_ClusterProminence_32_binCount | 0.3453611 | 0.121931649 | 0.535087534 |
| wavelet-HLL_glcm_ClusterShade_32_binCount | 0.1729118 | -0.06265098 | 0.389835972 |
| wavelet-HLL_glcm_ClusterTendency_32_binCount | 0.4359523 | 0.225609957 | 0.607105891 |
| wavelet-HLL_glcm_Contrast_32_binCount | 0.4744277 | 0.27109944 | 0.636879013 |
| wavelet-HLL_glcm_Correlation_32_binCount | 0.5538549 | 0.369921057 | 0.696124882 |
| wavelet-HLL_glcm_DifferenceAverage_32_binCount | 0.5206508 | 0.327206538 | 0.671930896 |
| wavelet-HLL_glcm_DifferenceEntropy_32_binCount | 0.5342761 | 0.344019275 | 0.682131946 |
| wavelet-HLL_glcm_DifferenceVariance_32_binCount | 0.486634 | 0.285788511 | 0.6461988 |
| wavelet-HLL_glcm_Id_32_binCount | 0.5562657 | 0.371464219 | 0.698455861 |
| wavelet-HLL_glcm_Idm_32_binCount | 0.5526728 | 0.366955104 | 0.695799684 |
| wavelet-HLL_glcm_Idmn_32_binCount | 0.4765949 | 0.273700312 | 0.638537272 |
| wavelet-HLL_glcm_Idn_32_binCount | 0.5258598 | 0.333615925 | 0.675838978 |
| wavelet-HLL_glcm_Imc1_32_binCount | 0.6474096 | 0.488228283 | 0.76475731 |
| wavelet-HLL_glcm_Imc2_32_binCount | 0.5860645 | 0.409194002 | 0.720337526 |
| wavelet-HLL_glcm_InverseVariance_32_binCount | 0.2479585 | 0.015984146 | 0.454283081 |
| wavelet-HLL_glcm_JointAverage_32_binCount | 0.0129216 | -0.21204313 | 0.239347702 |
| wavelet-HLL_glcm_JointEnergy_32_binCount | 0.5151481 | 0.321088395 | 0.667550905 |
| wavelet-HLL_glcm_JointEntropy_32_binCount | 0.5351535 | 0.345175034 | 0.682761115 |
| wavelet-HLL_glcm_MCC_32_binCount | 0.2641375 | 0.032414785 | 0.468338687 |
| wavelet-HLL_glcm_MaximumProbability_32_binCount | 0.4911297 | 0.291618473 | 0.649464503 |
| wavelet-HLL_glcm_SumAverage_32_binCount | 0.0129216 | -0.21204313 | 0.239347702 |
| wavelet-HLL_glcm_SumEntropy_32_binCount | 0.5448643 | 0.35726065 | 0.689985698 |
| wavelet-HLL_glcm_SumSquares_32_binCount | 0.4482813 | 0.240066134 | 0.616707609 |
| wavelet-HLL_glrlm_GrayLevelNonUniformity_32_binCount | 0.9660491 | 0.945843502 | 0.978758627 |
| wavelet-HLL_glrlm_GrayLevelNonUniformityNormalized_32_binCount | 0.5376902 | 0.348372254 | 0.684633762 |
| wavelet-HLL_glrlm_GrayLevelVariance_32_binCount | 0.4447184 | 0.235743056 | 0.613993363 |
| wavelet-HLL_glrlm_HighGrayLevelRunEmphasis_32_binCount | 0.0150933 | -0.21137193 | 0.242277541 |
| wavelet-HLL_glrlm_LongRunEmphasis_32_binCount | 0.5628189 | 0.380427552 | 0.703027858 |
| wavelet-HLL_glrlm_LongRunHighGrayLevelEmphasis_32_binCount | 0.4753672 | 0.272236687 | 0.637594053 |
| wavelet-HLL_glrlm_LongRunLowGrayLevelEmphasis_32_binCount | 0.3087929 | 0.087637528 | 0.502285087 |
| wavelet-HLL_glrlm_LowGrayLevelRunEmphasis_32_binCount | -0.007817 | -0.23386969 | 0.221053305 |
| wavelet-HLL_glrlm_RunEntropy_32_binCount | 0.2924493 | 0.062685586 | 0.492115266 |
| wavelet-HLL_glrlm_RunLengthNonUniformity_32_binCount | 0.6459163 | 0.486790081 | 0.763513959 |
| wavelet-HLL_glrlm_RunLengthNonUniformityNormalized_32_binCount | 0.6085778 | 0.437774101 | 0.73681032 |
| wavelet-HLL_glrlm_RunPercentage_32_binCount | 0.6300419 | 0.465620552 | 0.752281228 |
| wavelet-HLL_glrlm_RunVariance_32_binCount | 0.5106655 | 0.31605529 | 0.663998134 |
| wavelet-HLL_glrlm_ShortRunEmphasis_32_binCount | 0.6180352 | 0.449994612 | 0.743646593 |
| wavelet-HLL_glrlm_ShortRunHighGrayLevelEmphasis_32_binCount | 0.1855907 | -0.04242978 | 0.39709865 |
| wavelet-HLL_glrlm_ShortRunLowGrayLevelEmphasis_32_binCount | 0.045628 | -0.18315902 | 0.271540337 |
| wavelet-HLL_glszm_GrayLevelNonUniformity_32_binCount | 0.5959333 | 0.423764544 | 0.726832401 |
| wavelet-HLL_glszm_GrayLevelNonUniformityNormalized_32_binCount | 0.3405655 | 0.116532535 | 0.53121623 |
| wavelet-HLL_glszm_GrayLevelVariance_32_binCount | 0.3927453 | 0.17719475 | 0.572400256 |
| wavelet-HLL_glszm_HighGrayLevelZoneEmphasis_32_binCount | 0.003429 | -0.22264738 | 0.231366599 |
| wavelet-HLL_glszm_LargeAreaEmphasis_32_binCount | 0.6974157 | 0.555702921 | 0.799818229 |
| wavelet-HLL_glszm_LargeAreaHighGrayLevelEmphasis_32_binCount | 0.681202 | 0.533087418 | 0.788700683 |
| wavelet-HLL_glszm_LargeAreaLowGrayLevelEmphasis_32_binCount | 0.5772641 | 0.400452774 | 0.713003861 |
| wavelet-HLL_glszm_LowGrayLevelZoneEmphasis_32_binCount | 0.1346802 | -0.09603521 | 0.353080444 |
| wavelet-HLL_glszm_SizeZoneNonUniformity_32_binCount | 0.3874632 | 0.171455777 | 0.568064891 |
| wavelet-HLL_glszm_SizeZoneNonUniformityNormalized_32_binCount | 0.4118052 | 0.201304686 | 0.586562613 |
| wavelet-HLL_glszm_SmallAreaEmphasis_32_binCount | 0.4020732 | 0.190284945 | 0.578773963 |
| wavelet-HLL_glszm_SmallAreaHighGrayLevelEmphasis_32_binCount | 0.0712314 | -0.15515078 | 0.293339306 |
| wavelet-HLL_glszm_SmallAreaLowGrayLevelEmphasis_32_binCount | 0.2161267 | -0.01859858 | 0.427651545 |
| wavelet-HLL_glszm_ZoneEntropy_32_binCount | 0.4453017 | 0.236967163 | 0.614226953 |
| wavelet-HLL_glszm_ZonePercentage_32_binCount | 0.5587314 | 0.374603507 | 0.70026175 |
| wavelet-HLL_glszm_ZoneVariance_32_binCount | 0.6983943 | 0.557017999 | 0.800505338 |
| wavelet-HLL_gldm_DependenceEntropy_32_binCount | 0.4833644 | 0.283150543 | 0.64319135 |
| wavelet-HLL_gldm_DependenceNonUniformity_32_binCount | 0.8562286 | 0.779445954 | 0.907740883 |
| wavelet-HLL_gldm_DependenceNonUniformityNormalized_32_binCount | 0.5317285 | 0.341239094 | 0.680088672 |
| wavelet-HLL_gldm_DependenceVariance_32_binCount | 0.5866967 | 0.409943213 | 0.720819626 |
| wavelet-HLL_gldm_GrayLevelNonUniformity_32_binCount | 0.9057649 | 0.853112438 | 0.940163596 |
| wavelet-HLL_gldm_GrayLevelVariance_32_binCount | 0.4526156 | 0.245090864 | 0.620103359 |
| wavelet-HLL_gldm_HighGrayLevelEmphasis_32_binCount | 0.0147095 | -0.21183052 | 0.241973832 |
| wavelet-HLL_gldm_LargeDependenceEmphasis_32_binCount | 0.6259812 | 0.460474873 | 0.74931375 |
| wavelet-HLL_gldm_LargeDependenceHighGrayLevelEmphasis_32_binCount | 0.4815723 | 0.280160287 | 0.642152593 |
| wavelet-HLL_gldm_LargeDependenceLowGrayLevelEmphasis_32_binCount | 0.2923827 | 0.0667955 | 0.490077043 |
| wavelet-HLL_gldm_LowGrayLevelEmphasis_32_binCount | -0.012057 | -0.23814046 | 0.217187589 |
| wavelet-HLL_gldm_SmallDependenceEmphasis_32_binCount | 0.5593124 | 0.375180334 | 0.700747026 |
| wavelet-HLL_gldm_SmallDependenceHighGrayLevelEmphasis_32_binCount | 0.5387117 | 0.350693674 | 0.685000039 |
| wavelet-HLL_gldm_SmallDependenceLowGrayLevelEmphasis_32_binCount | 0.4088524 | 0.195156955 | 0.58539982 |
| wavelet-HLL_ngtdm_Busyness_32_binCount | 0.8565038 | 0.779627016 | 0.907984341 |
| wavelet-HLL_ngtdm_Coarseness_32_binCount | 0.8452074 | 0.762806917 | 0.90059236 |
| wavelet-HLL_ngtdm_Complexity_32_binCount | 0.3381551 | 0.113269203 | 0.529517469 |
| wavelet-HLL_ngtdm_Contrast_32_binCount | 0.5031254 | 0.306144103 | 0.658576424 |
| wavelet-HLL_ngtdm_Strength_32_binCount | 0.7110629 | 0.573328484 | 0.809623702 |
| wavelet-HLH_firstorder_10Percentile_32_binCount | 0.6851334 | 0.539378937 | 0.791132334 |
| wavelet-HLH_firstorder_90Percentile_32_binCount | 0.7215682 | 0.588504335 | 0.816654276 |
| wavelet-HLH_firstorder_Energy_32_binCount | 0.5735142 | 0.394874237 | 0.71055539 |
| wavelet-HLH_firstorder_Entropy_32_binCount | 0.5268729 | 0.33491448 | 0.676579307 |
| wavelet-HLH_firstorder_InterquartileRange_32_binCount | 0.6882933 | 0.543316213 | 0.793453522 |
| wavelet-HLH_firstorder_Kurtosis_32_binCount | 0.3672179 | 0.146073187 | 0.552910102 |
| wavelet-HLH_firstorder_Maximum_32_binCount | 0.655343 | 0.499879988 | 0.770005685 |
| wavelet-HLH_firstorder_MeanAbsoluteDeviation_32_binCount | 0.7943835 | 0.689533548 | 0.866623655 |
| wavelet-HLH_firstorder_Mean_32_binCount | 0.4581797 | 0.252080504 | 0.624242745 |
| wavelet-HLH_firstorder_Median_32_binCount | 0.5258092 | 0.3344277 | 0.67546969 |
| wavelet-HLH_firstorder_Minimum_32_binCount | 0.6561452 | 0.500447268 | 0.770742384 |
| wavelet-HLH_firstorder_Range_32_binCount | 0.7001318 | 0.55848627 | 0.802004351 |
| wavelet-HLH_firstorder_RobustMeanAbsoluteDeviation_32_binCount | 0.6958585 | 0.553570748 | 0.798737751 |
| wavelet-HLH_firstorder_RootMeanSquared_32_binCount | 0.7892315 | 0.681950182 | 0.863223371 |
| wavelet-HLH_firstorder_Skewness_32_binCount | 0.0272735 | -0.20324132 | 0.255898067 |
| wavelet-HLH_firstorder_TotalEnergy_32_binCount | 0.5735142 | 0.394874237 | 0.71055539 |
| wavelet-HLH_firstorder_Uniformity_32_binCount | 0.5533908 | 0.367821506 | 0.696343373 |
| wavelet-HLH_firstorder_Variance_32_binCount | 0.7162585 | 0.580488615 | 0.813210377 |
| wavelet-HLH_glcm_Autocorrelation_32_binCount | 0.1606216 | -0.06866991 | 0.375513421 |
| wavelet-HLH_glcm_ClusterProminence_32_binCount | 0.3571771 | 0.1347448 | 0.544834543 |
| wavelet-HLH_glcm_ClusterShade_32_binCount | -0.142092 | -0.35905913 | 0.090709491 |
| wavelet-HLH_glcm_ClusterTendency_32_binCount | 0.4028557 | 0.186884596 | 0.581238503 |
| wavelet-HLH_glcm_Contrast_32_binCount | 0.4470937 | 0.238818825 | 0.615724035 |
| wavelet-HLH_glcm_Correlation_32_binCount | 0.4964954 | 0.298481166 | 0.6533991 |
| wavelet-HLH_glcm_DifferenceAverage_32_binCount | 0.4749582 | 0.271811834 | 0.637254884 |
| wavelet-HLH_glcm_DifferenceEntropy_32_binCount | 0.5190727 | 0.325265564 | 0.670746314 |
| wavelet-HLH_glcm_DifferenceVariance_32_binCount | 0.5328427 | 0.342643959 | 0.680911408 |
| wavelet-HLH_glcm_Id_32_binCount | 0.5397804 | 0.350817873 | 0.686247229 |
| wavelet-HLH_glcm_Idm_32_binCount | 0.5347469 | 0.344582312 | 0.68249091 |
| wavelet-HLH_glcm_Idmn_32_binCount | 0.4349416 | 0.224437138 | 0.606313116 |
| wavelet-HLH_glcm_Idn_32_binCount | 0.4814007 | 0.279533881 | 0.642187252 |
| wavelet-HLH_glcm_Imc1_32_binCount | 0.4908929 | 0.293121069 | 0.648580504 |
| wavelet-HLH_glcm_Imc2_32_binCount | 0.6140924 | 0.445808667 | 0.740479647 |
| wavelet-HLH_glcm_InverseVariance_32_binCount | 0.2424416 | 0.010111242 | 0.449614246 |
| wavelet-HLH_glcm_JointAverage_32_binCount | 0.1706802 | -0.05705469 | 0.383648117 |
| wavelet-HLH_glcm_JointEnergy_32_binCount | 0.5539331 | 0.368824802 | 0.69662525 |
| wavelet-HLH_glcm_JointEntropy_32_binCount | 0.5230332 | 0.330142415 | 0.673716894 |
| wavelet-HLH_glcm_MCC_32_binCount | -0.033372 | -0.26499474 | 0.201197706 |
| wavelet-HLH_glcm_MaximumProbability_32_binCount | 0.5500014 | 0.363631249 | 0.693813804 |
| wavelet-HLH_glcm_SumAverage_32_binCount | 0.1706802 | -0.05705469 | 0.383648117 |
| wavelet-HLH_glcm_SumEntropy_32_binCount | 0.5385993 | 0.349378547 | 0.685357053 |
| wavelet-HLH_glcm_SumSquares_32_binCount | 0.4137188 | 0.199530062 | 0.589765159 |
| wavelet-HLH_glrlm_GrayLevelNonUniformity_32_binCount | 0.8838071 | 0.819619121 | 0.926023849 |
| wavelet-HLH_glrlm_GrayLevelNonUniformityNormalized_32_binCount | 0.5239996 | 0.331324666 | 0.674444518 |
| wavelet-HLH_glrlm_GrayLevelVariance_32_binCount | 0.4057532 | 0.190442317 | 0.583435486 |
| wavelet-HLH_glrlm_HighGrayLevelRunEmphasis_32_binCount | 0.1533446 | -0.0757402 | 0.368892787 |
| wavelet-HLH_glrlm_LongRunEmphasis_32_binCount | 0.582108 | 0.404056294 | 0.717480936 |
| wavelet-HLH_glrlm_LongRunHighGrayLevelEmphasis_32_binCount | 0.5923132 | 0.417938942 | 0.724622712 |
| wavelet-HLH_glrlm_LongRunLowGrayLevelEmphasis_32_binCount | 0.2901268 | 0.06244574 | 0.489106753 |
| wavelet-HLH_glrlm_LowGrayLevelRunEmphasis_32_binCount | 0.3283349 | 0.10959112 | 0.518174197 |
| wavelet-HLH_glrlm_RunEntropy_32_binCount | 0.2292811 | -0.00327998 | 0.438152873 |
| wavelet-HLH_glrlm_RunLengthNonUniformity_32_binCount | 0.6691272 | 0.518505364 | 0.779668904 |
| wavelet-HLH_glrlm_RunLengthNonUniformityNormalized_32_binCount | 0.5920844 | 0.416659687 | 0.724807807 |
| wavelet-HLH_glrlm_RunPercentage_32_binCount | 0.6215452 | 0.454596005 | 0.746159993 |
| wavelet-HLH_glrlm_RunVariance_32_binCount | 0.5825924 | 0.404693243 | 0.717827849 |
| wavelet-HLH_glrlm_ShortRunEmphasis_32_binCount | 0.598363 | 0.424719252 | 0.729370801 |
| wavelet-HLH_glrlm_ShortRunHighGrayLevelEmphasis_32_binCount | 0.2308696 | 0.003406138 | 0.436969898 |
| wavelet-HLH_glrlm_ShortRunLowGrayLevelEmphasis_32_binCount | 0.4417125 | 0.236958111 | 0.609687793 |
| wavelet-HLH_glszm_GrayLevelNonUniformity_32_binCount | 0.8052583 | 0.705426992 | 0.873833144 |
| wavelet-HLH_glszm_GrayLevelNonUniformityNormalized_32_binCount | 0.4559467 | 0.249106155 | 0.622649952 |
| wavelet-HLH_glszm_GrayLevelVariance_32_binCount | 0.4407615 | 0.233009044 | 0.610128185 |
| wavelet-HLH_glszm_HighGrayLevelZoneEmphasis_32_binCount | 0.1703419 | -0.05734748 | 0.383321301 |
| wavelet-HLH_glszm_LargeAreaEmphasis_32_binCount | 0.8465168 | 0.764896392 | 0.901410218 |
| wavelet-HLH_glszm_LargeAreaHighGrayLevelEmphasis_32_binCount | 0.8392542 | 0.754608782 | 0.8965081 |
| wavelet-HLH_glszm_LargeAreaLowGrayLevelEmphasis_32_binCount | 0.6862693 | 0.539822066 | 0.792284271 |
| wavelet-HLH_glszm_LowGrayLevelZoneEmphasis_32_binCount | 0.3990508 | 0.185339202 | 0.577013841 |
| wavelet-HLH_glszm_SizeZoneNonUniformity_32_binCount | 0.5902335 | 0.414261701 | 0.723470209 |
| wavelet-HLH_glszm_SizeZoneNonUniformityNormalized_32_binCount | 0.5374084 | 0.348978792 | 0.684064669 |
| wavelet-HLH_glszm_SmallAreaEmphasis_32_binCount | 0.5402608 | 0.353037472 | 0.685996675 |
| wavelet-HLH_glszm_SmallAreaHighGrayLevelEmphasis_32_binCount | 0.1738498 | -0.05054955 | 0.384653395 |
| wavelet-HLH_glszm_SmallAreaLowGrayLevelEmphasis_32_binCount | 0.4319695 | 0.220755213 | 0.604077768 |
| wavelet-HLH_glszm_ZoneEntropy_32_binCount | 0.2612353 | 0.029062061 | 0.46601716 |
| wavelet-HLH_glszm_ZonePercentage_32_binCount | 0.5078364 | 0.311527996 | 0.66227556 |
| wavelet-HLH_glszm_ZoneVariance_32_binCount | 0.847292 | 0.766033228 | 0.901922432 |
| wavelet-HLH_gldm_DependenceEntropy_32_binCount | 0.4753064 | 0.272479192 | 0.63742236 |
| wavelet-HLH_gldm_DependenceNonUniformity_32_binCount | 0.9005829 | 0.845505054 | 0.936748105 |
| wavelet-HLH_gldm_DependenceNonUniformityNormalized_32_binCount | 0.4772825 | 0.274532946 | 0.639060418 |
| wavelet-HLH_gldm_DependenceVariance_32_binCount | 0.5870688 | 0.410355774 | 0.721113482 |
| wavelet-HLH_gldm_GrayLevelNonUniformity_32_binCount | 0.9452096 | 0.913596384 | 0.96547093 |
| wavelet-HLH_gldm_GrayLevelVariance_32_binCount | 0.4143977 | 0.200330386 | 0.590293565 |
| wavelet-HLH_gldm_HighGrayLevelEmphasis_32_binCount | 0.1548309 | -0.0742454 | 0.370217945 |
| wavelet-HLH_gldm_LargeDependenceEmphasis_32_binCount | 0.6196122 | 0.452207572 | 0.744725414 |
| wavelet-HLH_gldm_LargeDependenceHighGrayLevelEmphasis_32_binCount | 0.5406691 | 0.354200265 | 0.68605345 |
| wavelet-HLH_gldm_LargeDependenceLowGrayLevelEmphasis_32_binCount | 0.3054242 | 0.081943575 | 0.500453523 |
| wavelet-HLH_gldm_LowGrayLevelEmphasis_32_binCount | 0.3242287 | 0.105437887 | 0.51462069 |
| wavelet-HLH_gldm_SmallDependenceEmphasis_32_binCount | 0.5223276 | 0.329297229 | 0.673178698 |
| wavelet-HLH_gldm_SmallDependenceHighGrayLevelEmphasis_32_binCount | 0.4766138 | 0.275029844 | 0.638032594 |
| wavelet-HLH_gldm_SmallDependenceLowGrayLevelEmphasis_32_binCount | 0.6808408 | 0.532569499 | 0.788457671 |
| wavelet-HLH_ngtdm_Busyness_32_binCount | 0.7266013 | 0.595393111 | 0.820141319 |
| wavelet-HLH_ngtdm_Coarseness_32_binCount | 0.8281424 | 0.737807822 | 0.889306701 |
| wavelet-HLH_ngtdm_Complexity_32_binCount | 0.3494117 | 0.125882797 | 0.538627848 |
| wavelet-HLH_ngtdm_Contrast_32_binCount | 0.4093963 | 0.194462939 | 0.586388423 |
| wavelet-HLH_ngtdm_Strength_32_binCount | 0.5944019 | 0.419648304 | 0.726487396 |
| wavelet-HHL_firstorder_10Percentile_32_binCount | 0.478591 | 0.279261629 | 0.638802157 |
| wavelet-HHL_firstorder_90Percentile_32_binCount | 0.4865366 | 0.288602101 | 0.644965202 |
| wavelet-HHL_firstorder_Energy_32_binCount | 0.5624997 | 0.381115771 | 0.702390512 |
| wavelet-HHL_firstorder_Entropy_32_binCount | 0.5236045 | 0.331475323 | 0.673906288 |
| wavelet-HHL_firstorder_InterquartileRange_32_binCount | 0.4758601 | 0.275251718 | 0.637004604 |
| wavelet-HHL_firstorder_Kurtosis_32_binCount | 0.5093101 | 0.313317776 | 0.66339168 |
| wavelet-HHL_firstorder_Maximum_32_binCount | 0.7966779 | 0.693303805 | 0.868022052 |
| wavelet-HHL_firstorder_MeanAbsoluteDeviation_32_binCount | 0.7190278 | 0.585501135 | 0.814741719 |
| wavelet-HHL_firstorder_Mean_32_binCount | 0.3927067 | 0.178307097 | 0.571868173 |
| wavelet-HHL_firstorder_Median_32_binCount | 0.1911562 | -0.04535504 | 0.406507001 |
| wavelet-HHL_firstorder_Minimum_32_binCount | 0.7720569 | 0.658638753 | 0.851296577 |
| wavelet-HHL_firstorder_Range_32_binCount | 0.8113653 | 0.714337742 | 0.87788645 |
| wavelet-HHL_firstorder_RobustMeanAbsoluteDeviation_32_binCount | 0.47758 | 0.277564582 | 0.638222184 |
| wavelet-HHL_firstorder_RootMeanSquared_32_binCount | 0.8563984 | 0.77969533 | 0.907852942 |
| wavelet-HHL_firstorder_Skewness_32_binCount | -0.134662 | -0.35910611 | 0.102924908 |
| wavelet-HHL_firstorder_TotalEnergy_32_binCount | 0.5624997 | 0.381115771 | 0.702390512 |
| wavelet-HHL_firstorder_Uniformity_32_binCount | 0.5650264 | 0.384054823 | 0.704344254 |
| wavelet-HHL_firstorder_Variance_32_binCount | 0.7334915 | 0.605144046 | 0.824817349 |
| wavelet-HHL_glcm_Autocorrelation_32_binCount | 0.0888662 | -0.14907259 | 0.316013747 |
| wavelet-HHL_glcm_ClusterProminence_32_binCount | 0.1467477 | -0.09119066 | 0.367824834 |
| wavelet-HHL_glcm_ClusterShade_32_binCount | 0.0923805 | -0.14134848 | 0.316771991 |
| wavelet-HHL_glcm_ClusterTendency_32_binCount | 0.2812216 | 0.05040948 | 0.482826551 |
| wavelet-HHL_glcm_Contrast_32_binCount | 0.284004 | 0.053465679 | 0.485122898 |
| wavelet-HHL_glcm_Correlation_32_binCount | 0.4369123 | 0.227368964 | 0.607593002 |
| wavelet-HHL_glcm_DifferenceAverage_32_binCount | 0.4128008 | 0.198358037 | 0.589088443 |
| wavelet-HHL_glcm_DifferenceEntropy_32_binCount | 0.5197969 | 0.326429757 | 0.671185815 |
| wavelet-HHL_glcm_DifferenceVariance_32_binCount | 0.3184269 | 0.091231892 | 0.513491554 |
| wavelet-HHL_glcm_Id_32_binCount | 0.5400987 | 0.351941544 | 0.686211901 |
| wavelet-HHL_glcm_Idm_32_binCount | 0.5347072 | 0.345103765 | 0.682247029 |
| wavelet-HHL_glcm_Idmn_32_binCount | 0.2938169 | 0.064170723 | 0.493250638 |
| wavelet-HHL_glcm_Idn_32_binCount | 0.4335091 | 0.222573418 | 0.60527251 |
| wavelet-HHL_glcm_Imc1_32_binCount | 0.4124894 | 0.198201942 | 0.588757366 |
| wavelet-HHL_glcm_Imc2_32_binCount | 0.5053751 | 0.308529994 | 0.660414728 |
| wavelet-HHL_glcm_InverseVariance_32_binCount | 0.3976865 | 0.18620179 | 0.574873453 |
| wavelet-HHL_glcm_JointAverage_32_binCount | 0.0437463 | -0.19293452 | 0.274571787 |
| wavelet-HHL_glcm_JointEnergy_32_binCount | 0.5487924 | 0.364259542 | 0.692119753 |
| wavelet-HHL_glcm_JointEntropy_32_binCount | 0.5208199 | 0.328079117 | 0.67180491 |
| wavelet-HHL_glcm_MCC_32_binCount | 0.353406 | 0.131268223 | 0.541452915 |
| wavelet-HHL_glcm_MaximumProbability_32_binCount | 0.5266023 | 0.337524212 | 0.675253617 |
| wavelet-HHL_glcm_SumAverage_32_binCount | 0.0437463 | -0.19293452 | 0.274571787 |
| wavelet-HHL_glcm_SumEntropy_32_binCount | 0.5353864 | 0.346217564 | 0.682651533 |
| wavelet-HHL_glcm_SumSquares_32_binCount | 0.2804291 | 0.049556177 | 0.482164159 |
| wavelet-HHL_glrlm_GrayLevelNonUniformity_32_binCount | 0.9084562 | 0.857235804 | 0.941891796 |
| wavelet-HHL_glrlm_GrayLevelNonUniformityNormalized_32_binCount | 0.5451901 | 0.357581892 | 0.690259309 |
| wavelet-HHL_glrlm_GrayLevelVariance_32_binCount | 0.2870845 | 0.056811655 | 0.487682277 |
| wavelet-HHL_glrlm_HighGrayLevelRunEmphasis_32_binCount | 0.0884462 | -0.14942398 | 0.315597149 |
| wavelet-HHL_glrlm_LongRunEmphasis_32_binCount | 0.3602052 | 0.143921559 | 0.544692711 |
| wavelet-HHL_glrlm_LongRunHighGrayLevelEmphasis_32_binCount | 0.3261932 | 0.106013523 | 0.51698517 |
| wavelet-HHL_glrlm_LongRunLowGrayLevelEmphasis_32_binCount | 0.2907705 | 0.066646995 | 0.487964942 |
| wavelet-HHL_glrlm_LowGrayLevelRunEmphasis_32_binCount | 0.0521747 | -0.1810226 | 0.280135674 |
| wavelet-HHL_glrlm_RunEntropy_32_binCount | 0.2120993 | -0.02297573 | 0.424276568 |
| wavelet-HHL_glrlm_RunLengthNonUniformity_32_binCount | 0.6478304 | 0.489084233 | 0.764955367 |
| wavelet-HHL_glrlm_RunLengthNonUniformityNormalized_32_binCount | 0.5980373 | 0.424377902 | 0.729106937 |
| wavelet-HHL_glrlm_RunPercentage_32_binCount | 0.6246722 | 0.459747009 | 0.748034382 |
| wavelet-HHL_glrlm_RunVariance_32_binCount | 0.3293784 | 0.110292492 | 0.519243097 |
| wavelet-HHL_glrlm_ShortRunEmphasis_32_binCount | 0.6170847 | 0.448814968 | 0.742942828 |
| wavelet-HHL_glrlm_ShortRunHighGrayLevelEmphasis_32_binCount | 0.2413855 | 0.008336849 | 0.449041975 |
| wavelet-HHL_glrlm_ShortRunLowGrayLevelEmphasis_32_binCount | 0.1793845 | -0.05338116 | 0.394107244 |
| wavelet-HHL_glszm_GrayLevelNonUniformity_32_binCount | 0.4628605 | 0.260722035 | 0.626604071 |
| wavelet-HHL_glszm_GrayLevelNonUniformityNormalized_32_binCount | 0.275332 | 0.044620052 | 0.477638324 |
| wavelet-HHL_glszm_GrayLevelVariance_32_binCount | 0.3959302 | 0.179221229 | 0.575627679 |
| wavelet-HHL_glszm_HighGrayLevelZoneEmphasis_32_binCount | 0.089789 | -0.14759027 | 0.316525637 |
| wavelet-HHL_glszm_LargeAreaEmphasis_32_binCount | 0.8552415 | 0.777622345 | 0.907193518 |
| wavelet-HHL_glszm_LargeAreaHighGrayLevelEmphasis_32_binCount | 0.7261365 | 0.593947622 | 0.820075543 |
| wavelet-HHL_glszm_LargeAreaLowGrayLevelEmphasis_32_binCount | 0.7809977 | 0.670525839 | 0.857582194 |
| wavelet-HHL_glszm_LowGrayLevelZoneEmphasis_32_binCount | 0.2060092 | -0.02815514 | 0.41843462 |
| wavelet-HHL_glszm_SizeZoneNonUniformity_32_binCount | 0.4606235 | 0.254639045 | 0.626266367 |
| wavelet-HHL_glszm_SizeZoneNonUniformityNormalized_32_binCount | 0.341794 | 0.119863645 | 0.531327568 |
| wavelet-HHL_glszm_SmallAreaEmphasis_32_binCount | 0.3106333 | 0.084540321 | 0.50621673 |
| wavelet-HHL_glszm_SmallAreaHighGrayLevelEmphasis_32_binCount | -0.014075 | -0.24646053 | 0.219342425 |
| wavelet-HHL_glszm_SmallAreaLowGrayLevelEmphasis_32_binCount | 0.240222 | 0.00657141 | 0.448317564 |
| wavelet-HHL_glszm_ZoneEntropy_32_binCount | 0.4663301 | 0.261409636 | 0.630668858 |
| wavelet-HHL_glszm_ZonePercentage_32_binCount | 0.4076634 | 0.192753389 | 0.584898124 |
| wavelet-HHL_glszm_ZoneVariance_32_binCount | 0.8560857 | 0.77887245 | 0.907747794 |
| wavelet-HHL_gldm_DependenceEntropy_32_binCount | 0.5092048 | 0.316496826 | 0.66202866 |
| wavelet-HHL_gldm_DependenceNonUniformity_32_binCount | 0.5425715 | 0.357290136 | 0.687192351 |
| wavelet-HHL_gldm_DependenceNonUniformityNormalized_32_binCount | 0.1594872 | -0.0719628 | 0.375677782 |
| wavelet-HHL_gldm_DependenceVariance_32_binCount | 0.4951832 | 0.299313455 | 0.65144891 |
| wavelet-HHL_gldm_GrayLevelNonUniformity_32_binCount | 0.9265087 | 0.883730584 | 0.953780212 |
| wavelet-HHL_gldm_GrayLevelVariance_32_binCount | 0.3036378 | 0.074917944 | 0.501359317 |
| wavelet-HHL_gldm_HighGrayLevelEmphasis_32_binCount | 0.0909507 | -0.14696265 | 0.317873263 |
| wavelet-HHL_gldm_LargeDependenceEmphasis_32_binCount | 0.6134563 | 0.44603157 | 0.739651624 |
| wavelet-HHL_gldm_LargeDependenceHighGrayLevelEmphasis_32_binCount | 0.4210591 | 0.210096067 | 0.594674088 |
| wavelet-HHL_gldm_LargeDependenceLowGrayLevelEmphasis_32_binCount | 0.4451482 | 0.238219545 | 0.613519783 |
| wavelet-HHL_gldm_LowGrayLevelEmphasis_32_binCount | 0.0309216 | -0.20156767 | 0.260421567 |
| wavelet-HHL_gldm_SmallDependenceEmphasis_32_binCount | 0.4245565 | 0.212237406 | 0.598224711 |
| wavelet-HHL_gldm_SmallDependenceHighGrayLevelEmphasis_32_binCount | 0.3533455 | 0.130323392 | 0.541794298 |
| wavelet-HHL_gldm_SmallDependenceLowGrayLevelEmphasis_32_binCount | 0.4576207 | 0.252063063 | 0.623550012 |
| wavelet-HHL_ngtdm_Busyness_32_binCount | 0.7123857 | 0.575140025 | 0.810540539 |
| wavelet-HHL_ngtdm_Coarseness_32_binCount | 0.7990047 | 0.695843938 | 0.869817627 |
| wavelet-HHL_ngtdm_Complexity_32_binCount | 0.2732814 | 0.041738794 | 0.476244841 |
| wavelet-HHL_ngtdm_Contrast_32_binCount | 0.2881315 | 0.058171302 | 0.488446972 |
| wavelet-HHL_ngtdm_Strength_32_binCount | 0.678815 | 0.530794061 | 0.786723192 |
| wavelet-HHH_firstorder_10Percentile_32_binCount | 0.5717898 | 0.392854779 | 0.709228222 |
| wavelet-HHH_firstorder_90Percentile_32_binCount | 0.5800681 | 0.403392943 | 0.715289301 |
| wavelet-HHH_firstorder_Energy_32_binCount | 0.5698467 | 0.390454324 | 0.707778296 |
| wavelet-HHH_firstorder_Entropy_32_binCount | 0.4781953 | 0.275790665 | 0.639694581 |
| wavelet-HHH_firstorder_InterquartileRange_32_binCount | 0.5151308 | 0.322790141 | 0.666874085 |
| wavelet-HHH_firstorder_Kurtosis_32_binCount | 0.5216072 | 0.328386691 | 0.672647321 |
| wavelet-HHH_firstorder_Maximum_32_binCount | 0.7250765 | 0.592555299 | 0.819322702 |
| wavelet-HHH_firstorder_MeanAbsoluteDeviation_32_binCount | 0.7350592 | 0.607309662 | 0.825897387 |
| wavelet-HHH_firstorder_Mean_32_binCount | 0.1143104 | -0.12132734 | 0.337565303 |
| wavelet-HHH_firstorder_Median_32_binCount | -0.176265 | -0.39142196 | 0.057469147 |
| wavelet-HHH_firstorder_Minimum_32_binCount | 0.7993436 | 0.697004979 | 0.869847122 |
| wavelet-HHH_firstorder_Range_32_binCount | 0.7858073 | 0.677476423 | 0.860795393 |
| wavelet-HHH_firstorder_RobustMeanAbsoluteDeviation_32_binCount | 0.5348813 | 0.34689775 | 0.681780264 |
| wavelet-HHH_firstorder_RootMeanSquared_32_binCount | 0.7957856 | 0.691887705 | 0.86746344 |
| wavelet-HHH_firstorder_Skewness_32_binCount | 0.1009619 | -0.13173612 | 0.323908333 |
| wavelet-HHH_firstorder_TotalEnergy_32_binCount | 0.5698467 | 0.390454324 | 0.707778296 |
| wavelet-HHH_firstorder_Uniformity_32_binCount | 0.525627 | 0.333976943 | 0.675419009 |
| wavelet-HHH_firstorder_Variance_32_binCount | 0.7279124 | 0.596849251 | 0.821158373 |
| wavelet-HHH_glcm_Autocorrelation_32_binCount | 0.0942776 | -0.14268525 | 0.320324628 |
| wavelet-HHH_glcm_ClusterProminence_32_binCount | 0.2990204 | 0.070760906 | 0.497128846 |
| wavelet-HHH_glcm_ClusterShade_32_binCount | 0.056446 | -0.18058876 | 0.286267981 |
| wavelet-HHH_glcm_ClusterTendency_32_binCount | 0.3179796 | 0.091274131 | 0.512878835 |
| wavelet-HHH_glcm_Contrast_32_binCount | 0.3590623 | 0.137203556 | 0.546204726 |
| wavelet-HHH_glcm_Correlation_32_binCount | 0.4968736 | 0.298423 | 0.65388745 |
| wavelet-HHH_glcm_DifferenceAverage_32_binCount | 0.4102975 | 0.195684177 | 0.587023106 |
| wavelet-HHH_glcm_DifferenceEntropy_32_binCount | 0.4761398 | 0.273261445 | 0.638146579 |
| wavelet-HHH_glcm_DifferenceVariance_32_binCount | 0.4463848 | 0.237918503 | 0.615200332 |
| wavelet-HHH_glcm_Id_32_binCount | 0.5004037 | 0.302737771 | 0.656552757 |
| wavelet-HHH_glcm_Idm_32_binCount | 0.4906142 | 0.290780724 | 0.649156373 |
| wavelet-HHH_glcm_Idmn_32_binCount | 0.3493448 | 0.126194285 | 0.538401248 |
| wavelet-HHH_glcm_Idn_32_binCount | 0.4197723 | 0.206680985 | 0.594469164 |
| wavelet-HHH_glcm_Imc1_32_binCount | 0.3728928 | 0.153430607 | 0.557052665 |
| wavelet-HHH_glcm_Imc2_32_binCount | 0.5710972 | 0.390198846 | 0.709368665 |
| wavelet-HHH_glcm_InverseVariance_32_binCount | 0.3865682 | 0.170075218 | 0.567507513 |
| wavelet-HHH_glcm_JointAverage_32_binCount | 0.1190044 | -0.1179024 | 0.342478644 |
| wavelet-HHH_glcm_JointEnergy_32_binCount | 0.5317619 | 0.342075641 | 0.679813212 |
| wavelet-HHH_glcm_JointEntropy_32_binCount | 0.475237 | 0.272231226 | 0.637434657 |
| wavelet-HHH_glcm_MCC_32_binCount | 0.0898883 | -0.14853735 | 0.317208644 |
| wavelet-HHH_glcm_MaximumProbability_32_binCount | 0.5203969 | 0.327625354 | 0.671461823 |
| wavelet-HHH_glcm_SumAverage_32_binCount | 0.1190044 | -0.1179024 | 0.342478644 |
| wavelet-HHH_glcm_SumEntropy_32_binCount | 0.4980778 | 0.299910033 | 0.654790839 |
| wavelet-HHH_glcm_SumSquares_32_binCount | 0.3311622 | 0.1059075 | 0.523636901 |
| wavelet-HHH_glrlm_GrayLevelNonUniformity_32_binCount | 0.8475193 | 0.766527734 | 0.90202694 |
| wavelet-HHH_glrlm_GrayLevelNonUniformityNormalized_32_binCount | 0.4620751 | 0.257064218 | 0.627103721 |
| wavelet-HHH_glrlm_GrayLevelVariance_32_binCount | 0.3064519 | 0.078114054 | 0.50362687 |
| wavelet-HHH_glrlm_HighGrayLevelRunEmphasis_32_binCount | 0.1027236 | -0.13416529 | 0.327870239 |
| wavelet-HHH_glrlm_LongRunEmphasis_32_binCount | 0.6409216 | 0.47972439 | 0.760118224 |
| wavelet-HHH_glrlm_LongRunHighGrayLevelEmphasis_32_binCount | 0.6806602 | 0.532386191 | 0.788311357 |
| wavelet-HHH_glrlm_LongRunLowGrayLevelEmphasis_32_binCount | 0.4684128 | 0.263961463 | 0.632242737 |
| wavelet-HHH_glrlm_LowGrayLevelRunEmphasis_32_binCount | 0.1737928 | -0.06123599 | 0.390336419 |
| wavelet-HHH_glrlm_RunEntropy_32_binCount | 0.2014191 | -0.03458321 | 0.415319756 |
| wavelet-HHH_glrlm_RunLengthNonUniformity_32_binCount | 0.370169 | 0.15170436 | 0.554269889 |
| wavelet-HHH_glrlm_RunLengthNonUniformityNormalized_32_binCount | 0.533755 | 0.343415088 | 0.681727473 |
| wavelet-HHH_glrlm_RunPercentage_32_binCount | 0.5916573 | 0.416261443 | 0.724444055 |
| wavelet-HHH_glrlm_RunVariance_32_binCount | 0.7139177 | 0.577212889 | 0.811610378 |
| wavelet-HHH_glrlm_ShortRunEmphasis_32_binCount | 0.5459752 | 0.358819691 | 0.690746252 |
| wavelet-HHH_glrlm_ShortRunHighGrayLevelEmphasis_32_binCount | 0.2498587 | 0.017066738 | 0.456351805 |
| wavelet-HHH_glrlm_ShortRunLowGrayLevelEmphasis_32_binCount | 0.25697 | 0.026490434 | 0.461455212 |
| wavelet-HHH_glszm_GrayLevelNonUniformity_32_binCount | 0.5662923 | 0.386592593 | 0.704928766 |
| wavelet-HHH_glszm_GrayLevelNonUniformityNormalized_32_binCount | 0.3395523 | 0.11476865 | 0.530678692 |
| wavelet-HHH_glszm_GrayLevelVariance_32_binCount | 0.3415413 | 0.117406008 | 0.532105337 |
| wavelet-HHH_glszm_HighGrayLevelZoneEmphasis_32_binCount | 0.0816073 | -0.15538968 | 0.308944634 |
| wavelet-HHH_glszm_LargeAreaEmphasis_32_binCount | 0.7662471 | 0.649507501 | 0.847635864 |
| wavelet-HHH_glszm_LargeAreaHighGrayLevelEmphasis_32_binCount | 0.6411112 | 0.480610823 | 0.760036746 |
| wavelet-HHH_glszm_LargeAreaLowGrayLevelEmphasis_32_binCount | 0.8343613 | 0.747364283 | 0.893293553 |
| wavelet-HHH_glszm_LowGrayLevelZoneEmphasis_32_binCount | 0.2660437 | 0.038119582 | 0.468160131 |
| wavelet-HHH_glszm_SizeZoneNonUniformity_32_binCount | 0.4026697 | 0.189114449 | 0.580048294 |
| wavelet-HHH_glszm_SizeZoneNonUniformityNormalized_32_binCount | 0.3220138 | 0.095233839 | 0.516410087 |
| wavelet-HHH_glszm_SmallAreaEmphasis_32_binCount | 0.3203473 | 0.093357748 | 0.515061928 |
| wavelet-HHH_glszm_SmallAreaHighGrayLevelEmphasis_32_binCount | 0.1274625 | -0.10945754 | 0.350055541 |
| wavelet-HHH_glszm_SmallAreaLowGrayLevelEmphasis_32_binCount | 0.3376573 | 0.117791236 | 0.526798697 |
| wavelet-HHH_glszm_ZoneEntropy_32_binCount | 0.1179718 | -0.11914018 | 0.341667707 |
| wavelet-HHH_glszm_ZonePercentage_32_binCount | 0.4449638 | 0.236034131 | 0.61418316 |
| wavelet-HHH_glszm_ZoneVariance_32_binCount | 0.7661595 | 0.649383982 | 0.847576429 |
| wavelet-HHH_gldm_DependenceEntropy_32_binCount | 0.5065604 | 0.310838848 | 0.660977041 |
| wavelet-HHH_gldm_DependenceNonUniformity_32_binCount | 0.7071591 | 0.568878212 | 0.806630907 |
| wavelet-HHH_gldm_DependenceNonUniformityNormalized_32_binCount | 0.4623793 | 0.256694364 | 0.627632294 |
| wavelet-HHH_gldm_DependenceVariance_32_binCount | 0.4956621 | 0.29696069 | 0.652965442 |
| wavelet-HHH_gldm_GrayLevelNonUniformity_32_binCount | 0.9103318 | 0.86005756 | 0.943109722 |
| wavelet-HHH_gldm_GrayLevelVariance_32_binCount | 0.3334192 | 0.108222096 | 0.52556357 |
| wavelet-HHH_gldm_HighGrayLevelEmphasis_32_binCount | 0.0978855 | -0.13907041 | 0.323563032 |
| wavelet-HHH_gldm_LargeDependenceEmphasis_32_binCount | 0.5961657 | 0.422253683 | 0.727648251 |
| wavelet-HHH_gldm_LargeDependenceHighGrayLevelEmphasis_32_binCount | 0.5472948 | 0.361281955 | 0.691422383 |
| wavelet-HHH_gldm_LargeDependenceLowGrayLevelEmphasis_32_binCount | 0.370891 | 0.15016289 | 0.55588574 |
| wavelet-HHH_gldm_LowGrayLevelEmphasis_32_binCount | 0.1641976 | -0.07093217 | 0.381858254 |
| wavelet-HHH_gldm_SmallDependenceEmphasis_32_binCount | 0.4441582 | 0.235078255 | 0.613560499 |
| wavelet-HHH_gldm_SmallDependenceHighGrayLevelEmphasis_32_binCount | 0.4124453 | 0.197984029 | 0.588792815 |
| wavelet-HHH_gldm_SmallDependenceLowGrayLevelEmphasis_32_binCount | 0.4683099 | 0.265695621 | 0.631418904 |
| wavelet-HHH_ngtdm_Busyness_32_binCount | 0.3738709 | 0.153578927 | 0.558255887 |
| wavelet-HHH_ngtdm_Coarseness_32_binCount | 0.7799617 | 0.669755533 | 0.856672061 |
| wavelet-HHH_ngtdm_Complexity_32_binCount | 0.2277604 | -0.00702668 | 0.437928666 |
| wavelet-HHH_ngtdm_Contrast_32_binCount | 0.3727933 | 0.153019462 | 0.55710366 |
| wavelet-HHH_ngtdm_Strength_32_binCount | 0.6808528 | 0.534185882 | 0.787938838 |
| wavelet-LLL_firstorder_10Percentile_32_binCount | 0.2858821 | 0.058533594 | 0.485242789 |
| wavelet-LLL_firstorder_90Percentile_32_binCount | 0.9501536 | 0.921313415 | 0.968607027 |
| wavelet-LLL_firstorder_Energy_32_binCount | 0.9946452 | 0.991427254 | 0.996657476 |
| wavelet-LLL_firstorder_Entropy_32_binCount | 0.576222 | 0.398868041 | 0.712337574 |
| wavelet-LLL_firstorder_InterquartileRange_32_binCount | 0.519411 | 0.328250533 | 0.670015558 |
| wavelet-LLL_firstorder_Kurtosis_32_binCount | 0.7112439 | 0.573862569 | 0.809658115 |
| wavelet-LLL_firstorder_Maximum_32_binCount | 0.6860955 | 0.540737 | 0.791786868 |
| wavelet-LLL_firstorder_MeanAbsoluteDeviation_32_binCount | 0.6696113 | 0.51924818 | 0.779978258 |
| wavelet-LLL_firstorder_Mean_32_binCount | 0.8983794 | 0.841909449 | 0.935391651 |
| wavelet-LLL_firstorder_Median_32_binCount | 0.9540978 | 0.927385871 | 0.971130127 |
| wavelet-LLL_firstorder_Minimum_32_binCount | 0.5944894 | 0.420182546 | 0.726401108 |
| wavelet-LLL_firstorder_Range_32_binCount | 0.7017009 | 0.561713247 | 0.802745145 |
| wavelet-LLL_firstorder_RobustMeanAbsoluteDeviation_32_binCount | 0.4998768 | 0.304820884 | 0.655088318 |
| wavelet-LLL_firstorder_RootMeanSquared_32_binCount | 0.9367987 | 0.90051943 | 0.960122238 |
| wavelet-LLL_firstorder_Skewness_32_binCount | 0.5705918 | 0.389698762 | 0.708946689 |
| wavelet-LLL_firstorder_TotalEnergy_32_binCount | 0.9946452 | 0.991427254 | 0.996657476 |
| wavelet-LLL_firstorder_Uniformity_32_binCount | 0.611236 | 0.441895547 | 0.738491874 |
| wavelet-LLL_firstorder_Variance_32_binCount | 0.7561835 | 0.636475457 | 0.840447487 |
| wavelet-LLL_glcm_Autocorrelation_32_binCount | 0.467859 | 0.265754262 | 0.630830951 |
| wavelet-LLL_glcm_ClusterProminence_32_binCount | 0.2734463 | 0.042855256 | 0.475933028 |
| wavelet-LLL_glcm_ClusterShade_32_binCount | 0.322767 | 0.098828902 | 0.515756845 |
| wavelet-LLL_glcm_ClusterTendency_32_binCount | 0.3923476 | 0.177130386 | 0.571914753 |
| wavelet-LLL_glcm_Contrast_32_binCount | 0.6753022 | 0.526827462 | 0.784002784 |
| wavelet-LLL_glcm_Correlation_32_binCount | 0.6121798 | 0.44238806 | 0.739428643 |
| wavelet-LLL_glcm_DifferenceAverage_32_binCount | 0.6455814 | 0.48776705 | 0.762790206 |
| wavelet-LLL_glcm_DifferenceEntropy_32_binCount | 0.6070523 | 0.438047532 | 0.734913489 |
| wavelet-LLL_glcm_DifferenceVariance_32_binCount | 0.680955 | 0.534276159 | 0.788026487 |
| wavelet-LLL_glcm_Id_32_binCount | 0.6324634 | 0.470658967 | 0.753370717 |
| wavelet-LLL_glcm_Idm_32_binCount | 0.6281254 | 0.465086108 | 0.750224106 |
| wavelet-LLL_glcm_Idmn_32_binCount | 0.6697918 | 0.519536027 | 0.78008958 |
| wavelet-LLL_glcm_Idn_32_binCount | 0.6412562 | 0.482139792 | 0.759680456 |
| wavelet-LLL_glcm_Imc1_32_binCount | 0.6382803 | 0.478266784 | 0.757540428 |
| wavelet-LLL_glcm_Imc2_32_binCount | 0.6719084 | 0.520934792 | 0.782059572 |
| wavelet-LLL_glcm_InverseVariance_32_binCount | 0.4214121 | 0.213772986 | 0.593560551 |
| wavelet-LLL_glcm_JointAverage_32_binCount | 0.4836142 | 0.285593461 | 0.642527713 |
| wavelet-LLL_glcm_JointEnergy_32_binCount | 0.5945832 | 0.419801716 | 0.726647346 |
| wavelet-LLL_glcm_JointEntropy_32_binCount | 0.56685 | 0.387146991 | 0.705395361 |
| wavelet-LLL_glcm_MCC_32_binCount | 0.5240611 | 0.334718217 | 0.673219967 |
| wavelet-LLL_glcm_MaximumProbability_32_binCount | 0.6034531 | 0.431457095 | 0.732996731 |
| wavelet-LLL_glcm_SumAverage_32_binCount | 0.4836142 | 0.285593461 | 0.642527713 |
| wavelet-LLL_glcm_SumEntropy_32_binCount | 0.561549 | 0.38009493 | 0.701623604 |
| wavelet-LLL_glcm_SumSquares_32_binCount | 0.4248303 | 0.214873699 | 0.59746982 |
| wavelet-LLL_glrlm_GrayLevelNonUniformity_32_binCount | 0.9426489 | 0.909266504 | 0.963930953 |
| wavelet-LLL_glrlm_GrayLevelNonUniformityNormalized_32_binCount | 0.5946885 | 0.421298362 | 0.726239066 |
| wavelet-LLL_glrlm_GrayLevelVariance_32_binCount | 0.4589494 | 0.256061572 | 0.623589133 |
| wavelet-LLL_glrlm_HighGrayLevelRunEmphasis_32_binCount | 0.4768648 | 0.277070299 | 0.637527846 |
| wavelet-LLL_glrlm_LongRunEmphasis_32_binCount | 0.6310706 | 0.46696068 | 0.753020147 |
| wavelet-LLL_glrlm_LongRunHighGrayLevelEmphasis_32_binCount | 0.3865045 | 0.17196627 | 0.566598958 |
| wavelet-LLL_glrlm_LongRunLowGrayLevelEmphasis_32_binCount | 0.4198506 | 0.20677882 | 0.594527733 |
| wavelet-LLL_glrlm_LowGrayLevelRunEmphasis_32_binCount | 0.2779602 | 0.056822642 | 0.475271007 |
| wavelet-LLL_glrlm_RunEntropy_32_binCount | 0.336757 | 0.119486952 | 0.524808962 |
| wavelet-LLL_glrlm_RunLengthNonUniformity_32_binCount | 0.741429 | 0.615311531 | 0.830533071 |
| wavelet-LLL_glrlm_RunLengthNonUniformityNormalized_32_binCount | 0.6778583 | 0.529894667 | 0.785922917 |
| wavelet-LLL_glrlm_RunPercentage_32_binCount | 0.6849591 | 0.538995547 | 0.791058853 |
| wavelet-LLL_glrlm_RunVariance_32_binCount | 0.6378422 | 0.476092283 | 0.757777901 |
| wavelet-LLL_glrlm_ShortRunEmphasis_32_binCount | 0.6584804 | 0.503811494 | 0.772309218 |
| wavelet-LLL_glrlm_ShortRunHighGrayLevelEmphasis_32_binCount | 0.5083505 | 0.315067838 | 0.661534478 |
| wavelet-LLL_glrlm_ShortRunLowGrayLevelEmphasis_32_binCount | 0.2263403 | 0.004668239 | 0.429951186 |
| wavelet-LLL_glszm_GrayLevelNonUniformity_32_binCount | 0.6488369 | 0.490167136 | 0.765754631 |
| wavelet-LLL_glszm_GrayLevelNonUniformityNormalized_32_binCount | 0.4440627 | 0.239780127 | 0.611488547 |
| wavelet-LLL_glszm_GrayLevelVariance_32_binCount | 0.3902439 | 0.179020462 | 0.568348213 |
| wavelet-LLL_glszm_HighGrayLevelZoneEmphasis_32_binCount | 0.4208661 | 0.212739027 | 0.593303208 |
| wavelet-LLL_glszm_LargeAreaEmphasis_32_binCount | 0.5853244 | 0.408873812 | 0.719573189 |
| wavelet-LLL_glszm_LargeAreaHighGrayLevelEmphasis_32_binCount | 0.8575875 | 0.780936916 | 0.908777981 |
| wavelet-LLL_glszm_LargeAreaLowGrayLevelEmphasis_32_binCount | 0.2685511 | 0.039332178 | 0.470988614 |
| wavelet-LLL_glszm_LowGrayLevelZoneEmphasis_32_binCount | 0.308626 | 0.090364254 | 0.500744424 |
| wavelet-LLL_glszm_SizeZoneNonUniformity_32_binCount | 0.4210483 | 0.212401658 | 0.593682039 |
| wavelet-LLL_glszm_SizeZoneNonUniformityNormalized_32_binCount | 0.4975116 | 0.302656601 | 0.653011482 |
| wavelet-LLL_glszm_SmallAreaEmphasis_32_binCount | 0.4783554 | 0.280033981 | 0.638193358 |
| wavelet-LLL_glszm_SmallAreaHighGrayLevelEmphasis_32_binCount | 0.439176 | 0.234394077 | 0.607532476 |
| wavelet-LLL_glszm_SmallAreaLowGrayLevelEmphasis_32_binCount | 0.1886198 | -0.03914687 | 0.399659581 |
| wavelet-LLL_glszm_ZoneEntropy_32_binCount | 0.5671109 | 0.386984243 | 0.705769635 |
| wavelet-LLL_glszm_ZonePercentage_32_binCount | 0.7228971 | 0.590640061 | 0.817475024 |
| wavelet-LLL_glszm_ZoneVariance_32_binCount | 0.5881757 | 0.412515608 | 0.721653876 |
| wavelet-LLL_gldm_DependenceEntropy_32_binCount | 0.477524 | 0.277251477 | 0.638278036 |
| wavelet-LLL_gldm_DependenceNonUniformity_32_binCount | 0.8350643 | 0.747866384 | 0.893908078 |
| wavelet-LLL_gldm_DependenceNonUniformityNormalized_32_binCount | 0.7064133 | 0.56837608 | 0.805946134 |
| wavelet-LLL_gldm_DependenceVariance_32_binCount | 0.7259104 | 0.594387011 | 0.819683373 |
| wavelet-LLL_gldm_GrayLevelNonUniformity_32_binCount | 0.882284 | 0.81761503 | 0.924957455 |
| wavelet-LLL_gldm_GrayLevelVariance_32_binCount | 0.4622533 | 0.259542783 | 0.626322438 |
| wavelet-LLL_gldm_HighGrayLevelEmphasis_32_binCount | 0.4781581 | 0.278489968 | 0.638571991 |
| wavelet-LLL_gldm_LargeDependenceEmphasis_32_binCount | 0.6683297 | 0.516210931 | 0.779515694 |
| wavelet-LLL_gldm_LargeDependenceHighGrayLevelEmphasis_32_binCount | 0.6011302 | 0.428492536 | 0.731303193 |
| wavelet-LLL_gldm_LargeDependenceLowGrayLevelEmphasis_32_binCount | 0.4475145 | 0.239928611 | 0.615800076 |
| wavelet-LLL_gldm_LowGrayLevelEmphasis_32_binCount | 0.2717482 | 0.050117306 | 0.470055513 |
| wavelet-LLL_gldm_SmallDependenceEmphasis_32_binCount | 0.7131945 | 0.577581378 | 0.810675244 |
| wavelet-LLL_gldm_SmallDependenceHighGrayLevelEmphasis_32_binCount | 0.6888959 | 0.544905417 | 0.793620471 |
| wavelet-LLL_gldm_SmallDependenceLowGrayLevelEmphasis_32_binCount | 0.514207 | 0.319709858 | 0.666928777 |
| wavelet-LLL_ngtdm_Busyness_32_binCount | 0.8813853 | 0.816198908 | 0.924390453 |
| wavelet-LLL_ngtdm_Coarseness_32_binCount | 0.8517226 | 0.772303674 | 0.904913012 |
| wavelet-LLL_ngtdm_Complexity_32_binCount | 0.4956017 | 0.299564724 | 0.651868568 |
| wavelet-LLL_ngtdm_Contrast_32_binCount | 0.6528916 | 0.496827463 | 0.768198534 |
| wavelet-LLL_ngtdm_Strength_32_binCount | 0.73623 | 0.608196348 | 0.826932404 |

Table S5 The image feature ICC (1,1) for every single features.

|  | ICC value | Lower 95% CI | Higher 95% CI |
| --- | --- | --- | --- |
| original_shape_Elongation | 0.953 | 0.937 | 0.967 |
| original_shape_Flatness | 0.963 | 0.95 | 0.974 |
| original_shape_LeastAxisLength | 0.995 | 0.993 | 0.996 |
| original_shape_MajorAxisLength | 0.996 | 0.995 | 0.997 |
| original_shape_Maximum2DDiameterColumn | 0.991 | 0.988 | 0.994 |
| original_shape_Maximum2DDiameterRow | 0.989 | 0.986 | 0.992 |
| original_shape_Maximum2DDiameterSlice | 0.996 | 0.995 | 0.997 |
| original_shape_Maximum3DDiameter | 0.998 | 0.997 | 0.998 |
| original_shape_MeshVolume | 0.997 | 0.996 | 0.998 |
| original_shape_MinorAxisLength | 0.995 | 0.994 | 0.997 |
| original_shape_Sphericity | 0.994 | 0.991 | 0.995 |
| original_shape_SurfaceArea | 0.999 | 0.999 | 0.999 |
| original_shape_SurfaceVolumeRatio | 0.988 | 0.984 | 0.992 |
| original_shape_VoxelVolume | 0.997 | 0.996 | 0.998 |
| original_firstorder_10Percentile_32_binCount | 0.957 | 0.942 | 0.969 |
| original_firstorder_90Percentile_32_binCount | 0.896 | 0.863 | 0.925 |
| original_firstorder_Energy_32_binCount | 0.996 | 0.994 | 0.997 |
| original_firstorder_Entropy_32_binCount | 0.805 | 0.752 | 0.855 |
| original_firstorder_InterquartileRange_32_binCount | 0.9 | 0.869 | 0.928 |
| original_firstorder_Kurtosis_32_binCount | 0.631 | 0.557 | 0.711 |
| original_firstorder_Maximum_32_binCount | 0.801 | 0.748 | 0.852 |
| original_firstorder_MeanAbsoluteDeviation_32_binCount | 0.853 | 0.81 | 0.892 |
| original_firstorder_Mean_32_binCount | 0.952 | 0.936 | 0.966 |
| original_firstorder_Median_32_binCount | 0.972 | 0.962 | 0.98 |
| original_firstorder_Minimum_32_binCount | 0.812 | 0.76 | 0.86 |
| original_firstorder_Range_32_binCount | 0.828 | 0.78 | 0.873 |
| original_firstorder_RobustMeanAbsoluteDeviation_32_binCount | 0.89 | 0.857 | 0.921 |
| original_firstorder_RootMeanSquared_32_binCount | 0.934 | 0.913 | 0.953 |
| original_firstorder_Skewness_32_binCount | 0.674 | 0.603 | 0.748 |
| original_firstorder_TotalEnergy_32_binCount | 0.996 | 0.994 | 0.997 |
| original_firstorder_Uniformity_32_binCount | 0.812 | 0.76 | 0.86 |
| original_firstorder_Variance_32_binCount | 0.729 | 0.664 | 0.794 |
| original_glcm_Autocorrelation_32_binCount | 0.611 | 0.535 | 0.692 |
| original_glcm_ClusterProminence_32_binCount | 0.593 | 0.516 | 0.677 |
| original_glcm_ClusterShade_32_binCount | 0.563 | 0.486 | 0.649 |
| original_glcm_ClusterTendency_32_binCount | 0.736 | 0.672 | 0.799 |
| original_glcm_Contrast_32_binCount | 0.777 | 0.719 | 0.833 |
| original_glcm_Correlation_32_binCount | 0.742 | 0.678 | 0.804 |
| original_glcm_DifferenceAverage_32_binCount | 0.815 | 0.764 | 0.863 |
| original_glcm_DifferenceEntropy_32_binCount | 0.815 | 0.764 | 0.863 |
| original_glcm_DifferenceVariance_32_binCount | 0.768 | 0.708 | 0.826 |
| original_glcm_Id_32_binCount | 0.828 | 0.78 | 0.873 |
| original_glcm_Idm_32_binCount | 0.826 | 0.778 | 0.872 |
| original_glcm_Idmn_32_binCount | 0.781 | 0.724 | 0.836 |
| original_glcm_Idn_32_binCount | 0.818 | 0.767 | 0.865 |
| original_glcm_Imc1_32_binCount | 0.834 | 0.787 | 0.878 |
| original_glcm_Imc2_32_binCount | 0.857 | 0.816 | 0.896 |
| original_glcm_InverseVariance_32_binCount | 0.669 | 0.597 | 0.743 |
| original_glcm_JointAverage_32_binCount | 0.606 | 0.53 | 0.688 |
| original_glcm_JointEnergy_32_binCount | 0.798 | 0.744 | 0.85 |
| original_glcm_JointEntropy_32_binCount | 0.791 | 0.735 | 0.844 |
| original_glcm_MCC_32_binCount | 0.646 | 0.573 | 0.724 |
| original_glcm_MaximumProbability_32_binCount | 0.787 | 0.731 | 0.841 |
| original_glcm_SumAverage_32_binCount | 0.606 | 0.53 | 0.688 |
| original_glcm_SumEntropy_32_binCount | 0.795 | 0.741 | 0.848 |
| original_glcm_SumSquares_32_binCount | 0.746 | 0.684 | 0.808 |
| original_glrlm_GrayLevelNonUniformity_32_binCount | 0.985 | 0.98 | 0.99 |
| original_glrlm_GrayLevelNonUniformityNormalized_32_binCount | 0.796 | 0.742 | 0.848 |
| original_glrlm_GrayLevelVariance_32_binCount | 0.756 | 0.695 | 0.816 |
| original_glrlm_HighGrayLevelRunEmphasis_32_binCount | 0.608 | 0.532 | 0.69 |
| original_glrlm_LongRunEmphasis_32_binCount | 0.829 | 0.781 | 0.874 |
| original_glrlm_LongRunHighGrayLevelEmphasis_32_binCount | 0.58 | 0.503 | 0.664 |
| original_glrlm_LongRunLowGrayLevelEmphasis_32_binCount | 0.414 | 0.34 | 0.505 |
| original_glrlm_LowGrayLevelRunEmphasis_32_binCount | 0.353 | 0.283 | 0.441 |
| original_glrlm_RunEntropy_32_binCount | 0.68 | 0.609 | 0.753 |
| original_glrlm_RunLengthNonUniformity_32_binCount | 0.916 | 0.89 | 0.94 |
| original_glrlm_RunLengthNonUniformityNormalized_32_binCount | 0.864 | 0.824 | 0.901 |
| original_glrlm_RunPercentage_32_binCount | 0.868 | 0.829 | 0.904 |
| original_glrlm_RunVariance_32_binCount | 0.808 | 0.756 | 0.858 |
| original_glrlm_ShortRunEmphasis_32_binCount | 0.859 | 0.818 | 0.897 |
| original_glrlm_ShortRunHighGrayLevelEmphasis_32_binCount | 0.636 | 0.562 | 0.715 |
| original_glrlm_ShortRunLowGrayLevelEmphasis_32_binCount | 0.417 | 0.342 | 0.508 |
| original_glszm_GrayLevelNonUniformity_32_binCount | 0.921 | 0.896 | 0.944 |
| original_glszm_GrayLevelNonUniformityNormalized_32_binCount | 0.654 | 0.581 | 0.731 |
| original_glszm_GrayLevelVariance_32_binCount | 0.655 | 0.582 | 0.731 |
| original_glszm_HighGrayLevelZoneEmphasis_32_binCount | 0.581 | 0.504 | 0.666 |
| original_glszm_LargeAreaEmphasis_32_binCount | 0.811 | 0.76 | 0.86 |
| original_glszm_LargeAreaHighGrayLevelEmphasis_32_binCount | 0.906 | 0.877 | 0.933 |
| original_glszm_LargeAreaLowGrayLevelEmphasis_32_binCount | 0.419 | 0.344 | 0.51 |
| original_glszm_LowGrayLevelZoneEmphasis_32_binCount | 0.439 | 0.363 | 0.53 |
| original_glszm_SizeZoneNonUniformity_32_binCount | 0.613 | 0.537 | 0.694 |
| original_glszm_SizeZoneNonUniformityNormalized_32_binCount | 0.738 | 0.675 | 0.802 |
| original_glszm_SmallAreaEmphasis_32_binCount | 0.727 | 0.662 | 0.792 |
| original_glszm_SmallAreaHighGrayLevelEmphasis_32_binCount | 0.608 | 0.532 | 0.69 |
| original_glszm_SmallAreaLowGrayLevelEmphasis_32_binCount | 0.408 | 0.334 | 0.499 |
| original_glszm_ZoneEntropy_32_binCount | 0.803 | 0.75 | 0.853 |
| original_glszm_ZonePercentage_32_binCount | 0.882 | 0.846 | 0.914 |
| original_glszm_ZoneVariance_32_binCount | 0.812 | 0.761 | 0.861 |
| original_gldm_DependenceEntropy_32_binCount | 0.802 | 0.749 | 0.853 |
| original_gldm_DependenceNonUniformity_32_binCount | 0.971 | 0.961 | 0.979 |
| original_gldm_DependenceNonUniformityNormalized_32_binCount | 0.861 | 0.82 | 0.899 |
| original_gldm_DependenceVariance_32_binCount | 0.857 | 0.815 | 0.895 |
| original_gldm_GrayLevelNonUniformity_32_binCount | 0.95 | 0.933 | 0.964 |
| original_gldm_GrayLevelVariance_32_binCount | 0.76 | 0.699 | 0.819 |
| original_gldm_HighGrayLevelEmphasis_32_binCount | 0.612 | 0.536 | 0.693 |
| original_gldm_LargeDependenceEmphasis_32_binCount | 0.863 | 0.822 | 0.9 |
| original_gldm_LargeDependenceHighGrayLevelEmphasis_32_binCount | 0.688 | 0.618 | 0.76 |
| original_gldm_LargeDependenceLowGrayLevelEmphasis_32_binCount | 0.33 | 0.263 | 0.417 |
| original_gldm_LowGrayLevelEmphasis_32_binCount | 0.324 | 0.257 | 0.41 |
| original_gldm_SmallDependenceEmphasis_32_binCount | 0.865 | 0.825 | 0.902 |
| original_gldm_SmallDependenceHighGrayLevelEmphasis_32_binCount | 0.772 | 0.713 | 0.829 |
| original_gldm_SmallDependenceLowGrayLevelEmphasis_32_binCount | 0.794 | 0.739 | 0.846 |
| original_ngtdm_Busyness_32_binCount | 0.802 | 0.749 | 0.853 |
| original_ngtdm_Coarseness_32_binCount | 0.964 | 0.952 | 0.975 |
| original_ngtdm_Complexity_32_binCount | 0.696 | 0.627 | 0.766 |
| original_ngtdm_Contrast_32_binCount | 0.802 | 0.749 | 0.853 |
| original_ngtdm_Strength_32_binCount | 0.896 | 0.863 | 0.925 |
| log-sigma-1-mm-3D_firstorder_10Percentile_32_binCount | 0.777 | 0.72 | 0.833 |
| log-sigma-1-mm-3D_firstorder_90Percentile_32_binCount | 0.901 | 0.87 | 0.929 |
| log-sigma-1-mm-3D_firstorder_Energy_32_binCount | 0.956 | 0.941 | 0.968 |
| log-sigma-1-mm-3D_firstorder_Entropy_32_binCount | 0.83 | 0.783 | 0.875 |
| log-sigma-1-mm-3D_firstorder_InterquartileRange_32_binCount | 0.919 | 0.893 | 0.942 |
| log-sigma-1-mm-3D_firstorder_Kurtosis_32_binCount | 0.492 | 0.414 | 0.582 |
| log-sigma-1-mm-3D_firstorder_Maximum_32_binCount | 0.858 | 0.817 | 0.896 |
| log-sigma-1-mm-3D_firstorder_MeanAbsoluteDeviation_32_binCount | 0.894 | 0.862 | 0.924 |
| log-sigma-1-mm-3D_firstorder_Mean_32_binCount | 0.831 | 0.783 | 0.875 |
| log-sigma-1-mm-3D_firstorder_Median_32_binCount | 0.902 | 0.872 | 0.929 |
| log-sigma-1-mm-3D_firstorder_Minimum_32_binCount | 0.777 | 0.72 | 0.833 |
| log-sigma-1-mm-3D_firstorder_Range_32_binCount | 0.838 | 0.793 | 0.881 |
| log-sigma-1-mm-3D_firstorder_RobustMeanAbsoluteDeviation_32_binCount | 0.916 | 0.889 | 0.94 |
| log-sigma-1-mm-3D_firstorder_RootMeanSquared_32_binCount | 0.872 | 0.833 | 0.907 |
| log-sigma-1-mm-3D_firstorder_Skewness_32_binCount | 0.478 | 0.4 | 0.568 |
| log-sigma-1-mm-3D_firstorder_TotalEnergy_32_binCount | 0.956 | 0.941 | 0.968 |
| log-sigma-1-mm-3D_firstorder_Uniformity_32_binCount | 0.816 | 0.765 | 0.864 |
| log-sigma-1-mm-3D_firstorder_Variance_32_binCount | 0.77 | 0.711 | 0.827 |
| log-sigma-1-mm-3D_glcm_Autocorrelation_32_binCount | 0.483 | 0.405 | 0.573 |
| log-sigma-1-mm-3D_glcm_ClusterProminence_32_binCount | 0.686 | 0.616 | 0.758 |
| log-sigma-1-mm-3D_glcm_ClusterShade_32_binCount | 0.577 | 0.5 | 0.662 |
| log-sigma-1-mm-3D_glcm_ClusterTendency_32_binCount | 0.764 | 0.704 | 0.823 |
| log-sigma-1-mm-3D_glcm_Contrast_32_binCount | 0.806 | 0.754 | 0.856 |
| log-sigma-1-mm-3D_glcm_Correlation_32_binCount | 0.678 | 0.607 | 0.751 |
| log-sigma-1-mm-3D_glcm_DifferenceAverage_32_binCount | 0.829 | 0.782 | 0.874 |
| log-sigma-1-mm-3D_glcm_DifferenceEntropy_32_binCount | 0.831 | 0.783 | 0.875 |
| log-sigma-1-mm-3D_glcm_DifferenceVariance_32_binCount | 0.808 | 0.756 | 0.858 |
| log-sigma-1-mm-3D_glcm_Id_32_binCount | 0.837 | 0.791 | 0.88 |
| log-sigma-1-mm-3D_glcm_Idm_32_binCount | 0.836 | 0.789 | 0.879 |
| log-sigma-1-mm-3D_glcm_Idmn_32_binCount | 0.808 | 0.756 | 0.858 |
| log-sigma-1-mm-3D_glcm_Idn_32_binCount | 0.831 | 0.784 | 0.876 |
| log-sigma-1-mm-3D_glcm_Imc1_32_binCount | 0.882 | 0.847 | 0.915 |
| log-sigma-1-mm-3D_glcm_Imc2_32_binCount | 0.913 | 0.885 | 0.937 |
| log-sigma-1-mm-3D_glcm_InverseVariance_32_binCount | 0.665 | 0.593 | 0.74 |
| log-sigma-1-mm-3D_glcm_JointAverage_32_binCount | 0.504 | 0.426 | 0.594 |
| log-sigma-1-mm-3D_glcm_JointEnergy_32_binCount | 0.772 | 0.713 | 0.829 |
| log-sigma-1-mm-3D_glcm_JointEntropy_32_binCount | 0.819 | 0.769 | 0.866 |
| log-sigma-1-mm-3D_glcm_MCC_32_binCount | 0.608 | 0.532 | 0.69 |
| log-sigma-1-mm-3D_glcm_MaximumProbability_32_binCount | 0.78 | 0.723 | 0.835 |
| log-sigma-1-mm-3D_glcm_SumAverage_32_binCount | 0.504 | 0.426 | 0.594 |
| log-sigma-1-mm-3D_glcm_SumEntropy_32_binCount | 0.824 | 0.775 | 0.87 |
| log-sigma-1-mm-3D_glcm_SumSquares_32_binCount | 0.777 | 0.719 | 0.833 |
| log-sigma-1-mm-3D_glrlm_GrayLevelNonUniformity_32_binCount | 0.986 | 0.981 | 0.99 |
| log-sigma-1-mm-3D_glrlm_GrayLevelNonUniformityNormalized_32_binCount | 0.809 | 0.757 | 0.859 |
| log-sigma-1-mm-3D_glrlm_GrayLevelVariance_32_binCount | 0.793 | 0.738 | 0.846 |
| log-sigma-1-mm-3D_glrlm_HighGrayLevelRunEmphasis_32_binCount | 0.481 | 0.403 | 0.571 |
| log-sigma-1-mm-3D_glrlm_LongRunEmphasis_32_binCount | 0.798 | 0.744 | 0.85 |
| log-sigma-1-mm-3D_glrlm_LongRunHighGrayLevelEmphasis_32_binCount | 0.709 | 0.642 | 0.778 |
| log-sigma-1-mm-3D_glrlm_LongRunLowGrayLevelEmphasis_32_binCount | 0.528 | 0.45 | 0.617 |
| log-sigma-1-mm-3D_glrlm_LowGrayLevelRunEmphasis_32_binCount | 0.603 | 0.527 | 0.686 |
| log-sigma-1-mm-3D_glrlm_RunEntropy_32_binCount | 0.719 | 0.653 | 0.786 |
| log-sigma-1-mm-3D_glrlm_RunLengthNonUniformity_32_binCount | 0.913 | 0.885 | 0.937 |
| log-sigma-1-mm-3D_glrlm_RunLengthNonUniformityNormalized_32_binCount | 0.865 | 0.826 | 0.902 |
| log-sigma-1-mm-3D_glrlm_RunPercentage_32_binCount | 0.867 | 0.828 | 0.903 |
| log-sigma-1-mm-3D_glrlm_RunVariance_32_binCount | 0.766 | 0.706 | 0.824 |
| log-sigma-1-mm-3D_glrlm_ShortRunEmphasis_32_binCount | 0.857 | 0.815 | 0.896 |
| log-sigma-1-mm-3D_glrlm_ShortRunHighGrayLevelEmphasis_32_binCount | 0.418 | 0.344 | 0.509 |
| log-sigma-1-mm-3D_glrlm_ShortRunLowGrayLevelEmphasis_32_binCount | 0.626 | 0.551 | 0.706 |
| log-sigma-1-mm-3D_glszm_GrayLevelNonUniformity_32_binCount | 0.946 | 0.928 | 0.961 |
| log-sigma-1-mm-3D_glszm_GrayLevelNonUniformityNormalized_32_binCount | 0.699 | 0.63 | 0.769 |
| log-sigma-1-mm-3D_glszm_GrayLevelVariance_32_binCount | 0.744 | 0.681 | 0.806 |
| log-sigma-1-mm-3D_glszm_HighGrayLevelZoneEmphasis_32_binCount | 0.484 | 0.406 | 0.574 |
| log-sigma-1-mm-3D_glszm_LargeAreaEmphasis_32_binCount | 0.737 | 0.673 | 0.8 |
| log-sigma-1-mm-3D_glszm_LargeAreaHighGrayLevelEmphasis_32_binCount | 0.645 | 0.572 | 0.723 |
| log-sigma-1-mm-3D_glszm_LargeAreaLowGrayLevelEmphasis_32_binCount | 0.844 | 0.799 | 0.885 |
| log-sigma-1-mm-3D_glszm_LowGrayLevelZoneEmphasis_32_binCount | 0.555 | 0.478 | 0.642 |
| log-sigma-1-mm-3D_glszm_SizeZoneNonUniformity_32_binCount | 0.637 | 0.563 | 0.716 |
| log-sigma-1-mm-3D_glszm_SizeZoneNonUniformityNormalized_32_binCount | 0.777 | 0.719 | 0.833 |
| log-sigma-1-mm-3D_glszm_SmallAreaEmphasis_32_binCount | 0.77 | 0.712 | 0.828 |
| log-sigma-1-mm-3D_glszm_SmallAreaHighGrayLevelEmphasis_32_binCount | 0.428 | 0.353 | 0.519 |
| log-sigma-1-mm-3D_glszm_SmallAreaLowGrayLevelEmphasis_32_binCount | 0.527 | 0.449 | 0.615 |
| log-sigma-1-mm-3D_glszm_ZoneEntropy_32_binCount | 0.799 | 0.746 | 0.851 |
| log-sigma-1-mm-3D_glszm_ZonePercentage_32_binCount | 0.877 | 0.84 | 0.91 |
| log-sigma-1-mm-3D_glszm_ZoneVariance_32_binCount | 0.737 | 0.673 | 0.801 |
| log-sigma-1-mm-3D_gldm_DependenceEntropy_32_binCount | 0.771 | 0.713 | 0.828 |
| log-sigma-1-mm-3D_gldm_DependenceNonUniformity_32_binCount | 0.968 | 0.957 | 0.978 |
| log-sigma-1-mm-3D_gldm_DependenceNonUniformityNormalized_32_binCount | 0.857 | 0.815 | 0.895 |
| log-sigma-1-mm-3D_gldm_DependenceVariance_32_binCount | 0.855 | 0.813 | 0.894 |
| log-sigma-1-mm-3D_gldm_GrayLevelNonUniformity_32_binCount | 0.942 | 0.923 | 0.959 |
| log-sigma-1-mm-3D_gldm_GrayLevelVariance_32_binCount | 0.792 | 0.737 | 0.845 |
| log-sigma-1-mm-3D_gldm_HighGrayLevelEmphasis_32_binCount | 0.479 | 0.402 | 0.57 |
| log-sigma-1-mm-3D_gldm_LargeDependenceEmphasis_32_binCount | 0.854 | 0.812 | 0.893 |
| log-sigma-1-mm-3D_gldm_LargeDependenceHighGrayLevelEmphasis_32_binCount | 0.747 | 0.685 | 0.809 |
| log-sigma-1-mm-3D_gldm_LargeDependenceLowGrayLevelEmphasis_32_binCount | 0.546 | 0.468 | 0.634 |
| log-sigma-1-mm-3D_gldm_LowGrayLevelEmphasis_32_binCount | 0.601 | 0.524 | 0.683 |
| log-sigma-1-mm-3D_gldm_SmallDependenceEmphasis_32_binCount | 0.863 | 0.823 | 0.9 |
| log-sigma-1-mm-3D_gldm_SmallDependenceHighGrayLevelEmphasis_32_binCount | 0.811 | 0.76 | 0.86 |
| log-sigma-1-mm-3D_gldm_SmallDependenceLowGrayLevelEmphasis_32_binCount | 0.765 | 0.705 | 0.823 |
| log-sigma-1-mm-3D_ngtdm_Busyness_32_binCount | 0.964 | 0.952 | 0.975 |
| log-sigma-1-mm-3D_ngtdm_Coarseness_32_binCount | 0.973 | 0.964 | 0.981 |
| log-sigma-1-mm-3D_ngtdm_Complexity_32_binCount | 0.715 | 0.648 | 0.782 |
| log-sigma-1-mm-3D_ngtdm_Contrast_32_binCount | 0.819 | 0.769 | 0.866 |
| log-sigma-1-mm-3D_ngtdm_Strength_32_binCount | 0.914 | 0.886 | 0.938 |
| log-sigma-2-mm-3D_firstorder_10Percentile_32_binCount | 0.79 | 0.734 | 0.843 |
| log-sigma-2-mm-3D_firstorder_90Percentile_32_binCount | 0.952 | 0.936 | 0.966 |
| log-sigma-2-mm-3D_firstorder_Energy_32_binCount | 0.978 | 0.97 | 0.984 |
| log-sigma-2-mm-3D_firstorder_Entropy_32_binCount | 0.835 | 0.788 | 0.879 |
| log-sigma-2-mm-3D_firstorder_InterquartileRange_32_binCount | 0.869 | 0.83 | 0.905 |
| log-sigma-2-mm-3D_firstorder_Kurtosis_32_binCount | 0.6 | 0.523 | 0.683 |
| log-sigma-2-mm-3D_firstorder_Maximum_32_binCount | 0.882 | 0.847 | 0.915 |
| log-sigma-2-mm-3D_firstorder_MeanAbsoluteDeviation_32_binCount | 0.893 | 0.86 | 0.923 |
| log-sigma-2-mm-3D_firstorder_Mean_32_binCount | 0.903 | 0.873 | 0.93 |
| log-sigma-2-mm-3D_firstorder_Median_32_binCount | 0.937 | 0.916 | 0.955 |
| log-sigma-2-mm-3D_firstorder_Minimum_32_binCount | 0.791 | 0.736 | 0.845 |
| log-sigma-2-mm-3D_firstorder_Range_32_binCount | 0.86 | 0.819 | 0.898 |
| log-sigma-2-mm-3D_firstorder_RobustMeanAbsoluteDeviation_32_binCount | 0.878 | 0.841 | 0.912 |
| log-sigma-2-mm-3D_firstorder_RootMeanSquared_32_binCount | 0.936 | 0.915 | 0.955 |
| log-sigma-2-mm-3D_firstorder_Skewness_32_binCount | 0.577 | 0.499 | 0.662 |
| log-sigma-2-mm-3D_firstorder_TotalEnergy_32_binCount | 0.978 | 0.97 | 0.984 |
| log-sigma-2-mm-3D_firstorder_Uniformity_32_binCount | 0.845 | 0.801 | 0.887 |
| log-sigma-2-mm-3D_firstorder_Variance_32_binCount | 0.837 | 0.791 | 0.88 |
| log-sigma-2-mm-3D_glcm_Autocorrelation_32_binCount | 0.494 | 0.416 | 0.584 |
| log-sigma-2-mm-3D_glcm_ClusterProminence_32_binCount | 0.721 | 0.655 | 0.787 |
| log-sigma-2-mm-3D_glcm_ClusterShade_32_binCount | 0.7 | 0.632 | 0.77 |
| log-sigma-2-mm-3D_glcm_ClusterTendency_32_binCount | 0.778 | 0.721 | 0.834 |
| log-sigma-2-mm-3D_glcm_Contrast_32_binCount | 0.816 | 0.766 | 0.864 |
| log-sigma-2-mm-3D_glcm_Correlation_32_binCount | 0.63 | 0.555 | 0.71 |
| log-sigma-2-mm-3D_glcm_DifferenceAverage_32_binCount | 0.847 | 0.803 | 0.888 |
| log-sigma-2-mm-3D_glcm_DifferenceEntropy_32_binCount | 0.849 | 0.806 | 0.89 |
| log-sigma-2-mm-3D_glcm_DifferenceVariance_32_binCount | 0.801 | 0.748 | 0.852 |
| log-sigma-2-mm-3D_glcm_Id_32_binCount | 0.867 | 0.828 | 0.903 |
| log-sigma-2-mm-3D_glcm_Idm_32_binCount | 0.865 | 0.825 | 0.902 |
| log-sigma-2-mm-3D_glcm_Idmn_32_binCount | 0.82 | 0.77 | 0.867 |
| log-sigma-2-mm-3D_glcm_Idn_32_binCount | 0.85 | 0.807 | 0.89 |
| log-sigma-2-mm-3D_glcm_Imc1_32_binCount | 0.842 | 0.797 | 0.884 |
| log-sigma-2-mm-3D_glcm_Imc2_32_binCount | 0.832 | 0.784 | 0.876 |
| log-sigma-2-mm-3D_glcm_InverseVariance_32_binCount | 0.732 | 0.668 | 0.797 |
| log-sigma-2-mm-3D_glcm_JointAverage_32_binCount | 0.529 | 0.451 | 0.618 |
| log-sigma-2-mm-3D_glcm_JointEnergy_32_binCount | 0.833 | 0.786 | 0.877 |
| log-sigma-2-mm-3D_glcm_JointEntropy_32_binCount | 0.825 | 0.777 | 0.871 |
| log-sigma-2-mm-3D_glcm_MCC_32_binCount | 0.487 | 0.409 | 0.577 |
| log-sigma-2-mm-3D_glcm_MaximumProbability_32_binCount | 0.84 | 0.795 | 0.883 |
| log-sigma-2-mm-3D_glcm_SumAverage_32_binCount | 0.529 | 0.451 | 0.618 |
| log-sigma-2-mm-3D_glcm_SumEntropy_32_binCount | 0.822 | 0.772 | 0.868 |
| log-sigma-2-mm-3D_glcm_SumSquares_32_binCount | 0.787 | 0.731 | 0.841 |
| log-sigma-2-mm-3D_glrlm_GrayLevelNonUniformity_32_binCount | 0.988 | 0.984 | 0.992 |
| log-sigma-2-mm-3D_glrlm_GrayLevelNonUniformityNormalized_32_binCount | 0.83 | 0.783 | 0.875 |
| log-sigma-2-mm-3D_glrlm_GrayLevelVariance_32_binCount | 0.8 | 0.747 | 0.852 |
| log-sigma-2-mm-3D_glrlm_HighGrayLevelRunEmphasis_32_binCount | 0.488 | 0.41 | 0.578 |
| log-sigma-2-mm-3D_glrlm_LongRunEmphasis_32_binCount | 0.86 | 0.819 | 0.898 |
| log-sigma-2-mm-3D_glrlm_LongRunHighGrayLevelEmphasis_32_binCount | 0.759 | 0.699 | 0.819 |
| log-sigma-2-mm-3D_glrlm_LongRunLowGrayLevelEmphasis_32_binCount | 0.772 | 0.714 | 0.829 |
| log-sigma-2-mm-3D_glrlm_LowGrayLevelRunEmphasis_32_binCount | 0.807 | 0.755 | 0.857 |
| log-sigma-2-mm-3D_glrlm_RunEntropy_32_binCount | 0.659 | 0.586 | 0.735 |
| log-sigma-2-mm-3D_glrlm_RunLengthNonUniformity_32_binCount | 0.95 | 0.934 | 0.965 |
| log-sigma-2-mm-3D_glrlm_RunLengthNonUniformityNormalized_32_binCount | 0.897 | 0.865 | 0.926 |
| log-sigma-2-mm-3D_glrlm_RunPercentage_32_binCount | 0.903 | 0.872 | 0.93 |
| log-sigma-2-mm-3D_glrlm_RunVariance_32_binCount | 0.853 | 0.811 | 0.893 |
| log-sigma-2-mm-3D_glrlm_ShortRunEmphasis_32_binCount | 0.891 | 0.858 | 0.921 |
| log-sigma-2-mm-3D_glrlm_ShortRunHighGrayLevelEmphasis_32_binCount | 0.476 | 0.399 | 0.567 |
| log-sigma-2-mm-3D_glrlm_ShortRunLowGrayLevelEmphasis_32_binCount | 0.814 | 0.763 | 0.862 |
| log-sigma-2-mm-3D_glszm_GrayLevelNonUniformity_32_binCount | 0.946 | 0.928 | 0.962 |
| log-sigma-2-mm-3D_glszm_GrayLevelNonUniformityNormalized_32_binCount | 0.683 | 0.613 | 0.756 |
| log-sigma-2-mm-3D_glszm_GrayLevelVariance_32_binCount | 0.732 | 0.668 | 0.797 |
| log-sigma-2-mm-3D_glszm_HighGrayLevelZoneEmphasis_32_binCount | 0.423 | 0.348 | 0.514 |
| log-sigma-2-mm-3D_glszm_LargeAreaEmphasis_32_binCount | 0.803 | 0.749 | 0.853 |
| log-sigma-2-mm-3D_glszm_LargeAreaHighGrayLevelEmphasis_32_binCount | 0.708 | 0.64 | 0.777 |
| log-sigma-2-mm-3D_glszm_LargeAreaLowGrayLevelEmphasis_32_binCount | 0.861 | 0.82 | 0.898 |
| log-sigma-2-mm-3D_glszm_LowGrayLevelZoneEmphasis_32_binCount | 0.713 | 0.646 | 0.781 |
| log-sigma-2-mm-3D_glszm_SizeZoneNonUniformity_32_binCount | 0.632 | 0.557 | 0.711 |
| log-sigma-2-mm-3D_glszm_SizeZoneNonUniformityNormalized_32_binCount | 0.76 | 0.699 | 0.819 |
| log-sigma-2-mm-3D_glszm_SmallAreaEmphasis_32_binCount | 0.75 | 0.688 | 0.811 |
| log-sigma-2-mm-3D_glszm_SmallAreaHighGrayLevelEmphasis_32_binCount | 0.409 | 0.335 | 0.5 |
| log-sigma-2-mm-3D_glszm_SmallAreaLowGrayLevelEmphasis_32_binCount | 0.506 | 0.429 | 0.596 |
| log-sigma-2-mm-3D_glszm_ZoneEntropy_32_binCount | 0.867 | 0.828 | 0.903 |
| log-sigma-2-mm-3D_glszm_ZonePercentage_32_binCount | 0.9 | 0.87 | 0.928 |
| log-sigma-2-mm-3D_glszm_ZoneVariance_32_binCount | 0.803 | 0.75 | 0.854 |
| log-sigma-2-mm-3D_gldm_DependenceEntropy_32_binCount | 0.857 | 0.815 | 0.896 |
| log-sigma-2-mm-3D_gldm_DependenceNonUniformity_32_binCount | 0.98 | 0.973 | 0.986 |
| log-sigma-2-mm-3D_gldm_DependenceNonUniformityNormalized_32_binCount | 0.88 | 0.844 | 0.913 |
| log-sigma-2-mm-3D_gldm_DependenceVariance_32_binCount | 0.914 | 0.887 | 0.938 |
| log-sigma-2-mm-3D_gldm_GrayLevelNonUniformity_32_binCount | 0.961 | 0.948 | 0.972 |
| log-sigma-2-mm-3D_gldm_GrayLevelVariance_32_binCount | 0.802 | 0.749 | 0.853 |
| log-sigma-2-mm-3D_gldm_HighGrayLevelEmphasis_32_binCount | 0.49 | 0.412 | 0.58 |
| log-sigma-2-mm-3D_gldm_LargeDependenceEmphasis_32_binCount | 0.897 | 0.866 | 0.926 |
| log-sigma-2-mm-3D_gldm_LargeDependenceHighGrayLevelEmphasis_32_binCount | 0.795 | 0.741 | 0.847 |
| log-sigma-2-mm-3D_gldm_LargeDependenceLowGrayLevelEmphasis_32_binCount | 0.715 | 0.649 | 0.783 |
| log-sigma-2-mm-3D_gldm_LowGrayLevelEmphasis_32_binCount | 0.806 | 0.754 | 0.856 |
| log-sigma-2-mm-3D_gldm_SmallDependenceEmphasis_32_binCount | 0.887 | 0.852 | 0.918 |
| log-sigma-2-mm-3D_gldm_SmallDependenceHighGrayLevelEmphasis_32_binCount | 0.887 | 0.853 | 0.918 |
| log-sigma-2-mm-3D_gldm_SmallDependenceLowGrayLevelEmphasis_32_binCount | 0.789 | 0.733 | 0.843 |
| log-sigma-2-mm-3D_ngtdm_Busyness_32_binCount | 0.975 | 0.967 | 0.982 |
| log-sigma-2-mm-3D_ngtdm_Coarseness_32_binCount | 0.968 | 0.957 | 0.977 |
| log-sigma-2-mm-3D_ngtdm_Complexity_32_binCount | 0.753 | 0.691 | 0.814 |
| log-sigma-2-mm-3D_ngtdm_Contrast_32_binCount | 0.835 | 0.788 | 0.879 |
| log-sigma-2-mm-3D_ngtdm_Strength_32_binCount | 0.925 | 0.901 | 0.946 |
| log-sigma-3-mm-3D_firstorder_10Percentile_32_binCount | 0.836 | 0.789 | 0.879 |
| log-sigma-3-mm-3D_firstorder_90Percentile_32_binCount | 0.962 | 0.949 | 0.973 |
| log-sigma-3-mm-3D_firstorder_Energy_32_binCount | 0.987 | 0.982 | 0.991 |
| log-sigma-3-mm-3D_firstorder_Entropy_32_binCount | 0.828 | 0.78 | 0.874 |
| log-sigma-3-mm-3D_firstorder_InterquartileRange_32_binCount | 0.874 | 0.836 | 0.909 |
| log-sigma-3-mm-3D_firstorder_Kurtosis_32_binCount | 0.657 | 0.585 | 0.733 |
| log-sigma-3-mm-3D_firstorder_Maximum_32_binCount | 0.951 | 0.934 | 0.965 |
| log-sigma-3-mm-3D_firstorder_MeanAbsoluteDeviation_32_binCount | 0.891 | 0.857 | 0.921 |
| log-sigma-3-mm-3D_firstorder_Mean_32_binCount | 0.938 | 0.917 | 0.956 |
| log-sigma-3-mm-3D_firstorder_Median_32_binCount | 0.955 | 0.939 | 0.968 |
| log-sigma-3-mm-3D_firstorder_Minimum_32_binCount | 0.785 | 0.728 | 0.839 |
| log-sigma-3-mm-3D_firstorder_Range_32_binCount | 0.876 | 0.838 | 0.91 |
| log-sigma-3-mm-3D_firstorder_RobustMeanAbsoluteDeviation_32_binCount | 0.883 | 0.847 | 0.915 |
| log-sigma-3-mm-3D_firstorder_RootMeanSquared_32_binCount | 0.957 | 0.942 | 0.969 |
| log-sigma-3-mm-3D_firstorder_Skewness_32_binCount | 0.676 | 0.605 | 0.749 |
| log-sigma-3-mm-3D_firstorder_TotalEnergy_32_binCount | 0.987 | 0.982 | 0.991 |
| log-sigma-3-mm-3D_firstorder_Uniformity_32_binCount | 0.84 | 0.795 | 0.883 |
| log-sigma-3-mm-3D_firstorder_Variance_32_binCount | 0.845 | 0.8 | 0.886 |
| log-sigma-3-mm-3D_glcm_Autocorrelation_32_binCount | 0.576 | 0.499 | 0.661 |
| log-sigma-3-mm-3D_glcm_ClusterProminence_32_binCount | 0.627 | 0.553 | 0.707 |
| log-sigma-3-mm-3D_glcm_ClusterShade_32_binCount | 0.66 | 0.588 | 0.736 |
| log-sigma-3-mm-3D_glcm_ClusterTendency_32_binCount | 0.764 | 0.704 | 0.822 |
| log-sigma-3-mm-3D_glcm_Contrast_32_binCount | 0.808 | 0.756 | 0.857 |
| log-sigma-3-mm-3D_glcm_Correlation_32_binCount | 0.681 | 0.61 | 0.754 |
| log-sigma-3-mm-3D_glcm_DifferenceAverage_32_binCount | 0.852 | 0.809 | 0.892 |
| log-sigma-3-mm-3D_glcm_DifferenceEntropy_32_binCount | 0.854 | 0.812 | 0.893 |
| log-sigma-3-mm-3D_glcm_DifferenceVariance_32_binCount | 0.783 | 0.726 | 0.838 |
| log-sigma-3-mm-3D_glcm_Id_32_binCount | 0.88 | 0.844 | 0.913 |
| log-sigma-3-mm-3D_glcm_Idm_32_binCount | 0.879 | 0.842 | 0.912 |
| log-sigma-3-mm-3D_glcm_Idmn_32_binCount | 0.812 | 0.76 | 0.861 |
| log-sigma-3-mm-3D_glcm_Idn_32_binCount | 0.857 | 0.815 | 0.895 |
| log-sigma-3-mm-3D_glcm_Imc1_32_binCount | 0.819 | 0.769 | 0.866 |
| log-sigma-3-mm-3D_glcm_Imc2_32_binCount | 0.758 | 0.698 | 0.818 |
| log-sigma-3-mm-3D_glcm_InverseVariance_32_binCount | 0.759 | 0.698 | 0.819 |
| log-sigma-3-mm-3D_glcm_JointAverage_32_binCount | 0.587 | 0.511 | 0.672 |
| log-sigma-3-mm-3D_glcm_JointEnergy_32_binCount | 0.845 | 0.8 | 0.886 |
| log-sigma-3-mm-3D_glcm_JointEntropy_32_binCount | 0.825 | 0.777 | 0.871 |
| log-sigma-3-mm-3D_glcm_MCC_32_binCount | 0.514 | 0.436 | 0.603 |
| log-sigma-3-mm-3D_glcm_MaximumProbability_32_binCount | 0.838 | 0.792 | 0.881 |
| log-sigma-3-mm-3D_glcm_SumAverage_32_binCount | 0.587 | 0.511 | 0.672 |
| log-sigma-3-mm-3D_glcm_SumEntropy_32_binCount | 0.811 | 0.76 | 0.86 |
| log-sigma-3-mm-3D_glcm_SumSquares_32_binCount | 0.771 | 0.712 | 0.828 |
| log-sigma-3-mm-3D_glrlm_GrayLevelNonUniformity_32_binCount | 0.989 | 0.985 | 0.992 |
| log-sigma-3-mm-3D_glrlm_GrayLevelNonUniformityNormalized_32_binCount | 0.825 | 0.777 | 0.871 |
| log-sigma-3-mm-3D_glrlm_GrayLevelVariance_32_binCount | 0.782 | 0.726 | 0.837 |
| log-sigma-3-mm-3D_glrlm_HighGrayLevelRunEmphasis_32_binCount | 0.547 | 0.47 | 0.635 |
| log-sigma-3-mm-3D_glrlm_LongRunEmphasis_32_binCount | 0.914 | 0.887 | 0.938 |
| log-sigma-3-mm-3D_glrlm_LongRunHighGrayLevelEmphasis_32_binCount | 0.801 | 0.748 | 0.852 |
| log-sigma-3-mm-3D_glrlm_LongRunLowGrayLevelEmphasis_32_binCount | 0.513 | 0.435 | 0.603 |
| log-sigma-3-mm-3D_glrlm_LowGrayLevelRunEmphasis_32_binCount | 0.577 | 0.5 | 0.662 |
| log-sigma-3-mm-3D_glrlm_RunEntropy_32_binCount | 0.72 | 0.654 | 0.787 |
| log-sigma-3-mm-3D_glrlm_RunLengthNonUniformity_32_binCount | 0.963 | 0.95 | 0.974 |
| log-sigma-3-mm-3D_glrlm_RunLengthNonUniformityNormalized_32_binCount | 0.909 | 0.88 | 0.934 |
| log-sigma-3-mm-3D_glrlm_RunPercentage_32_binCount | 0.92 | 0.894 | 0.942 |
| log-sigma-3-mm-3D_glrlm_RunVariance_32_binCount | 0.922 | 0.897 | 0.944 |
| log-sigma-3-mm-3D_glrlm_ShortRunEmphasis_32_binCount | 0.906 | 0.876 | 0.932 |
| log-sigma-3-mm-3D_glrlm_ShortRunHighGrayLevelEmphasis_32_binCount | 0.601 | 0.525 | 0.684 |
| log-sigma-3-mm-3D_glrlm_ShortRunLowGrayLevelEmphasis_32_binCount | 0.593 | 0.517 | 0.677 |
| log-sigma-3-mm-3D_glszm_GrayLevelNonUniformity_32_binCount | 0.945 | 0.927 | 0.961 |
| log-sigma-3-mm-3D_glszm_GrayLevelNonUniformityNormalized_32_binCount | 0.697 | 0.628 | 0.767 |
| log-sigma-3-mm-3D_glszm_GrayLevelVariance_32_binCount | 0.702 | 0.633 | 0.771 |
| log-sigma-3-mm-3D_glszm_HighGrayLevelZoneEmphasis_32_binCount | 0.462 | 0.385 | 0.553 |
| log-sigma-3-mm-3D_glszm_LargeAreaEmphasis_32_binCount | 0.861 | 0.82 | 0.898 |
| log-sigma-3-mm-3D_glszm_LargeAreaHighGrayLevelEmphasis_32_binCount | 0.795 | 0.74 | 0.847 |
| log-sigma-3-mm-3D_glszm_LargeAreaLowGrayLevelEmphasis_32_binCount | 0.905 | 0.876 | 0.932 |
| log-sigma-3-mm-3D_glszm_LowGrayLevelZoneEmphasis_32_binCount | 0.608 | 0.533 | 0.69 |
| log-sigma-3-mm-3D_glszm_SizeZoneNonUniformity_32_binCount | 0.643 | 0.569 | 0.721 |
| log-sigma-3-mm-3D_glszm_SizeZoneNonUniformityNormalized_32_binCount | 0.752 | 0.69 | 0.813 |
| log-sigma-3-mm-3D_glszm_SmallAreaEmphasis_32_binCount | 0.741 | 0.678 | 0.804 |
| log-sigma-3-mm-3D_glszm_SmallAreaHighGrayLevelEmphasis_32_binCount | 0.618 | 0.542 | 0.699 |
| log-sigma-3-mm-3D_glszm_SmallAreaLowGrayLevelEmphasis_32_binCount | 0.502 | 0.425 | 0.592 |
| log-sigma-3-mm-3D_glszm_ZoneEntropy_32_binCount | 0.894 | 0.861 | 0.923 |
| log-sigma-3-mm-3D_glszm_ZonePercentage_32_binCount | 0.896 | 0.864 | 0.925 |
| log-sigma-3-mm-3D_glszm_ZoneVariance_32_binCount | 0.861 | 0.82 | 0.899 |
| log-sigma-3-mm-3D_gldm_DependenceEntropy_32_binCount | 0.92 | 0.894 | 0.943 |
| log-sigma-3-mm-3D_gldm_DependenceNonUniformity_32_binCount | 0.986 | 0.98 | 0.99 |
| log-sigma-3-mm-3D_gldm_DependenceNonUniformityNormalized_32_binCount | 0.884 | 0.849 | 0.916 |
| log-sigma-3-mm-3D_gldm_DependenceVariance_32_binCount | 0.925 | 0.9 | 0.946 |
| log-sigma-3-mm-3D_gldm_GrayLevelNonUniformity_32_binCount | 0.971 | 0.961 | 0.98 |
| log-sigma-3-mm-3D_gldm_GrayLevelVariance_32_binCount | 0.789 | 0.733 | 0.843 |
| log-sigma-3-mm-3D_gldm_HighGrayLevelEmphasis_32_binCount | 0.558 | 0.481 | 0.645 |
| log-sigma-3-mm-3D_gldm_LargeDependenceEmphasis_32_binCount | 0.92 | 0.894 | 0.943 |
| log-sigma-3-mm-3D_gldm_LargeDependenceHighGrayLevelEmphasis_32_binCount | 0.822 | 0.773 | 0.869 |
| log-sigma-3-mm-3D_gldm_LargeDependenceLowGrayLevelEmphasis_32_binCount | 0.541 | 0.463 | 0.629 |
| log-sigma-3-mm-3D_gldm_LowGrayLevelEmphasis_32_binCount | 0.566 | 0.489 | 0.652 |
| log-sigma-3-mm-3D_gldm_SmallDependenceEmphasis_32_binCount | 0.881 | 0.846 | 0.914 |
| log-sigma-3-mm-3D_gldm_SmallDependenceHighGrayLevelEmphasis_32_binCount | 0.907 | 0.877 | 0.933 |
| log-sigma-3-mm-3D_gldm_SmallDependenceLowGrayLevelEmphasis_32_binCount | 0.78 | 0.723 | 0.835 |
| log-sigma-3-mm-3D_ngtdm_Busyness_32_binCount | 0.985 | 0.979 | 0.989 |
| log-sigma-3-mm-3D_ngtdm_Coarseness_32_binCount | 0.96 | 0.946 | 0.971 |
| log-sigma-3-mm-3D_ngtdm_Complexity_32_binCount | 0.792 | 0.737 | 0.845 |
| log-sigma-3-mm-3D_ngtdm_Contrast_32_binCount | 0.821 | 0.772 | 0.868 |
| log-sigma-3-mm-3D_ngtdm_Strength_32_binCount | 0.887 | 0.852 | 0.918 |
| log-sigma-4-mm-3D_firstorder_10Percentile_32_binCount | 0.886 | 0.851 | 0.917 |
| log-sigma-4-mm-3D_firstorder_90Percentile_32_binCount | 0.98 | 0.972 | 0.986 |
| log-sigma-4-mm-3D_firstorder_Energy_32_binCount | 0.989 | 0.985 | 0.992 |
| log-sigma-4-mm-3D_firstorder_Entropy_32_binCount | 0.865 | 0.826 | 0.902 |
| log-sigma-4-mm-3D_firstorder_InterquartileRange_32_binCount | 0.877 | 0.84 | 0.911 |
| log-sigma-4-mm-3D_firstorder_Kurtosis_32_binCount | 0.687 | 0.617 | 0.759 |
| log-sigma-4-mm-3D_firstorder_Maximum_32_binCount | 0.976 | 0.968 | 0.983 |
| log-sigma-4-mm-3D_firstorder_MeanAbsoluteDeviation_32_binCount | 0.917 | 0.89 | 0.94 |
| log-sigma-4-mm-3D_firstorder_Mean_32_binCount | 0.959 | 0.945 | 0.971 |
| log-sigma-4-mm-3D_firstorder_Median_32_binCount | 0.967 | 0.955 | 0.977 |
| log-sigma-4-mm-3D_firstorder_Minimum_32_binCount | 0.826 | 0.777 | 0.872 |
| log-sigma-4-mm-3D_firstorder_Range_32_binCount | 0.908 | 0.879 | 0.934 |
| log-sigma-4-mm-3D_firstorder_RobustMeanAbsoluteDeviation_32_binCount | 0.894 | 0.861 | 0.923 |
| log-sigma-4-mm-3D_firstorder_RootMeanSquared_32_binCount | 0.964 | 0.952 | 0.975 |
| log-sigma-4-mm-3D_firstorder_Skewness_32_binCount | 0.726 | 0.66 | 0.791 |
| log-sigma-4-mm-3D_firstorder_TotalEnergy_32_binCount | 0.989 | 0.985 | 0.992 |
| log-sigma-4-mm-3D_firstorder_Uniformity_32_binCount | 0.854 | 0.812 | 0.893 |
| log-sigma-4-mm-3D_firstorder_Variance_32_binCount | 0.895 | 0.862 | 0.924 |
| log-sigma-4-mm-3D_glcm_Autocorrelation_32_binCount | 0.764 | 0.705 | 0.823 |
| log-sigma-4-mm-3D_glcm_ClusterProminence_32_binCount | 0.765 | 0.705 | 0.823 |
| log-sigma-4-mm-3D_glcm_ClusterShade_32_binCount | 0.734 | 0.669 | 0.798 |
| log-sigma-4-mm-3D_glcm_ClusterTendency_32_binCount | 0.822 | 0.773 | 0.869 |
| log-sigma-4-mm-3D_glcm_Contrast_32_binCount | 0.865 | 0.826 | 0.902 |
| log-sigma-4-mm-3D_glcm_Correlation_32_binCount | 0.798 | 0.744 | 0.85 |
| log-sigma-4-mm-3D_glcm_DifferenceAverage_32_binCount | 0.899 | 0.868 | 0.927 |
| log-sigma-4-mm-3D_glcm_DifferenceEntropy_32_binCount | 0.896 | 0.864 | 0.925 |
| log-sigma-4-mm-3D_glcm_DifferenceVariance_32_binCount | 0.854 | 0.811 | 0.893 |
| log-sigma-4-mm-3D_glcm_Id_32_binCount | 0.916 | 0.889 | 0.94 |
| log-sigma-4-mm-3D_glcm_Idm_32_binCount | 0.916 | 0.889 | 0.94 |
| log-sigma-4-mm-3D_glcm_Idmn_32_binCount | 0.869 | 0.83 | 0.904 |
| log-sigma-4-mm-3D_glcm_Idn_32_binCount | 0.903 | 0.872 | 0.93 |
| log-sigma-4-mm-3D_glcm_Imc1_32_binCount | 0.835 | 0.789 | 0.879 |
| log-sigma-4-mm-3D_glcm_Imc2_32_binCount | 0.766 | 0.707 | 0.824 |
| log-sigma-4-mm-3D_glcm_InverseVariance_32_binCount | 0.843 | 0.798 | 0.885 |
| log-sigma-4-mm-3D_glcm_JointAverage_32_binCount | 0.762 | 0.702 | 0.821 |
| log-sigma-4-mm-3D_glcm_JointEnergy_32_binCount | 0.847 | 0.803 | 0.888 |
| log-sigma-4-mm-3D_glcm_JointEntropy_32_binCount | 0.866 | 0.827 | 0.903 |
| log-sigma-4-mm-3D_glcm_MCC_32_binCount | 0.653 | 0.58 | 0.73 |
| log-sigma-4-mm-3D_glcm_MaximumProbability_32_binCount | 0.839 | 0.793 | 0.881 |
| log-sigma-4-mm-3D_glcm_SumAverage_32_binCount | 0.762 | 0.702 | 0.821 |
| log-sigma-4-mm-3D_glcm_SumEntropy_32_binCount | 0.849 | 0.805 | 0.889 |
| log-sigma-4-mm-3D_glcm_SumSquares_32_binCount | 0.828 | 0.779 | 0.873 |
| log-sigma-4-mm-3D_glrlm_GrayLevelNonUniformity_32_binCount | 0.99 | 0.986 | 0.993 |
| log-sigma-4-mm-3D_glrlm_GrayLevelNonUniformityNormalized_32_binCount | 0.847 | 0.803 | 0.888 |
| log-sigma-4-mm-3D_glrlm_GrayLevelVariance_32_binCount | 0.842 | 0.796 | 0.884 |
| log-sigma-4-mm-3D_glrlm_HighGrayLevelRunEmphasis_32_binCount | 0.735 | 0.671 | 0.799 |
| log-sigma-4-mm-3D_glrlm_LongRunEmphasis_32_binCount | 0.934 | 0.912 | 0.953 |
| log-sigma-4-mm-3D_glrlm_LongRunHighGrayLevelEmphasis_32_binCount | 0.84 | 0.795 | 0.883 |
| log-sigma-4-mm-3D_glrlm_LongRunLowGrayLevelEmphasis_32_binCount | 0.652 | 0.579 | 0.729 |
| log-sigma-4-mm-3D_glrlm_LowGrayLevelRunEmphasis_32_binCount | 0.696 | 0.627 | 0.766 |
| log-sigma-4-mm-3D_glrlm_RunEntropy_32_binCount | 0.837 | 0.791 | 0.88 |
| log-sigma-4-mm-3D_glrlm_RunLengthNonUniformity_32_binCount | 0.967 | 0.955 | 0.977 |
| log-sigma-4-mm-3D_glrlm_RunLengthNonUniformityNormalized_32_binCount | 0.933 | 0.911 | 0.952 |
| log-sigma-4-mm-3D_glrlm_RunPercentage_32_binCount | 0.942 | 0.923 | 0.959 |
| log-sigma-4-mm-3D_glrlm_RunVariance_32_binCount | 0.942 | 0.923 | 0.959 |
| log-sigma-4-mm-3D_glrlm_ShortRunEmphasis_32_binCount | 0.929 | 0.906 | 0.949 |
| log-sigma-4-mm-3D_glrlm_ShortRunHighGrayLevelEmphasis_32_binCount | 0.778 | 0.721 | 0.834 |
| log-sigma-4-mm-3D_glrlm_ShortRunLowGrayLevelEmphasis_32_binCount | 0.712 | 0.645 | 0.78 |
| log-sigma-4-mm-3D_glszm_GrayLevelNonUniformity_32_binCount | 0.953 | 0.938 | 0.967 |
| log-sigma-4-mm-3D_glszm_GrayLevelNonUniformityNormalized_32_binCount | 0.777 | 0.719 | 0.833 |
| log-sigma-4-mm-3D_glszm_GrayLevelVariance_32_binCount | 0.773 | 0.714 | 0.83 |
| log-sigma-4-mm-3D_glszm_HighGrayLevelZoneEmphasis_32_binCount | 0.638 | 0.564 | 0.717 |
| log-sigma-4-mm-3D_glszm_LargeAreaEmphasis_32_binCount | 0.881 | 0.845 | 0.914 |
| log-sigma-4-mm-3D_glszm_LargeAreaHighGrayLevelEmphasis_32_binCount | 0.824 | 0.775 | 0.87 |
| log-sigma-4-mm-3D_glszm_LargeAreaLowGrayLevelEmphasis_32_binCount | 0.934 | 0.913 | 0.953 |
| log-sigma-4-mm-3D_glszm_LowGrayLevelZoneEmphasis_32_binCount | 0.621 | 0.546 | 0.702 |
| log-sigma-4-mm-3D_glszm_SizeZoneNonUniformity_32_binCount | 0.684 | 0.614 | 0.757 |
| log-sigma-4-mm-3D_glszm_SizeZoneNonUniformityNormalized_32_binCount | 0.782 | 0.726 | 0.837 |
| log-sigma-4-mm-3D_glszm_SmallAreaEmphasis_32_binCount | 0.753 | 0.692 | 0.814 |
| log-sigma-4-mm-3D_glszm_SmallAreaHighGrayLevelEmphasis_32_binCount | 0.735 | 0.671 | 0.799 |
| log-sigma-4-mm-3D_glszm_SmallAreaLowGrayLevelEmphasis_32_binCount | 0.531 | 0.453 | 0.62 |
| log-sigma-4-mm-3D_glszm_ZoneEntropy_32_binCount | 0.926 | 0.902 | 0.947 |
| log-sigma-4-mm-3D_glszm_ZonePercentage_32_binCount | 0.931 | 0.908 | 0.95 |
| log-sigma-4-mm-3D_glszm_ZoneVariance_32_binCount | 0.881 | 0.845 | 0.914 |
| log-sigma-4-mm-3D_gldm_DependenceEntropy_32_binCount | 0.943 | 0.924 | 0.959 |
| log-sigma-4-mm-3D_gldm_DependenceNonUniformity_32_binCount | 0.987 | 0.983 | 0.991 |
| log-sigma-4-mm-3D_gldm_DependenceNonUniformityNormalized_32_binCount | 0.917 | 0.891 | 0.941 |
| log-sigma-4-mm-3D_gldm_DependenceVariance_32_binCount | 0.937 | 0.916 | 0.955 |
| log-sigma-4-mm-3D_gldm_GrayLevelNonUniformity_32_binCount | 0.973 | 0.964 | 0.981 |
| log-sigma-4-mm-3D_gldm_GrayLevelVariance_32_binCount | 0.847 | 0.803 | 0.888 |
| log-sigma-4-mm-3D_gldm_HighGrayLevelEmphasis_32_binCount | 0.748 | 0.685 | 0.809 |
| log-sigma-4-mm-3D_gldm_LargeDependenceEmphasis_32_binCount | 0.94 | 0.92 | 0.957 |
| log-sigma-4-mm-3D_gldm_LargeDependenceHighGrayLevelEmphasis_32_binCount | 0.863 | 0.822 | 0.9 |
| log-sigma-4-mm-3D_gldm_LargeDependenceLowGrayLevelEmphasis_32_binCount | 0.689 | 0.619 | 0.761 |
| log-sigma-4-mm-3D_gldm_LowGrayLevelEmphasis_32_binCount | 0.691 | 0.621 | 0.762 |
| log-sigma-4-mm-3D_gldm_SmallDependenceEmphasis_32_binCount | 0.916 | 0.889 | 0.94 |
| log-sigma-4-mm-3D_gldm_SmallDependenceHighGrayLevelEmphasis_32_binCount | 0.929 | 0.906 | 0.949 |
| log-sigma-4-mm-3D_gldm_SmallDependenceLowGrayLevelEmphasis_32_binCount | 0.821 | 0.771 | 0.868 |
| log-sigma-4-mm-3D_ngtdm_Busyness_32_binCount | 0.989 | 0.986 | 0.993 |
| log-sigma-4-mm-3D_ngtdm_Coarseness_32_binCount | 0.968 | 0.957 | 0.977 |
| log-sigma-4-mm-3D_ngtdm_Complexity_32_binCount | 0.87 | 0.832 | 0.906 |
| log-sigma-4-mm-3D_ngtdm_Contrast_32_binCount | 0.866 | 0.826 | 0.902 |
| log-sigma-4-mm-3D_ngtdm_Strength_32_binCount | 0.944 | 0.925 | 0.96 |
| log-sigma-5-mm-3D_firstorder_10Percentile_32_binCount | 0.913 | 0.885 | 0.937 |
| log-sigma-5-mm-3D_firstorder_90Percentile_32_binCount | 0.983 | 0.977 | 0.988 |
| log-sigma-5-mm-3D_firstorder_Energy_32_binCount | 0.99 | 0.987 | 0.993 |
| log-sigma-5-mm-3D_firstorder_Entropy_32_binCount | 0.867 | 0.828 | 0.903 |
| log-sigma-5-mm-3D_firstorder_InterquartileRange_32_binCount | 0.924 | 0.9 | 0.946 |
| log-sigma-5-mm-3D_firstorder_Kurtosis_32_binCount | 0.768 | 0.709 | 0.826 |
| log-sigma-5-mm-3D_firstorder_Maximum_32_binCount | 0.987 | 0.982 | 0.991 |
| log-sigma-5-mm-3D_firstorder_MeanAbsoluteDeviation_32_binCount | 0.938 | 0.918 | 0.956 |
| log-sigma-5-mm-3D_firstorder_Mean_32_binCount | 0.971 | 0.961 | 0.98 |
| log-sigma-5-mm-3D_firstorder_Median_32_binCount | 0.973 | 0.964 | 0.981 |
| log-sigma-5-mm-3D_firstorder_Minimum_32_binCount | 0.862 | 0.822 | 0.899 |
| log-sigma-5-mm-3D_firstorder_Range_32_binCount | 0.925 | 0.901 | 0.946 |
| log-sigma-5-mm-3D_firstorder_RobustMeanAbsoluteDeviation_32_binCount | 0.928 | 0.904 | 0.948 |
| log-sigma-5-mm-3D_firstorder_RootMeanSquared_32_binCount | 0.961 | 0.948 | 0.973 |
| log-sigma-5-mm-3D_firstorder_Skewness_32_binCount | 0.821 | 0.772 | 0.868 |
| log-sigma-5-mm-3D_firstorder_TotalEnergy_32_binCount | 0.99 | 0.987 | 0.993 |
| log-sigma-5-mm-3D_firstorder_Uniformity_32_binCount | 0.852 | 0.809 | 0.892 |
| log-sigma-5-mm-3D_firstorder_Variance_32_binCount | 0.932 | 0.911 | 0.952 |
| log-sigma-5-mm-3D_glcm_Autocorrelation_32_binCount | 0.852 | 0.809 | 0.892 |
| log-sigma-5-mm-3D_glcm_ClusterProminence_32_binCount | 0.777 | 0.719 | 0.833 |
| log-sigma-5-mm-3D_glcm_ClusterShade_32_binCount | 0.793 | 0.738 | 0.845 |
| log-sigma-5-mm-3D_glcm_ClusterTendency_32_binCount | 0.827 | 0.779 | 0.872 |
| log-sigma-5-mm-3D_glcm_Contrast_32_binCount | 0.877 | 0.84 | 0.911 |
| log-sigma-5-mm-3D_glcm_Correlation_32_binCount | 0.81 | 0.758 | 0.859 |
| log-sigma-5-mm-3D_glcm_DifferenceAverage_32_binCount | 0.918 | 0.891 | 0.941 |
| log-sigma-5-mm-3D_glcm_DifferenceEntropy_32_binCount | 0.905 | 0.875 | 0.932 |
| log-sigma-5-mm-3D_glcm_DifferenceVariance_32_binCount | 0.863 | 0.823 | 0.9 |
| log-sigma-5-mm-3D_glcm_Id_32_binCount | 0.937 | 0.917 | 0.955 |
| log-sigma-5-mm-3D_glcm_Idm_32_binCount | 0.938 | 0.917 | 0.956 |
| log-sigma-5-mm-3D_glcm_Idmn_32_binCount | 0.88 | 0.844 | 0.913 |
| log-sigma-5-mm-3D_glcm_Idn_32_binCount | 0.922 | 0.897 | 0.944 |
| log-sigma-5-mm-3D_glcm_Imc1_32_binCount | 0.856 | 0.814 | 0.895 |
| log-sigma-5-mm-3D_glcm_Imc2_32_binCount | 0.771 | 0.713 | 0.828 |
| log-sigma-5-mm-3D_glcm_InverseVariance_32_binCount | 0.861 | 0.82 | 0.899 |
| log-sigma-5-mm-3D_glcm_JointAverage_32_binCount | 0.849 | 0.805 | 0.889 |
| log-sigma-5-mm-3D_glcm_JointEnergy_32_binCount | 0.855 | 0.812 | 0.894 |
| log-sigma-5-mm-3D_glcm_JointEntropy_32_binCount | 0.878 | 0.841 | 0.911 |
| log-sigma-5-mm-3D_glcm_MCC_32_binCount | 0.732 | 0.668 | 0.797 |
| log-sigma-5-mm-3D_glcm_MaximumProbability_32_binCount | 0.841 | 0.795 | 0.883 |
| log-sigma-5-mm-3D_glcm_SumAverage_32_binCount | 0.849 | 0.805 | 0.889 |
| log-sigma-5-mm-3D_glcm_SumEntropy_32_binCount | 0.847 | 0.803 | 0.888 |
| log-sigma-5-mm-3D_glcm_SumSquares_32_binCount | 0.831 | 0.784 | 0.876 |
| log-sigma-5-mm-3D_glrlm_GrayLevelNonUniformity_32_binCount | 0.991 | 0.987 | 0.993 |
| log-sigma-5-mm-3D_glrlm_GrayLevelNonUniformityNormalized_32_binCount | 0.847 | 0.803 | 0.888 |
| log-sigma-5-mm-3D_glrlm_GrayLevelVariance_32_binCount | 0.842 | 0.797 | 0.884 |
| log-sigma-5-mm-3D_glrlm_HighGrayLevelRunEmphasis_32_binCount | 0.834 | 0.787 | 0.878 |
| log-sigma-5-mm-3D_glrlm_LongRunEmphasis_32_binCount | 0.941 | 0.922 | 0.958 |
| log-sigma-5-mm-3D_glrlm_LongRunHighGrayLevelEmphasis_32_binCount | 0.877 | 0.84 | 0.911 |
| log-sigma-5-mm-3D_glrlm_LongRunLowGrayLevelEmphasis_32_binCount | 0.714 | 0.647 | 0.782 |
| log-sigma-5-mm-3D_glrlm_LowGrayLevelRunEmphasis_32_binCount | 0.769 | 0.71 | 0.827 |
| log-sigma-5-mm-3D_glrlm_RunEntropy_32_binCount | 0.874 | 0.837 | 0.909 |
| log-sigma-5-mm-3D_glrlm_RunLengthNonUniformity_32_binCount | 0.97 | 0.959 | 0.979 |
| log-sigma-5-mm-3D_glrlm_RunLengthNonUniformityNormalized_32_binCount | 0.946 | 0.929 | 0.962 |
| log-sigma-5-mm-3D_glrlm_RunPercentage_32_binCount | 0.955 | 0.94 | 0.968 |
| log-sigma-5-mm-3D_glrlm_RunVariance_32_binCount | 0.949 | 0.932 | 0.964 |
| log-sigma-5-mm-3D_glrlm_ShortRunEmphasis_32_binCount | 0.94 | 0.921 | 0.958 |
| log-sigma-5-mm-3D_glrlm_ShortRunHighGrayLevelEmphasis_32_binCount | 0.849 | 0.806 | 0.89 |
| log-sigma-5-mm-3D_glrlm_ShortRunLowGrayLevelEmphasis_32_binCount | 0.777 | 0.719 | 0.833 |
| log-sigma-5-mm-3D_glszm_GrayLevelNonUniformity_32_binCount | 0.956 | 0.941 | 0.969 |
| log-sigma-5-mm-3D_glszm_GrayLevelNonUniformityNormalized_32_binCount | 0.765 | 0.705 | 0.823 |
| log-sigma-5-mm-3D_glszm_GrayLevelVariance_32_binCount | 0.789 | 0.734 | 0.843 |
| log-sigma-5-mm-3D_glszm_HighGrayLevelZoneEmphasis_32_binCount | 0.723 | 0.657 | 0.789 |
| log-sigma-5-mm-3D_glszm_LargeAreaEmphasis_32_binCount | 0.884 | 0.849 | 0.916 |
| log-sigma-5-mm-3D_glszm_LargeAreaHighGrayLevelEmphasis_32_binCount | 0.828 | 0.78 | 0.873 |
| log-sigma-5-mm-3D_glszm_LargeAreaLowGrayLevelEmphasis_32_binCount | 0.939 | 0.919 | 0.957 |
| log-sigma-5-mm-3D_glszm_LowGrayLevelZoneEmphasis_32_binCount | 0.596 | 0.519 | 0.679 |
| log-sigma-5-mm-3D_glszm_SizeZoneNonUniformity_32_binCount | 0.685 | 0.615 | 0.757 |
| log-sigma-5-mm-3D_glszm_SizeZoneNonUniformityNormalized_32_binCount | 0.762 | 0.702 | 0.821 |
| log-sigma-5-mm-3D_glszm_SmallAreaEmphasis_32_binCount | 0.719 | 0.653 | 0.786 |
| log-sigma-5-mm-3D_glszm_SmallAreaHighGrayLevelEmphasis_32_binCount | 0.688 | 0.618 | 0.759 |
| log-sigma-5-mm-3D_glszm_SmallAreaLowGrayLevelEmphasis_32_binCount | 0.522 | 0.444 | 0.611 |
| log-sigma-5-mm-3D_glszm_ZoneEntropy_32_binCount | 0.931 | 0.909 | 0.951 |
| log-sigma-5-mm-3D_glszm_ZonePercentage_32_binCount | 0.941 | 0.922 | 0.958 |
| log-sigma-5-mm-3D_glszm_ZoneVariance_32_binCount | 0.884 | 0.848 | 0.916 |
| log-sigma-5-mm-3D_gldm_DependenceEntropy_32_binCount | 0.95 | 0.933 | 0.964 |
| log-sigma-5-mm-3D_gldm_DependenceNonUniformity_32_binCount | 0.989 | 0.985 | 0.992 |
| log-sigma-5-mm-3D_gldm_DependenceNonUniformityNormalized_32_binCount | 0.932 | 0.91 | 0.951 |
| log-sigma-5-mm-3D_gldm_DependenceVariance_32_binCount | 0.95 | 0.934 | 0.965 |
| log-sigma-5-mm-3D_gldm_GrayLevelNonUniformity_32_binCount | 0.977 | 0.969 | 0.984 |
| log-sigma-5-mm-3D_gldm_GrayLevelVariance_32_binCount | 0.851 | 0.807 | 0.891 |
| log-sigma-5-mm-3D_gldm_HighGrayLevelEmphasis_32_binCount | 0.843 | 0.798 | 0.885 |
| log-sigma-5-mm-3D_gldm_LargeDependenceEmphasis_32_binCount | 0.949 | 0.933 | 0.964 |
| log-sigma-5-mm-3D_gldm_LargeDependenceHighGrayLevelEmphasis_32_binCount | 0.897 | 0.865 | 0.926 |
| log-sigma-5-mm-3D_gldm_LargeDependenceLowGrayLevelEmphasis_32_binCount | 0.659 | 0.587 | 0.735 |
| log-sigma-5-mm-3D_gldm_LowGrayLevelEmphasis_32_binCount | 0.771 | 0.712 | 0.828 |
| log-sigma-5-mm-3D_gldm_SmallDependenceEmphasis_32_binCount | 0.928 | 0.905 | 0.949 |
| log-sigma-5-mm-3D_gldm_SmallDependenceHighGrayLevelEmphasis_32_binCount | 0.927 | 0.903 | 0.948 |
| log-sigma-5-mm-3D_gldm_SmallDependenceLowGrayLevelEmphasis_32_binCount | 0.836 | 0.789 | 0.879 |
| log-sigma-5-mm-3D_ngtdm_Busyness_32_binCount | 0.992 | 0.989 | 0.994 |
| log-sigma-5-mm-3D_ngtdm_Coarseness_32_binCount | 0.961 | 0.947 | 0.972 |
| log-sigma-5-mm-3D_ngtdm_Complexity_32_binCount | 0.886 | 0.851 | 0.917 |
| log-sigma-5-mm-3D_ngtdm_Contrast_32_binCount | 0.87 | 0.832 | 0.906 |
| log-sigma-5-mm-3D_ngtdm_Strength_32_binCount | 0.945 | 0.927 | 0.961 |
| wavelet-LLH_firstorder_10Percentile_32_binCount | 0.819 | 0.769 | 0.866 |
| wavelet-LLH_firstorder_90Percentile_32_binCount | 0.77 | 0.712 | 0.828 |
| wavelet-LLH_firstorder_Energy_32_binCount | 0.184 | 0.138 | 0.25 |
| wavelet-LLH_firstorder_Entropy_32_binCount | 0.842 | 0.797 | 0.884 |
| wavelet-LLH_firstorder_InterquartileRange_32_binCount | 0.948 | 0.931 | 0.963 |
| wavelet-LLH_firstorder_Kurtosis_32_binCount | 0.541 | 0.463 | 0.629 |
| wavelet-LLH_firstorder_Maximum_32_binCount | 0.555 | 0.477 | 0.642 |
| wavelet-LLH_firstorder_MeanAbsoluteDeviation_32_binCount | 0.883 | 0.848 | 0.915 |
| wavelet-LLH_firstorder_Mean_32_binCount | 0.734 | 0.67 | 0.798 |
| wavelet-LLH_firstorder_Median_32_binCount | 0.836 | 0.79 | 0.879 |
| wavelet-LLH_firstorder_Minimum_32_binCount | 0.73 | 0.666 | 0.795 |
| wavelet-LLH_firstorder_Range_32_binCount | 0.727 | 0.662 | 0.792 |
| wavelet-LLH_firstorder_RobustMeanAbsoluteDeviation_32_binCount | 0.922 | 0.898 | 0.944 |
| wavelet-LLH_firstorder_RootMeanSquared_32_binCount | 0.741 | 0.678 | 0.804 |
| wavelet-LLH_firstorder_Skewness_32_binCount | 0.394 | 0.321 | 0.485 |
| wavelet-LLH_firstorder_TotalEnergy_32_binCount | 0.184 | 0.138 | 0.25 |
| wavelet-LLH_firstorder_Uniformity_32_binCount | 0.828 | 0.78 | 0.873 |
| wavelet-LLH_firstorder_Variance_32_binCount | 0.431 | 0.356 | 0.523 |
| wavelet-LLH_glcm_Autocorrelation_32_binCount | 0.365 | 0.295 | 0.454 |
| wavelet-LLH_glcm_ClusterProminence_32_binCount | 0.76 | 0.699 | 0.819 |
| wavelet-LLH_glcm_ClusterShade_32_binCount | 0.679 | 0.608 | 0.752 |
| wavelet-LLH_glcm_ClusterTendency_32_binCount | 0.773 | 0.714 | 0.83 |
| wavelet-LLH_glcm_Contrast_32_binCount | 0.611 | 0.535 | 0.692 |
| wavelet-LLH_glcm_Correlation_32_binCount | 0.597 | 0.52 | 0.68 |
| wavelet-LLH_glcm_DifferenceAverage_32_binCount | 0.795 | 0.74 | 0.847 |
| wavelet-LLH_glcm_DifferenceEntropy_32_binCount | 0.822 | 0.773 | 0.869 |
| wavelet-LLH_glcm_DifferenceVariance_32_binCount | 0.542 | 0.464 | 0.63 |
| wavelet-LLH_glcm_Id_32_binCount | 0.839 | 0.793 | 0.882 |
| wavelet-LLH_glcm_Idm_32_binCount | 0.836 | 0.79 | 0.879 |
| wavelet-LLH_glcm_Idmn_32_binCount | 0.684 | 0.614 | 0.756 |
| wavelet-LLH_glcm_Idn_32_binCount | 0.806 | 0.754 | 0.856 |
| wavelet-LLH_glcm_Imc1_32_binCount | 0.876 | 0.839 | 0.91 |
| wavelet-LLH_glcm_Imc2_32_binCount | 0.868 | 0.829 | 0.904 |
| wavelet-LLH_glcm_InverseVariance_32_binCount | 0.583 | 0.506 | 0.667 |
| wavelet-LLH_glcm_JointAverage_32_binCount | 0.381 | 0.309 | 0.471 |
| wavelet-LLH_glcm_JointEnergy_32_binCount | 0.756 | 0.695 | 0.816 |
| wavelet-LLH_glcm_JointEntropy_32_binCount | 0.822 | 0.773 | 0.869 |
| wavelet-LLH_glcm_MCC_32_binCount | 0.675 | 0.604 | 0.749 |
| wavelet-LLH_glcm_MaximumProbability_32_binCount | 0.814 | 0.763 | 0.862 |
| wavelet-LLH_glcm_SumAverage_32_binCount | 0.381 | 0.309 | 0.471 |
| wavelet-LLH_glcm_SumEntropy_32_binCount | 0.829 | 0.781 | 0.874 |
| wavelet-LLH_glcm_SumSquares_32_binCount | 0.779 | 0.722 | 0.835 |
| wavelet-LLH_glrlm_GrayLevelNonUniformity_32_binCount | 0.992 | 0.99 | 0.995 |
| wavelet-LLH_glrlm_GrayLevelNonUniformityNormalized_32_binCount | 0.825 | 0.776 | 0.871 |
| wavelet-LLH_glrlm_GrayLevelVariance_32_binCount | 0.808 | 0.756 | 0.858 |
| wavelet-LLH_glrlm_HighGrayLevelRunEmphasis_32_binCount | 0.358 | 0.288 | 0.446 |
| wavelet-LLH_glrlm_LongRunEmphasis_32_binCount | 0.608 | 0.532 | 0.69 |
| wavelet-LLH_glrlm_LongRunHighGrayLevelEmphasis_32_binCount | 0.593 | 0.517 | 0.677 |
| wavelet-LLH_glrlm_LongRunLowGrayLevelEmphasis_32_binCount | 0.133 | 0.096 | 0.187 |
| wavelet-LLH_glrlm_LowGrayLevelRunEmphasis_32_binCount | 0.463 | 0.386 | 0.554 |
| wavelet-LLH_glrlm_RunEntropy_32_binCount | 0.701 | 0.632 | 0.77 |
| wavelet-LLH_glrlm_RunLengthNonUniformity_32_binCount | 0.92 | 0.894 | 0.942 |
| wavelet-LLH_glrlm_RunLengthNonUniformityNormalized_32_binCount | 0.877 | 0.84 | 0.911 |
| wavelet-LLH_glrlm_RunPercentage_32_binCount | 0.886 | 0.852 | 0.918 |
| wavelet-LLH_glrlm_RunVariance_32_binCount | 0.6 | 0.524 | 0.683 |
| wavelet-LLH_glrlm_ShortRunEmphasis_32_binCount | 0.877 | 0.84 | 0.911 |
| wavelet-LLH_glrlm_ShortRunHighGrayLevelEmphasis_32_binCount | 0.426 | 0.351 | 0.517 |
| wavelet-LLH_glrlm_ShortRunLowGrayLevelEmphasis_32_binCount | 0.516 | 0.438 | 0.606 |
| wavelet-LLH_glszm_GrayLevelNonUniformity_32_binCount | 0.963 | 0.951 | 0.974 |
| wavelet-LLH_glszm_GrayLevelNonUniformityNormalized_32_binCount | 0.671 | 0.6 | 0.745 |
| wavelet-LLH_glszm_GrayLevelVariance_32_binCount | 0.769 | 0.71 | 0.826 |
| wavelet-LLH_glszm_HighGrayLevelZoneEmphasis_32_binCount | 0.338 | 0.27 | 0.426 |
| wavelet-LLH_glszm_LargeAreaEmphasis_32_binCount | 0.686 | 0.616 | 0.758 |
| wavelet-LLH_glszm_LargeAreaHighGrayLevelEmphasis_32_binCount | 0.698 | 0.629 | 0.768 |
| wavelet-LLH_glszm_LargeAreaLowGrayLevelEmphasis_32_binCount | 0.213 | 0.162 | 0.285 |
| wavelet-LLH_glszm_LowGrayLevelZoneEmphasis_32_binCount | 0.417 | 0.343 | 0.508 |
| wavelet-LLH_glszm_SizeZoneNonUniformity_32_binCount | 0.651 | 0.578 | 0.728 |
| wavelet-LLH_glszm_SizeZoneNonUniformityNormalized_32_binCount | 0.739 | 0.675 | 0.802 |
| wavelet-LLH_glszm_SmallAreaEmphasis_32_binCount | 0.736 | 0.673 | 0.8 |
| wavelet-LLH_glszm_SmallAreaHighGrayLevelEmphasis_32_binCount | 0.395 | 0.322 | 0.485 |
| wavelet-LLH_glszm_SmallAreaLowGrayLevelEmphasis_32_binCount | 0.389 | 0.317 | 0.48 |
| wavelet-LLH_glszm_ZoneEntropy_32_binCount | 0.797 | 0.743 | 0.849 |
| wavelet-LLH_glszm_ZonePercentage_32_binCount | 0.857 | 0.816 | 0.896 |
| wavelet-LLH_glszm_ZoneVariance_32_binCount | 0.687 | 0.617 | 0.759 |
| wavelet-LLH_gldm_DependenceEntropy_32_binCount | 0.745 | 0.682 | 0.807 |
| wavelet-LLH_gldm_DependenceNonUniformity_32_binCount | 0.826 | 0.778 | 0.872 |
| wavelet-LLH_gldm_DependenceNonUniformityNormalized_32_binCount | 0.796 | 0.741 | 0.848 |
| wavelet-LLH_gldm_DependenceVariance_32_binCount | 0.873 | 0.835 | 0.908 |
| wavelet-LLH_gldm_GrayLevelNonUniformity_32_binCount | 0.95 | 0.933 | 0.964 |
| wavelet-LLH_gldm_GrayLevelVariance_32_binCount | 0.809 | 0.757 | 0.858 |
| wavelet-LLH_gldm_HighGrayLevelEmphasis_32_binCount | 0.36 | 0.29 | 0.449 |
| wavelet-LLH_gldm_LargeDependenceEmphasis_32_binCount | 0.879 | 0.842 | 0.912 |
| wavelet-LLH_gldm_LargeDependenceHighGrayLevelEmphasis_32_binCount | 0.717 | 0.651 | 0.784 |
| wavelet-LLH_gldm_LargeDependenceLowGrayLevelEmphasis_32_binCount | 0.427 | 0.351 | 0.518 |
| wavelet-LLH_gldm_LowGrayLevelEmphasis_32_binCount | 0.479 | 0.401 | 0.569 |
| wavelet-LLH_gldm_SmallDependenceEmphasis_32_binCount | 0.843 | 0.798 | 0.885 |
| wavelet-LLH_gldm_SmallDependenceHighGrayLevelEmphasis_32_binCount | 0.747 | 0.684 | 0.808 |
| wavelet-LLH_gldm_SmallDependenceLowGrayLevelEmphasis_32_binCount | 0.73 | 0.666 | 0.795 |
| wavelet-LLH_ngtdm_Busyness_32_binCount | 0.92 | 0.895 | 0.943 |
| wavelet-LLH_ngtdm_Coarseness_32_binCount | 0.942 | 0.923 | 0.959 |
| wavelet-LLH_ngtdm_Complexity_32_binCount | 0.635 | 0.56 | 0.714 |
| wavelet-LLH_ngtdm_Contrast_32_binCount | 0.695 | 0.625 | 0.765 |
| wavelet-LLH_ngtdm_Strength_32_binCount | 0.857 | 0.815 | 0.896 |
| wavelet-LHL_firstorder_10Percentile_32_binCount | 0.782 | 0.725 | 0.837 |
| wavelet-LHL_firstorder_90Percentile_32_binCount | 0.853 | 0.811 | 0.893 |
| wavelet-LHL_firstorder_Energy_32_binCount | 0.924 | 0.899 | 0.946 |
| wavelet-LHL_firstorder_Entropy_32_binCount | 0.821 | 0.771 | 0.868 |
| wavelet-LHL_firstorder_InterquartileRange_32_binCount | 0.938 | 0.918 | 0.956 |
| wavelet-LHL_firstorder_Kurtosis_32_binCount | 0.548 | 0.47 | 0.635 |
| wavelet-LHL_firstorder_Maximum_32_binCount | 0.798 | 0.744 | 0.85 |
| wavelet-LHL_firstorder_MeanAbsoluteDeviation_32_binCount | 0.849 | 0.805 | 0.889 |
| wavelet-LHL_firstorder_Mean_32_binCount | 0.477 | 0.4 | 0.568 |
| wavelet-LHL_firstorder_Median_32_binCount | 0.616 | 0.541 | 0.698 |
| wavelet-LHL_firstorder_Minimum_32_binCount | 0.776 | 0.718 | 0.832 |
| wavelet-LHL_firstorder_Range_32_binCount | 0.831 | 0.783 | 0.875 |
| wavelet-LHL_firstorder_RobustMeanAbsoluteDeviation_32_binCount | 0.927 | 0.903 | 0.948 |
| wavelet-LHL_firstorder_RootMeanSquared_32_binCount | 0.769 | 0.71 | 0.826 |
| wavelet-LHL_firstorder_Skewness_32_binCount | 0.213 | 0.162 | 0.284 |
| wavelet-LHL_firstorder_TotalEnergy_32_binCount | 0.924 | 0.899 | 0.946 |
| wavelet-LHL_firstorder_Uniformity_32_binCount | 0.81 | 0.758 | 0.859 |
| wavelet-LHL_firstorder_Variance_32_binCount | 0.597 | 0.52 | 0.68 |
| wavelet-LHL_glcm_Autocorrelation_32_binCount | 0.206 | 0.156 | 0.276 |
| wavelet-LHL_glcm_ClusterProminence_32_binCount | 0.618 | 0.543 | 0.699 |
| wavelet-LHL_glcm_ClusterShade_32_binCount | 0.406 | 0.333 | 0.497 |
| wavelet-LHL_glcm_ClusterTendency_32_binCount | 0.724 | 0.659 | 0.79 |
| wavelet-LHL_glcm_Contrast_32_binCount | 0.731 | 0.667 | 0.796 |
| wavelet-LHL_glcm_Correlation_32_binCount | 0.59 | 0.513 | 0.674 |
| wavelet-LHL_glcm_DifferenceAverage_32_binCount | 0.793 | 0.738 | 0.846 |
| wavelet-LHL_glcm_DifferenceEntropy_32_binCount | 0.811 | 0.759 | 0.86 |
| wavelet-LHL_glcm_DifferenceVariance_32_binCount | 0.723 | 0.658 | 0.789 |
| wavelet-LHL_glcm_Id_32_binCount | 0.82 | 0.77 | 0.867 |
| wavelet-LHL_glcm_Idm_32_binCount | 0.819 | 0.769 | 0.866 |
| wavelet-LHL_glcm_Idmn_32_binCount | 0.742 | 0.678 | 0.804 |
| wavelet-LHL_glcm_Idn_32_binCount | 0.799 | 0.745 | 0.85 |
| wavelet-LHL_glcm_Imc1_32_binCount | 0.84 | 0.795 | 0.883 |
| wavelet-LHL_glcm_Imc2_32_binCount | 0.897 | 0.865 | 0.925 |
| wavelet-LHL_glcm_InverseVariance_32_binCount | 0.566 | 0.489 | 0.652 |
| wavelet-LHL_glcm_JointAverage_32_binCount | 0.209 | 0.158 | 0.279 |
| wavelet-LHL_glcm_JointEnergy_32_binCount | 0.758 | 0.697 | 0.818 |
| wavelet-LHL_glcm_JointEntropy_32_binCount | 0.812 | 0.761 | 0.861 |
| wavelet-LHL_glcm_MCC_32_binCount | 0.443 | 0.367 | 0.534 |
| wavelet-LHL_glcm_MaximumProbability_32_binCount | 0.773 | 0.715 | 0.83 |
| wavelet-LHL_glcm_SumAverage_32_binCount | 0.209 | 0.158 | 0.279 |
| wavelet-LHL_glcm_SumEntropy_32_binCount | 0.818 | 0.768 | 0.866 |
| wavelet-LHL_glcm_SumSquares_32_binCount | 0.736 | 0.672 | 0.8 |
| wavelet-LHL_glrlm_GrayLevelNonUniformity_32_binCount | 0.978 | 0.971 | 0.985 |
| wavelet-LHL_glrlm_GrayLevelNonUniformityNormalized_32_binCount | 0.807 | 0.755 | 0.857 |
| wavelet-LHL_glrlm_GrayLevelVariance_32_binCount | 0.749 | 0.687 | 0.81 |
| wavelet-LHL_glrlm_HighGrayLevelRunEmphasis_32_binCount | 0.206 | 0.156 | 0.276 |
| wavelet-LHL_glrlm_LongRunEmphasis_32_binCount | 0.704 | 0.636 | 0.773 |
| wavelet-LHL_glrlm_LongRunHighGrayLevelEmphasis_32_binCount | 0.629 | 0.554 | 0.709 |
| wavelet-LHL_glrlm_LongRunLowGrayLevelEmphasis_32_binCount | 0.471 | 0.394 | 0.562 |
| wavelet-LHL_glrlm_LowGrayLevelRunEmphasis_32_binCount | 0.253 | 0.196 | 0.331 |
| wavelet-LHL_glrlm_RunEntropy_32_binCount | 0.677 | 0.606 | 0.751 |
| wavelet-LHL_glrlm_RunLengthNonUniformity_32_binCount | 0.881 | 0.845 | 0.914 |
| wavelet-LHL_glrlm_RunLengthNonUniformityNormalized_32_binCount | 0.847 | 0.803 | 0.888 |
| wavelet-LHL_glrlm_RunPercentage_32_binCount | 0.858 | 0.816 | 0.896 |
| wavelet-LHL_glrlm_RunVariance_32_binCount | 0.662 | 0.59 | 0.738 |
| wavelet-LHL_glrlm_ShortRunEmphasis_32_binCount | 0.843 | 0.798 | 0.885 |
| wavelet-LHL_glrlm_ShortRunHighGrayLevelEmphasis_32_binCount | 0.294 | 0.231 | 0.378 |
| wavelet-LHL_glrlm_ShortRunLowGrayLevelEmphasis_32_binCount | 0.345 | 0.276 | 0.433 |
| wavelet-LHL_glszm_GrayLevelNonUniformity_32_binCount | 0.934 | 0.913 | 0.953 |
| wavelet-LHL_glszm_GrayLevelNonUniformityNormalized_32_binCount | 0.702 | 0.634 | 0.772 |
| wavelet-LHL_glszm_GrayLevelVariance_32_binCount | 0.71 | 0.643 | 0.778 |
| wavelet-LHL_glszm_HighGrayLevelZoneEmphasis_32_binCount | 0.212 | 0.161 | 0.283 |
| wavelet-LHL_glszm_LargeAreaEmphasis_32_binCount | 0.824 | 0.776 | 0.87 |
| wavelet-LHL_glszm_LargeAreaHighGrayLevelEmphasis_32_binCount | 0.782 | 0.725 | 0.837 |
| wavelet-LHL_glszm_LargeAreaLowGrayLevelEmphasis_32_binCount | 0.475 | 0.397 | 0.566 |
| wavelet-LHL_glszm_LowGrayLevelZoneEmphasis_32_binCount | 0.3 | 0.236 | 0.384 |
| wavelet-LHL_glszm_SizeZoneNonUniformity_32_binCount | 0.643 | 0.569 | 0.721 |
| wavelet-LHL_glszm_SizeZoneNonUniformityNormalized_32_binCount | 0.673 | 0.602 | 0.747 |
| wavelet-LHL_glszm_SmallAreaEmphasis_32_binCount | 0.675 | 0.604 | 0.748 |
| wavelet-LHL_glszm_SmallAreaHighGrayLevelEmphasis_32_binCount | 0.266 | 0.207 | 0.346 |
| wavelet-LHL_glszm_SmallAreaLowGrayLevelEmphasis_32_binCount | 0.433 | 0.357 | 0.524 |
| wavelet-LHL_glszm_ZoneEntropy_32_binCount | 0.702 | 0.634 | 0.771 |
| wavelet-LHL_glszm_ZonePercentage_32_binCount | 0.831 | 0.783 | 0.875 |
| wavelet-LHL_glszm_ZoneVariance_32_binCount | 0.825 | 0.776 | 0.871 |
| wavelet-LHL_gldm_DependenceEntropy_32_binCount | 0.774 | 0.716 | 0.831 |
| wavelet-LHL_gldm_DependenceNonUniformity_32_binCount | 0.784 | 0.728 | 0.839 |
| wavelet-LHL_gldm_DependenceNonUniformityNormalized_32_binCount | 0.737 | 0.673 | 0.801 |
| wavelet-LHL_gldm_DependenceVariance_32_binCount | 0.809 | 0.757 | 0.858 |
| wavelet-LHL_gldm_GrayLevelNonUniformity_32_binCount | 0.948 | 0.931 | 0.963 |
| wavelet-LHL_gldm_GrayLevelVariance_32_binCount | 0.748 | 0.686 | 0.81 |
| wavelet-LHL_gldm_HighGrayLevelEmphasis_32_binCount | 0.206 | 0.156 | 0.276 |
| wavelet-LHL_gldm_LargeDependenceEmphasis_32_binCount | 0.847 | 0.803 | 0.888 |
| wavelet-LHL_gldm_LargeDependenceHighGrayLevelEmphasis_32_binCount | 0.706 | 0.638 | 0.775 |
| wavelet-LHL_gldm_LargeDependenceLowGrayLevelEmphasis_32_binCount | 0.36 | 0.29 | 0.449 |
| wavelet-LHL_gldm_LowGrayLevelEmphasis_32_binCount | 0.247 | 0.19 | 0.324 |
| wavelet-LHL_gldm_SmallDependenceEmphasis_32_binCount | 0.817 | 0.767 | 0.865 |
| wavelet-LHL_gldm_SmallDependenceHighGrayLevelEmphasis_32_binCount | 0.721 | 0.655 | 0.787 |
| wavelet-LHL_gldm_SmallDependenceLowGrayLevelEmphasis_32_binCount | 0.713 | 0.646 | 0.781 |
| wavelet-LHL_ngtdm_Busyness_32_binCount | 0.838 | 0.792 | 0.881 |
| wavelet-LHL_ngtdm_Coarseness_32_binCount | 0.965 | 0.953 | 0.975 |
| wavelet-LHL_ngtdm_Complexity_32_binCount | 0.668 | 0.596 | 0.742 |
| wavelet-LHL_ngtdm_Contrast_32_binCount | 0.769 | 0.71 | 0.827 |
| wavelet-LHL_ngtdm_Strength_32_binCount | 0.892 | 0.859 | 0.922 |
| wavelet-LHH_firstorder_10Percentile_32_binCount | 0.862 | 0.821 | 0.899 |
| wavelet-LHH_firstorder_90Percentile_32_binCount | 0.848 | 0.804 | 0.888 |
| wavelet-LHH_firstorder_Energy_32_binCount | 0.908 | 0.879 | 0.934 |
| wavelet-LHH_firstorder_Entropy_32_binCount | 0.795 | 0.741 | 0.848 |
| wavelet-LHH_firstorder_InterquartileRange_32_binCount | 0.928 | 0.905 | 0.949 |
| wavelet-LHH_firstorder_Kurtosis_32_binCount | 0.22 | 0.168 | 0.293 |
| wavelet-LHH_firstorder_Maximum_32_binCount | 0.765 | 0.705 | 0.823 |
| wavelet-LHH_firstorder_MeanAbsoluteDeviation_32_binCount | 0.88 | 0.844 | 0.913 |
| wavelet-LHH_firstorder_Mean_32_binCount | 0.226 | 0.172 | 0.299 |
| wavelet-LHH_firstorder_Median_32_binCount | 0.323 | 0.257 | 0.409 |
| wavelet-LHH_firstorder_Minimum_32_binCount | 0.703 | 0.635 | 0.772 |
| wavelet-LHH_firstorder_Range_32_binCount | 0.771 | 0.712 | 0.828 |
| wavelet-LHH_firstorder_RobustMeanAbsoluteDeviation_32_binCount | 0.919 | 0.893 | 0.942 |
| wavelet-LHH_firstorder_RootMeanSquared_32_binCount | 0.85 | 0.807 | 0.89 |
| wavelet-LHH_firstorder_Skewness_32_binCount | 0.148 | 0.109 | 0.206 |
| wavelet-LHH_firstorder_TotalEnergy_32_binCount | 0.908 | 0.879 | 0.934 |
| wavelet-LHH_firstorder_Uniformity_32_binCount | 0.799 | 0.746 | 0.851 |
| wavelet-LHH_firstorder_Variance_32_binCount | 0.708 | 0.64 | 0.776 |
| wavelet-LHH_glcm_Autocorrelation_32_binCount | 0.155 | 0.114 | 0.214 |
| wavelet-LHH_glcm_ClusterProminence_32_binCount | 0.622 | 0.546 | 0.702 |
| wavelet-LHH_glcm_ClusterShade_32_binCount | 0.318 | 0.252 | 0.403 |
| wavelet-LHH_glcm_ClusterTendency_32_binCount | 0.669 | 0.598 | 0.744 |
| wavelet-LHH_glcm_Contrast_32_binCount | 0.696 | 0.627 | 0.767 |
| wavelet-LHH_glcm_Correlation_32_binCount | 0.665 | 0.593 | 0.74 |
| wavelet-LHH_glcm_DifferenceAverage_32_binCount | 0.746 | 0.683 | 0.808 |
| wavelet-LHH_glcm_DifferenceEntropy_32_binCount | 0.778 | 0.72 | 0.834 |
| wavelet-LHH_glcm_DifferenceVariance_32_binCount | 0.723 | 0.657 | 0.789 |
| wavelet-LHH_glcm_Id_32_binCount | 0.796 | 0.741 | 0.848 |
| wavelet-LHH_glcm_Idm_32_binCount | 0.792 | 0.737 | 0.845 |
| wavelet-LHH_glcm_Idmn_32_binCount | 0.697 | 0.628 | 0.767 |
| wavelet-LHH_glcm_Idn_32_binCount | 0.753 | 0.691 | 0.813 |
| wavelet-LHH_glcm_Imc1_32_binCount | 0.875 | 0.838 | 0.909 |
| wavelet-LHH_glcm_Imc2_32_binCount | 0.873 | 0.835 | 0.908 |
| wavelet-LHH_glcm_InverseVariance_32_binCount | 0.56 | 0.483 | 0.647 |
| wavelet-LHH_glcm_JointAverage_32_binCount | 0.149 | 0.109 | 0.207 |
| wavelet-LHH_glcm_JointEnergy_32_binCount | 0.759 | 0.699 | 0.819 |
| wavelet-LHH_glcm_JointEntropy_32_binCount | 0.781 | 0.724 | 0.836 |
| wavelet-LHH_glcm_MCC_32_binCount | 0.521 | 0.444 | 0.611 |
| wavelet-LHH_glcm_MaximumProbability_32_binCount | 0.766 | 0.707 | 0.824 |
| wavelet-LHH_glcm_SumAverage_32_binCount | 0.149 | 0.109 | 0.207 |
| wavelet-LHH_glcm_SumEntropy_32_binCount | 0.79 | 0.734 | 0.843 |
| wavelet-LHH_glcm_SumSquares_32_binCount | 0.682 | 0.612 | 0.755 |
| wavelet-LHH_glrlm_GrayLevelNonUniformity_32_binCount | 0.974 | 0.965 | 0.982 |
| wavelet-LHH_glrlm_GrayLevelNonUniformityNormalized_32_binCount | 0.783 | 0.726 | 0.838 |
| wavelet-LHH_glrlm_GrayLevelVariance_32_binCount | 0.714 | 0.648 | 0.782 |
| wavelet-LHH_glrlm_HighGrayLevelRunEmphasis_32_binCount | 0.155 | 0.114 | 0.214 |
| wavelet-LHH_glrlm_LongRunEmphasis_32_binCount | 0.701 | 0.632 | 0.77 |
| wavelet-LHH_glrlm_LongRunHighGrayLevelEmphasis_32_binCount | 0.665 | 0.593 | 0.74 |
| wavelet-LHH_glrlm_LongRunLowGrayLevelEmphasis_32_binCount | 0.576 | 0.499 | 0.661 |
| wavelet-LHH_glrlm_LowGrayLevelRunEmphasis_32_binCount | 0.3 | 0.236 | 0.384 |
| wavelet-LHH_glrlm_RunEntropy_32_binCount | 0.57 | 0.492 | 0.656 |
| wavelet-LHH_glrlm_RunLengthNonUniformity_32_binCount | 0.926 | 0.903 | 0.947 |
| wavelet-LHH_glrlm_RunLengthNonUniformityNormalized_32_binCount | 0.837 | 0.791 | 0.88 |
| wavelet-LHH_glrlm_RunPercentage_32_binCount | 0.855 | 0.813 | 0.894 |
| wavelet-LHH_glrlm_RunVariance_32_binCount | 0.678 | 0.607 | 0.751 |
| wavelet-LHH_glrlm_ShortRunEmphasis_32_binCount | 0.844 | 0.799 | 0.885 |
| wavelet-LHH_glrlm_ShortRunHighGrayLevelEmphasis_32_binCount | 0.276 | 0.216 | 0.357 |
| wavelet-LHH_glrlm_ShortRunLowGrayLevelEmphasis_32_binCount | 0.429 | 0.353 | 0.52 |
| wavelet-LHH_glszm_GrayLevelNonUniformity_32_binCount | 0.928 | 0.905 | 0.949 |
| wavelet-LHH_glszm_GrayLevelNonUniformityNormalized_32_binCount | 0.677 | 0.607 | 0.751 |
| wavelet-LHH_glszm_GrayLevelVariance_32_binCount | 0.712 | 0.645 | 0.78 |
| wavelet-LHH_glszm_HighGrayLevelZoneEmphasis_32_binCount | 0.154 | 0.113 | 0.213 |
| wavelet-LHH_glszm_LargeAreaEmphasis_32_binCount | 0.752 | 0.69 | 0.813 |
| wavelet-LHH_glszm_LargeAreaHighGrayLevelEmphasis_32_binCount | 0.601 | 0.525 | 0.684 |
| wavelet-LHH_glszm_LargeAreaLowGrayLevelEmphasis_32_binCount | 0.763 | 0.703 | 0.821 |
| wavelet-LHH_glszm_LowGrayLevelZoneEmphasis_32_binCount | 0.353 | 0.283 | 0.441 |
| wavelet-LHH_glszm_SizeZoneNonUniformity_32_binCount | 0.728 | 0.663 | 0.793 |
| wavelet-LHH_glszm_SizeZoneNonUniformityNormalized_32_binCount | 0.626 | 0.551 | 0.706 |
| wavelet-LHH_glszm_SmallAreaEmphasis_32_binCount | 0.617 | 0.542 | 0.698 |
| wavelet-LHH_glszm_SmallAreaHighGrayLevelEmphasis_32_binCount | 0.214 | 0.163 | 0.286 |
| wavelet-LHH_glszm_SmallAreaLowGrayLevelEmphasis_32_binCount | 0.44 | 0.365 | 0.532 |
| wavelet-LHH_glszm_ZoneEntropy_32_binCount | 0.55 | 0.472 | 0.637 |
| wavelet-LHH_glszm_ZonePercentage_32_binCount | 0.797 | 0.743 | 0.849 |
| wavelet-LHH_glszm_ZoneVariance_32_binCount | 0.755 | 0.693 | 0.815 |
| wavelet-LHH_gldm_DependenceEntropy_32_binCount | 0.752 | 0.69 | 0.813 |
| wavelet-LHH_gldm_DependenceNonUniformity_32_binCount | 0.788 | 0.732 | 0.842 |
| wavelet-LHH_gldm_DependenceNonUniformityNormalized_32_binCount | 0.673 | 0.602 | 0.747 |
| wavelet-LHH_gldm_DependenceVariance_32_binCount | 0.789 | 0.733 | 0.842 |
| wavelet-LHH_gldm_GrayLevelNonUniformity_32_binCount | 0.961 | 0.947 | 0.972 |
| wavelet-LHH_gldm_GrayLevelVariance_32_binCount | 0.715 | 0.648 | 0.782 |
| wavelet-LHH_gldm_HighGrayLevelEmphasis_32_binCount | 0.155 | 0.114 | 0.214 |
| wavelet-LHH_gldm_LargeDependenceEmphasis_32_binCount | 0.847 | 0.803 | 0.888 |
| wavelet-LHH_gldm_LargeDependenceHighGrayLevelEmphasis_32_binCount | 0.71 | 0.643 | 0.779 |
| wavelet-LHH_gldm_LargeDependenceLowGrayLevelEmphasis_32_binCount | 0.605 | 0.529 | 0.687 |
| wavelet-LHH_gldm_LowGrayLevelEmphasis_32_binCount | 0.294 | 0.231 | 0.377 |
| wavelet-LHH_gldm_SmallDependenceEmphasis_32_binCount | 0.787 | 0.731 | 0.841 |
| wavelet-LHH_gldm_SmallDependenceHighGrayLevelEmphasis_32_binCount | 0.687 | 0.617 | 0.759 |
| wavelet-LHH_gldm_SmallDependenceLowGrayLevelEmphasis_32_binCount | 0.732 | 0.668 | 0.797 |
| wavelet-LHH_ngtdm_Busyness_32_binCount | 0.846 | 0.801 | 0.887 |
| wavelet-LHH_ngtdm_Coarseness_32_binCount | 0.969 | 0.959 | 0.978 |
| wavelet-LHH_ngtdm_Complexity_32_binCount | 0.584 | 0.508 | 0.669 |
| wavelet-LHH_ngtdm_Contrast_32_binCount | 0.76 | 0.7 | 0.82 |
| wavelet-LHH_ngtdm_Strength_32_binCount | 0.913 | 0.885 | 0.937 |
| wavelet-HLL_firstorder_10Percentile_32_binCount | 0.829 | 0.781 | 0.874 |
| wavelet-HLL_firstorder_90Percentile_32_binCount | 0.94 | 0.92 | 0.957 |
| wavelet-HLL_firstorder_Energy_32_binCount | 0.905 | 0.876 | 0.932 |
| wavelet-HLL_firstorder_Entropy_32_binCount | 0.813 | 0.762 | 0.861 |
| wavelet-HLL_firstorder_InterquartileRange_32_binCount | 0.954 | 0.938 | 0.967 |
| wavelet-HLL_firstorder_Kurtosis_32_binCount | 0.606 | 0.53 | 0.688 |
| wavelet-HLL_firstorder_Maximum_32_binCount | 0.856 | 0.814 | 0.895 |
| wavelet-HLL_firstorder_MeanAbsoluteDeviation_32_binCount | 0.906 | 0.877 | 0.933 |
| wavelet-HLL_firstorder_Mean_32_binCount | 0.626 | 0.552 | 0.706 |
| wavelet-HLL_firstorder_Median_32_binCount | 0.747 | 0.685 | 0.809 |
| wavelet-HLL_firstorder_Minimum_32_binCount | 0.819 | 0.769 | 0.866 |
| wavelet-HLL_firstorder_Range_32_binCount | 0.869 | 0.83 | 0.904 |
| wavelet-HLL_firstorder_RobustMeanAbsoluteDeviation_32_binCount | 0.949 | 0.932 | 0.964 |
| wavelet-HLL_firstorder_RootMeanSquared_32_binCount | 0.839 | 0.793 | 0.882 |
| wavelet-HLL_firstorder_Skewness_32_binCount | 0.339 | 0.271 | 0.427 |
| wavelet-HLL_firstorder_TotalEnergy_32_binCount | 0.905 | 0.876 | 0.932 |
| wavelet-HLL_firstorder_Uniformity_32_binCount | 0.798 | 0.744 | 0.85 |
| wavelet-HLL_firstorder_Variance_32_binCount | 0.758 | 0.697 | 0.817 |
| wavelet-HLL_glcm_Autocorrelation_32_binCount | 0.283 | 0.221 | 0.364 |
| wavelet-HLL_glcm_ClusterProminence_32_binCount | 0.665 | 0.593 | 0.74 |
| wavelet-HLL_glcm_ClusterShade_32_binCount | 0.264 | 0.205 | 0.344 |
| wavelet-HLL_glcm_ClusterTendency_32_binCount | 0.74 | 0.676 | 0.803 |
| wavelet-HLL_glcm_Contrast_32_binCount | 0.737 | 0.673 | 0.8 |
| wavelet-HLL_glcm_Correlation_32_binCount | 0.627 | 0.552 | 0.707 |
| wavelet-HLL_glcm_DifferenceAverage_32_binCount | 0.788 | 0.732 | 0.842 |
| wavelet-HLL_glcm_DifferenceEntropy_32_binCount | 0.807 | 0.754 | 0.857 |
| wavelet-HLL_glcm_DifferenceVariance_32_binCount | 0.733 | 0.668 | 0.797 |
| wavelet-HLL_glcm_Id_32_binCount | 0.811 | 0.759 | 0.86 |
| wavelet-HLL_glcm_Idm_32_binCount | 0.809 | 0.757 | 0.859 |
| wavelet-HLL_glcm_Idmn_32_binCount | 0.744 | 0.681 | 0.806 |
| wavelet-HLL_glcm_Idn_32_binCount | 0.792 | 0.738 | 0.845 |
| wavelet-HLL_glcm_Imc1_32_binCount | 0.856 | 0.815 | 0.895 |
| wavelet-HLL_glcm_Imc2_32_binCount | 0.895 | 0.862 | 0.924 |
| wavelet-HLL_glcm_InverseVariance_32_binCount | 0.59 | 0.514 | 0.674 |
| wavelet-HLL_glcm_JointAverage_32_binCount | 0.292 | 0.229 | 0.375 |
| wavelet-HLL_glcm_JointEnergy_32_binCount | 0.75 | 0.688 | 0.811 |
| wavelet-HLL_glcm_JointEntropy_32_binCount | 0.802 | 0.749 | 0.853 |
| wavelet-HLL_glcm_MCC_32_binCount | 0.455 | 0.378 | 0.546 |
| wavelet-HLL_glcm_MaximumProbability_32_binCount | 0.761 | 0.7 | 0.82 |
| wavelet-HLL_glcm_SumAverage_32_binCount | 0.292 | 0.229 | 0.375 |
| wavelet-HLL_glcm_SumEntropy_32_binCount | 0.811 | 0.759 | 0.86 |
| wavelet-HLL_glcm_SumSquares_32_binCount | 0.749 | 0.687 | 0.811 |
| wavelet-HLL_glrlm_GrayLevelNonUniformity_32_binCount | 0.973 | 0.964 | 0.981 |
| wavelet-HLL_glrlm_GrayLevelNonUniformityNormalized_32_binCount | 0.8 | 0.746 | 0.851 |
| wavelet-HLL_glrlm_GrayLevelVariance_32_binCount | 0.764 | 0.705 | 0.823 |
| wavelet-HLL_glrlm_HighGrayLevelRunEmphasis_32_binCount | 0.278 | 0.217 | 0.36 |
| wavelet-HLL_glrlm_LongRunEmphasis_32_binCount | 0.709 | 0.642 | 0.777 |
| wavelet-HLL_glrlm_LongRunHighGrayLevelEmphasis_32_binCount | 0.66 | 0.587 | 0.735 |
| wavelet-HLL_glrlm_LongRunLowGrayLevelEmphasis_32_binCount | 0.199 | 0.15 | 0.268 |
| wavelet-HLL_glrlm_LowGrayLevelRunEmphasis_32_binCount | 0.251 | 0.194 | 0.328 |
| wavelet-HLL_glrlm_RunEntropy_32_binCount | 0.653 | 0.58 | 0.73 |
| wavelet-HLL_glrlm_RunLengthNonUniformity_32_binCount | 0.853 | 0.811 | 0.893 |
| wavelet-HLL_glrlm_RunLengthNonUniformityNormalized_32_binCount | 0.842 | 0.797 | 0.884 |
| wavelet-HLL_glrlm_RunPercentage_32_binCount | 0.852 | 0.81 | 0.892 |
| wavelet-HLL_glrlm_RunVariance_32_binCount | 0.677 | 0.606 | 0.75 |
| wavelet-HLL_glrlm_ShortRunEmphasis_32_binCount | 0.843 | 0.798 | 0.885 |
| wavelet-HLL_glrlm_ShortRunHighGrayLevelEmphasis_32_binCount | 0.311 | 0.246 | 0.396 |
| wavelet-HLL_glrlm_ShortRunLowGrayLevelEmphasis_32_binCount | 0.32 | 0.254 | 0.406 |
| wavelet-HLL_glszm_GrayLevelNonUniformity_32_binCount | 0.899 | 0.868 | 0.927 |
| wavelet-HLL_glszm_GrayLevelNonUniformityNormalized_32_binCount | 0.695 | 0.625 | 0.765 |
| wavelet-HLL_glszm_GrayLevelVariance_32_binCount | 0.711 | 0.644 | 0.779 |
| wavelet-HLL_glszm_HighGrayLevelZoneEmphasis_32_binCount | 0.27 | 0.21 | 0.351 |
| wavelet-HLL_glszm_LargeAreaEmphasis_32_binCount | 0.81 | 0.758 | 0.859 |
| wavelet-HLL_glszm_LargeAreaHighGrayLevelEmphasis_32_binCount | 0.741 | 0.677 | 0.804 |
| wavelet-HLL_glszm_LargeAreaLowGrayLevelEmphasis_32_binCount | 0.125 | 0.09 | 0.177 |
| wavelet-HLL_glszm_LowGrayLevelZoneEmphasis_32_binCount | 0.261 | 0.202 | 0.34 |
| wavelet-HLL_glszm_SizeZoneNonUniformity_32_binCount | 0.584 | 0.507 | 0.668 |
| wavelet-HLL_glszm_SizeZoneNonUniformityNormalized_32_binCount | 0.686 | 0.616 | 0.758 |
| wavelet-HLL_glszm_SmallAreaEmphasis_32_binCount | 0.675 | 0.604 | 0.749 |
| wavelet-HLL_glszm_SmallAreaHighGrayLevelEmphasis_32_binCount | 0.29 | 0.228 | 0.373 |
| wavelet-HLL_glszm_SmallAreaLowGrayLevelEmphasis_32_binCount | 0.381 | 0.309 | 0.471 |
| wavelet-HLL_glszm_ZoneEntropy_32_binCount | 0.685 | 0.615 | 0.757 |
| wavelet-HLL_glszm_ZonePercentage_32_binCount | 0.822 | 0.773 | 0.869 |
| wavelet-HLL_glszm_ZoneVariance_32_binCount | 0.811 | 0.76 | 0.86 |
| wavelet-HLL_gldm_DependenceEntropy_32_binCount | 0.765 | 0.705 | 0.823 |
| wavelet-HLL_gldm_DependenceNonUniformity_32_binCount | 0.764 | 0.705 | 0.823 |
| wavelet-HLL_gldm_DependenceNonUniformityNormalized_32_binCount | 0.714 | 0.647 | 0.781 |
| wavelet-HLL_gldm_DependenceVariance_32_binCount | 0.798 | 0.744 | 0.85 |
| wavelet-HLL_gldm_GrayLevelNonUniformity_32_binCount | 0.946 | 0.928 | 0.961 |
| wavelet-HLL_gldm_GrayLevelVariance_32_binCount | 0.763 | 0.703 | 0.822 |
| wavelet-HLL_gldm_HighGrayLevelEmphasis_32_binCount | 0.279 | 0.218 | 0.36 |
| wavelet-HLL_gldm_LargeDependenceEmphasis_32_binCount | 0.84 | 0.795 | 0.883 |
| wavelet-HLL_gldm_LargeDependenceHighGrayLevelEmphasis_32_binCount | 0.737 | 0.673 | 0.8 |
| wavelet-HLL_gldm_LargeDependenceLowGrayLevelEmphasis_32_binCount | 0.185 | 0.139 | 0.251 |
| wavelet-HLL_gldm_LowGrayLevelEmphasis_32_binCount | 0.247 | 0.191 | 0.325 |
| wavelet-HLL_gldm_SmallDependenceEmphasis_32_binCount | 0.811 | 0.76 | 0.86 |
| wavelet-HLL_gldm_SmallDependenceHighGrayLevelEmphasis_32_binCount | 0.716 | 0.649 | 0.783 |
| wavelet-HLL_gldm_SmallDependenceLowGrayLevelEmphasis_32_binCount | 0.684 | 0.614 | 0.756 |
| wavelet-HLL_ngtdm_Busyness_32_binCount | 0.678 | 0.607 | 0.751 |
| wavelet-HLL_ngtdm_Coarseness_32_binCount | 0.963 | 0.95 | 0.974 |
| wavelet-HLL_ngtdm_Complexity_32_binCount | 0.654 | 0.581 | 0.731 |
| wavelet-HLL_ngtdm_Contrast_32_binCount | 0.764 | 0.704 | 0.822 |
| wavelet-HLL_ngtdm_Strength_32_binCount | 0.89 | 0.856 | 0.921 |
| wavelet-HLH_firstorder_10Percentile_32_binCount | 0.927 | 0.904 | 0.948 |
| wavelet-HLH_firstorder_90Percentile_32_binCount | 0.921 | 0.896 | 0.943 |
| wavelet-HLH_firstorder_Energy_32_binCount | 0.932 | 0.91 | 0.952 |
| wavelet-HLH_firstorder_Entropy_32_binCount | 0.83 | 0.783 | 0.875 |
| wavelet-HLH_firstorder_InterquartileRange_32_binCount | 0.97 | 0.959 | 0.979 |
| wavelet-HLH_firstorder_Kurtosis_32_binCount | 0.517 | 0.439 | 0.606 |
| wavelet-HLH_firstorder_Maximum_32_binCount | 0.79 | 0.734 | 0.843 |
| wavelet-HLH_firstorder_MeanAbsoluteDeviation_32_binCount | 0.925 | 0.901 | 0.946 |
| wavelet-HLH_firstorder_Mean_32_binCount | 0.32 | 0.254 | 0.405 |
| wavelet-HLH_firstorder_Median_32_binCount | 0.525 | 0.447 | 0.614 |
| wavelet-HLH_firstorder_Minimum_32_binCount | 0.844 | 0.8 | 0.886 |
| wavelet-HLH_firstorder_Range_32_binCount | 0.85 | 0.806 | 0.89 |
| wavelet-HLH_firstorder_RobustMeanAbsoluteDeviation_32_binCount | 0.966 | 0.954 | 0.976 |
| wavelet-HLH_firstorder_RootMeanSquared_32_binCount | 0.888 | 0.853 | 0.919 |
| wavelet-HLH_firstorder_Skewness_32_binCount | 0.228 | 0.174 | 0.302 |
| wavelet-HLH_firstorder_TotalEnergy_32_binCount | 0.932 | 0.91 | 0.952 |
| wavelet-HLH_firstorder_Uniformity_32_binCount | 0.814 | 0.763 | 0.862 |
| wavelet-HLH_firstorder_Variance_32_binCount | 0.83 | 0.782 | 0.874 |
| wavelet-HLH_glcm_Autocorrelation_32_binCount | 0.204 | 0.154 | 0.274 |
| wavelet-HLH_glcm_ClusterProminence_32_binCount | 0.698 | 0.629 | 0.768 |
| wavelet-HLH_glcm_ClusterShade_32_binCount | 0.349 | 0.28 | 0.438 |
| wavelet-HLH_glcm_ClusterTendency_32_binCount | 0.746 | 0.684 | 0.808 |
| wavelet-HLH_glcm_Contrast_32_binCount | 0.744 | 0.681 | 0.806 |
| wavelet-HLH_glcm_Correlation_32_binCount | 0.674 | 0.603 | 0.748 |
| wavelet-HLH_glcm_DifferenceAverage_32_binCount | 0.796 | 0.741 | 0.848 |
| wavelet-HLH_glcm_DifferenceEntropy_32_binCount | 0.821 | 0.771 | 0.868 |
| wavelet-HLH_glcm_DifferenceVariance_32_binCount | 0.755 | 0.694 | 0.816 |
| wavelet-HLH_glcm_Id_32_binCount | 0.831 | 0.783 | 0.875 |
| wavelet-HLH_glcm_Idm_32_binCount | 0.83 | 0.782 | 0.875 |
| wavelet-HLH_glcm_Idmn_32_binCount | 0.748 | 0.685 | 0.809 |
| wavelet-HLH_glcm_Idn_32_binCount | 0.802 | 0.749 | 0.853 |
| wavelet-HLH_glcm_Imc1_32_binCount | 0.868 | 0.829 | 0.904 |
| wavelet-HLH_glcm_Imc2_32_binCount | 0.884 | 0.848 | 0.916 |
| wavelet-HLH_glcm_InverseVariance_32_binCount | 0.57 | 0.492 | 0.655 |
| wavelet-HLH_glcm_JointAverage_32_binCount | 0.194 | 0.146 | 0.262 |
| wavelet-HLH_glcm_JointEnergy_32_binCount | 0.753 | 0.692 | 0.814 |
| wavelet-HLH_glcm_JointEntropy_32_binCount | 0.818 | 0.768 | 0.865 |
| wavelet-HLH_glcm_MCC_32_binCount | 0.52 | 0.442 | 0.609 |
| wavelet-HLH_glcm_MaximumProbability_32_binCount | 0.774 | 0.715 | 0.83 |
| wavelet-HLH_glcm_SumAverage_32_binCount | 0.194 | 0.146 | 0.262 |
| wavelet-HLH_glcm_SumEntropy_32_binCount | 0.822 | 0.773 | 0.869 |
| wavelet-HLH_glcm_SumSquares_32_binCount | 0.75 | 0.688 | 0.811 |
| wavelet-HLH_glrlm_GrayLevelNonUniformity_32_binCount | 0.968 | 0.958 | 0.978 |
| wavelet-HLH_glrlm_GrayLevelNonUniformityNormalized_32_binCount | 0.817 | 0.767 | 0.865 |
| wavelet-HLH_glrlm_GrayLevelVariance_32_binCount | 0.768 | 0.709 | 0.826 |
| wavelet-HLH_glrlm_HighGrayLevelRunEmphasis_32_binCount | 0.207 | 0.157 | 0.277 |
| wavelet-HLH_glrlm_LongRunEmphasis_32_binCount | 0.652 | 0.579 | 0.729 |
| wavelet-HLH_glrlm_LongRunHighGrayLevelEmphasis_32_binCount | 0.518 | 0.44 | 0.607 |
| wavelet-HLH_glrlm_LongRunLowGrayLevelEmphasis_32_binCount | 0.612 | 0.536 | 0.694 |
| wavelet-HLH_glrlm_LowGrayLevelRunEmphasis_32_binCount | 0.294 | 0.231 | 0.378 |
| wavelet-HLH_glrlm_RunEntropy_32_binCount | 0.647 | 0.574 | 0.725 |
| wavelet-HLH_glrlm_RunLengthNonUniformity_32_binCount | 0.889 | 0.855 | 0.92 |
| wavelet-HLH_glrlm_RunLengthNonUniformityNormalized_32_binCount | 0.862 | 0.821 | 0.899 |
| wavelet-HLH_glrlm_RunPercentage_32_binCount | 0.868 | 0.829 | 0.904 |
| wavelet-HLH_glrlm_RunVariance_32_binCount | 0.624 | 0.549 | 0.704 |
| wavelet-HLH_glrlm_ShortRunEmphasis_32_binCount | 0.864 | 0.824 | 0.901 |
| wavelet-HLH_glrlm_ShortRunHighGrayLevelEmphasis_32_binCount | 0.347 | 0.278 | 0.435 |
| wavelet-HLH_glrlm_ShortRunLowGrayLevelEmphasis_32_binCount | 0.414 | 0.339 | 0.505 |
| wavelet-HLH_glszm_GrayLevelNonUniformity_32_binCount | 0.906 | 0.876 | 0.932 |
| wavelet-HLH_glszm_GrayLevelNonUniformityNormalized_32_binCount | 0.714 | 0.647 | 0.781 |
| wavelet-HLH_glszm_GrayLevelVariance_32_binCount | 0.726 | 0.661 | 0.792 |
| wavelet-HLH_glszm_HighGrayLevelZoneEmphasis_32_binCount | 0.21 | 0.159 | 0.281 |
| wavelet-HLH_glszm_LargeAreaEmphasis_32_binCount | 0.846 | 0.802 | 0.887 |
| wavelet-HLH_glszm_LargeAreaHighGrayLevelEmphasis_32_binCount | 0.605 | 0.529 | 0.687 |
| wavelet-HLH_glszm_LargeAreaLowGrayLevelEmphasis_32_binCount | 0.783 | 0.726 | 0.838 |
| wavelet-HLH_glszm_LowGrayLevelZoneEmphasis_32_binCount | 0.364 | 0.294 | 0.454 |
| wavelet-HLH_glszm_SizeZoneNonUniformity_32_binCount | 0.719 | 0.653 | 0.786 |
| wavelet-HLH_glszm_SizeZoneNonUniformityNormalized_32_binCount | 0.654 | 0.581 | 0.73 |
| wavelet-HLH_glszm_SmallAreaEmphasis_32_binCount | 0.647 | 0.574 | 0.725 |
| wavelet-HLH_glszm_SmallAreaHighGrayLevelEmphasis_32_binCount | 0.29 | 0.228 | 0.373 |
| wavelet-HLH_glszm_SmallAreaLowGrayLevelEmphasis_32_binCount | 0.46 | 0.384 | 0.551 |
| wavelet-HLH_glszm_ZoneEntropy_32_binCount | 0.568 | 0.49 | 0.654 |
| wavelet-HLH_glszm_ZonePercentage_32_binCount | 0.839 | 0.794 | 0.882 |
| wavelet-HLH_glszm_ZoneVariance_32_binCount | 0.846 | 0.802 | 0.888 |
| wavelet-HLH_gldm_DependenceEntropy_32_binCount | 0.753 | 0.692 | 0.814 |
| wavelet-HLH_gldm_DependenceNonUniformity_32_binCount | 0.74 | 0.677 | 0.803 |
| wavelet-HLH_gldm_DependenceNonUniformityNormalized_32_binCount | 0.74 | 0.676 | 0.803 |
| wavelet-HLH_gldm_DependenceVariance_32_binCount | 0.796 | 0.742 | 0.848 |
| wavelet-HLH_gldm_GrayLevelNonUniformity_32_binCount | 0.952 | 0.936 | 0.966 |
| wavelet-HLH_gldm_GrayLevelVariance_32_binCount | 0.77 | 0.712 | 0.828 |
| wavelet-HLH_gldm_HighGrayLevelEmphasis_32_binCount | 0.207 | 0.157 | 0.277 |
| wavelet-HLH_gldm_LargeDependenceEmphasis_32_binCount | 0.848 | 0.804 | 0.889 |
| wavelet-HLH_gldm_LargeDependenceHighGrayLevelEmphasis_32_binCount | 0.657 | 0.584 | 0.733 |
| wavelet-HLH_gldm_LargeDependenceLowGrayLevelEmphasis_32_binCount | 0.696 | 0.628 | 0.767 |
| wavelet-HLH_gldm_LowGrayLevelEmphasis_32_binCount | 0.289 | 0.226 | 0.371 |
| wavelet-HLH_gldm_SmallDependenceEmphasis_32_binCount | 0.83 | 0.782 | 0.875 |
| wavelet-HLH_gldm_SmallDependenceHighGrayLevelEmphasis_32_binCount | 0.744 | 0.681 | 0.806 |
| wavelet-HLH_gldm_SmallDependenceLowGrayLevelEmphasis_32_binCount | 0.745 | 0.682 | 0.807 |
| wavelet-HLH_ngtdm_Busyness_32_binCount | 0.807 | 0.755 | 0.857 |
| wavelet-HLH_ngtdm_Coarseness_32_binCount | 0.967 | 0.956 | 0.977 |
| wavelet-HLH_ngtdm_Complexity_32_binCount | 0.645 | 0.572 | 0.723 |
| wavelet-HLH_ngtdm_Contrast_32_binCount | 0.787 | 0.731 | 0.841 |
| wavelet-HLH_ngtdm_Strength_32_binCount | 0.91 | 0.881 | 0.935 |
| wavelet-HHL_firstorder_10Percentile_32_binCount | 0.832 | 0.785 | 0.876 |
| wavelet-HHL_firstorder_90Percentile_32_binCount | 0.813 | 0.761 | 0.861 |
| wavelet-HHL_firstorder_Energy_32_binCount | 0.879 | 0.842 | 0.912 |
| wavelet-HHL_firstorder_Entropy_32_binCount | 0.776 | 0.719 | 0.832 |
| wavelet-HHL_firstorder_InterquartileRange_32_binCount | 0.893 | 0.86 | 0.923 |
| wavelet-HHL_firstorder_Kurtosis_32_binCount | 0.522 | 0.444 | 0.611 |
| wavelet-HHL_firstorder_Maximum_32_binCount | 0.816 | 0.765 | 0.864 |
| wavelet-HHL_firstorder_MeanAbsoluteDeviation_32_binCount | 0.828 | 0.78 | 0.873 |
| wavelet-HHL_firstorder_Mean_32_binCount | 0.052 | 0.033 | 0.082 |
| wavelet-HHL_firstorder_Median_32_binCount | 0.297 | 0.234 | 0.381 |
| wavelet-HHL_firstorder_Minimum_32_binCount | 0.803 | 0.751 | 0.854 |
| wavelet-HHL_firstorder_Range_32_binCount | 0.844 | 0.8 | 0.886 |
| wavelet-HHL_firstorder_RobustMeanAbsoluteDeviation_32_binCount | 0.884 | 0.849 | 0.916 |
| wavelet-HHL_firstorder_RootMeanSquared_32_binCount | 0.796 | 0.742 | 0.848 |
| wavelet-HHL_firstorder_Skewness_32_binCount | 0.078 | 0.053 | 0.117 |
| wavelet-HHL_firstorder_TotalEnergy_32_binCount | 0.879 | 0.842 | 0.912 |
| wavelet-HHL_firstorder_Uniformity_32_binCount | 0.743 | 0.68 | 0.806 |
| wavelet-HHL_firstorder_Variance_32_binCount | 0.708 | 0.641 | 0.777 |
| wavelet-HHL_glcm_Autocorrelation_32_binCount | 0.098 | 0.068 | 0.142 |
| wavelet-HHL_glcm_ClusterProminence_32_binCount | 0.622 | 0.547 | 0.703 |
| wavelet-HHL_glcm_ClusterShade_32_binCount | 0.027 | 0.014 | 0.049 |
| wavelet-HHL_glcm_ClusterTendency_32_binCount | 0.68 | 0.61 | 0.753 |
| wavelet-HHL_glcm_Contrast_32_binCount | 0.711 | 0.644 | 0.779 |
| wavelet-HHL_glcm_Correlation_32_binCount | 0.382 | 0.31 | 0.472 |
| wavelet-HHL_glcm_DifferenceAverage_32_binCount | 0.754 | 0.693 | 0.815 |
| wavelet-HHL_glcm_DifferenceEntropy_32_binCount | 0.771 | 0.712 | 0.828 |
| wavelet-HHL_glcm_DifferenceVariance_32_binCount | 0.731 | 0.666 | 0.795 |
| wavelet-HHL_glcm_Id_32_binCount | 0.773 | 0.715 | 0.83 |
| wavelet-HHL_glcm_Idm_32_binCount | 0.775 | 0.717 | 0.831 |
| wavelet-HHL_glcm_Idmn_32_binCount | 0.712 | 0.645 | 0.78 |
| wavelet-HHL_glcm_Idn_32_binCount | 0.759 | 0.698 | 0.818 |
| wavelet-HHL_glcm_Imc1_32_binCount | 0.733 | 0.668 | 0.797 |
| wavelet-HHL_glcm_Imc2_32_binCount | 0.868 | 0.829 | 0.904 |
| wavelet-HHL_glcm_InverseVariance_32_binCount | 0.503 | 0.425 | 0.593 |
| wavelet-HHL_glcm_JointAverage_32_binCount | 0.099 | 0.069 | 0.144 |
| wavelet-HHL_glcm_JointEnergy_32_binCount | 0.683 | 0.613 | 0.756 |
| wavelet-HHL_glcm_JointEntropy_32_binCount | 0.767 | 0.707 | 0.825 |
| wavelet-HHL_glcm_MCC_32_binCount | 0.401 | 0.328 | 0.492 |
| wavelet-HHL_glcm_MaximumProbability_32_binCount | 0.7 | 0.632 | 0.77 |
| wavelet-HHL_glcm_SumAverage_32_binCount | 0.099 | 0.069 | 0.144 |
| wavelet-HHL_glcm_SumEntropy_32_binCount | 0.769 | 0.71 | 0.827 |
| wavelet-HHL_glcm_SumSquares_32_binCount | 0.702 | 0.634 | 0.772 |
| wavelet-HHL_glrlm_GrayLevelNonUniformity_32_binCount | 0.935 | 0.914 | 0.954 |
| wavelet-HHL_glrlm_GrayLevelNonUniformityNormalized_32_binCount | 0.727 | 0.662 | 0.792 |
| wavelet-HHL_glrlm_GrayLevelVariance_32_binCount | 0.716 | 0.649 | 0.783 |
| wavelet-HHL_glrlm_HighGrayLevelRunEmphasis_32_binCount | 0.104 | 0.073 | 0.15 |
| wavelet-HHL_glrlm_LongRunEmphasis_32_binCount | 0.644 | 0.571 | 0.722 |
| wavelet-HHL_glrlm_LongRunHighGrayLevelEmphasis_32_binCount | 0.565 | 0.488 | 0.651 |
| wavelet-HHL_glrlm_LongRunLowGrayLevelEmphasis_32_binCount | 0.547 | 0.469 | 0.634 |
| wavelet-HHL_glrlm_LowGrayLevelRunEmphasis_32_binCount | 0.167 | 0.124 | 0.23 |
| wavelet-HHL_glrlm_RunEntropy_32_binCount | 0.486 | 0.409 | 0.577 |
| wavelet-HHL_glrlm_RunLengthNonUniformity_32_binCount | 0.792 | 0.737 | 0.845 |
| wavelet-HHL_glrlm_RunLengthNonUniformityNormalized_32_binCount | 0.811 | 0.759 | 0.86 |
| wavelet-HHL_glrlm_RunPercentage_32_binCount | 0.817 | 0.767 | 0.865 |
| wavelet-HHL_glrlm_RunVariance_32_binCount | 0.65 | 0.577 | 0.727 |
| wavelet-HHL_glrlm_ShortRunEmphasis_32_binCount | 0.802 | 0.748 | 0.853 |
| wavelet-HHL_glrlm_ShortRunHighGrayLevelEmphasis_32_binCount | 0.313 | 0.248 | 0.398 |
| wavelet-HHL_glrlm_ShortRunLowGrayLevelEmphasis_32_binCount | 0.271 | 0.211 | 0.351 |
| wavelet-HHL_glszm_GrayLevelNonUniformity_32_binCount | 0.873 | 0.835 | 0.908 |
| wavelet-HHL_glszm_GrayLevelNonUniformityNormalized_32_binCount | 0.595 | 0.519 | 0.679 |
| wavelet-HHL_glszm_GrayLevelVariance_32_binCount | 0.611 | 0.535 | 0.692 |
| wavelet-HHL_glszm_HighGrayLevelZoneEmphasis_32_binCount | 0.11 | 0.078 | 0.158 |
| wavelet-HHL_glszm_LargeAreaEmphasis_32_binCount | 0.837 | 0.791 | 0.881 |
| wavelet-HHL_glszm_LargeAreaHighGrayLevelEmphasis_32_binCount | 0.752 | 0.691 | 0.813 |
| wavelet-HHL_glszm_LargeAreaLowGrayLevelEmphasis_32_binCount | 0.763 | 0.702 | 0.821 |
| wavelet-HHL_glszm_LowGrayLevelZoneEmphasis_32_binCount | 0.239 | 0.183 | 0.314 |
| wavelet-HHL_glszm_SizeZoneNonUniformity_32_binCount | 0.642 | 0.568 | 0.72 |
| wavelet-HHL_glszm_SizeZoneNonUniformityNormalized_32_binCount | 0.538 | 0.461 | 0.627 |
| wavelet-HHL_glszm_SmallAreaEmphasis_32_binCount | 0.529 | 0.451 | 0.617 |
| wavelet-HHL_glszm_SmallAreaHighGrayLevelEmphasis_32_binCount | 0.213 | 0.162 | 0.285 |
| wavelet-HHL_glszm_SmallAreaLowGrayLevelEmphasis_32_binCount | 0.362 | 0.291 | 0.451 |
| wavelet-HHL_glszm_ZoneEntropy_32_binCount | 0.532 | 0.454 | 0.621 |
| wavelet-HHL_glszm_ZonePercentage_32_binCount | 0.787 | 0.731 | 0.841 |
| wavelet-HHL_glszm_ZoneVariance_32_binCount | 0.838 | 0.792 | 0.881 |
| wavelet-HHL_gldm_DependenceEntropy_32_binCount | 0.715 | 0.649 | 0.783 |
| wavelet-HHL_gldm_DependenceNonUniformity_32_binCount | 0.678 | 0.607 | 0.751 |
| wavelet-HHL_gldm_DependenceNonUniformityNormalized_32_binCount | 0.54 | 0.462 | 0.628 |
| wavelet-HHL_gldm_DependenceVariance_32_binCount | 0.698 | 0.629 | 0.768 |
| wavelet-HHL_gldm_GrayLevelNonUniformity_32_binCount | 0.944 | 0.926 | 0.96 |
| wavelet-HHL_gldm_GrayLevelVariance_32_binCount | 0.718 | 0.652 | 0.785 |
| wavelet-HHL_gldm_HighGrayLevelEmphasis_32_binCount | 0.103 | 0.073 | 0.149 |
| wavelet-HHL_gldm_LargeDependenceEmphasis_32_binCount | 0.788 | 0.732 | 0.842 |
| wavelet-HHL_gldm_LargeDependenceHighGrayLevelEmphasis_32_binCount | 0.582 | 0.505 | 0.666 |
| wavelet-HHL_gldm_LargeDependenceLowGrayLevelEmphasis_32_binCount | 0.377 | 0.305 | 0.467 |
| wavelet-HHL_gldm_LowGrayLevelEmphasis_32_binCount | 0.165 | 0.122 | 0.227 |
| wavelet-HHL_gldm_SmallDependenceEmphasis_32_binCount | 0.78 | 0.723 | 0.836 |
| wavelet-HHL_gldm_SmallDependenceHighGrayLevelEmphasis_32_binCount | 0.68 | 0.609 | 0.753 |
| wavelet-HHL_gldm_SmallDependenceLowGrayLevelEmphasis_32_binCount | 0.743 | 0.68 | 0.806 |
| wavelet-HHL_ngtdm_Busyness_32_binCount | 0.593 | 0.517 | 0.677 |
| wavelet-HHL_ngtdm_Coarseness_32_binCount | 0.951 | 0.934 | 0.965 |
| wavelet-HHL_ngtdm_Complexity_32_binCount | 0.604 | 0.528 | 0.687 |
| wavelet-HHL_ngtdm_Contrast_32_binCount | 0.743 | 0.68 | 0.806 |
| wavelet-HHL_ngtdm_Strength_32_binCount | 0.814 | 0.763 | 0.863 |
| wavelet-HHH_firstorder_10Percentile_32_binCount | 0.862 | 0.822 | 0.899 |
| wavelet-HHH_firstorder_90Percentile_32_binCount | 0.872 | 0.834 | 0.907 |
| wavelet-HHH_firstorder_Energy_32_binCount | 0.907 | 0.878 | 0.933 |
| wavelet-HHH_firstorder_Entropy_32_binCount | 0.801 | 0.748 | 0.852 |
| wavelet-HHH_firstorder_InterquartileRange_32_binCount | 0.915 | 0.888 | 0.939 |
| wavelet-HHH_firstorder_Kurtosis_32_binCount | 0.47 | 0.393 | 0.561 |
| wavelet-HHH_firstorder_Maximum_32_binCount | 0.783 | 0.726 | 0.838 |
| wavelet-HHH_firstorder_MeanAbsoluteDeviation_32_binCount | 0.875 | 0.837 | 0.909 |
| wavelet-HHH_firstorder_Mean_32_binCount | 0.08 | 0.054 | 0.119 |
| wavelet-HHH_firstorder_Median_32_binCount | 0.219 | 0.167 | 0.292 |
| wavelet-HHH_firstorder_Minimum_32_binCount | 0.804 | 0.751 | 0.854 |
| wavelet-HHH_firstorder_Range_32_binCount | 0.824 | 0.775 | 0.87 |
| wavelet-HHH_firstorder_RobustMeanAbsoluteDeviation_32_binCount | 0.902 | 0.871 | 0.929 |
| wavelet-HHH_firstorder_RootMeanSquared_32_binCount | 0.855 | 0.813 | 0.894 |
| wavelet-HHH_firstorder_Skewness_32_binCount | 0.062 | 0.04 | 0.095 |
| wavelet-HHH_firstorder_TotalEnergy_32_binCount | 0.907 | 0.878 | 0.933 |
| wavelet-HHH_firstorder_Uniformity_32_binCount | 0.764 | 0.704 | 0.822 |
| wavelet-HHH_firstorder_Variance_32_binCount | 0.733 | 0.668 | 0.797 |
| wavelet-HHH_glcm_Autocorrelation_32_binCount | 0.059 | 0.038 | 0.091 |
| wavelet-HHH_glcm_ClusterProminence_32_binCount | 0.635 | 0.561 | 0.714 |
| wavelet-HHH_glcm_ClusterShade_32_binCount | 0.027 | 0.013 | 0.047 |
| wavelet-HHH_glcm_ClusterTendency_32_binCount | 0.692 | 0.622 | 0.763 |
| wavelet-HHH_glcm_Contrast_32_binCount | 0.726 | 0.661 | 0.792 |
| wavelet-HHH_glcm_Correlation_32_binCount | 0.492 | 0.414 | 0.582 |
| wavelet-HHH_glcm_DifferenceAverage_32_binCount | 0.767 | 0.708 | 0.825 |
| wavelet-HHH_glcm_DifferenceEntropy_32_binCount | 0.793 | 0.738 | 0.845 |
| wavelet-HHH_glcm_DifferenceVariance_32_binCount | 0.755 | 0.694 | 0.816 |
| wavelet-HHH_glcm_Id_32_binCount | 0.791 | 0.736 | 0.844 |
| wavelet-HHH_glcm_Idm_32_binCount | 0.794 | 0.739 | 0.846 |
| wavelet-HHH_glcm_Idmn_32_binCount | 0.725 | 0.66 | 0.791 |
| wavelet-HHH_glcm_Idn_32_binCount | 0.772 | 0.714 | 0.829 |
| wavelet-HHH_glcm_Imc1_32_binCount | 0.777 | 0.719 | 0.833 |
| wavelet-HHH_glcm_Imc2_32_binCount | 0.875 | 0.838 | 0.909 |
| wavelet-HHH_glcm_InverseVariance_32_binCount | 0.506 | 0.428 | 0.596 |
| wavelet-HHH_glcm_JointAverage_32_binCount | 0.059 | 0.038 | 0.091 |
| wavelet-HHH_glcm_JointEnergy_32_binCount | 0.703 | 0.635 | 0.772 |
| wavelet-HHH_glcm_JointEntropy_32_binCount | 0.786 | 0.73 | 0.84 |
| wavelet-HHH_glcm_MCC_32_binCount | 0.514 | 0.436 | 0.603 |
| wavelet-HHH_glcm_MaximumProbability_32_binCount | 0.709 | 0.642 | 0.778 |
| wavelet-HHH_glcm_SumAverage_32_binCount | 0.059 | 0.038 | 0.091 |
| wavelet-HHH_glcm_SumEntropy_32_binCount | 0.787 | 0.731 | 0.841 |
| wavelet-HHH_glcm_SumSquares_32_binCount | 0.712 | 0.645 | 0.78 |
| wavelet-HHH_glrlm_GrayLevelNonUniformity_32_binCount | 0.937 | 0.917 | 0.955 |
| wavelet-HHH_glrlm_GrayLevelNonUniformityNormalized_32_binCount | 0.762 | 0.702 | 0.821 |
| wavelet-HHH_glrlm_GrayLevelVariance_32_binCount | 0.742 | 0.679 | 0.805 |
| wavelet-HHH_glrlm_HighGrayLevelRunEmphasis_32_binCount | 0.059 | 0.038 | 0.091 |
| wavelet-HHH_glrlm_LongRunEmphasis_32_binCount | 0.737 | 0.674 | 0.801 |
| wavelet-HHH_glrlm_LongRunHighGrayLevelEmphasis_32_binCount | 0.682 | 0.612 | 0.755 |
| wavelet-HHH_glrlm_LongRunLowGrayLevelEmphasis_32_binCount | 0.661 | 0.589 | 0.737 |
| wavelet-HHH_glrlm_LowGrayLevelRunEmphasis_32_binCount | 0.355 | 0.286 | 0.444 |
| wavelet-HHH_glrlm_RunEntropy_32_binCount | 0.544 | 0.466 | 0.632 |
| wavelet-HHH_glrlm_RunLengthNonUniformity_32_binCount | 0.836 | 0.79 | 0.879 |
| wavelet-HHH_glrlm_RunLengthNonUniformityNormalized_32_binCount | 0.83 | 0.782 | 0.875 |
| wavelet-HHH_glrlm_RunPercentage_32_binCount | 0.828 | 0.78 | 0.873 |
| wavelet-HHH_glrlm_RunVariance_32_binCount | 0.753 | 0.691 | 0.813 |
| wavelet-HHH_glrlm_ShortRunEmphasis_32_binCount | 0.821 | 0.772 | 0.868 |
| wavelet-HHH_glrlm_ShortRunHighGrayLevelEmphasis_32_binCount | 0.256 | 0.198 | 0.334 |
| wavelet-HHH_glrlm_ShortRunLowGrayLevelEmphasis_32_binCount | 0.52 | 0.442 | 0.609 |
| wavelet-HHH_glszm_GrayLevelNonUniformity_32_binCount | 0.894 | 0.861 | 0.923 |
| wavelet-HHH_glszm_GrayLevelNonUniformityNormalized_32_binCount | 0.632 | 0.557 | 0.711 |
| wavelet-HHH_glszm_GrayLevelVariance_32_binCount | 0.648 | 0.575 | 0.725 |
| wavelet-HHH_glszm_HighGrayLevelZoneEmphasis_32_binCount | 0.062 | 0.04 | 0.095 |
| wavelet-HHH_glszm_LargeAreaEmphasis_32_binCount | 0.692 | 0.623 | 0.763 |
| wavelet-HHH_glszm_LargeAreaHighGrayLevelEmphasis_32_binCount | 0.739 | 0.675 | 0.802 |
| wavelet-HHH_glszm_LargeAreaLowGrayLevelEmphasis_32_binCount | 0.59 | 0.514 | 0.674 |
| wavelet-HHH_glszm_LowGrayLevelZoneEmphasis_32_binCount | 0.417 | 0.343 | 0.508 |
| wavelet-HHH_glszm_SizeZoneNonUniformity_32_binCount | 0.798 | 0.745 | 0.85 |
| wavelet-HHH_glszm_SizeZoneNonUniformityNormalized_32_binCount | 0.489 | 0.412 | 0.58 |
| wavelet-HHH_glszm_SmallAreaEmphasis_32_binCount | 0.487 | 0.41 | 0.578 |
| wavelet-HHH_glszm_SmallAreaHighGrayLevelEmphasis_32_binCount | 0.116 | 0.083 | 0.165 |
| wavelet-HHH_glszm_SmallAreaLowGrayLevelEmphasis_32_binCount | 0.463 | 0.386 | 0.554 |
| wavelet-HHH_glszm_ZoneEntropy_32_binCount | 0.409 | 0.335 | 0.499 |
| wavelet-HHH_glszm_ZonePercentage_32_binCount | 0.82 | 0.77 | 0.867 |
| wavelet-HHH_glszm_ZoneVariance_32_binCount | 0.694 | 0.625 | 0.765 |
| wavelet-HHH_gldm_DependenceEntropy_32_binCount | 0.74 | 0.676 | 0.803 |
| wavelet-HHH_gldm_DependenceNonUniformity_32_binCount | 0.75 | 0.688 | 0.811 |
| wavelet-HHH_gldm_DependenceNonUniformityNormalized_32_binCount | 0.64 | 0.566 | 0.718 |
| wavelet-HHH_gldm_DependenceVariance_32_binCount | 0.679 | 0.608 | 0.752 |
| wavelet-HHH_gldm_GrayLevelNonUniformity_32_binCount | 0.96 | 0.946 | 0.971 |
| wavelet-HHH_gldm_GrayLevelVariance_32_binCount | 0.746 | 0.683 | 0.808 |
| wavelet-HHH_gldm_HighGrayLevelEmphasis_32_binCount | 0.059 | 0.038 | 0.091 |
| wavelet-HHH_gldm_LargeDependenceEmphasis_32_binCount | 0.796 | 0.741 | 0.848 |
| wavelet-HHH_gldm_LargeDependenceHighGrayLevelEmphasis_32_binCount | 0.684 | 0.614 | 0.756 |
| wavelet-HHH_gldm_LargeDependenceLowGrayLevelEmphasis_32_binCount | 0.551 | 0.473 | 0.638 |
| wavelet-HHH_gldm_LowGrayLevelEmphasis_32_binCount | 0.342 | 0.274 | 0.43 |
| wavelet-HHH_gldm_SmallDependenceEmphasis_32_binCount | 0.814 | 0.763 | 0.862 |
| wavelet-HHH_gldm_SmallDependenceHighGrayLevelEmphasis_32_binCount | 0.7 | 0.632 | 0.77 |
| wavelet-HHH_gldm_SmallDependenceLowGrayLevelEmphasis_32_binCount | 0.767 | 0.708 | 0.825 |
| wavelet-HHH_ngtdm_Busyness_32_binCount | 0.626 | 0.551 | 0.706 |
| wavelet-HHH_ngtdm_Coarseness_32_binCount | 0.94 | 0.92 | 0.957 |
| wavelet-HHH_ngtdm_Complexity_32_binCount | 0.598 | 0.522 | 0.681 |
| wavelet-HHH_ngtdm_Contrast_32_binCount | 0.789 | 0.733 | 0.842 |
| wavelet-HHH_ngtdm_Strength_32_binCount | 0.857 | 0.816 | 0.896 |
| wavelet-LLL_firstorder_10Percentile_32_binCount | 0.959 | 0.945 | 0.971 |
| wavelet-LLL_firstorder_90Percentile_32_binCount | 0.867 | 0.828 | 0.903 |
| wavelet-LLL_firstorder_Energy_32_binCount | 0.996 | 0.994 | 0.997 |
| wavelet-LLL_firstorder_Entropy_32_binCount | 0.809 | 0.757 | 0.858 |
| wavelet-LLL_firstorder_InterquartileRange_32_binCount | 0.897 | 0.865 | 0.925 |
| wavelet-LLL_firstorder_Kurtosis_32_binCount | 0.642 | 0.568 | 0.72 |
| wavelet-LLL_firstorder_Maximum_32_binCount | 0.805 | 0.753 | 0.856 |
| wavelet-LLL_firstorder_MeanAbsoluteDeviation_32_binCount | 0.845 | 0.801 | 0.887 |
| wavelet-LLL_firstorder_Mean_32_binCount | 0.95 | 0.933 | 0.964 |
| wavelet-LLL_firstorder_Median_32_binCount | 0.971 | 0.961 | 0.98 |
| wavelet-LLL_firstorder_Minimum_32_binCount | 0.812 | 0.761 | 0.861 |
| wavelet-LLL_firstorder_Range_32_binCount | 0.832 | 0.785 | 0.876 |
| wavelet-LLL_firstorder_RobustMeanAbsoluteDeviation_32_binCount | 0.885 | 0.85 | 0.917 |
| wavelet-LLL_firstorder_RootMeanSquared_32_binCount | 0.934 | 0.912 | 0.953 |
| wavelet-LLL_firstorder_Skewness_32_binCount | 0.677 | 0.606 | 0.75 |
| wavelet-LLL_firstorder_TotalEnergy_32_binCount | 0.996 | 0.994 | 0.997 |
| wavelet-LLL_firstorder_Uniformity_32_binCount | 0.818 | 0.768 | 0.866 |
| wavelet-LLL_firstorder_Variance_32_binCount | 0.737 | 0.674 | 0.801 |
| wavelet-LLL_glcm_Autocorrelation_32_binCount | 0.573 | 0.495 | 0.658 |
| wavelet-LLL_glcm_ClusterProminence_32_binCount | 0.609 | 0.533 | 0.691 |
| wavelet-LLL_glcm_ClusterShade_32_binCount | 0.419 | 0.345 | 0.51 |
| wavelet-LLL_glcm_ClusterTendency_32_binCount | 0.735 | 0.671 | 0.799 |
| wavelet-LLL_glcm_Contrast_32_binCount | 0.721 | 0.655 | 0.787 |
| wavelet-LLL_glcm_Correlation_32_binCount | 0.713 | 0.646 | 0.78 |
| wavelet-LLL_glcm_DifferenceAverage_32_binCount | 0.819 | 0.769 | 0.866 |
| wavelet-LLL_glcm_DifferenceEntropy_32_binCount | 0.822 | 0.773 | 0.869 |
| wavelet-LLL_glcm_DifferenceVariance_32_binCount | 0.641 | 0.567 | 0.719 |
| wavelet-LLL_glcm_Id_32_binCount | 0.838 | 0.792 | 0.881 |
| wavelet-LLL_glcm_Idm_32_binCount | 0.836 | 0.79 | 0.88 |
| wavelet-LLL_glcm_Idmn_32_binCount | 0.758 | 0.697 | 0.818 |
| wavelet-LLL_glcm_Idn_32_binCount | 0.824 | 0.775 | 0.87 |
| wavelet-LLL_glcm_Imc1_32_binCount | 0.829 | 0.782 | 0.874 |
| wavelet-LLL_glcm_Imc2_32_binCount | 0.847 | 0.802 | 0.888 |
| wavelet-LLL_glcm_InverseVariance_32_binCount | 0.675 | 0.604 | 0.749 |
| wavelet-LLL_glcm_JointAverage_32_binCount | 0.593 | 0.517 | 0.677 |
| wavelet-LLL_glcm_JointEnergy_32_binCount | 0.805 | 0.752 | 0.855 |
| wavelet-LLL_glcm_JointEntropy_32_binCount | 0.794 | 0.74 | 0.847 |
| wavelet-LLL_glcm_MCC_32_binCount | 0.604 | 0.528 | 0.687 |
| wavelet-LLL_glcm_MaximumProbability_32_binCount | 0.8 | 0.746 | 0.851 |
| wavelet-LLL_glcm_SumAverage_32_binCount | 0.593 | 0.517 | 0.677 |
| wavelet-LLL_glcm_SumEntropy_32_binCount | 0.797 | 0.743 | 0.849 |
| wavelet-LLL_glcm_SumSquares_32_binCount | 0.748 | 0.686 | 0.81 |
| wavelet-LLL_glrlm_GrayLevelNonUniformity_32_binCount | 0.986 | 0.981 | 0.99 |
| wavelet-LLL_glrlm_GrayLevelNonUniformityNormalized_32_binCount | 0.803 | 0.75 | 0.854 |
| wavelet-LLL_glrlm_GrayLevelVariance_32_binCount | 0.758 | 0.698 | 0.818 |
| wavelet-LLL_glrlm_HighGrayLevelRunEmphasis_32_binCount | 0.578 | 0.501 | 0.663 |
| wavelet-LLL_glrlm_LongRunEmphasis_32_binCount | 0.837 | 0.791 | 0.88 |
| wavelet-LLL_glrlm_LongRunHighGrayLevelEmphasis_32_binCount | 0.61 | 0.535 | 0.692 |
| wavelet-LLL_glrlm_LongRunLowGrayLevelEmphasis_32_binCount | 0.543 | 0.466 | 0.631 |
| wavelet-LLL_glrlm_LowGrayLevelRunEmphasis_32_binCount | 0.43 | 0.355 | 0.521 |
| wavelet-LLL_glrlm_RunEntropy_32_binCount | 0.68 | 0.61 | 0.753 |
| wavelet-LLL_glrlm_RunLengthNonUniformity_32_binCount | 0.93 | 0.908 | 0.95 |
| wavelet-LLL_glrlm_RunLengthNonUniformityNormalized_32_binCount | 0.875 | 0.838 | 0.909 |
| wavelet-LLL_glrlm_RunPercentage_32_binCount | 0.878 | 0.841 | 0.911 |
| wavelet-LLL_glrlm_RunVariance_32_binCount | 0.83 | 0.782 | 0.875 |
| wavelet-LLL_glrlm_ShortRunEmphasis_32_binCount | 0.869 | 0.83 | 0.905 |
| wavelet-LLL_glrlm_ShortRunHighGrayLevelEmphasis_32_binCount | 0.603 | 0.527 | 0.685 |
| wavelet-LLL_glrlm_ShortRunLowGrayLevelEmphasis_32_binCount | 0.451 | 0.374 | 0.542 |
| wavelet-LLL_glszm_GrayLevelNonUniformity_32_binCount | 0.927 | 0.903 | 0.948 |
| wavelet-LLL_glszm_GrayLevelNonUniformityNormalized_32_binCount | 0.659 | 0.587 | 0.735 |
| wavelet-LLL_glszm_GrayLevelVariance_32_binCount | 0.672 | 0.601 | 0.746 |
| wavelet-LLL_glszm_HighGrayLevelZoneEmphasis_32_binCount | 0.559 | 0.481 | 0.645 |
| wavelet-LLL_glszm_LargeAreaEmphasis_32_binCount | 0.811 | 0.76 | 0.86 |
| wavelet-LLL_glszm_LargeAreaHighGrayLevelEmphasis_32_binCount | 0.894 | 0.861 | 0.923 |
| wavelet-LLL_glszm_LargeAreaLowGrayLevelEmphasis_32_binCount | 0.505 | 0.427 | 0.595 |
| wavelet-LLL_glszm_LowGrayLevelZoneEmphasis_32_binCount | 0.398 | 0.324 | 0.488 |
| wavelet-LLL_glszm_SizeZoneNonUniformity_32_binCount | 0.627 | 0.553 | 0.707 |
| wavelet-LLL_glszm_SizeZoneNonUniformityNormalized_32_binCount | 0.737 | 0.673 | 0.8 |
| wavelet-LLL_glszm_SmallAreaEmphasis_32_binCount | 0.732 | 0.667 | 0.796 |
| wavelet-LLL_glszm_SmallAreaHighGrayLevelEmphasis_32_binCount | 0.582 | 0.505 | 0.667 |
| wavelet-LLL_glszm_SmallAreaLowGrayLevelEmphasis_32_binCount | 0.373 | 0.301 | 0.462 |
| wavelet-LLL_glszm_ZoneEntropy_32_binCount | 0.845 | 0.8 | 0.886 |
| wavelet-LLL_glszm_ZonePercentage_32_binCount | 0.885 | 0.85 | 0.917 |
| wavelet-LLL_glszm_ZoneVariance_32_binCount | 0.812 | 0.761 | 0.861 |
| wavelet-LLL_gldm_DependenceEntropy_32_binCount | 0.824 | 0.776 | 0.87 |
| wavelet-LLL_gldm_DependenceNonUniformity_32_binCount | 0.975 | 0.967 | 0.983 |
| wavelet-LLL_gldm_DependenceNonUniformityNormalized_32_binCount | 0.869 | 0.83 | 0.904 |
| wavelet-LLL_gldm_DependenceVariance_32_binCount | 0.87 | 0.831 | 0.905 |
| wavelet-LLL_gldm_GrayLevelNonUniformity_32_binCount | 0.955 | 0.94 | 0.968 |
| wavelet-LLL_gldm_GrayLevelVariance_32_binCount | 0.763 | 0.703 | 0.821 |
| wavelet-LLL_gldm_HighGrayLevelEmphasis_32_binCount | 0.581 | 0.504 | 0.666 |
| wavelet-LLL_gldm_LargeDependenceEmphasis_32_binCount | 0.87 | 0.831 | 0.905 |
| wavelet-LLL_gldm_LargeDependenceHighGrayLevelEmphasis_32_binCount | 0.737 | 0.674 | 0.801 |
| wavelet-LLL_gldm_LargeDependenceLowGrayLevelEmphasis_32_binCount | 0.525 | 0.447 | 0.614 |
| wavelet-LLL_gldm_LowGrayLevelEmphasis_32_binCount | 0.423 | 0.348 | 0.514 |
| wavelet-LLL_gldm_SmallDependenceEmphasis_32_binCount | 0.868 | 0.829 | 0.904 |
| wavelet-LLL_gldm_SmallDependenceHighGrayLevelEmphasis_32_binCount | 0.735 | 0.671 | 0.799 |
| wavelet-LLL_gldm_SmallDependenceLowGrayLevelEmphasis_32_binCount | 0.775 | 0.718 | 0.832 |
| wavelet-LLL_ngtdm_Busyness_32_binCount | 0.846 | 0.802 | 0.887 |
| wavelet-LLL_ngtdm_Coarseness_32_binCount | 0.946 | 0.929 | 0.962 |
| wavelet-LLL_ngtdm_Complexity_32_binCount | 0.704 | 0.636 | 0.773 |
| wavelet-LLL_ngtdm_Contrast_32_binCount | 0.759 | 0.699 | 0.819 |
| wavelet-LLL_ngtdm_Strength_32_binCount | 0.872 | 0.834 | 0.907 |
